# Explainable artificial intelligence on life satisfaction, diabetes mellitus and its comorbid condition

**DOI:** 10.1038/s41598-023-36285-z

**Published:** 2023-07-19

**Authors:** Ranyeong Kim, Chae-Won Kim, Hyuntae Park, Kwang-Sig Lee

**Affiliations:** 1 Department of Public Health Sciences, Graduate School of Korea University, Seoul, South Korea; 2 Interdisciplinary Program in Precision Public Health, Department of Public Health Sciences, Graduate School of Korea University, Seoul, South Korea; 3 AI Center, Korea University College of Medicine, 73 Inchon-Ro, Seongbook-Gu, Seoul, 02841 Korea; 4 School of Health & Environmental Science, Korea University, Seoul, Korea; 5 Department of Obstetrics and Gynecology, Korea University College of Medicine, 73 Inchon-Ro, Seongbook-Gu, Seoul, 02841 Korea

**Keywords:** Computational biology and bioinformatics, Diseases, Mathematics and computing

## Abstract

This study uses artificial intelligence for testing (1) whether the comorbidity of diabetes and its comorbid condition is very strong in the middle-aged or old (hypothesis 1) and (2) whether major determinants of the comorbidity are similar for different pairs of diabetes and its comorbid condition (hypothesis 2). Three pairs are considered, diabetes-cancer, diabetes-heart disease and diabetes-mental disease. Data came from the Korean Longitudinal Study of Ageing (2016–2018), with 5527 participants aged 56 or more. The evaluation of the hypotheses were based on (1) whether diabetes and its comorbid condition in 2016 were top-5 determinants of the comorbidity in 2018 (hypothesis 1) and (2) whether top-10 determinants of the comorbidity in 2018 were similar for different pairs of diabetes and its comorbid condition (hypothesis 2). Based on random forest variable importance, diabetes and its comorbid condition in 2016 were top-2 determinants of the comorbidity in 2018. Top-10 determinants of the comorbidity in 2018 were the same for different pairs of diabetes and its comorbid condition: body mass index, income, age, life satisfaction—health, life satisfaction—economic, life satisfaction—overall, subjective health and children alive in 2016. In terms of SHAP values, the probability of the comorbidity is expected to decrease by 0.02–0.03 in case life satisfaction overall is included to the model. This study supports the two hypotheses, highlighting the importance of preventive measures for body mass index, socioeconomic status, life satisfaction and family support to manage diabetes and its comorbid condition.

## Introduction

Diabetes mellitus, cancer, heart disease and mental disease are major parts of disease burden in the world. The global diabetes prevalence is predicted to rise from 425 to 693 million during 2017–2045^1^. Cancer, the second cause of global mortality in 2018, was estimated to be responsible for 9.6 million deaths globally^2^. Cardiovascular disease constituted the greatest part (32%) of global mortality in 2013, that is, 17 million of 54 million deaths^3^. Moreover, mental disease including depression is a major public health problem. For example, depression is a leading cause of disability in the world, affecting more than 350 million globally^4^. This global trend agrees with its Korean counterpart. Cancer, heart disease, suicide and diabetes were the first, second, fifth and sixth causes of death in Korea for the year 2018, i.e., 154.3, 62.4, 26.6 and 17.1 per 100,000, respectively^5^. Cancer (1681), unipolar depression (1508), ischemic heart disease (562) and diabetes (275) were the first, second, third and ninth causes of disability-adjusted life years per 100,000 in the nation for the year 2010^6^.

Then, do these diseases have a strong comorbidity (or association), i.e., diabetes, cancer, heart disease and mental disease? If so, what determines the comorbidity? A couple of previous studies examined these issues, even though they used different variables from this study. For example, one previous study reports that family support (children alive, marriage), socioeconomic status (education, income) and social activity (friendship activity) are major determinants of comorbidity among cerebrovascular disease, hearing loss and cognitive impairment in a middle-aged or old population in Korea and that comorbidity among the three diseases is very strong in the middle-aged or old^7,8^. Likewise, another previous study reports that family support (brothers/sisters cohabiting, parents alive), socioeconomic status (income) and social activity (voluntary activity, family activity, leisure activity, friendship meeting) are major determinants of comorbidity among diabetes mellitus, visual impairment and hearing loss in a middle-aged or old population in the nation^8,9^. These previous studies used Korean Longitudinal Study of Ageing (2014–2016) data and artificial intelligence models (the artificial/recurrent neural network) for testing the hypotheses on the comorbidity of the diseases and its major determinants.

This study is an extension of the framework above to comorbidity among diabetes, cancer, heart disease and mental disease in a middle-aged or old population. Moreover, we introduced the Shapley additive explanations (SHAP) values to analyze the direction of association between a major determinant and the comorbidity in the prediction model. To our best knowledge, this is one of the earliest endeavors to adopt a cutting-edge method of explainable artificial intelligence. This study is expected to have global implications, given that cancer, ischemic heart disease, depressive disorders and diabetes were top-4 causes of death or disability in the world for 2017–2018^1–4,10^. In this context, this study tests the following hypotheses from the literature above, considering three pairs of diabetes and its comorbid condition (diabetes-cancer, diabetes-heart disease and diabetes-mental disease):

### Hypothesis 1

The comorbidity of diabetes and its comorbid condition is very strong in the middle-aged or old.

### Hypothesis 2

Major determinants of the comorbidity are similar for different pairs of diabetes and its comorbid condition.

## Results

Descriptive statistics for participants’ categorical and continuous variables are shown in Tables S1 and S2 (Supplementary Tables), respectively. Among the 5527 participants in 2018, 1532 (27.7%) were diagnosed as diabetes and/or cancer, 1621 (29.3%) as diabetes and/or heart disease, and 1461 (26.4%) as diabetes and/or mental disease. Among the same participants in 2016, 1138 (20.6%), 344 (6.2%), 553 (10.0%) and 273 (4.9%) were characterized by the diagnosis of diabetes, cancer, heart disease and mental disease, respectively. On average, the age, monthly income and body mass index of the participant were 71, $1205 and 24, respectively. According to Table 1, the random forest, the recurrent neural network and logistic regression were the best models in terms of accuracy and the area under the receiver-operating-characteristic curve. Their accuracy measures were 0.9725, 0.9688 and 0.9720 for diabetes-cancer, 0.9776, 0.9703 and 0.9776 for diabetes-heart disease and 0.9797, 0.9711 and 0.9797 for diabetes-mental health, respectively. Likewise, their areas under the receiver-operating-characteristic curves were 0.9625, 0.9630 and 0.9625 for diabetes-cancer, 0.9725, 0.9747 and 0.9725 for diabetes-heart disease, and 0.9775, 0.9779 and 0.9800 for diabetes-mental health, respectively.

**Table 1:** Model performance.

Model	Diabetes-cancer		Diabetes-heart disease		Diabetes-mental disease	
	Accuracy	AUC	Accuracy	AUC	Accuracy	AUC
Logistic regression	0.9720	0.9625	0.9776	0.9725	0.9797	0.9800
Decision tree	0.9428	0.9250	0.9356	0.9325	0.9508	0.9300
Naive Bayes	0.7660	0.9575	0.9465	0.9650	0.8944	0.9650
Random forest	0.9725	0.9625	0.9776	0.9725	0.9797	0.9775
Support vector machine	0.7260	0.8250	0.7569	0.8250	0.7981	0.8225
Artificial neural network	0.8900	0.9675	0.7308	0.9750	0.8669	0.9850
Recurrent neural network	0.9688	0.9630	0.9703	0.9747	0.9711	0.9779

*AUC* area under the receiver-operating-characteristic curve.

**Figure 1:**
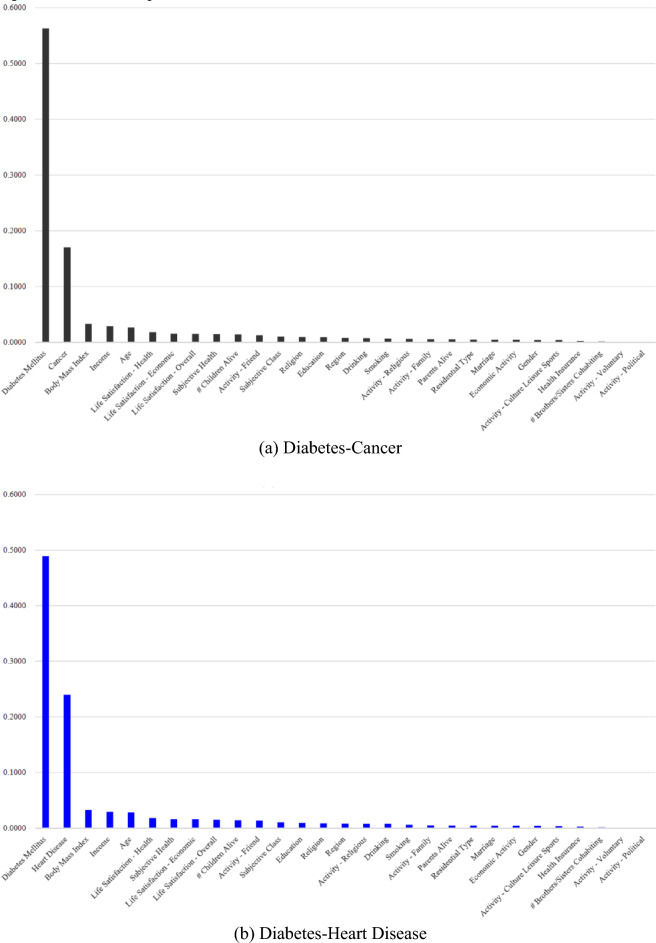

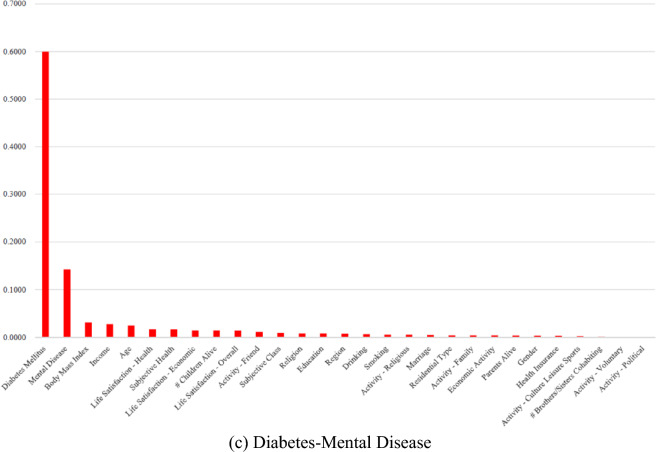
Variable Importance from the Random Forest. Diabetes and its comorbid condition in 2016 were top-2 determinants of the comorbidity in 2018 (This supports the hypothesis 1). Top-10 determinants of the comorbidity in 2018 were the same for different pairs of diabetes and its comorbid condition: body mass index, income, age, life satisfaction—health, life satisfaction—economic, life satisfaction—overall, subjective health and children alive in 2016 (This supports the hypothesis 2).

Based on variable importance from the random forest (Table 2, Fig. 1), diabetes and its comorbid condition in 2016 were top-2 determinants of the comorbidity in 2018 (This supports the hypothesis 1). Top-10 determinants of the comorbidity in 2018 were the same for different pairs of diabetes and its comorbid condition: body mass index, income, age, life satisfaction—health, life satisfaction—economic, life satisfaction—overall, subjective health and children alive in 2016 (This supports the hypothesis 2). The logistic regression results (Table S3, a supplementary table) provide useful information about the effect of a determinant on the comorbidity. For example, let’s consider the odds of having diabetes only compared to the odds of having no disease in 2018 (“Diabetes-Cancer YN” Column). This odds is 100 times as high for those with diabetes in 2016 as for those without the disease in 2016. This odds will increase by 3% if body mass index in 2016 increases by one unit.

**Table 2:** Top-10 variables for comorbidity among different chronic diseases.

Rank	Diabetes-cancer	Diabetes-heart disease	Diabetes-mental disease
*01*	Diabetes mellitus	Diabetes mellitus	Diabetes mellitus
*02*	Cancer	Heart disease	Mental disease
*03*	Body mass index	Body mass index	Body mass index
*04*	Income	Income	Income
*05*	Age	Age	Age
*06*	Life satisfaction—health	Life satisfaction—health	Life satisfaction—health
*07*	Life satisfaction—economic	Subjective health	Subjective health
*08*	Life satisfaction—overall	Life satisfaction—economic	Life satisfaction—economic
*09*	Subjective health	Life satisfaction—Overall	# Children alive
*10*	# Children alive	# Children alive	Life satisfaction—overall

**Figure 2:**
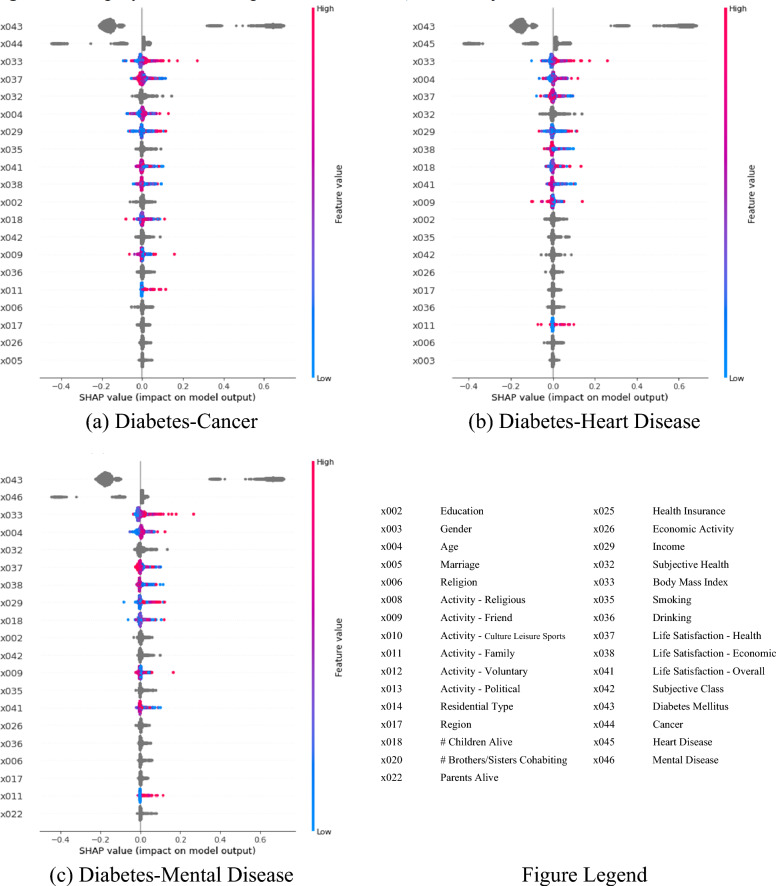
Shapley Additive Explanations (SHAP) Summary Plot from the Random Forest.: The SHAP value of a particular determinant for a particular observation measures a difference between what the model (the random forest) predicts for the probability of the comorbidity for the observation with and without the determinant. The SHAP values of body mass index (× 033) have the range of (− 0.09, 0.27), (− 0.10, 0.26) and (− 0.04, 0.27) for diabetes-cancer, diabetes-heart disease and diabetes-mental disease, respectively. There exists a strong positive association between body mass index and the comorbidity. Here, the probability of the comorbidity is expected to increase by 0.26–0.27 in case body mass index is included in the model.

**Figure 3:**
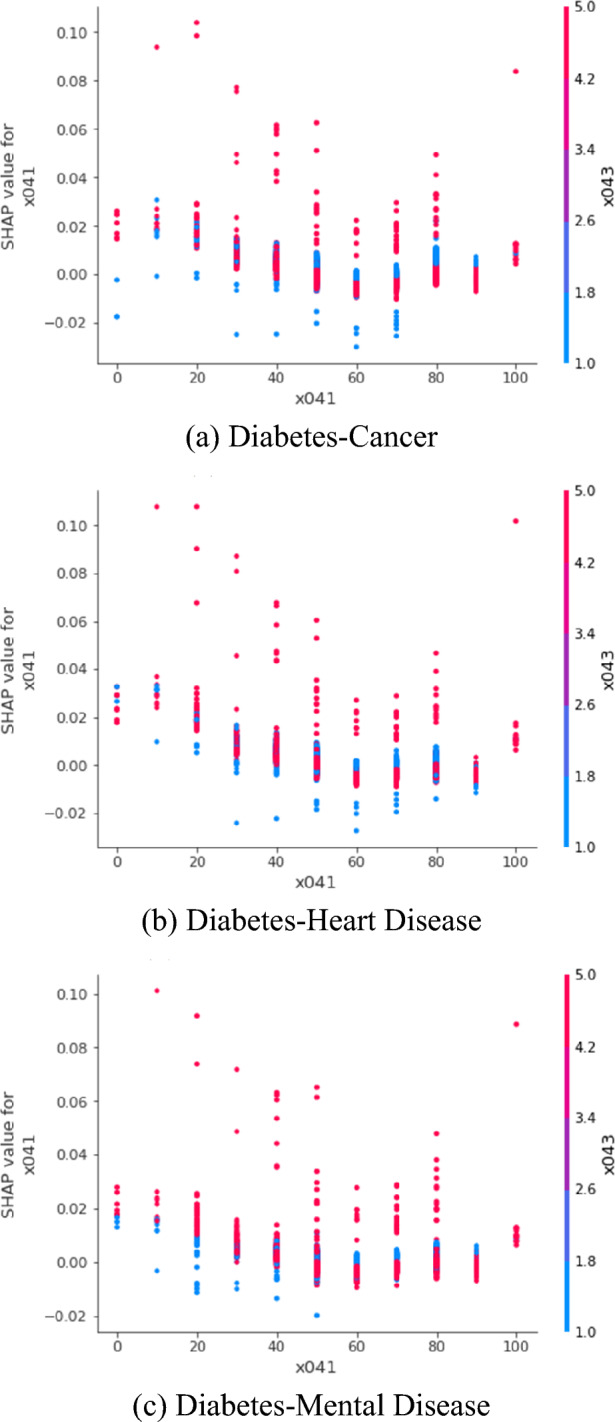
Shapley Additive Explanations (SHAP) Dependence Plot from the Random Forest: Life Satisfaction Overall with Diabetes. The SHAP dependence plot reveals an interaction between two determinants regarding their effects on the probability prediction of the comorbidity. The SHAP values of life satisfaction overall (× 041) are (1) lower for those with high life satisfaction overall and (2) higher for those with diabetes (× 043) in red dots. Here, the probability of the comorbidity is expected to decrease by 0.02–0.03 in case life satisfaction overall is included in the model.

SHAP values are shown in Table 3 and Figs. 2 and 3. Table 3 and Fig. 2 denote SHAP summary tables and plots, respectively. Figure 3 represents SHAP dependence plots. The SHAP value of a particular determinant for a particular observation measures a difference between what the model (the random forest) predicts for the probability of the comorbidity for the observation with and without the determinant. Indeed, the SHAP dependence plot reveals an interaction between two determinants regarding their effects on the probability prediction of the comorbidity. In Table 3 and Fig. 2, the SHAP values of body mass index (× 033) have the range of (− 0.09, 0.27), (− 0.10, 0.26) and (− 0.04, 0.27) for diabetes-cancer, diabetes-heart disease and diabetes-mental disease, respectively. There exists a strong positive association between body mass index and the comorbidity. Here, the probability of the comorbidity is expected to increase by 0.26–0.27 in case body mass index is included in the model. Based on Table 3 and Fig. 2, an association looks positive between life satisfaction overall (× 041) and the comorbidity as well. But Fig. 3a–c reveal the opposite pattern. In Fig. 3a–c, the SHAP values of life satisfaction overall (× 041) are (1) lower for those with high life satisfaction overall and (2) higher for those with diabetes (× 043) in red dots. Here, the probability of the comorbidity is expected to decrease by 0.02–0.03 in case life satisfaction overall is included in the model.

**Table 3:** Shapley additive explanations (SHAP) from the random forest.

	Variable	Diabetes-cancer		Diabetes-heart		Diabetes-mental	
		Min	Max	Min	Max	Min	Max
× 002	Education	− 0.0595	0.0620	− 0.0411	0.0678	− 0.0329	0.0571
× 003	Gender	− 0.0138	0.0294	− 0.0154	0.0285	− 0.0071	0.0266
× 004	Age	− 0.0760	0.1296	− 0.0642	0.1194	− 0.0514	0.1239
× 005	Marriage	− 0.0176	0.0476	− 0.0227	0.0399	− 0.1076	0.0425
× 006	Religion	− 0.0550	0.0528	− 0.0415	0.0490	− 0.0110	0.0575
× 008	Activity—religious	− 0.0555	0.1088	− 0.0757	0.1083	− 0.0292	0.1174
× 009	Activity—friend	− 0.0622	0.1587	− 0.1011	0.1413	− 0.0225	0.1655
× 010	Activity—culture leisure sports	− 0.0147	0.1226	− 0.0193	0.0993	− 0.0045	0.1056
× 011	Activity—family	− 0.0061	0.1165	− 0.0709	0.1011	− 0.0108	0.1143
× 012	Activity—voluntary	− 0.0147	0.0060	− 0.0134	0.0058	− 0.0015	0.0075
× 013	Activity—political	− 0.0002	0.0069	− 0.0002	0.0090	− 0.0005	0.0079
× 014	Residential type	− 0.0163	0.0245	− 0.0147	0.0232	− 0.0101	0.0260
× 017	Region	− 0.0256	0.0383	− 0.0240	0.0397	− 0.0105	0.0374
× 018	# Children alive	− 0.0798	0.1112	− 0.0328	0.1348	− 0.0608	0.1204
× 020	# Brothers/sisters cohabiting	− 0.0203	0.0978	− 0.0078	0.0942	− 0.0376	0.0904
× 022	Parents alive	− 0.0196	0.0867	− 0.0314	0.0808	− 0.0160	0.0813
× 025	Health insurance	− 0.0023	0.0400	− 0.0375	0.0542	− 0.0103	0.0518
× 026	Economic activity	− 0.0155	0.0425	− 0.0352	0.0455	− 0.0231	0.0434
× 029	Income	− 0.0677	0.1165	− 0.0650	0.1151	− 0.0818	0.1224
× 032	Subjective health	− 0.0512	0.1429	− 0.0626	0.1384	− 0.0320	0.1335
× 033	Body mass index	− 0.0930	0.2721	− 0.1011	0.2601	− 0.0351	0.2663
× 035	Smoking	− 0.0320	0.0918	− 0.0234	0.0761	− 0.0221	0.0762
× 036	Drinking	− 0.0250	0.0601	− 0.0267	0.0523	− 0.0097	0.0498
× 037	Life satisfaction—health	− 0.0531	0.1151	− 0.0777	0.0957	− 0.0237	0.1011
× 038	Life satisfaction—economic	− 0.0446	0.0952	− 0.0406	0.0999	− 0.0173	0.1147
× 041	Life satisfaction—overall	− 0.0299	0.1039	− 0.0273	0.1077	− 0.0198	0.1011
× 042	Subjective class	− 0.0340	0.0860	− 0.0555	0.0857	− 0.0122	0.0988
× 043	Diabetes mellitus	− 0.2139	0.6986	− 0.2027	0.6839	− 0.2252	0.7175
× 044	Cancer	− 0.4475	0.0395				
× 045	Heart disease			− 0.4234	0.0831		
× 046	Mental disease					− 0.4444	0.0356

## Discussion

In summary, (1) the comorbidity of diabetes and its comorbid condition is very strong in the middle-aged or old and (2) major determinants of the comorbidity are similar for different pairs of diabetes and its comorbid condition (body mass index, income, age, life satisfaction—health, life satisfaction—economic, life satisfaction—overall, subjective health and children alive). Three pairs are considered, diabetes-cancer, diabetes-heart disease and diabetes-mental disease. A few previous studies investigated these issues albeit with different dependent and independent variables from this study. One previous study reported that family support (children alive, marriage), socioeconomic status (education, income) and social activity (friendship activity) are major determinants of association among cerebrovascular disease, hearing loss and cognitive impairment in a middle-aged or old population in Korea and that association among the three diseases is very strong in the middle-aged or old^7^. Likewise, another previous study suggested that family support (brothers/sisters cohabiting, parents alive), socioeconomic status (income) and social activity (voluntary activity, family activity, leisure activity, friendship meeting) are major determinants of association among diabetes mellitus, visual impairment and hearing loss in a middle-aged or old population in the nation^9^.

To our best knowledge, this study is the first artificial-intelligence study to compare major determinants across three pairs of diabetes and its comorbid conditions. The largest cohort data in this line of research was obtained from the KLoSA (2016–2018) for 5527 subjects aged 56 or more. The random forest and the recurrent neural network registered remarkable performance in terms of the area under the receiver operating characteristic curve within the range of 96.3–97.8. Furthermore, we calculated the SHAP values to identify the direction of association between a major determinant and the comorbidity in the prediction model (random forest). To our best knowledge, this is one of the earliest achievements to introduce a cutting-edge approach of explainable artificial intelligence.

Specifically, three comments are available in the context of existing literature. Firstly, the results of this study are consistent with previous studies on social determinants of chronic diseases, requesting due attention to socioeconomic status (income) and family support (children alive)^7,9,11,12^. For example, a review study reports that family support is likely to reduce morbidity and mortality by improving cardiovascular, neuroendocrine and immune functions besides promoting health behavior and mental status^12^. Secondly, it was also found in this study that the comorbidity of diabetes and its comorbid condition is very strong in a middle-aged or old population. These findings suggest that preventive measures for diabetes and its comorbid condition should become central policy. Thirdly, this study sheds new light on the importance of body mass index and life satisfaction in managing diabetes and its comorbid conditions across board. Several analyses of national surveys in the United States reported a positive association between body mass index and diabetes: the Study to Help Improve Early evaluation and management of risk factors Leading to Diabetes 1994^13^; the National Health and Nutrition Examination Surveys 1999–2002^13^; the National Health Interview Survey 1997–2004^14^; the Medicare Current Beneficiary Survey 1991–2010^15^; and a case–control study using electronic health records in the Middle Atlantic region of the United States during 2004–2011^16^. However, no artificial-intelligence study was available to analyze a negative association of body mass index with three pairs of diabetes and its comorbid conditions. This study is the first investigation in this direction.

In a similar vein, a negative linkage was reported between life satisfaction and diabetes or mental disease in a few studies^17–20^. Moreover, several previous studies found that life satisfaction may played as a protective factor for cancer and heart disease^21–23^. To our best knowledge, however, no machine-learning examination has been available on the significance of life satisfaction in managing various pairs of diabetes and its comorbid conditions. Based on the findings of this study, life satisfaction is a major protective factor against diabetes and its comorbid conditions. Existing literature suggests two plausible mechanisms^24–26^. Firstly, life satisfaction has direct effects on behavioral and physiological systems^24^. It brings autonomic nervous system activation, which reduces the levels of the heart rate, blood pressure and stress-related hormones such as epinephrine and norepinephrine^24^. Moreover, life satisfaction causes hypothalamic–pituitary–adrenal (HPA) axis activation, which decreases cortisol and increases oxytocin and growth hormone: They play important roles in many physiological outcomes such as immune and inflammatory diseases^24^. Secondly, life satisfaction can aid in coping with stressors, thereby preventing unhealthy behavioral and physiological responses^24,25^. These indirect effects of life satisfaction are more apparent among those with greater social capital, i.e., greater social activity, bonding and network^26^. The discussion above requests due attention to the issue of life satisfaction, which should be a central part of clinical consultation for those with diabetes and its comorbid conditions. More comprehensive effort is need in this direction and this study would be a good starting point for further research.

However, this study had some limitations. Firstly, expanding the scope of this study to other chronic diseases and other determinants of comorbidity such as medication would add a great contribution to this line of research. Secondly, this study did not consider possible relationships or mediating effects among independent variables. Thirdly, sub-group analysis across age and gender (for example, men below 71, women below 71, men above 70, women above 70) would offer more insight on the major determinants of the comorbidity among the three diseases. Fourthly, the variables of life satisfaction and mental disease were measured based on single questions. This would be a good research topic to evaluate and improve their validity and reliability. Finally, different artificial intelligence methods would highlight different social determinants of chronic diseases but little study is available and more investigation is needed on this issue.

In conclusion, the comorbidity of diabetes and its comorbid condition is very strong in the middle-aged or old, and major determinants of the comorbidity are similar for different pairs of diabetes and its comorbid condition. Preventive measures for body mass index, socioeconomic status, life satisfaction and family support would be needed for the effective management of diabetes and its comorbid condition.

## Methods

### Participants and variables

The data source of this study was the Korean Longitudinal Study of Ageing (KLoSA) (2016–2018)^8^. This study did not require the approval of the ethics committee given that data were publicly available (https://survey.keis.or.kr/eng/klosa/klosa01.jsp) and de-identified. Among the 6618 participants, 1091 with missing values on any of three dependent variables and thirty one independent variables were deleted (piecewise deletion). The final sample of this study consisted of 5527 subjects aged 56 or more. The purpose of the KLoSA is to build a data source for the preparation of population aging in Korea. It is a good data source for artificial intelligence, given that its size is big and its quality is high enough for the great performance of artificial intelligence. Another desirable data source for artificial intelligence would be Korea National Health Insurance Service (KNHIS) data, which is designed to provide the socioeconomic qualification and medical utilization of all citizens in Korea (https://nhiss.nhis.or.kr/bd/ab/bdaba022eng.do). The KLoSA presents rich information on socioeconomic status and qualification of the old population, whereas the KNHIS data offers a variety of data on medical status and utilization of the entire population.

The KLoSA question on diabetes (or cancer/heart disease/mental disease) in 2016 and 2018 was “Since the last survey, have you ever been diagnosed by a doctor diabetes (or cancer/heart disease/mental disease)? 1. Yes. 5. No.” [C011 (or C016/C033/C043)]. The dependent variable, the comorbidity of diabetes and its comorbid condition in 2018, was divided into four categories: “0” for having no disease; “1” and “2” for having diabetes only and its comorbid condition only, respectively; and “3” for having both diseases. This study focuses on association among diseases as their comorbidity instead of complication. The independent variables were the following determinants in 2016^7,9^: (1) diabetes (no, yes) and its comorbid condition (no, yes); (2) demographic information, i.e., age, gender; (3) family support including children alive, brothers/sisters cohabiting, parents alive (father & mother, father, mother, none), marital status (married, separated, divorced, widowed, unmarried); (4) socioeconomic conditions such as educational level (elementary school or below, junior high school, senior high school, college or above), income (monthly, normalized between 0 and 1), health insurance (Medicare, Medicaid), economic activity (employed, unemployed); (5) social activity (monthly frequency), that is, religious, friendship, leisure, family, voluntary, political; (6) health-related information, i.e., subjective health (very good, good, middle [neither good nor poor], poor, very poor), body mass index, smoker (non, former, current), drinker (non, former, current); and (7) other determinants including region (big urban, small urban, rural), religion (non, Protestant, Catholic, Buddhist, Won-Buddhist, other), residential type (apartment, other), subjective class (high-A, high-B, middle-A, middle-B, low-A, low-B), life satisfaction—health (0–100), life satisfaction—economic (0–100) and life satisfaction—overall (0–100). The English version of the KLoSA questionnaire (2016–2018) is given as a supplementary file in this article.

### Analysis

Seven popular artificial intelligence approaches were compared for the prediction of the comorbidity: logistic regression, decision tree, naïve Bayes, random forest, support vector machine, artificial neural network, and recurrent neural network^7^. Data on 5527 participants were divided into training and validation sets with a 75:25 ratio (4145 vs. 1382 observations). Accuracy, a ratio of correct predictions among 1382 observations, was introduced as a criterion for validating the models trained. Variable importance from the random forest, an accuracy (or mean-impurity) gap between a complete model and a model excluding a certain variable, was used for testing the two hypotheses of this study. The evaluation of the hypothesis 1 was based on whether diabetes and its comorbid condition in 2016 were top-5 determinants of the comorbidity in 2018. The evaluation of the hypothesis 2 was based on whether top-10 determinants of the comorbidity in 2018 were similar for different pairs of diabetes and its comorbid condition. Finally, the SHAP values were calculated to analyze the direction of association between a major determinant and the comorbidity in the model (random forest). The SHAP value of a particular determinant for a particular observation measures a difference between what the model (the random forest) predicts for the probability of the comorbidity for the observation with and without the determinant.

In practice, experts in artificial intelligence use random forest variable importance to derive the rankings and values of all predictors for the prediction of the dependent variable. Then, they employ the SHAP plots to evaluate the directions of associations between the predictors and the dependent variable. Linear or logistic regression used to play this role before the SHAP approach took it over. This is because the SHAP approach has a notable strength compared to linear or logistic regression: the former considers all realistic scenarios, unlike the latter. Let us assume that there are three predictors of the comorbidity, i.e., diabetes, life satisfaction overall and age as in Fig. 3. As defined above, the SHAP value of diabetes for the comorbidity for a particular participant is the difference between what machine learning predicts for the probability of the comorbidity with and without diabetes for the participant. Here, the SHAP value for the participant is the average of the following four scenarios for the participant: (1) life satisfaction overall excluded, age excluded; (2) life satisfaction overall included, age excluded; (3) life satisfaction overall excluded, age included; and (4) life satisfaction overall included, age included. In other words, the SHAP value combines the results of all possible sub-group analyses, which are ignored in linear or logistic regression with an unrealistic assumption of *ceteris paribus*, i.e., “all the other variables staying constant”. Python 3.52 (Centrum voor Wiskunde en Informatica, Amsterdam, Netherlands) was employed for the analysis on November 2022.

### Ethics approval and consent to participate

This study did not require either the approval of the ethics committee or the informed consent of human subjects given that (1) data were publicly available (https://survey.keis.or.kr/eng/klosa/klosa01.jsp) and (2) data were de-identified (patient anonymity was preserved).

## Supplementary Information

Supplementary Information 1.

Supplementary Tables.

## Data Availability

The data used for this study are available from the Korean Longitudinal Study of Ageing (KLoSA) (https://survey.keis.or.kr/eng/klosa/klosa01.jsp).
