# Supplementary material for: Explainable artificial intelligence on life satisfaction, diabetes mellitus and its comorbid condition
Source: Sci Rep. 2023 Jul 19;13:11651. doi: 10.1038/s41598-023-36285-z (PMC10356918; doi:10.1038/s41598-023-36285-z)
Supplement: Supplementary file 1 — Supplementary Information 1. [file 41598_2023_36285_MOESM1_ESM.docx]

ㅊㅊ

**2018 KLoSA**

**Wave 7 Questionnaire**

SURVIVAL INTERVIEW

**TABLE OF CONTENTS**

[Table of contents]

- [**CV. COVERSCREEN** 2](file:///C:\BACK%20UP\Work\Part-Time%20(2016~2017)\한국고용정보원\2.%20Questionnaire\각%20차수%20Final%20Q\FINAL\2016년%206차%20고령화연구패널조사_0712.docx#_Toc487647331)
- [**A. DEMOGRAPHICS** 6](file:///C:\BACK%20UP\Work\Part-Time%20(2016~2017)\한국고용정보원\2.%20Questionnaire\각%20차수%20Final%20Q\FINAL\2016년%206차%20고령화연구패널조사_0712.docx#_Toc487647332)
- [**Ba. FAMILY AND FAMILY TRANSFER (Children & Grandchildren)** 15](file:///C:\BACK%20UP\Work\Part-Time%20(2016~2017)\한국고용정보원\2.%20Questionnaire\각%20차수%20Final%20Q\FINAL\2016년%206차%20고령화연구패널조사_0712.docx#_Toc487647333)
- [**Bb. FAMILY AND FAMILY TRANSFER (Parents & Siblings)** 33](file:///C:\BACK%20UP\Work\Part-Time%20(2016~2017)\한국고용정보원\2.%20Questionnaire\각%20차수%20Final%20Q\FINAL\2016년%206차%20고령화연구패널조사_0712.docx#_Toc487647334)
- [**C1. HEALTH STATUS** 78](file:///C:\BACK%20UP\Work\Part-Time%20(2016~2017)\한국고용정보원\2.%20Questionnaire\각%20차수%20Final%20Q\FINAL\2016년%206차%20고령화연구패널조사_0712.docx#_Toc487647335)
- [**C2. FUNCTIONAL LIMITATIONS AND HELPERS** 111](file:///C:\BACK%20UP\Work\Part-Time%20(2016~2017)\한국고용정보원\2.%20Questionnaire\각%20차수%20Final%20Q\FINAL\2016년%206차%20고령화연구패널조사_0712.docx#_Toc487647336)
- [**C3. HEALTH INSURANCE AND SERVICES** 11](file:///C:\BACK%20UP\Work\Part-Time%20(2016~2017)\한국고용정보원\2.%20Questionnaire\각%20차수%20Final%20Q\FINAL\2016년%206차%20고령화연구패널조사_0712.docx#_Toc487647337)8
- [**C4. COGNITION** 1](file:///C:\BACK%20UP\Work\Part-Time%20(2016~2017)\한국고용정보원\2.%20Questionnaire\각%20차수%20Final%20Q\FINAL\2016년%206차%20고령화연구패널조사_0712.docx#_Toc487647338)33
- [**C5. GRIP STRENGTH** 1](file:///C:\BACK%20UP\Work\Part-Time%20(2016~2017)\한국고용정보원\2.%20Questionnaire\각%20차수%20Final%20Q\FINAL\2016년%206차%20고령화연구패널조사_0712.docx#_Toc487647339)38
- [**D. EMPLOYMENT** 1](file:///C:\BACK%20UP\Work\Part-Time%20(2016~2017)\한국고용정보원\2.%20Questionnaire\각%20차수%20Final%20Q\FINAL\2016년%206차%20고령화연구패널조사_0712.docx#_Toc487647343)41
- [**E. INCOME AND CONSUMPTION** 2](file:///C:\BACK%20UP\Work\Part-Time%20(2016~2017)\한국고용정보원\2.%20Questionnaire\각%20차수%20Final%20Q\FINAL\2016년%206차%20고령화연구패널조사_0712.docx#_Toc487647362)19
- [**F. ASSETS AND DEBTS** 2](file:///C:\BACK%20UP\Work\Part-Time%20(2016~2017)\한국고용정보원\2.%20Questionnaire\각%20차수%20Final%20Q\FINAL\2016년%206차%20고령화연구패널조사_0712.docx#_Toc487647363)69
- [**G. EXPECTATIONS AND LIFE SATISFACTION** 31](file:///C:\BACK%20UP\Work\Part-Time%20(2016~2017)\한국고용정보원\2.%20Questionnaire\각%20차수%20Final%20Q\FINAL\2016년%206차%20고령화연구패널조사_0712.docx#_Toc487647364)3
- [**EXIT INTERVIEW** 32](file:///C:\BACK%20UP\Work\Part-Time%20(2016~2017)\한국고용정보원\2.%20Questionnaire\각%20차수%20Final%20Q\FINAL\2016년%206차%20고령화연구패널조사_0712.docx#_Toc487647365)5

# **CV. COVERSCREEN**

**Logic**

- **COVERSCREEN section is answered by one panel member in the household.**
- **Any panel member who has moved out or set up a separate household (distinguished in GPMS) is given a new household ID to indicate that fact.**
- **If a panel member has died, skip COVERSCREEN section and conduct an exit interview with a proxy respondent.**

CV001. Since 2006, we have been taking the KLoSA. This time, we conduct the KLoSA for the 6th wave. Thank you very much for your co-operation in this survey. You will be asked for the changes since the previous interview.

1. Continue

CV002. Then, can you please tell us your name?

____________________________________________________

CV003. What is your gender?

1. Male ⑤ Female

CV004. Are you currently residing in an apartment?

1. Yes ⑤ No

**Logic**

- **Loop CV005 ~CV011 as many times as the number of non-panel household members.**

[IWER: Ask each of the following questions per each of non-panel household members

CV005. What is his/her name?

[IWER: If R doesn’t know the name of non-panel household member, mark as a non-panel member 1, non-panel member 2, etc.]

____________________________________________________

CV006. What is [CV005_HH MEMBER’s name]’s gender?

1. Male ⑤ Female

CV007. What is [CV005_HH MEMBER’s name]’s relationship to you?

1. Father/mother →Go to CV009
2. Father‐in‐law/mother‐in‐law →Go to CV009
3. Spouse →Go to CV009
4. Son/daughter →Go to CV009
5. Son‐in‐law/daughter‐in‐law →Go to CV009
6. Brother/sister →Go to CV009
7. Grandchild →Go to CV009
8. Child of brother/sister →Go to CV009
9. Brother-in-law/sister-in-law →Go to CV009
10. Child of brother-in-law/sister-in-law →Go to CV009
11. Spouse of grandchild →Go to CV009
12. Great-grandchild →Go to CV009

[97] Others →Go to CV008

CV008. If others, please specify.

____________________________________________________

CV009. In what year was [CV005_HH MEMBER’s name] born?

_____________________________________________ [Range: 1900~2018]

CV010. What is [CV005_HH MEMBER’s name]’s current marital status?

1. Married or living with a spouse
2. Separated / Divorced / Widowed
3. Never married

**Logic**

- **If this is the loop for the non-panel house members newly added in HHSize_1, go to Total Check question.**

CV011. Is [CV005_HH MEMBER’s name] currently co-residing with you?

1. Yes ⑤ No

**Logic**

- **CV005 ~ CV011 Non-panel household member.**

CV012. Are there any other non-panel household member who newly moved in to your house since the previous survey?

1. Yes → HHSize_1 ⑤ No →Go to TotalCheck

HHSize_1. How many non-panel household member who newly moved in to your house since the previous survey? ______________________ →Go to CV005

TotalCheck. You just mentioned [the number of non-panel household member]. Are there other household members?

1. Yes, add the non-panel household member →Go to CV012 ⑤ No

CV012_sum. What is the total number of household members you currently live with, including all non-panel household members? Please tell the number of total household members including you.

______________________ [Range: 1 ~ 20]

CV012_gn. How many generations are included in the number of total household members you just mentioned at [CV012_sum]? ‘Generation’ means that ‘one generation is for a single person household or a couple household’, ‘two generation is for the parents with children’, and ‘three generation is for a grandparents, children and grandchildren’, etc.

| **Generation** | **Generation Composition** |
| --- | --- |
| 1. One generation | 11. Single-family household 12. Respondent + spouse [couple household] 13. Respondent (spouse) + brothers/sisters 14. Respondent (spouse) + other relatives 19. Other type of one generation |
| 2 Two generation | 21. Respondent (spouse) + children 22. Parent(s) + respondent (spouse) 23. Respondent (spouse) + children + brothers/sisters 24. Respondent (spouse) + children + other relatives 25. Respondent (spouse) + (great) grandchildren 29. Other type of two generation |
| 3. Three generation | 31. Parent(s) + respondent (spouse) + children 32. Parent(s) + respondent (spouse) + grandchildren 33. Respondent (spouse) + children + grandchildren 39. Other type of three generation |
| 4. Four generation | 41. Parent(s) + respondent (spouse) + children + grandchildren 42. Respondent (spouse) +children + grandchildren + great-grandchildren 49. Other type of four generation |
| 5. Others | 55. Non-relative household  (Other generation composition, such as when people who are not related to blood live together(ex. Co-workers, friends)) |

**Logic**

- **If CV013=⑤, then go to CV017. Loop CV013~CV016 until CV013=⑤.**

CV013. Have you moved since the previous interview?

[IWER: If the address does not change and the house that R is actually residing in has changed, mark as ‘Yes’.]

1. Yes ⑤ No →Go to CV017

CV014. When did you move? (unit: year and month combined into 6 digits)

[IWER:

- “If you have moved twice or more, please start with the most recent one.”

- Enter the year and month using 6 digits. For example, mark 201801 for January 2018. If the month is not clear, enter 201800.]

___________________________________ [Range: 190000~survey year]

CV015. Why did you move?

1. Because of the transfer of business/ workplace of the respondent or respondent’s spouse
2. Because of the transfer of business/ workplace of the other family member than the respondent or respondent’s spouse
3. Because of the education for children or grandchildren
4. For the better residential environment
5. To sell up the housing and to invest that money in other assets
6. To sell up the housing and spend that money on living expenses
7. (In case of deposit-based rental house or monthly rental house) Due to the expiration of the contract

[97] Others →Go to CV016

For code 1~7, Go to CV017

CV016. If others, please specify.

____________________________________________________

CV017. [IWER] Please enter code 1 for completion of cover screen section.

1. Completion

# **A. DEMOGRAPHICS**

(K1) What is your gender?

1. Male ⑤ Female

(K2) Where do you live in?

____________________________________________________

[Check the date]

(DC1) Let me check the date first. Is [date appeared in the computer] correct for today?

1. Yes →A_Intro ⑤ No

(DC2) If no, what date is it today?

_________________Year____Month___Day

(A_Intro) [IWER] Now, this is to start A.DEMOGRAPHICS section.

1. Check

A001. First, I will confirm the basic information you provided in the previous interview. Is your date of birth (yyyy/mm/dd)?

[IWER: The date of birth is based on the actual date of birth not the one stated in the resident registration card or family register.]

1. Yes →Go to A004 ⑤ No

A002. If no, please tell me your exact date of birth.

[IWER: For those who respond by a zodiac sign, refer to the index card provided.]

_________________Year____Month___Day

A003. Is your date of birth according to the solar calendar or the lunar calendar?

1. Solar calendar ⑤ Lunar calendar

(A001_Intro) [IWER] Check the age of R: [(2018) – (date of birth) + 1]

1. Check

**< Level of Education >**

A004. In the previous interview you responded that you had completed [level of education] and [graduation status]. Is this correct?

1. Yes → Go to A008 ⑤ No

A005. If no, can you please tell us the highest level of education?

1. No formal education (illiterate) →Go to A008
2. No formal education (literate) →Go to A008
3. Elementary school →Go to A007
4. Middle school (or equivalent qualification exam) →Go to A007
5. High school (or equivalent qualification exam) →Go to A007
6. Professional/ vocational school/ college →Go to A007
7. University →Go to A007
8. Graduate school (Master degree) →Go to A007
9. Post college (PhD) →Go to A007

[97] Others

[98] Don’t know →Go to A011

[99] Refuse to answer →Go to A011

A006. If others, please specify.

________________________→Go to A008

A007. For the school you mentioned, did you graduate, drop out or obtain an equivalent qualification by passing the qualification exam though not completing the school? Please answer only about the qualification you have completed through graduation or the qualification exam except the one you are now attending.

1. Currently attending
2. Graduated
3. Dropped out
4. Completed
5. Obtained an equivalent qualification through the qualification exam

A008. Have you attained further formal education including equivalent qualification exams since the previous interview?

[IWER: In case of ‘currently attending’, choose ‘⑤ No’ because R has not earned a degree yet.]

1. Yes ⑤ No →Go to A011

A009. What is the level of formal education you have attained?

1. Elementary school →Go to A011
2. Middle school (or equivalent qualification exam) →Go to A011
3. High school (or equivalent qualification exam) →Go to A011
4. Professional/ vocational school/ college →Go to A011
5. University →Go to A011
6. Graduate school (Master degree) →Go to A011
7. Post college (PhD) →Go to A011

[97] Others

[98] Don’t know →Go to A011

[99] Refuse to answer →Go to A011

A010. If others, please specify.

________________________

**< Marital Status >**

A011. In the previous interview you responded that your marital status was [marital status]. Is this correct?

[IWER: This question is intended to check R’s marital status at the time of the previous interview, not now.]

1. Yes →Go to A013 ⑤ No

A012. If no, can you tell us your exact marital status at that time?

1. Married or living with a partner
2. Separated
3. Divorced
4. Widowed or missing (dispersed family)
5. Never married

**Logic**

- **If A013=①, then go to A014. Loop A013~A015 until A013=⑤.**

A013. Has your marital status changed since the previous interview?

[IWER: Marital status includes divorce, separation by death, separation, marriage, etc.]

1. Yes ⑤ No →Go to A016

A014. How has it changed? Please describe all the changes in a time sequence.

[IWER: “For example, if you got divorced and then married again, please say divorce first.”]

1. Married
2. Separated
3. Divorced
4. Widowed

A015. When did you get [response in A014]? (unit: year and month combined into 6 digits)

[IWER: Enter the year and month using 6 digits. For example, mark 201801 for January 2018. If the month is not clear, enter 201800.]

________________________________________ [Range: 200600~201811]

**Logic**

- **If a respondent has a spouse now, then go to A016.**

A016. Did your current spouse participate in the previous KLoSA survey?

1. Yes →Go to A030
2. No →Go to A017 (In case the same spouse with the previous interview)

A018 (In case the new spouse)

**< Spouse >**

**Logic**

- **A017~A029 are to get basic information on R’s spouse and only for the case that the spouse is currently married to R and non-panel.**
- **If R’s spouse is a panel member, then go to A030.**

A017. Is your spouse’s name [spouse’s name]?

1. Yes →Go to A019 ⑤ No

A018. If no, what is your spouse’s name?

[IWER: if the R does not want to tell the spouse’s name, just write on ‘a spouse’.]

_____________________________________________________

A019. Is your spouse’s year of birth [year of birth]?

[IWER:

- For those who respond by a zodiac sign, refer to the index card provided

- If there is no previous information on this, mark ⑤ No, then ask A020]

1. Yes →Go to A021 ⑤ No →Go to A020

**Logic**

- **If R has the same spouse now as at the time of the previous interview, then ask A020~A029. If R has a new spouse, then ask A020, A022~A024 and A028~A029.**

A020. If no, please tell me your spouse [spouse’s name]’s exact year of birth again.

I am going to ask about your spouse [spouse’s name]’s year of birth and level of education. What year was your spouse born?

________________________________________ [Range: 1900~1990]

**Logic**

- **If R has the same spouses now as at the time of the previous interview, then ask A021. If R has a new spouse, then ask A022**

A021. You responded that your spouse [spouse’s name] had completed [level of education] and [graduation status]. Is this correct?

[IWER: if there is no previous information on this, mark ⑤ No, then ask A022]

1. Yes →Go to A025 ⑤ No

A022. If no, can you please tell us the highest level of education of your spouse?

1. No formal education (illiterate) →Go to A025
2. No formal education(literate) →Go to A025
3. Elementary school →Go to A024
4. Middle school (or equivalent qualification exam) →Go to A024
5. High school (or equivalent qualification exam) →Go to A024
6. Professional/ vocational school/ college →Go to A024
7. University →Go to A024
8. Graduate school (Master degree) →Go to A024
9. Post college (PhD) →Go to A024

[97] Others

[98] Don’t know →Go to A025

[99] Refuse to answer →Go to A025

A023. If others, please specify.

________________________→Go to A025

A024. For the school you mentioned did your spouse graduate, drop out or obtain an equivalent qualification by passing the qualification exam though not completing the school?

1. Currently attending
2. Graduated
3. Dropped out
4. Completed
5. Obtained an equivalent qualification through the qualification exam

**Logic**

- **If R has the same spouse now as the previous interview, then ask A025.**
- **If R has a new spouse, then go to A028.**

A025. Has your spouse attained further formal education since the previous interview?

1. Yes ⑤ No →Go to A028

A026. What is the level of formal education your spouse has attained?

1. Elementary school →Go to A028
2. Middle school (or equivalent qualification exam) →Go to A028
3. High school (or equivalent qualification exam) →Go to A028
4. Professional/ vocational school/ college →Go to A028
5. University →Go to A028
6. Graduate school (Master degree) →Go to A028
7. Post college (PhD) →Go to A028

[97] Others

[98] Don’t know →Go to A028

[99] Refuse to answer →Go to A028

A027. If others, please specify.

________________________

A028. Now I would like to know about your spouse’s current employment status. Please answer if your spouse is currently employed, temporarily laid off, looking for work, retired, or has never been employed. If she/he is employed, please answer if she/he works full time or part time.

[IWER: Retirement refers to having stopped income-earning activities and currently not working or engaging only in pastime work. Also, she/he doesn’t have intention to do anything more serious than pastime work as long as there is no special change in circumstances.]

1. Full-time worker →Go to A030
2. Part-time worker →Go to A030
3. Temporarily laid off on sick or other leave →Go to A030
4. Unemployed and looking for work →Go to A030
5. Retired →Go to A030
6. Haven’t worked after marriage →Go to A030

[97] Others

A029. If others, please specify.

__________________________

**< Religion and Social Relationship and Social Activity >**

A030. If any, what is your religion?

1. No religion →Go to A032
2. Protestantism →Go to A032
3. Catholicism →Go to A032
4. Buddhism →Go to A032
5. Won Buddhism →Go to A032
6. Others

A031. If others, please specify.

__________________________

A032. Do you have any friends or relatives who live nearby and have a close relationship with you? If so, how often do you meet them in person?

1. Almost every day (more than 4 times per week)
2. Once a week
3. 2‐3 times a week
4. Once a month
5. Twice a month (every two weeks)
6. Once or twice a year
7. Three or four times a year (once every three or four months)
8. Five or six times a year (every two months)
9. Almost never a year
10. No close friend or relative

A033. Are you a member of any of the following organizations, clubs, or societies?

Choose all that apply.

1. Religious groups →Go to A035
2. Social clubs (e.g. private savings club, senior citizens’ club, etc.) →Go to A035
3. Leisure/culture/sports related groups (class for the elderly, etc.)→Go to A035
4. Alumni associations, hometown communities, family councils →Go to A035
5. Volunteer groups →Go to A035
6. Political parties, NGOs, interest groups →Go to A035
7. Others
8. None →Go to A036

[98] Don’t know →Go to A036

[99] Refuse to answer →Go to A036

A034. If others, please specify.

________________________________

**Logic**

- **Loop A035 as many times as the number chosen in A033.**

A035. As for the organization [chosen in A033] of which you are a member, how often do you engage in its activities?

1. Almost every day (more than 4 times per week)
2. Once a week
3. 2-3 times a week
4. Once a month
5. Twice a month (every two weeks)
6. Once or twice a year
7. Three or four times a year (once every three or four months)
8. Five or six times a year (every two months)
9. Almost never a year
10. Almost never engaged

A036. Are you the household head?

1. Yes →Go to A037 ⑤ No →Go to A036_1

A036_1. Then, who is the household head in your family?

1. Spouse
2. Parents-in-law
3. Children who are currently single
4. Children who are currently married
5. Brother/ sister
6. Grandchildren
7. Grand parents
8. Others →Go to A036_2

For code 1~7, go to A037

A036_2. If others, please specify.

_______________________

A037. [IWER: How often did R receive assistance in answering section A-DEMOGRAPHICS?]

1. Never → Go to section Ba.
2. A few times → Go to section Ba.
3. Most of the time → Go to section Ba.
4. The section was done by a proxy respondent

A038. If done by a proxy respondent, what is the proxy’s relationship to R? Please answer in view of the proxy

1. Spouse
2. Mother
3. Father
4. Mother-in-law
5. Father-in-law
6. Brother/sister
7. Brother-in-law/sister-in-law
8. Son/daughter
9. Son-in-law/Daughter-in-law
10. Grandchild
11. Other relatives
12. Helper or other non-relatives

→ Go to section Ba.

(A_end) [IWER] Please enter code 1 for completion of demographic section.

1. Completion

# **Ba. FAMILY AND FAMILY TRANSFER**

**(CHILDREN & GRANDCHILDREN)**

(Ba_Intro) [IWER] Now, this is to start Ba. FAMILY (CHILDREN & GRANDCHILDREN) section.

1. Check

**Logic**

- **In regards to the number of children, if R answered ‘Refuse to answer/Don’t know’ in the previous interview, then ask Ba003. Others, ask Ba001.**
- **Among a couple, only the representative respondent for CHILDREN section is asked Ba001~Ba017.**

Ba001. Now I am going to ask you about your children. In the previous interview, you responded that you had [no. of children] children. Is this correct?

[IWER: According to the previous interview, if the number of children is same but member of children is different, check ‘⑤ No’]

1. Yes →Go to Ba003 ⑤ No

Ba002. How many children did you have at the time of the previous interview?

_______________________________ (unit: number of children)

Ba003. How many children are currently alive? (unit: number of children)

[IWER: Any child, who is not confirmed dead since he/she is missing or has run away from home, etc., is considered currently alive.]

_______________________________(unit: number of children)

→Go to Ba004 (If Ba001=① and the number of children in the previous interview > Ba003)

→Go to Ba006 (If Ba001=⑤ or ‘Refuse to answer’/‘Don’t know’ about the number of children in the previous interview, or if the number of children has increased)

→Go to Ba008 (If Ba001=① and the number of children in the previous interview=Ba003)

→Go to Ba072 (If the number of children in the previous interview=0 and Ba003=0)

Don’t know →Go to Ba072

Refuse to answer →Go to Ba072

**Logic**

- **If the number of children in the previous interview > Ba003, then ask Ba004.**

Ba004. Who are those dead children? Please list all of them. (Select multiple responses.)

_______________________________01~10 [child's name]

**Logic**

- **Loop Ba005 as many times as the number of children in Ba004.**

Ba005. When did [child's name selected in Ba004] die? (unit: year and month combined into 6 digits) [IWER: Enter the year and month using 6 digits. For example, mark 201801 for January 2018. If the month is not clear, enter 201800.]

_______________________________ [Range: 200600~survey year and month]

**< Basic information on children >**

**Logic: If any of following cases is applied, start with Ba006. Otherwise, start with Ba008**

1. **If (Ba001=⑤ and Ba003>=1)**
2. **If R answered ‘Don’t know/Refuse to answer’ about the number of children in the previous interview and Ba003>=1**
3. **If the number of children has increased since the previous interview**

**Logic:**

- **Loop as many times as the number of children reported in either Ba003 or [Ba003 - (number of children in the previous interview)].**

Ba006. What is your child's name? (Please start with the eldest, in birth order)

[IWER: Please double check the spelling of the name, if the same name is used in OVERSCREEN. If R does not provide the child’s name, describe him/her as ‘the first child’, ‘the second child’, etc.]

_______________________________

Ba007. Is [child's name] a son or daughter?

1. Son ⑤ Daughter

**Logic: Loop as many times as the number of children.**

Ba008. How old is [child's name]? (unit: an age)

[IWER:

- For those who respond by a zodiac sign, refer to the index card provided.

- Current age based on the response in the previous interview (year/month/day): [child’s age] years old]

_______________________________ years old

Ba009. What is the highest level of education [child's name] has completed?

[IWER: Response in the previous interview (year/month/day): [child’s level of education and graduation status]]

1. No formal education (illiterate) →Go to Ba012
2. No formal education (literate) →Go to Ba012
3. Elementary school →Go to Ba011
4. Middle school (or equivalent qualification exam) →Go to Ba011
5. High school (or equivalent qualification exam) →Go to Ba011
6. Professional/ vocational school/ college →Go to Ba011
7. University →Go to Ba011
8. Graduate school (Master degree) →Go to Ba011
9. Post college (PhD) →Go to Ba011

[97] Others

Ba010. If others, please specify.

[IWER: Response in the previous interview (year/month/day): [child’s level of education and graduation status]

_______________________________

Ba011. Did he/she graduate, drop out or obtain an equivalent qualification by passing the qualification exam though not completing the school?

[IWER: Response in the previous interview (year/month/day): [child’s level of education and graduation status]]

1. Currently attending
2. Graduated
3. Dropped out
4. Completed
5. Obtained an equivalent qualification through the qualification exam

Ba012. Does [child's name] work for pay?

[IWER: Response in the previous interview: whether the child works for pay]

1. Yes ⑤ No

Ba013. Is [child's name] currently co-residing with you?

[IWER: If the child lives away from home due to study or work, he/she is not co-residing with R.

Response in the previous interview: co-residing/not co-residing]

1. Yes ⑤ No

Ba014. At the time of the previous interview, you [were /weren’t co-residing] with [child's name].

If you lived with your child in any month in the last calendar year (2017), how many months did yo live with your child last year?

[IWER: If R didn’t live together with child last year, mark ‘0’]

_______________________________ months [Range:0~12]

Ba015. Does [child's name] own a house?

[IWER: Response in the previous interview: [whether the child owns a house]]

1. Yes ⑤ No

**Logic**

- **If the child is aged 15 or over, then ask Ba016~Ba017.**

Ba016. What is [child's name]’s current marital status?

[IWER: Response in the previous interview: [child’s marital status]]

1. Married or living with a partner
2. Separated
3. Divorced
4. Widowed or missing (dispersed family)
5. Never married

(8) Don’t know

(9) Refuse to answer

Ba017. Does [child's name] have any children? If so, how many?

[IWER: Response in the previous interview: [number of children] persons]]

_______________________________ [Range: 0~10]

**< Every children who do not live together: Distance, frequency of contact, transfer >**

**< All regardless of age >**

**Logic**

- **If Ba013=⑤, then ask Ba018~Ba020. Ask to each member of the couple.**

Ba018. [Not live together_ask to each member of the couple] How close does [child's name] live to you?

1. Within a 30‐minute distance by public transportation
2. Within a 1‐hour distance by public transportation
3. Within a 2‐hour distance by public transportation
4. More than a 2‐hour distance by public transportation

Ba019. [Not live together_ask to each member of the couple] How often do you meet [child's name] in person?

1. Almost every day (more than 4 times per week)
2. Once a week
3. 2~3 times a week
4. Once a month
5. Twice a month (every two weeks)
6. Once or twice a year
7. Three or four times a year (once every three or four months)
8. Five or six times a year (every two months)
9. Almost never a year
10. Never met

Ba020. [Not live together_ask to each member of the couple] How often do you contact [child's name] by phone, mail or e-mail?

1. Almost every day (more than 4 times per week)
2. Once a week
3. 2~3 times a week
4. Once a month
5. Twice a month (every two weeks)
6. Once or twice a year
7. Three or four times a year (once every three or four months)
8. Five or six times a year (every two months
9. Almost never a year
10. Never contacted

**Logic**

- **Among a couple, only the representative respondent for children section is asked Ba021~Ba072.**
- **If R did not live with his/her child for more than one month in 2017, go to Ba021~Ba056.**

Ba021. [Not live together_Receipt of regular financial support] In the last calendar year (2017), while living separately did you and your spouse receive from [child’s name] any regular financial support such as pocket money, living expenses or medical expenses?

1. Yes →Go to Ba022 ⑤ No →Go to Ba029

| **[**IWER**]**  **<Regular support/ occasional support>**   - Regular support: monetary transfer regularly repeated in a certain time interval (e.g. each month, every two months) - Occasional support: monetary transfer irregularly occurred except birthday allowance or holiday allowances (e.g. paying for medical bills, schooling, occasional allowances)   **<Financial support/ Non-financial support>**   - Financial support: receiving cash or helping pay bills, or covering specific costs for medical care, insurance, schooling, down payment for a home or rent, etc. - Non-financial support: gifts or goods (e.g. food, clothes etc.) received |
| --- |

Ba022. [Not live together_ Receipt of regular financial support] What was the monthly average amount of regular financial support such as pocket money, living expenses, medical expenses etc., that you received from [child’s name] in the last calendar year (2017)? (unit: 10,000 Korean won)

[IWER: For example, if R received 1,500,000 Korean won from your child while you were hospitalized for 3 months in 2017, mark it as 500,000 won = 50 MW.]

_______________________________ MW [Range: 1 ~ 9997] →Go to Ba028

Don’t know →Go to Ba023~Ba027 unfolding

Refuse to answer →Go to Ba023~Ba027 unfolding

| **< Notes on unfolding bracket questions>**   - When R says “don’t know” or “refuse to answer” to financial questions, R is randomly given the certain amount and then is asked whether the amount is “less than,” “about equal to,” or “more than” - There are 5 thresholds for unfolding questions: 1, 2, 3, 4 and 5, in increasing order - R is asked a maximum of 3 questions, presented in the following 3 scenarios - R is randomly assigned to 1 of these 3 scenarios - If R answers the amount during the interview, then go back to Ba022 and record |
| --- |

**<Ba023~Ba027. Unfolding bracket questions >**

[IWER: Go back to Ba022 and record if R answers the amount during the interview.]

Ba023. Did it amount to less than, about equal to or more than 10 MW (10,000 Korean won)?

1. Less than 10 MW
2. About 10 MW

⑤ More than 10 MW

Ba024. Did it amount to less than, about equal to or more than 30 MW (10,000 Korean won)?

1. Less than 30 MW
2. About 30 MW
3. More than 30 MW

Ba025. Did it amount to less than, about equal to or more than 50 MW (10,000 Korean won)?

1. Less than 50 MW
2. About 50 MW
3. More than 50 MW

Ba026. Did it amount to less than, about equal to or more than 100 MW (10,000 Korean won)?

1. Less than 100 MW
2. About 100 MW

⑤ More than 100 MW

Ba027. Did it amount to less than, about equal to or more than 200 MW (10,000 Korean won)?

1. Less than 200 MW
2. About 200 MW
3. More than 200 MW

**Logic**

- **If Ba028 > (12 – Ba014), do hard checking.**

Ba028. [Not live together_ Receipt of regular financial support] How many month(s) did you receive any financial support from [child’s name] in the last calendar year (2017)? (unit: month)

[IWER: If you alternated between co-residing and not co-residing in 2017, please consider only the financial support you received while you did not live together]

_______________________________ month [Range: 0~12]

Ba029. [Not live together_ Receipt of occasional financial support] In the last calendar year (2017), while living separately did you and your spouse receive from [child’s name] any occasional financial support such as pocket money, living expenses or medical expenses?

1. Yes →Go to Ba030 ⑤ No →Go to Ba036

| **[**IWER**]**  **<Regular support/ occasional support>**   - Regular support: monetary transfer regularly repeated in a certain time interval (e.g. each month, every two months) - Occasional support: monetary transfer irregularly occurred except birthday allowance or holiday allowances (e.g. paying for medical bills, schooling, occasional allowances)   **<Financial support/ Non-financial support>**   - Financial support: receiving cash or helping pay bills, or covering specific costs for medical care, insurance, schooling, down payment for a home or rent, etc. - Non-financial support: gifts or goods (e.g. food, clothes etc.) received |
| --- |

Ba030. [Not live together_ Receipt of occasional financial support] What was the total amount of occasional financial support such as pocket money, living expenses, medical expenses etc., that you received from [child’s name] in the last calendar year (2017)?

[IWER: Please enter ‘100,000,000 Korean won (one hundred million won)’ first then, enter ’10,000 Korean won (MW)’.]

_______one hundred million won [Range: 1~9] _____MW [Range: 0 ~ 9997]

Don’t know →Go to Ba031~Ba035 unfolding

Refuse to answer →Go to Ba031~Ba035 unfolding

**< Ba031~Ba035. Unfolding bracket questions >**

[IWER: Go back to Ba030 and record if R answers the amount during the interview.]

Ba031. Did it amount to less than, about equal to or more than 100 MW (10,000 Korean won)?

1. Less than 100 MW
2. About 100 MW

⑤ More than 100 MW

Ba032. Did it amount to less than, about equal to or more than 300 MW (10,000 Korean won)?

1. Less than 300 MW
2. About 300 MW

⑤ More than 300 MW

Ba033. Did it amount to less than, about equal to or more than 500 MW (10,000 Korean won)?

1. Less than 500 MW
2. About 500 MW
3. More than 500 MW

Ba034. Did it amount to less than, about equal to or more than 1,000 MW (10,000 Korean won)?

1. Less than 1,000 MW
2. About 1,000 MW
3. More than 1,000 MW

Ba035. Did it amount to less than, about equal to or more than 2,000 MW (10,000 Korean won)?

1. Less than 2,000 MW
2. About 2,000 MW
3. More than 2,000 MW

Ba036. [Not live together_ Receipt of non-financial support] In the last calendar year (2017), while living separately did you and your spouse receive from [child’s name] any gifts or goods (not cash)?

1. Yes →Go to Ba037 ⑤ No →Go to Ba039

Ba037. [Not live together_ Receipt of non-financial support] Then, what was the type of non‐financial support you received from [child’s name] in the last calendar year (2017)? (Select multiple responses.)

1. Leisure (e.g. travel) →Go to Ba039
2. Health‐related products (e.g. vitamins, equipment, etc.) →Go to Ba039
3. Household items →Go to Ba039
4. Electronics →Go to Ba039
5. Dining out and food →Go to Ba039
6. Clothes and shoes →Go to Ba039
7. Others →Go to Ba038

Ba038. If others, please specify.

_______________________________

Ba039. [Not live together_Offer of regular financial support] In the last calendar year (2017), while living separately did you and your spouse give to [child’s name] any financial support such as pocket money, living expenses or medical expenses?

1. Yes →Go to Ba040 ⑤ No →Go to Ba047

| **[**IWER**]**  **<Regular support/ occasional support>**   - Regular support: monetary transfer regularly repeated in a certain time interval (e.g. each month, every two months) - Occasional support: monetary transfer irregularly occurred except birthday allowance or holiday allowances (e.g. paying for medical bills, schooling, occasional allowances)   **<Financial support/ Non-financial support>**   - Financial support: receiving cash or helping pay bills, or covering specific costs for medical care, insurance, schooling, down payment for a home or rent, etc. - Non-financial support: gifts or goods (e.g. food, clothes etc.) received |
| --- |

Ba040. [Not live together_Offer of regular financial support] What was the monthly average amount of regular financial support such as pocket money, living expenses, medical expenses etc., that you give to [child’s name] in the last calendar year (2017)? (unit: 10,000 Korean won)

[IWER: This refers to the monthly average amount while you support regularly. For example, if you gave 1,200,000 Korean won in total to your child for 6 months, then, record ‘200,000 Korean won’.]

_______________________________ MW [Range: 1 ~ 9997] →Go to Ba046

Don’t know →Go to Ba041~Ba045 unfolding

Refuse to answer →Go to Ba041~Ba045 unfolding

**< Ba041~Ba045. Unfolding bracket questions >**

[IWER: Go back to Ba040 and record if R answers the amount during the interview.]

Ba041. Did it amount to less than, about equal to or more than 10 MW (10,000 Korean won)?

1. Less than 10 MW
2. About 10 MW
3. More than 10 MW

Ba042. Did it amount to less than, about equal to or more than 30 MW (10,000 Korean won)?

1. Less than 30 MW
2. About 30 MW

⑤ More than 30 MW

Ba043. Did it amount to less than, about equal to or more than 50 MW (10,000 Korean won)?

1. Less than 50 MW
2. About 50 MW

⑤ More than 50 MW

Ba044. Did it amount to less than, about equal to or more than 100 MW (10,000 Korean won)?

1. Less than 100 MW
2. About 100 MW

⑤ More than 100 MW

Ba045. Did it amount to less than, about equal to or more than 200 MW (10,000 Korean won)?

1. Less than 200 MW
2. About 200 MW

⑤ More than 200 MW

**Logic**

- **If Ba046 >= (12 – Ba014), do hard checking.**

Ba046. [Not live together_Offer of regular financial support] How many month(s) did you give any financial support to [child’s name] in the last calendar year (2017)?

[IWER: If you alternated between co-residing and not co-residing in 2017, please consider only the financial support you offered while you did not live together]

_______________________________ months [Range: 0~12]

Ba047. [Not live together_ Offer of occasional financial support] In the last calendar year (2017), while living separately did you and your spouse give to [child’s name] any occasional financial support such as pocket money, living expenses or medical expenses?

1. Yes →Go to Ba048 ⑤ No →Go to Ba054

| **[**IWER**]**  **<Regular support/ occasional support>**   - Regular support: monetary transfer regularly repeated in a certain time interval (e.g. each month, every two months) - Occasional support: monetary transfer irregularly occurred except birthday allowance or holiday allowances (e.g. paying for medical bills, schooling, occasional allowances)   **<Financial support/ Non-financial support>**   - Financial support: receiving cash or helping pay bills, or covering specific costs for medical care, insurance, schooling, down payment for a home or rent, etc. - Non-financial support: gifts or goods (e.g. food, clothes etc.) received |
| --- |

Ba048. [Not live together_ Offer of occasional financial support] What was the monthly average amount of occasional financial support such as pocket money, living expenses, medical expenses etc., that you gave to [child’s name] in the last calendar year (2017)?

[IWER: Please enter ‘100,000,000 Korean won (one hundred million won)’ first then, enter ’10,000 Korean won (MW)’.]

_______one hundred million won [Range: 1~9] _____MW [Range: 0 ~ 9997]

Don’t know →Go to Ba049~Ba053 unfolding

Refuse to answer →Go to Ba049~Ba053 unfolding

**< Ba049~Ba053. Unfolding bracket questions >**

[IWER: Go back to Ba048 and record if R answers the amount during the interview.]

Ba049. Did it amount to less than, about equal to or more than 100 MW (10,000 Korean won)?

1. Less than 100 MW
2. About 100 MW

⑤ More than 100 MW

Ba050. Did it amount to less than, about equal to or more than 300 MW (10,000 Korean won)?

1. Less than 300 MW
2. About 300 MW

⑤ More than 300 MW

Ba051. Did it amount to less than, about equal to or more than 500 MW (10,000 Korean won)?

1. Less than 500 MW
2. About 500 MW
3. More than 500 MW

Ba052. Did it amount to less than, about equal to or more than 1,000 MW (10,000 Korean won)?

1. Less than 1,000 MW
2. About 1,000 MW
3. More than 1,000 MW

Ba053. Did it amount to less than, about equal to or more than 2,000 MW (10,000 Korean won)?

1. Less than 2,000 MW
2. About 2,000 MW
3. More than 2,000 MW

Ba054. [Not live together_Offer of non-financial support] In the last calendar year (2017), while living separately did you and your spouse give to [child’s name] any gifts or goods (not cash)?

1. Yes →Go to Ba055 ⑤ No →Go to Ba057

Ba055. [Not live together_Offer of non-financial support] Then, what was the type of non‐financial support you gave to [child’s name] in the last calendar year (2017)? (Select multiple responses.)

1. Leisure (e.g. travel) →Go to Ba057
2. Health-related products (e.g. vitamins, equipment, etc.) →Go to Ba057
3. Household items →Go to Ba057
4. Electronics →Go to Ba057
5. Dining out and food →Go to Ba057
6. Clothes and shoes →Go to Ba057
7. Others →Go to Ba056

Ba056. If others, please specify.

_______________________________

**< Children who live together: Transfer >**

**Logic**

- **If R lived with his/her child for more than one month in 2017, then ask Ba057~B70.**

Ba057. [Live together_Receipt of financial support] In the last calendar year (2017), did you and your spouse receive any financial support from [child’s name] while living together?

[IWER: Provision of dwelling space, food, etc., is excluded. Financial support includes pocket money, medical expenses, travel expenses, business funds, etc. Paying for the bill instead is included]

1. Yes ⑤ No →Go to Ba064

Ba058. [Live together_Receipt of financial support] If so, what was the total amount of financial support you received from [child’s name] in the last calendar year (2017)?

[IWER: Please enter ‘100,000,000 Korean won (one hundred million won)’ first then, enter ’10,000 Korean won (MW)’.]

______one hundred million won [Range: 1~9] _______ MW [Range: 0 ~ 9997] → Ba064

Don’t know →Go to Ba059~Ba063 unfolding

Refuse to answer →Go to Ba059~Ba063 unfolding

**< Ba059~Ba063. Unfolding bracket questions >**

[IWER: Go back to Ba058 and record if R answers the amount during the interview.]

Ba059. Did it amount to less than, about equal to or more than 100 MW (10,000 Korean won)?

1. Less than 100 MW
2. About 100 MW

⑤ More than 100 MW

Ba060. Did it amount to less than, about equal to or more than 300 MW (10,000 Korean won)?

1. Less than 300 MW
2. About 300 MW

⑤ More than 300 MW

Ba061. Did it amount to less than, about equal to or more than 500 MW (10,000 Korean won)?

1. Less than 500 MW
2. About 500 MW

⑤ More than 500 MW

Ba062. Did it amount to less than, about equal to or more than 1,000 MW (10,000 Korean won)?

1. Less than 1,000 MW
2. About 1,000 MW

⑤ More than 1,000 MW

Ba063. Did it amount to less than, about equal to or more than 2,000 MW (10,000 Korean won)?

1. Less than 2,000 MW
2. About 2,000 MW

⑤ More than 2,000 MW

Ba064. [Live together_Offer of financial support] In the last calendar year (2017), did you and your spouse give any financial support to [child’s name] while living together?

[IWER: Provision of dwelling space, food, etc., is excluded. Financial support includes pocket money, medical expenses, travel expenses, business funds, etc. Paying for the bill instead is included.]

1. Yes ⑤ No →Go to Ba071

Ba065. [Live together_Offer of financial support] If so, what was the total amount of financial support given in the last calendar year (2017)?

[IWER: Please enter ‘100,000,000 Korean won (one hundred million won)’ first then, enter ’10,000 Korean won (MW)’.]

______one hundred million won [Range: 1~9] _________MW [Range: 0 ~ 9997] → Ba071

Don’t know →Go to Ba066~Ba070 unfolding

Refuse to answer →Go to Ba066~Ba070 unfolding

**< Ba066~Ba070. Unfolding bracket questions >**

[IWER: Go back to Ba065 and record if R answers the amount during the interview.]

Ba066. Did it amount to less than, about equal to or more than 100 MW (10,000 Korean won)?

1. Less than 100 MW
2. About 100 MW

⑤ More than 100 MW

Ba067. Did it amount to less than, about equal to or more than 300 MW (10,000 Korean won)?

1. Less than 300 MW
2. About 300 MW

⑤ More than 300 MW

Ba068. Did it amount to less than, about equal to or more than 500 MW (10,000 Korean won)?

1. Less than 500 MW
2. About 500 MW

⑤ More than 500 MW

Ba069. Did it amount to less than, about equal to or more than 1,000 MW (10,000 Korean won)?

1. Less than 1,000 MW
2. About 1,000 MW

⑤ More than 1,000 MW

Ba070. Did it amount to less than, about equal to or more than 2,000 MW (10,000 Korean won)?

1. Less than 2,000 MW
2. About 2,000 MW

⑤ More than 2,000 MW

**< Use of money that R gives R’s children>**

**Logic**

- **Ask Ba071 for both children who co-resided and those who did not.**
- **If Ba040=0, Ba048=0, or Ba065=0, go to Ba072**
- **If ‘other expenses’ at Ba071 > 0, ask Ba071_1**

Ba071. You have said that you and your spouse gave [child’s name] approximately [transfer amount] MW (10,000 Korean won) in the last calendar year (2017). Among these, how much accounts for educational expenses, wedding expenses, business expenses, medical expenses, and other expenses?

[IWER: The sum of all items must be equal to [(Ba040× Ba046)+Ba048+Ba065] MW.]

| ITEMS | AMOUNT(unit: 10,000 Korean won) |
| --- | --- |
| Educational expenses |  |
| Wedding expenses |  |
| Business expenses |  |
| Medical expenses |  |
| Other expenses |  |
| Total | [(Ba040× Ba046)+Ba048+Ba065] MW |

Ba071_1. What are those other expenses for? Please tell one thing with the highest weight.

_______________________________

**< Grandchild(ren): Total number of grandchildren (including dead/missing grandchildren), Ask about care labor >**

**Logic**

- **Ba072 is a loop value of Ba017. If Ba072<=R’s total number of grandchildren reported in Ba017, check for errors.**
- **Each member of the couple is asked Ba072~Ba084.**

Ba072. Now I’d like to ask you a few questions about grandchildren. Altogether, how many living grandchildren do you have? Please include those whose parent is dead or missing. (unit: number of grandchildren)

_______________________________ [Range: 0~50]

If 0 (Zero) →Go to Ba085

**Logic**

- **If Ba072 $\boldsymbol{\geq}$ 1, then go to Ba073.**

Ba073. Did you take care of any of those grandchildren before he/she reached the age of 10?

[IWER: Please enter ‘① Yes’ if R took care of a grandchild under the age of 10, even if she/ he is now aged 10 or over.]

1. Yes ⑤ No →Go to Ba085

Ba074. If so, how many grandchildren did you take care of? [unit: number of grandchildren]

[IWER: It is possible to enter a maximum of ten grandchildren R took care of. If the number exceeds 10, R is asked to choose ten whom R took care of the most.]

_______________________________ [Range: 1~10]

**Logic**

- **Loop Ba075~Ba078 as many times as the number of grandchildren in Ba074.**

Ba075. What is the name of the grandchild you took care of? (Please start with the eldest, in birth order) [IWER: If R does not provide the grandchild’s name, describe him/her as ‘the first grandchild of the first daughter’, ‘the first grandchild of the second son’, etc.]

_______________________________

Ba076. Whose child is [grandchild's name in Ba075]?

[IWER: In case of grandchildren whose parent is dead or missing, enter ‘⑪ Others’.]

①~⑩ Children list (of currently alive children)

[21] Others (missing or dead children)

Ba077. How long did you take care of [grandchild's name in Ba075]? (unit: a year)

[IWER: If the period of taking care of a grandchild is less than 1 year, enter 0]

_______________________________ year [Range: 0~9]

Ba078. On average how many hours per week did you care for [grandchild's name in Ba075] during that period? (unit: an average hour per week)

[IWER: If R took care of two or more grandchildren at the same time, R is advised to answer separately for each grandchild and not to double-count care hours. For example, if R cared for two grandchildren for 10 hours in total, enter 5 hours for each.]

_______________________________ average hour per week [Range: 1~168]

**Logic**

- **If Ba073=①, ask Ba079.**

Ba079. Did you take care of any of your grandchildren under the age of 10 during the past one year? [IWER: Past one year means a period of one year prior to now, not 2017.]

1. Yes ⑤ No →Go to Ba085

Ba080. How many grandchildren did you take care of during the past one year?

[IWER: The maximum number is to be 5. If the number exceeds 5, R is asked to choose the five he/she helped most to take care of.]

_______________________________ number [Range: 1~5]

**Logic**

- **Loop Ba081~Ba084 as many times as the number of grandchildren in Ba080.**

Ba081. What is the name of the grandchild you took care of? (Please start with the eldest, in birth order) [IWER: If R does not provide the grandchild’s name, describe him/her as ‘the first grandchild of the first daughter’, ‘the first grandchild of the second son’, etc.]

_______________________________

Ba082. Whose child is [grandchild's name in Ba081]?

[IWER: In case of grandchildren whose parent is dead or missing, enter ‘⑪ Others’.]

①~⑩ Children list (of currently alive children)

[21] Others (missing or dead children)

Ba083. How long did you take care of [grandchild's name in Ba077] during the past one year?

[IWER: Calculate 1 month as 4 weeks, 6 months as 26 weeks, 1 year as 52 weeks. Past one year means a period of one year prior to now, not 2017.]

_______________________________ weeks [Range: 1~52]

Ba084. On average how many hours per week did you spend on caring for [grandchild's name in Ba081] during the past one year? (unit: average number of hours per week)

[IWER: If R took care of two or more grandchildren at the same time, R is advised to answer separately for each grandchild and not to double-count care hours. For example, if R cared for two grandchildren for 10 hours in total, enter 5 hours for each.]

_______________________________ hour [Range: 1~168]

Ba085. [IWER: How often did R receive assistance in answering section Ba-FAMILY AND FAMILY TRANSFER (CHILDREN & GRANDCHILDREN)?]

1. Never → Go to section Bb.
2. A few times → Go to section Bb.
3. Most of the time → Go to section Bb.
4. The section was done by a proxy respondent

Ba086. If done by a proxy respondent, what is the proxy’s relationship to R? Please answer in view of the proxy.

1. Spouse
2. Mother
3. Father
4. Mother-in-law
5. Father-in-law
6. Brother/sister
7. Brother-in-law/sister-in-law
8. Son/daughter
9. Son-in-law/Daughter-in-law
10. Grandchild
11. Other relatives
12. Helper or other non-relatives

→Go to section Bb.

(Ba_end) [IWER] Please enter code 1 for completion of Ba. family (children and grand children) section.

1. Completion

# **Bb. FAMILY AND FAMILY TRANSFER**

**(PARENTS & SIBLINGS)**

(Bb_Intro) [IWER] Now, this is to start Bb. FAMILY (PARENTS & SIBLINGS) section.

1. Check

**< Information about R’s siblings (survival and living with R) >**

**Logic**

- **Loop Bb001~003 as many times as the number of siblings in the previous interview.**
- **If R did not have any siblings in the previous interview, then go to Bb004.**

Bb001. Now I am going to ask you about your sibling, [sibling's name]. Is [sibling's name] currently alive? [IWER: This question is intended to find out whether R’s sibling has died or not.]

1. Yes →Go to Bb003 ⑤ No (passed away)

Bb002. When did [sibling's name] pass away?

[IWER: Enter the year and month using 6 digits. For example, mark 201801 for January 2018. If the month is not clear, enter 201800.]

_______________________________ [Range: 190000~201811] →Go to Bb004

Bb003. Does [sibling's name] live with you?

1. Yes ⑤ No

**<Whether R’s parents died or not and basic information>**

**Logic**

- **If both R’s father and mother were alive in the previous interview, then ask Bb004~Bb015**
- **If only R’s father was alive in the previous interview, then ask Bb004~Bb009**
- **If only R’s mother was alive in the previous interview, then ask Bb010~Bb015**
- **If both R’s father and mother were passed away, then, ask Bb185**

Bb004. Now I have some questions about your father. Is [father’s name] currently alive?

[IWER: This question is intended to find out whether R’s father has died or not.]

1. Yes →Go to Ba006 ⑤ No (passed away)

Bb005. When did he die?

[IWER: Enter the year and month using 6 digits. For example, mark 201801 for January 2018. If the month is not clear, enter 201800.]

_____________________→Go to Bb010

Bb006. Does your father work for pay?

1. Yes ⑤ No

Bb007. Does your father own a house?

1. Yes ⑤ No

Bb008. Does your father live with you?

1. Yes ⑤ No

Bb009. At the time of the previous interview, you [were/weren’t co-residing] with your father. If you lived with your father in any month in the last calendar year (2017), how many months did yo live with your father last year? (unit: month)

[IWER: If R didn’t live together with father last year, mark 0]

_______________________________ months [Range:0~12]

Bb010. Now I have some questions about your mother. Is [mother’s name] currently alive?

[IWER: This question is intended to find out whether R’s mother has died or not.]

1. Yes →Go to Ba012 ⑤ No (passed away)

Bb011. When did she die?

[IWER: Enter the year and month using 6 digits. For example, mark 201801 for January 2018. If the month is not clear, enter 201800.]

_____________________→Go to Bb073

Bb012. Does your mother work for pay?

1. Yes ⑤ No

Bb013. Does your mother own a house?

1. Yes ⑤ No

Bb014. Does your mother live with you?

1. Yes ⑤ No

Bb015. At the time of the previous interview, you [were co-residing/weren’t co-residing] with your mother. If you lived with your mother in any month in the last calendar year (2017), how many months did you live with your father last year?

[IWER: If R didn’t live together with mother last year, mark 0]

_______________________________ months [Range:0~12]

**< Contact with R’s parents >**

**Logic**

- **If Bb004=①, Bb010=①, Bb008=⑤ and Bb014=⑤, then ask Bb016~Bb022**
- **If Bb004=⑤ and Bb010=⑤, then ask Bb185**
- **If (Bb004=①,Bb008=⑤ and Bb010=⑤) or Bb016=⑤, then ask Bb073~Bb078**
- **If (Bb004=⑤, Bb010=① and Bb014=⑤) or Bb016=⑤, then ask Bb129~Bb134**

Bb016. Do your parents live together?

[IWER: If R’s parents are separated or either of them is hospitalized for illness, enter ‘⑤ No’.]

1. Yes, they live together ⑤ No, they live apart →Go to Bb073

Bb017. Do they live by themselves, or with other children?

① By themselves →Go to Bb020

③ With other children

⑤ Others →Go to Bb019

Bb018. With whom do they live? If they live with multiple children, please identify all co‐residents.

[IWER: if children take in turn in living with parents, possible for multiple answers]

[27∼46] Sibling list →Go to Bb020

Bb019. If others, please specify.

_______________________________

Bb020. How close do you live to your parents?

1. Within a 30-minute distance by public transportation
2. Within a 1-hour distance by public transportation
3. Within a 2-hour distance by public transportation
4. More than a 2-hour distance by public transportation

Bb021. How often do you meet your parents in person?

1. Almost every day (more than 4 times per week)
2. Once a week
3. 2~3 times a week
4. Once a month
5. Twice a month (every two weeks)
6. Once or twice a year
7. Three or four times a year (once every three or four months)
8. Five or six times a year (every two months)
9. Almost never a year
10. Never met

Bb022. How often do you contact your parents by phone, mail, or e-mail?

1. Almost every day (more than 4 times per week)
2. Once a week
3. 2~3 times a week
4. Once a month
5. Twice a month (every two weeks)
6. Once or twice a year
7. Three or four times a year (once every three or four months)
8. Five or six times a year (every two months)
9. Almost never a year
10. Never contacted

**< Transfer to R’s parents who did not live with R >**

**Logic**

- **If R did not live with his/her parents for more than one month in 2017, then ask Bb023~Bb058.**

Bb023. [Not live together_Receipt of regular financial support] In the last calendar year (2017), while living separately did you receive from your parents any regular financial support such as pocket money, living expenses or medical expenses?

1. Yes →Go to Bb024 ⑤ No →Go to Bb031

| **[**IWER**]**  **<Regular support/ occasional support>**   - Regular support: monetary transfer regularly repeated in a certain time interval (e.g. each month, every two months) - Occasional support: monetary transfer irregularly occurred except birthday allowance or holiday allowances (e.g. paying for medical bills, schooling, occasional allowances)   **<Financial support/ Non-financial support>**   - Financial support: receiving cash or helping pay bills, or covering specific costs for medical care, insurance, schooling, down payment for a home or rent, etc. - Non-financial support: gifts or goods (e.g. food, clothes etc.) received |
| --- |

Bb024. [Not live together_Receipt of regular financial support] What was the monthly average amount of regular financial support you received from your parents in the last calendar year (2017)? (unit: 10,000 Korean won)

_______________________________ MW [Range: 1 ~ 9997] →Go to Bb030

Don’t know →Go to Bb025~Bb029 unfolding

Refuse to answer →Go to Bb025~Bb029 unfolding

**< Bb025~Bb029. Unfolding bracket questions >**

[IWER: Go back to Bb024 and record if R answers the amount during the interview.]

Bb025. Did it amount to less than, about equal to or more than 10 MW (10,000 Korean won) in the last calendar year (2017)?

1. Less than 10 MW
2. About 10 MW

⑤ More than 10 MW

Bb026. Did it amount to less than, about equal to or more than 30 MW (10,000 Korean won) in the last calendar year (2017)?

1. Less than 30 MW
2. About 30 MW

⑤ More than 30 MW

Bb027. Did it amount to less than, about equal to or more than 50 MW (10,000 Korean won) in the last calendar year (2017)?

1. Less than 50 MW
2. About 50 MW

⑤ More than 50 MW

Bb028. Did it amount to less than, about equal to or more than 100 MW (10,000 Korean won) in the last calendar year (2017)?

1. Less than 100 MW
2. About 100 MW

⑤ More than 100 MW

Bb029. Did it amount to less than, about equal to or more than 200 MW (10,000 Korean won) in the last calendar year (2017)?

1. Less than 200 MW
2. About 200 MW

⑤ More than 200 MW

Bb030. [Not live together_Receipt of regular financial support] How many month(s) did you receive regular financial support from your parents in the last calendar year (2017)? (unit: month)

[IWER: If you alternated between co-residing and not co-residing in 2017, please consider only the financial support you received while you did not live together]

_______________________________ months [Range:0~12]

Bb031. [Not live together_ Receipt of occasional financial support] In the last calendar year (2017), while living separately did you receive from your parents any occasional financial support such as pocket money, living expenses or medical expenses?

1. Yes →Go to Bb032 ⑤ No →Go to Bb038

Bb032. [Not live together_ Receipt of occasional financial support] What was the total amount of occasional financial support you received from your parents in the last calendar year (2017)?

[IWER: Please enter ‘100,000,000 Korean won (one hundred million won)’ first then, enter ’10,000 Korean won (MW)’.]

______one hundred million won [Range: 1~9] ________ MW [Range: 0 ~ 9997] → Bb038

Don’t know →Go to Bb033~Bb037 unfolding

Refuse to answer →Go to Bb033~Bb037 unfolding

**< Bb033~Bb037. Unfolding bracket questions >**

[IWER: Go back to Bb032 and record if R answers the amount during the interview.]

Bb033. Did it amount to less than, about equal to or more than 100 MW (10,000 Korean won) in the last calendar year (2017)?

1. Less than 100 MW
2. About 100 MW

⑤ More than 100 MW

Bb034. Did it amount to less than, about equal to or more than 300 MW (10,000 Korean won) in the last calendar year (2017)?

1. Less than 300 MW
2. About 300 MW

⑤ More than 300 MW

Bb035. Did it amount to less than, about equal to or more than 500 MW (10,000 Korean won) in the last calendar year (2017)?

1. Less than 500 MW
2. About 500 MW

⑤ More than 500 MW

Bb036. Did it amount to less than, about equal to or more than 1,000 MW (10,000 Korean won) in the last calendar year (2017)?

1. Less than 1,000 MW
2. About 1,000 MW

⑤ More than 1,000 MW

Bb037. Did it amount to less than, about equal to or more than 2,000 MW (10,000 Korean won) in the last calendar year (2017)?

1. Less than 2,000 MW
2. About 2,000 MW

⑤ More than 2,000 MW

Bb038. [Not live together_ Receipt of non-financial support] In the last calendar year (2017), while living separately did you receive from your parents any gifts or goods (not cash)?

1. Yes →Go to Bb039 ⑤ No →Go to Bb041

Bb039. [Not live together_Receipt of non-financial support] Then, what was the type of non‐financial support you received from your parents in the last calendar year (2017)? Choose all that apply. (Select multiple responses.)

1. Leisure (e.g. travel) →Go to Bb041
2. Health‐related products (e.g. vitamins, equipment, etc.) →Go to Bb041
3. Household items →Go to Bb041
4. Electronics →Go to Bb041
5. Dining out and food →Go to Bb041
6. Clothes and shoes →Go to Bb041
7. Others →Go to Bb040

Bb040. If others, please specify.

_______________________________

Bb041. [Not live together_ Offer of regular financial support] In the last calendar year (2017), while living separately did you give to your parents any financial support such as pocket money, living expenses or medical expenses?

1. Yes →Go to Bb042 ⑤ No →Go to Bb049

| **[**IWER**]**  **<Regular support/ occasional support>**   - Regular support: monetary transfer regularly repeated in a certain time interval (e.g. each month, every two months) - Occasional support: monetary transfer irregularly occurred except birthday allowance or holiday allowances (e.g. paying for medical bills, schooling, occasional allowances)   **<Financial support/ Non-financial support>**   - Financial support: receiving cash or helping pay bills, or covering specific costs for medical care, insurance, schooling, down payment for a home or rent, etc. - Non-financial support: gifts or goods (e.g. food, clothes etc.) received |
| --- |

Bb042. [Not live together_Offer of regular financial support] What was the monthly average amount of regular financial support you gave to your parents in the last calendar year (2017)? (unit: 10,000 Korean won)

_______________________________ MW [Range: 1 ~ 9997] →Go to Bb048

Don’t know →Go to Bb043~Bb047 unfolding

Refuse to answer →Go to Bb043~Bb047 unfolding

**< Bb043~Bb047. Unfolding bracket questions >**

[IWER: Go back to Bb042 and record if R answers the amount during the interview.]

Bb043. Did it amount to less than, about equal to or more than 10 MW (10,000 Korean won) in the last calendar year (2017)?

1. Less than 10 MW
2. About 10 MW

⑤ More than 10 MW

Bb044. Did it amount to less than, about equal to or more than 30 MW (10,000 Korean won) in the last calendar year (2017)?

1. Less than 30 MW
2. About 30 MW

⑤ More than 30 MW

Bb045. Did it amount to less than, about equal to or more than 50 MW (10,000 Korean won) in the last calendar year (2017)?

1. Less than 50 MW
2. About 50 MW

⑤ More than 50 MW

Bb046. Did it amount to less than, about equal to or more than 100 MW (10,000 Korean won) in the last calendar year (2017)?

1. Less than 100 MW
2. About 100 MW

⑤ More than 100 MW

Bb047. Did it amount to less than, about equal to or more than 200 MW (10,000 Korean won) in the last calendar year (2017)?

1. Less than 200 MW
2. About 200 MW

⑤ More than 200 MW

Bb048. [Not live together_Offer of regular financial support] How many months did you give regular financial support to your parents in the last calendar year (2017)?

[IWER: If you alternated between co-residing and not co-residing in 2017, please consider only the financial support you gave while you did not live together]

_______________________________ months [Range:0~12]

Bb049. [Not live together_Offer of non-financial support] In the last calendar year (2017), while living separately did you give any occasional financial support such as pocket money, living expenses or medical expenses to your parents?

1. Yes →Go to Bb050 ⑤ No →Go to Bb056

| **[**IWER**]**  **<Regular support/ occasional support>**   - Regular support: monetary transfer regularly repeated in a certain time interval (e.g. each month, every two months) - Occasional support: monetary transfer irregularly occurred except birthday allowance or holiday allowances (e.g. paying for medical bills, schooling, occasional allowances)   **<Financial support/ Non-financial support>**   - Financial support: receiving cash or helping pay bills, or covering specific costs for medical care, insurance, schooling, down payment for a home or rent, etc. - Non-financial support: gifts or goods (e.g. food, clothes etc.) received |
| --- |

Bb050. [Not live together_ Offer of occasional financial support] What was the total amount of occasional financial support given to your parents in the last calendar year (2017)?

[IWER: Please enter ‘100,000,000 Korean won (one hundred million won)’ first then, enter ’10,000 Korean won (MW)’.]

______one hundred million won [Range: 1~9] _________MW [Range: 0 ~ 9997] → Bb056

Don’t know →Go to Bb051~Bb055 unfolding

Refuse to answer →Go to Bb051~Bb055 unfolding

**< Bb051~Bb055. Unfolding bracket questions >**

[IWER: Go back to Bb050 and record if R answers the amount during the interview.]

Bb051. Did it amount to less than, about equal to or more than 100 MW (10,000 Korean won) in the last calendar year (2017)?

1. Less than 100 MW
2. About 100 MW

⑤ More than 100 MW

Bb052. Did it amount to less than, about equal to or more than 300 MW (10,000 Korean won) in the last calendar year (2017)?

1. Less than 300 MW
2. About 300 MW

⑤ More than 300 MW

Bb053. Did it amount to less than, about equal to or more than 500 MW (10,000 Korean won) in the last calendar year (2017)?

1. Less than 500 MW
2. About 500 MW

⑤ More than 500 MW

Bb054. Did it amount to less than, about equal to or more than 1,000 MW (10,000 Korean won) in the last calendar year (2017)?

1. Less than 1,000 MW
2. About 1,000 MW

⑤ More than 1,000 MW

Bb055. Did it amount to less than, about equal to or more than 2,000 MW (10,000 Korean won) in the last calendar year (2017)?

1. Less than 2,000 MW
2. About 2,000 MW

⑤ More than 2,000 MW

Bb056. [Not live together_ Offer of non-financial support] In the last calendar year (2017), while living separately did you give to your parents any gifts or goods (not cash)?

1. Yes →Go to Bb057 ⑤ No →Go to Bb059

Bb057. [Not live together_ Offer of non-financial support] Then, what was the type of non‐financial support you gave to your parents in the last calendar year (2017)? (Select multiple responses)

1. Leisure (e.g. travel) →Go to Bb059
2. Health-related products (e.g. vitamins, equipment, etc) →Go to Bb059
3. Household items →Go to Bb059
4. Electronics →Go to Bb059
5. Dining out and food →Go to Bb059
6. Clothes and shoes →Go to Bb059
7. Others →Go to Bb058

Bb058. If others, please specify.

_______________________________

**< Transfer to R’s parents who lived with R >**

**Logic**

- **If R lived with his/her parents for more than one month in 2017, then ask Bb059~Bb072.**

Bb059. [Live together_Receipt of financial support] In the last calendar year (2017), did you receive any financial support from your parents while living together?

[IWER: Provision of dwelling space, food, etc., is excluded. Financial support includes pocket money, medical expenses, travel expenses, business funds, etc. Paying for the bill instead is included.]

1. Yes ⑤ No →Go to Bb066

Bb060. If so, what was the total amount of financial support you received from your parents in the last calendar year (2017)?

[IWER: Please enter ‘100,000,000 Korean won (one hundred million won)’ first then, enter ’10,000 Korean won (MW)’.]

______one hundred million won [Range: 1~9] _________MW [Range: 0 ~ 9997] → Bb066

Don’t know →Go to Bb061~Bb065 unfolding

Refuse to answer →Go to Bb061~Bb065 unfolding

**< Bb061~Bb065. Unfolding bracket questions >**

[IWER: Go back to Bb060 and record if R answers the amount during the interview.]

Bb061. Did it amount to less than, about equal to or more than 100 MW (10,000 Korean won)?

1. Less than 100 MW
2. About 100 MW

⑤ More than 100 MW

Bb062.Did it amount to less than, about equal to or more than 300 MW (10,000 Korean won)?

1. Less than 300 MW
2. About 300 MW

⑤ More than 300 MW

Bb063. Did it amount to less than, about equal to or more than 500 MW (10,000 Korean won)?

1. Less than 500 MW
2. About 500 MW

⑤ More than 500 MW

Bb064. Did it amount to less than, about equal to or more than 1,000 MW (10,000 Korean won)?

1. Less than 1,000 MW
2. About 1,000 MW

⑤ More than 1,000 MW

Bb065. Did it amount to less than, about equal to or more than 2,000 MW (10,000 Korean won)?

1. Less than 2,000 MW
2. About 2,000 MW

⑤ More than 2,000 MW

Bb066. [Live together_Offer of financial support] In the last calendar year (2017), did you give any financial support to your parents while living together?

[IWER: Provision of dwelling space, food, etc., is excluded. Financial support includes pocket money, medical expenses, travel expenses, business funds, etc. Paying for the bill instead is included]

1. Yes ⑤ No →Go to Bb185

Bb067. [Live together_Offer of financial support] If so, what was the total amount of financial support you gave to your parents in the last calendar year (2017)?

[IWER: Please enter ‘100,000,000 Korean won (one hundred million won)’ first then, enter ’10,000 Korean won (MW)’.]

______one hundred million won [Range: 1~9] _________MW [Range: 0 ~ 9997] → Bb185

Don’t know →Go to Bb068~Bb072 unfolding

Refuse to answer →Go to Bb068~Bb072 unfolding

**< Bb068~Bb072. Unfolding bracket questions >**

[IWER: Go back to Bb067 and record if R answers the amount during the interview.]

Bb068. Did it amount to less than, about equal to or more than 100 MW (10,000 Korean won)?

1. Less than 100 MW
2. About 100 MW

⑤ More than 100 MW

Bb069.Did it amount to less than, about equal to or more than 300 MW (10,000 Korean won)?

1. Less than 300 MW
2. About 300 MW

⑤ More than 300 MW

Bb070. Did it amount to less than, about equal to or more than 500 MW (10,000 Korean won)?

1. Less than 500 MW
2. About 500 MW

⑤ More than 500 MW

Bb071. Did it amount to less than, about equal to or more than 1,000 MW (10,000 Korean won)?

1. Less than 1,000 MW
2. About 1,000 MW

⑤ More than 1,000 MW

Bb072. Did it amount to less than, about equal to or more than 2,000 MW (10,000 Korean won)?

1. Less than 2,000 MW
2. About 2,000 MW

⑤ More than 2,000 MW

**< Contact with R’s father >**

**Logic**

- **If(Bb004=①, Bb008=⑤ and Bb010=⑤) or Bb016=⑤, then ask Bb073~Bb078.**

Bb073. Does your father live by himself or with other children?

① By himself →Go to Bb076

③ With other children

⑤ Others →Go to Bb075

Bb074. With whom does he live? Please list all, if he lives with children. [IWER: Choose all that apply.]

[27~46] Sibling list →Go to Bb076

Bb075. If others, please specify.

_______________________________

Bb076. How close do you live to your father?

1. Within a 30-minute distance by public transportation
2. Within a 1-hour distance by public transportation
3. Within a 2-hour distance by public transportation
4. More than a 2-hour distance by public transportation

Bb077. How often do you meet your father in person?

1. Almost every day (more than 4 times per week)
2. Once a week
3. 2~3 times a week
4. Once a month
5. Twice a month (every two weeks)
6. Once or twice a year
7. Three or four times a year (once every three or four months)
8. Five or six times a year (every two months)
9. Almost never a year
10. Never met

Bb078. How often do you contact your father by phone, mail, or e-mail?

1. Almost every day (more than 4 times per week)
2. Once a week
3. 2~3 times a week
4. Once a month
5. Twice a month (every two weeks)
6. Once or twice a year
7. Three or four times a year (once every three or four months)
8. Five or six times a year (every two months)
9. Almost never a year
10. Never contacted

**< Transfer to R’s father >**

**Logic**

- **If R did not live with his/her father for more than one month in 2017, then ask Bb079~Bb114.**

Bb079. [Not live together_ Receipt of regular financial support] In the last calendar year (2017), while living separately did you receive from your father any financial support such as pocket money, living expenses or medical expenses?

1. Yes →Go to Bb080 ⑤ No →Go to Bb087

| **[IWER]**  **<Regular support/ occasional support>**   - Regular support: monetary transfer regularly repeated in a certain time interval (e.g. each month, every two months) - Occasional support: monetary transfer irregularly occurred except birthday allowance or holiday allowances (e.g. paying for medical bills, schooling, occasional allowances)   **<Financial support/ Non-financial support>**   - Financial support: receiving cash or helping pay bills, or covering specific costs for medical care, insurance, schooling, down payment for a home or rent, etc. - Non-financial support: gifts or goods (e.g. food, clothes etc.) received |
| --- |

Bb080. [Not live together_Receipt of regular financial support] What was the monthly average amount of regular financial support you received from your father in the last calendar year (2017)? (unit: 10,000 Korean won)

_______________________________ MW [Range: 1 ~ 9997] →Go to Bb086

Don’t know →Go to Bb081~Bb085 unfolding

Refuse to answer →Go to Bb081~Bb085 unfolding

**< Bb081~Bb085. Unfolding bracket questions >**

[IWER: Go back to Bb080 and record if R answers the amount during the interview.]

Bb081. Did it amount to less than, about equal to or more than 10 MW (10,000 Korean won) in the last calendar year (2017)?

1. Less than 10 MW
2. About 10 MW

⑤ More than 10 MW

Bb082. Did it amount to less than, about equal to or more than 30 MW (10,000 Korean won) in the last calendar year (2017)?

1. Less than 30 MW
2. About 30 MW

⑤ More than 30 MW

Bb083. Did it amount to less than, about equal to or more than 50 MW (10,000 Korean won) in the last calendar year (2017)?

1. Less than 50 MW
2. About 50 MW

⑤ More than 50 MW

Bb084. Did it amount to less than, about equal to or more than 100 MW (10,000 Korean won) in the last calendar year (2017)?

1. Less than 100 MW
2. About 100 MW

⑤ More than 100 MW

Bb085. Did it amount to less than, about equal to or more than 200 MW (10,000 Korean won) in the last calendar year (2017)?

1. Less than 200 MW
2. About 200 MW

⑤ More than 200 MW

**Logic**

- **If Bb086 >= (12 – Bb009), do hard checking.**

Bb086. [Not live together_ Receipt of regular financial support] How many months did you receive regular financial support from your father in the last calendar year (2017)? (unit: month)

[IWER: If you alternated between co-residing and not co-residing in 2017, please consider only the financial support you received while you did not live together]

_______________________________ months [Range:0~12]

Bb087. [Not live together_ Receipt of occasional financial support] In the last calendar year (2017), while living separately did you receive any occasional financial support such as pocket money, living expenses or medical expenses from your father?

1. Yes →Go to Bb088 ⑤ No →Go to Bb094

Bb088. [Not live together_Receipt of occasional financial support] What was the total amount of occasional financial support you received from your father in the last calendar year (2017)?

[IWER: Please enter ‘100,000,000 Korean won (one hundred million won)’ first then, enter ’10,000 Korean won (MW)’.]

______one hundred million won [Range: 1~9] _________MW [Range: 0 ~ 9997] → Bb094

Don’t know →Go to Bb089~Bb093 unfolding

Refuse to answer →Go to Bb089~Bb093 unfolding

**< Bb089~Bb093. Unfolding bracket questions >**

[IWER: Go back to Bb088 and record if R answers the amount during the interview.]

Bb089. Did it amount to less than, about equal to or more than 100 MW (10,000 Korean won) in the last calendar year (2017)?

1. Less than 100 MW
2. About 100 MW

⑤ More than 100 MW

Bb090. Did it amount to less than, about equal to or more than 300 MW (10,000 Korean won) in the last calendar year (2017)?

1. Less than 300 MW
2. About 300 MW

⑤ More than 300 MW

Bb091. Did it amount to less than, about equal to or more than 500 MW (10,000 Korean won) in the last calendar year (2017)?

1. Less than 500 MW
2. About 500 MW

⑤ More than 500 MW

Bb092. Did it amount to less than, about equal to or more than 1,000 MW (10,000 Korean won) in the last calendar year (2017)?

1. Less than 1,000 MW
2. About 1,000 MW

⑤ More than 1,000 MW

Bb093. Did it amount to less than, about equal to or more than 2,000 MW (10,000 Korean won) in the last calendar year (2017)?

1. Less than 2,000 MW
2. About 2,000 MW

⑤ More than 2,000 MW

Bb094. [Not live together_Receipt of non-financial support] In the last calendar year (2017), while living separately did you receive any gifts or goods (not cash) from your father?

1. Yes →Go to Bb095 ⑤ No →Go to Bb097

Bb095. [Not live together_Receipt of non-financial support] Then, what was the type of non-financial support you received from your father in the last calendar year (2017)? (Select multiple responses)

1. Leisure (e.g. travel) →Go to Bb097
2. Health-related products (e.g. vitamins, equipment, etc) →Go to Bb097
3. Household items →Go to Bb097
4. Electronics →Go to Bb097
5. Dining out and food →Go to Bb097
6. Clothes and shoes →Go to Bb097
7. Others →Go to Bb096

Bb096. If others, please specify.

_______________________________

Bb097. [Not live together_Offer of regular financial support] In the last calendar year (2017), while living separately did you give to your father any financial support such as pocket money, living expenses or medical expenses?

1. Yes →Go to Bb098 ⑤ No →Go to Bb105

Bb098. [Not live together_Offer of regular financial support] What was the monthly average amount of regular financial support you gave to your father in the last calendar year (2017)? (unit: 10,000 Korean won)

_______________________________ MW [Range: 1 ~ 9997] →Go to Bb104

Don’t know →Go to Bb099~Bb103 unfolding

Refuse to answer →Go to Bb099~Bb103 unfolding

**< Bb099~Bb103. Unfolding bracket questions >**

[IWER: Go back to Bb098 and record if R answers the amount during the interview.]

Bb099. Did it amount to less than, about equal to or more than 10 MW (10,000 Korean won) in the last calendar year (2017)?

1. Less than 10 MW
2. About 10 MW

⑤ More than 10 MW

Bb100. Did it amount to less than, about equal to or more than 30 MW (10,000 Korean won) in the last calendar year (2017)?

1. Less than 30 MW
2. About 30 MW

⑤ More than 30 MW

Bb101. Did it amount to less than, about equal to or more than 50 MW (10,000 Korean won) in the last calendar year (2017)?

1. Less than 50 MW
2. About 50 MW

⑤ More than 50 MW

Bb102. Did it amount to less than, about equal to or more than 100 MW (10,000 Korean won) in the last calendar year (2017)?

1. Less than 100 MW
2. About 100 MW

⑤ More than 100 MW

Bb103. Did it amount to less than, about equal to or more than 200 MW (10,000 Korean won) in the last calendar year (2017)?

1. Less than 200 MW
2. About 200 MW

⑤ More than 200 MW

**Logic**

- **If Bb104 >= (12 – Bb009), do hard checking.**

Bb104. [Not live together_Offer of regular financial support] How many months did you give regular financial support to your father in the last calendar year (2017)?

[IWER: If you alternated between co-residing and not co-residing in 2017, please consider only the financial support you gave while you did not live together]

_______________________________ months [Range:0~12]

Bb105. [Not live together_Offer of occasional financial support] In the last calendar year (2017), while living separately did you give to your father any occasional financial support such as pocket money, living expenses or medical expenses?

1. Yes →Go to Bb106 ⑤ No →Go to Bb112

Bb106. [Not live together_ Offer of occasional financial support] What was the total amount of occasional financial support given to your father in the last calendar year (2017)?

[IWER: Please enter ‘100,000,000 Korean won (one hundred million won)’ first then, enter ’10,000 Korean won (MW)’.]

______one hundred million won [Range: 1~9] ________ MW [Range: 0 ~ 9997] → Bb112

Don’t know →Go to Bb107~Bb111 unfolding

Refuse to answer →Go to Bb107~Bb111 unfolding

**< Bb107~Bb111. Unfolding bracket questions >**

[IWER: Go back to Bb106 and record if R answers the amount during the interview.]

Bb107. Did it amount to less than, about equal to or more than 100 MW (10,000 Korean won) in the last calendar year (2017)?

1. Less than 100 MW
2. About 100 MW

⑤ More than 100 MW

Bb108. Did it amount to less than, about equal to or more than 300 MW (10,000 Korean won) in the last calendar year (2017)?

1. Less than 300 MW
2. About 300 MW

⑤ More than 300 MW

Bb109. Did it amount to less than, about equal to or more than 500 MW (10,000 Korean won) in the last calendar year (2017)?

1. Less than 500 MW
2. About 500 MW

⑤ More than 500 MW

Bb110. Did it amount to less than, about equal to or more than 1,000 MW (10,000 Korean won) in the last calendar year (2017)?

1. Less than 1,000 MW
2. About 1,000 MW

⑤ More than 1,000 MW

Bb111. Did it amount to less than, about equal to or more than 2,000 MW (10,000 Korean won) in the last calendar year (2017)?

1. Less than 2,000 MW
2. About 2,000 MW

⑤ More than 2,000 MW

Bb112. [Not live together_ Offer of non-financial support] In the last calendar year (2017), while living separately did you give any gifts or goods (not cash) to your father?

1. Yes →Go to Bb113 ⑤ No →Go to Bb115

Bb113. [Not live together_ Offer of non-financial support] Then, what was the type of non-financial support you gave to your father in the last calendar year (2017)? Choose all that apply. (Select multiple responses)

1. Leisure (e.g. travel) →Go to Bb115 or Bb129
2. Health-related products (e.g. vitamins, equipment, etc) → Go to Bb115 or Bb129
3. Household items → Go to Bb115 or Bb129
4. Electronics → Go to Bb115 or Bb129
5. Dining out and food → Go to Bb115 or Bb129
6. Clothes and shoes → Go to Bb115 or Bb129
7. Others

Bb114. If others, please specify.

_______________________________ →Go to Bb115 or Bb129

**<Transfer to R’s father who lived with R>**

**Logic**

- **If R lived with his/her father for more than one month in 2017, then ask Bb115~Bb128.**

Bb115. [Live together_Receipt of financial support] In the last calendar year (2017), did you receive any financial support from your father while living together?

[IWER: Provision of dwelling space, food, etc., is excluded. Financial support includes pocket money, medical expenses, travel expenses, business funds, etc. Paying for the bill instead is included.]

1. Yes ⑤ No →Go to Bb122

Bb116. [Live together_Receipt of financial support] If so, what was the total amount of financial support you received from your father in the last calendar year (2017)?

[IWER: Please enter ‘100,000,000 Korean won (one hundred million won)’ first then, enter ’10,000 Korean won (MW)’.]

______one hundred million won [Range: 1~9] ________ MW [Range: 0 ~ 9997] → Bb122

Don’t know →GO to Bb117~Bb121 unfolding

Refuse to answer →GO to Bb117~Bb121 unfolding

**< Bb117~Bb121. Unfolding bracket questions >**

[IWER: Go back to Bb116 and record if R answers the amount during the interview.]

Bb117. Did it amount to less than, about equal to or more than 100 MW (10,000 Korean won) in the last calendar year (2017)?

1. Less than 100 MW
2. About 100 MW

⑤ More than 100 MW

Bb118. Did it amount to less than, about equal to or more than 300 MW (10,000 Korean won) in the last calendar year (2017)?

1. Less than 300 MW
2. About 300 MW

⑤ More than 300 MW

Bb119. Did it amount to less than, about equal to or more than 500 MW (10,000 Korean won) in the last calendar year (2017)?

1. Less than 500 MW
2. About 500 MW

⑤ More than 500 MW

Bb120. Did it amount to less than, about equal to or more than 1,000 MW (10,000 Korean won) in the last calendar year (2017)?

1. Less than 1,000 MW
2. About 1,000 MW

⑤ More than 1,000 MW

Bb121. Did it amount to less than, about equal to or more than 2,000 MW (10,000 Korean won) in the last calendar year (2017)?

1. Less than 2,000 MW
2. About 2,000 MW

⑤ More than 2,000 MW

Bb122. [Live together_Offer of financial support] In the last calendar year (2017), did you give any financial support to your father while living together?

[IWER: Provision of dwelling space, food, etc., is excluded. Financial support includes pocket money, medical expenses, travel expenses, business funds, etc. Paying for the bill instead is included.]

1. Yes ⑤ No →Go to Bb185

Bb123. [Live together_offer of financial support] If so, what was the total amount of financial support you gave to your father in the last calendar year (2017)?

[IWER: Please enter ‘100,000,000 Korean won (one hundred million won)’ first then, enter ’10,000 Korean won (MW)’.]

______one hundred million won [Range: 1~9] _________MW [Range: 0 ~ 9997] → Bb185

Don’t know →Go to Bb124~Bb128 unfolding

Refuse to answer →Go to Bb124~Bb128 unfolding

**< Bb124~Bb128. Unfolding bracket questions >**

[IWER: Go back to Bb123 and record if R answers the amount during the interview.]

Bb124. Did it amount to less than, about equal to or more than 100 MW (10,000 Korean won)?

1. Less than 100 MW
2. About 100 MW

⑤ More than 100 MW

Bb125. Did it amount to less than, about equal to or more than 300 MW (10,000 Korean won)?

1. Less than 300 MW
2. About 300 MW

⑤ More than 300 MW

Bb126. Did it amount to less than, about equal to or more than 500 MW (10,000 Korean won)?

1. Less than 500 MW
2. About 500 MW

⑤ More than 500 MW

Bb127. Did it amount to less than, about equal to or more than 1,000 MW (10,000 Korean won)?

1. Less than 1,000 MW
2. About 1,000 MW

⑤ More than 1,000 MW

Bb128. Did it amount to less than, about equal to or more than 2,000 MW (10,000 Korean won)?

1. Less than 2,000 MW
2. About 2,000 MW

⑤ More than 2,000 MW

**< Contact with R’s mother >**

**Logic**

- **If (Bb004=⑤, Bb010=①, and Bb014=⑤) or Bb016=⑤, then ask Bb129~Bb134.**

Bb129. Does your mother live by herself or with other children?

① By herself →Go to Bb132

③ With other children

⑤ Others →Go to Bb131

Bb130. With whom does she live? Please list all, if she lives with children. [IWER: Choose all that apply]

[27∼46] Sibling list →Go to Bb132

Bb131. If others, please specify.

_______________________________

Bb132. How close do you live to your mother?

1. Within a 30-minute distance by public transportation
2. Within a 1-hour distance by public transportation
3. Within a 2-hour distance by public transportation
4. More than a 2-hour distance by public transportation

Bb133. How often do you meet your mother in person?

1. Almost every day (more than 4 times per week)
2. Once a week
3. 2~3 times a week
4. Once a month
5. Twice a month (every two weeks)
6. Once or twice a year
7. Three or four times a year (once every three or four months)
8. Five or six times a year (every two months)
9. Almost never a year
10. Never met

Bb134. How often do you contact your mother by phone, mail, or e-mail?

1. Almost every day (more than 4 times per week)
2. Once a week
3. 2~3 times a week
4. Once a month
5. Twice a month (every two weeks)
6. Once or twice a year
7. Three or four times a year (once every three or four months)
8. Five or six times a year (every two months)
9. Almost never a year
10. Never contacted

**< Transfer to R’s mother >**

**Logic**

- **If R did not live with his/her mother for more than one month in 2017, then ask Bb135~Bb170.**

Bb135. [Not live together_Receipt of financial support] In the last calendar year (2017), while living separately did you receive from your mother any financial support such as pocket money, living expenses or medical expenses?

1. Yes →Go to Bb136 ⑤ No →Go to Bb143

| **[**IWER**]**  **<Regular support/ occasional support>**   - Regular support: monetary transfer regularly repeated in a certain time interval (e.g. each month, every two months) - Occasional support: monetary transfer irregularly occurred except birthday allowance or holiday allowances (e.g. paying for medical bills, schooling, occasional allowances)   **<Financial support/ Non-financial support>**   - Financial support: receiving cash or helping pay bills, or covering specific costs for medical care, insurance, schooling, down payment for a home or rent, etc. - Non-financial support: gifts or goods (e.g. food, clothes etc.) received |
| --- |

Bb136. [Not live together_Receipt of financial support] What was the monthly average amount of regular financial support you received from your mother in the last calendar year (2017)? (unit: 10,000 Korean won)

_______________________________ MW [Range: 1 ~ 9997] →Go to Bb142

Don’t know →Bb137~Bb141 unfolding

Refuse to answer →Bb137~Bb141 unfolding

**< Bb137~Bb141. Unfolding bracket questions >**

[IWER: Go back to Bb136 and record if R answers the amount during the interview.]

Bb137. Did it amount to less than, about equal to or more than 10 MW (10,000 Korean won) in the last calendar year (2017)?

1. Less than 10 MW
2. About 10 MW

⑤ More than 10 MW

Bb138. Did it amount to less than, about equal to or more than 30 MW (10,000 Korean won) in the last calendar year (2017)?

1. Less than 30 MW
2. About 30 MW

⑤ More than 30 MW

Bb139. Did it amount to less than, about equal to or more than 50 MW (10,000 Korean won) in the last calendar year (2017)?

1. Less than 50 MW
2. About 50 MW

⑤ More than 50 MW

Bb140. Did it amount to less than, about equal to or more than 100 MW (10,000 Korean won) in the last calendar year (2017)?

1. Less than 100 MW
2. About 100 MW

⑤ More than 100 MW

Bb141. Did it amount to less than, about equal to or more than 200 MW (10,000 Korean won) in the last calendar year (2017)?

1. Less than 200 MW
2. About 200 MW

⑤ More than 200 MW

**Logic**

- **If Bb142 >= (12 - Bb015), do hard checking.**

Bb142. [Not live together_Receipt of regular financial support] How many month(s) did you receive regular financial support from your mother in the last calendar year (2017)?

[IWER: If you alternated between co-residing and not co-residing in 2017, please consider only the financial support you received while you did not live together]

_______________________________ months [Range:0~12]

Bb143. [Not live together_ Receipt of occasional financial support] In the last calendar year (2017), while living separately did you receive from your mother any occasional financial support such as pocket money, living expenses or medical expenses?

1. Yes →Go to Bb144 ⑤ No →Go to Bb150

Bb144. What was the total amount of occasional financial support you received from your mother in the last calendar year (2017)?

[IWER: Please enter ‘100,000,000 Korean won (one hundred million won)’ first then, enter ’10,000 Korean won (MW)’.]

______one hundred million won [Range: 1~9] ________ MW [Range: 0 ~ 9997] → Bb150

Don’t know →Bb145~Bb149 unfolding

Refuse to answer →Bb145~Bb149 unfolding

**< Bb145~Bb149. Unfolding bracket questions >**

[IWER: Go back to Bb144 and record if R answers the amount during the interview.]

Bb145. Did it amount to less than, about equal to or more than 100 MW (10,000 Korean won) in the last calendar year (2017)?

1. Less than 100 MW
2. About 100 MW

⑤ More than 100 MW

Bb146. Did it amount to less than, about equal to or more than 300 MW (10,000 Korean won) in the last calendar year (2017)?

1. Less than 300 MW
2. About 300 MW

⑤ More than 300 MW

Bb147. Did it amount to less than, about equal to or more than 500 MW (10,000 Korean won) in the last calendar year (2017)?

1. Less than 500 MW
2. About 500 MW

⑤ More than 500 MW

Bb148. Did it amount to less than, about equal to or more than 1,000 MW (10,000 Korean won) in the last calendar year (2017)?

1. Less than 1,000 MW
2. About 1,000 MW

⑤ More than 1,000 MW

Bb149. Did it amount to less than, about equal to or more than 2,000 MW (10,000 Korean won) in the last calendar year (2017)?

1. Less than 2,000 MW
2. About 2,000 MW

⑤ More than 2,000 MW

Bb150. [Not live together_ Receipt of non-financial support] In the last calendar year (2017), while living separately did you receive any gifts or goods (not cash) from your mother?

1. Yes →Go to Bb151 ⑤ No →Go to Bb153

Bb151. [Not live together_ Receipt of non-financial support] What was the type of non‐financial support you received from your mother in the last calendar year (2017)? Choose all that apply. (Select multiple responses)

1. Leisure (e.g. travel) →Go to Bb153
2. Health‐related products (e.g. vitamins, equipment, etc) →Go to Bb153
3. Household items →Go to Bb153
4. Electronics →Go to Bb153
5. Dining out and food →Go to Bb153
6. Clothes and shoes →Go to Bb153
7. Others

Bb152. If others, please specify.

_______________________________

Bb153. [Not live together_ Offer of regular financial support] In the last calendar year (2017), while living separately did you give to your mother any regular financial support such as pocket money, living expenses or medical expenses?

1. Yes →Go to Bb154 ⑤ No →Go to Bb161

Bb154. [Not live together_ Offer of regular financial support] What was the monthly average amount of regular financial support you gave to your mother in the last calendar year (2017)? (unit: 10,000 Korean won)

_______________________________ MW [Range: 1 ~ 9997] →Go to Bb160

Don’t know →Go to Bb155~Bb159 unfolding

Refuse to answer →Go to Bb155~Bb159 unfolding

**< Bb155~Bb159. Unfolding bracket questions >**

[IWER: Go back to Bb154 and record if R answers the amount during the interview.]

Bb155. Did it amount to less than, about equal to or more than 10 MW (10,000 Korean won) in the last calendar year (2017)?

1. Less than 10 MW
2. About 10 MW

⑤ More than 10 MW

Bb156. Did it amount to less than, about equal to or more than 30 MW (10,000 Korean won) in the last calendar year (2017)?

1. Less than 30 MW
2. About 30 MW

⑤ More than 30 MW

Bb157. Did it amount to less than, about equal to or more than 50 MW (10,000 Korean won) in the last calendar year (2017)?

1. Less than 50 MW
2. About 50 MW

⑤ More than 50 MW

Bb158. Did it amount to less than, about equal to or more than 100 MW (10,000 Korean won) in the last calendar year (2017)?

1. Less than 100 MW
2. About 100 MW

⑤ More than 100 MW

Bb159. Did it amount to less than, about equal to or more than 200 MW (10,000 Korean won) in the last calendar year (2017)?

1. Less than 200 MW
2. About 200 MW

⑤ More than 200 MW

**Logic**

- **If Bb160 >= (12 - Bb015), do hard checking.**

Bb160. [Not live together_ Offer of regular financial support] How many month(s) did you give regular financial support to your mother in the last calendar year (2017)? (unit: month)

[IWER: If you alternated between co-residing and not co-residing in 2017, please consider only the financial support you gave while you did not live together]

_______________________________ months [Range:0~12]

Bb161. [Not live together_ Offer of occasional financial support] In the last calendar year (2017), while living separately did you give to your mother any occasional financial support such as pocket money, living expenses or medical expenses?

1. Yes →Go to Bb162 ⑤ No →Go to Bb168

Bb162. What was the total amount of occasional financial support you gave to your mother in the last calendar year (2017)?

[IWER: Please enter ‘100,000,000 Korean won (one hundred million won)’ first then, enter ’10,000 Korean won (MW)’.]

______one hundred million won [Range: 1~9] ________MW [Range: 0 ~ 9997] → Bb168

Don’t know → Go to Bb163~Bb167 unfolding

Refuse to answer → Go to Bb163~Bb167 unfolding

**< Bb163~Bb167. Unfolding bracket questions >**

[IWER: Go back to Bb162 and record if R answers the amount during the interview.]

Bb163. Did it amount to less than, about equal to or more than 100 MW (10,000 Korean won) in the last calendar year (2017)?

1. Less than 100 MW
2. About 100 MW

⑤ More than 100 MW

Bb164. Did it amount to less than, about equal to or more than 300 MW (10,000 Korean won) in the last calendar year (2017)?

1. Less than 300 MW
2. About 300 MW

⑤ More than 300 MW

Bb165. Did it amount to less than, about equal to or more than 500 MW (10,000 Korean won) in the last calendar year (2017)?

1. Less than 500 MW
2. About 500 MW

⑤ More than 500 MW

Bb166. Did it amount to less than, about equal to or more than 1,000 MW (10,000 Korean won) in the last calendar year (2017)?

1. Less than 1,000 MW
2. About 1,000 MW

⑤ More than 1,000 MW

Bb167. Did it amount to less than, about equal to or more than 2,000 MW (10,000 Korean won) in the last calendar year (2017)?

1. Less than 2,000 MW
2. About 2,000 MW

⑤ More than 2,000 MW

Bb168. . [Not live together_ Offer of non-financial support] In the last calendar year (2017), while living separately did you give any gifts or goods (not cash) to your mother?

1. Yes →Go to Bb169 ⑤ No →Go to Bb171

Bb169. What was the type of non-financial support you gave to your mother in the last calendar year (2017)? (Select multiple responses)

1. Leisure (e.g. travel) →Go to Bb171 or Bb185
2. Health‐related products (e.g. vitamins, equipment, etc) → Go to Bb171 or Bb185
3. Household items → Go to Bb171 or Bb185
4. Electronics → Go to Bb171 or Bb185
5. Dining out and food → Go to Bb171 or Bb185
6. Clothes and shoes → Go to Bb171 or Bb185
7. Others

Bb170. If others, please specify.

_______________________________ → Go to Bb171 or Bb185

**<Transfer to R’s mother who lived with R>**

**Logic**

- **If R lived with his/her mother for more than one month in 2017, then ask Bb171~Bb184.**

Bb171. [Live together_Receipt of financial support] In the last calendar year (2017), did you receive any financial support from your mother while living together?

[IWER: Provision of dwelling space, food, etc., is excluded. Financial support includes pocket money, medical expenses, travel expenses, business funds, etc. Paying for the bill instead is included.]

1. Yes ⑤ No →Go to Bb178

Bb172. [Live together_Receipt of financial support] If so, what was the total amount of financial support you received from your mother in the last calendar year (2017)?

[IWER: Please enter ‘100,000,000 Korean won (one hundred million won)’ first then, enter ’10,000 Korean won (MW)’.]

______one hundred million won [Range: 1~9] _______ MW [Range: 0 ~ 9997] → Bb178

Don’t know → Bb173~Bb177 unfolding

Refuse to answer → Bb173~Bb177 unfolding

**< Bb173~Bb177. Unfolding bracket questions >**

[IWER: Go back to Bb172 and record if R answers the amount during the interview.]

Bb173. Did it amount to less than, about equal to or more than 100 MW (10,000 Korean won)?

1. Less than 100 MW
2. About 100 MW

⑤ More than 100 MW

Bb174. Did it amount to less than, about equal to or more than 300 MW (10,000 Korean won)?

1. Less than 300 MW
2. About 300 MW

⑤ More than 300 MW

Bb175. Did it amount to less than, about equal to or more than 500 MW (10,000 Korean won)?

1. Less than 500 MW
2. About 500 MW

⑤ More than 500 MW

Bb176. Did it amount to less than, about equal to or more than 1,000 MW (10,000 Korean won)?

1. Less than 1,000 MW
2. About 1,000 MW

⑤ More than 1,000 MW

Bb177. Did it amount to less than, about equal to or more than 2,000 MW (10,000 Korean won)?

1. Less than 2,000 MW
2. About 2,000 MW

⑤ More than 2,000 MW

Bb178. [Live together_ Offer of financial support] In the last calendar year (2017), did you give any financial support to your mother while living together?

[IWER: Provision of dwelling space, food, etc., is excluded. Financial support includes pocket money, medical expenses, travel expenses, business funds, etc. Paying for the bill instead is included.]

1. Yes ⑤ No → Go to Bb185

Bb179. [Live together_ Offer of financial support] If so, what was the total amount of financial support you gave to your mother in the last calendar year (2017)?

[IWER: Please enter ‘100,000,000 Korean won (one hundred million won)’ first then, enter ’10,000 Korean won (MW)’.]

______one hundred million won [Range: 1~9] ________ MW [Range: 0 ~ 9997] → Bb185

Don’t know → Go to Bb180~Bb184 unfolding

Refuse to answer → Go to Bb180~Bb184 unfolding

**< Bb180~Bb184. Unfolding bracket questions >**

[IWER: Go back to Bb179 and record if R answers the amount during the interview.]

Bb180. Did it amount to less than, about equal to or more than 100 MW (10,000 Korean won)?

1. Less than 100 MW
2. About 100 MW

⑤ More than 100 MW

Bb181. Did it amount to less than, about equal to or more than 300 MW (10,000 Korean won)?

1. Less than 300 MW
2. About 300 MW

⑤ More than 300 MW

Bb182. Did it amount to less than, about equal to or more than 500 MW (10,000 Korean won)?

1. Less than 500 MW
2. About 500 MW

⑤ More than 500 MW

Bb183. Did it amount to less than, about equal to or more than 1,000 MW (10,000 Korean won)?

1. Less than 1,000 MW
2. About 1,000 MW

⑤ More than 1,000 MW

Bb184. Did it amount to less than, about equal to or more than 2,000 MW (10,000 Korean won)?

1. Less than 2,000 MW
2. About 2,000 MW

⑤ More than 2,000 MW

**<Transfer by other family member>**

Bb185. In the last calendar year (2017), while living separately did you receive any financial support such as pocket money, living expenses or medical expenses or gifts from any family member, other than your parents and children(e.g. parents-in-law, siblings, siblings-in-law, grandchildren, etc.)?

| **[IWER]**  **<Regular support/ occasional support>**   - Regular support: monetary transfer regularly repeated in a certain time interval (e.g. each month, every two months) - Occasional support: monetary transfer irregularly occurred except birthday allowance or holiday allowances (e.g. paying for medical bills, schooling, occasional allowances)   **<Financial support/ Non-financial support>**   - Financial support: receiving cash or helping pay bills, or covering specific costs for medical care, insurance, schooling, down payment for a home or rent, etc. - Non-financial support: gifts or goods (e.g. food, clothes etc.) received   * Only include financial/ non-financial support from other family members who don’t live together for more than one month in the last year 2017 |
| --- |

1. Yes, I received financial support.

⑤ No, I did not receive any financial support →Go to Bb204

Bb186. From whom did you receive financial support? Please identify all transfer givers. (Select multiple responses)

[IWER: R is asked about financial/ non-financial support from other family members while not living with them for more than one month in the last calendar year (2017).]

1. Mother-in-law →Go to Bb188
2. Father-in-law →Go to Bb188

[27~46] Sibling list →Go to Bb188

[47] Brother-in-law/sister-in-law →Go to Bb188

[48] Son-in-law/daughter-in-law →Go to Bb188

[49] Grandchildren

[50] Other relatives

Bb187. If others, please specify.

_______________________________

**Logic**

- **Loop Bb188 ~Bb203 as many times as the number of transfer givers in Bb186.**

Bb188. What kind of financial help did you receive from [giver’s name chosen in Bb132]? Choose all that apply. (Select multiple responses)

1. Regular financial support
2. Occasional financial support
3. Non‐financial support (Ex- gifts, food, clothes, home appliances etc.)

→ If code 1 chosen, go to Bb189

→ If code 2 chosen, go to Bb196

→ If code 3 chosen, go to Bb202

**Logic**

- **If Bb188=①, ①②, ①③, ①②③, then ask Bb189.**

Bb189. [Not live together_Receipt of regular financial support] Then, what was the monthly average amount of regular financial support you received from [giver’s name chosen] in the last calendar year (2017)? (unit: 10,000 Korean won)

_______________________________ MW [Range: 1 ~ 9997] →Go to Bb195

Don’t know →Go to Bb190~Bb194 unfolding

Refuse to answer →Go to Bb190~Bb194 unfolding

**< Bb190~Bb194. Unfolding bracket questions >**

[IWER: Go back to Bb189 and record if R answers the amount during the interview.]

Bb190. Did it amount to less than, about equal to or more than 10 MW (10,000 Korean won) in the last calendar year (2017)?

1. Less than 10 MW
2. About 10 MW

⑤ More than 10 MW

Bb191. Did it amount to less than, about equal to or more than 30 MW (10,000 Korean won) in the last calendar year (2017)?

1. Less than 30 MW
2. About 30 MW

⑤ More than 30 MW

Bb192. Did it amount to less than, about equal to or more than 50 MW (10,000 Korean won) in the last calendar year (2017)?

1. Less than 50 MW
2. About 50 MW

⑤ More than 50 MW

Bb193. Did it amount to less than, about equal to or more than 100 MW (10,000 Korean won) in the last calendar year (2017)?

1. Less than 100 MW
2. About 100 MW

⑤ More than 100 MW

Bb194. Did it amount to less than, about equal to or more than 200 MW (10,000 Korean won) in the last calendar year (2017)?

1. Less than 200 MW
2. About 200 MW

⑤ More than 200 MW

Bb195. [Not live together_Receipt of regular financial support] How many month(s) did you receive regular financial support from [giver’s name chosen] in the last calendar year (2017)?

[IWER: If you alternated between co-residing and not co-residing in 2017, please consider only the financial support you received while you did not live together]

_______________________________ months [Range:0~12]

**Logic**

- **If Bb188=②, ②③, ①②, ①②③, then ask Bb196.**

Bb196. [Not live together_ Receipt of occasional financial support] What was the total amount of occasional financial support you received from [giver’s name chosen] in the last calendar year (2017)?

[IWER: Please enter ‘100,000,000 Korean won (one hundred million won)’ first then, enter ’10,000 Korean won (MW)’.]

______one hundred million won [Range: 1~9] ________ MW [Range: 0 ~ 9997]

Don’t know →Go to Bb197~Bb201 unfolding

Refuse to answer →Go to Bb197~Bb201 unfolding

**< Bb197~Bb201. Unfolding bracket questions >**

[IWER: Go back to Bb196 and record if R answers the amount during the interview.]

Bb197. Did it amount to less than, about equal to or more than 100 MW (10,000 Korean won) in the last calendar year (2017)?

1. Less than 100 MW
2. About 100 MW

⑤ More than 100 MW

Bb198. Did it amount to less than, about equal to or more than 300 MW (10,000 Korean won) in the last calendar year (2017)?

1. Less than 300 MW
2. About 300 MW

⑤ More than 300 MW

Bb199. Did it amount to less than, about equal to or more than 500 MW (10,000 Korean won) in the last calendar year (2017)?

1. Less than 500 MW
2. About 500 MW

⑤ More than 500 MW

Bb200. Did it amount to less than, about equal to or more than 1,000 MW (10,000 Korean won) in the last calendar year (2017)?

1. Less than 1,000 MW
2. About 1,000 MW

⑤ More than 1,000 MW

Bb201. Did it amount to less than, about equal to or more than 2,000 MW (10,000 Korean won) in the last calendar year (2017)?

1. Less than 2,000 MW
2. About 2,000 MW

⑤ More than 2,000 MW

**Logic**

- **If Bb188=③, ①③, ②③, ①②③, then go to Bb202.**

Bb202. [Not live together_ Receipt of non-financial support] If you have received, what was the type of non-financial support you received from [giver’s name chosen] in the last calendar year (2017)? Choose all that apply. (Select multiple responses.)

1. Leisure (e.g. travel) →Go to Bb204
2. Health-related products (e.g. vitamins, equipment, etc.) →Go to Bb204
3. Household items →Go to Bb204
4. Electronics →Go to Bb204
5. Dining out and food →Go to Bb204
6. Clothes and shoes →Go to Bb204
7. Others

Bb203. If others, please specify.

_______________________________

Bb204. In the last calendar year (2017), while living separately did you give financial/ non-financial support to any family members, other than your parents and children, (e.g. parents-in-law, siblings, siblings-in-law, grandchildren, etc.)?

[IWER: R is asked about financial/ non-financial support from other family members who don’t live together for more than one month in the last year 2017.]

1. Yes, I gave financial support.

⑤ No, I did not give any financial support. →Go to Bb223

Bb205. To whom did you give financial help? Please identify all transfer receivers. (Select multiple responses)

[IWER: R is asked about financial/ non-financial support from other family members who don’t live together for more than one month in the last year 2017.]

1. Mother-in-law →Go to Bb207
2. Father-in-law →Go to Bb207

[27~46] Sibling list →Go to Bb207

[47] Brother-in-law/sister-in-law →Go to Bb207

[48] Son-in-law/daughter-in-law →Go to Bb207

[49] Grandchildren →Go to Bb207

[50] Other relatives

Bb206. If others, please specify.

_______________________________

**Logic**

- **Loop Bb207~Bb222 as many times as the number of transfer receivers in Bb205.**

Bb207. What kind of financial support did you give to [receiver’s name chosen inBb151]? Choose all that apply. (Select multiple responses)

1. Regular financial support
2. Occasional financial support
3. Non‐financial support (Ex- gifts, food, clothes, home appliances)

→ If code 1 chosen, go to Bb208

→ If code 2 chosen, go to Bb215

→ If code 3 chosen, go to Bb221

**Logic**

- **If Bb207=①, ①②, ①③, ①②③, then ask Bb208.**

Bb208. [Not live together_ Offer of regular financial support] What was the monthly average amount of regular financial support you gave to [receiver’s name chosen] in the last calendar year (2017)? (unit: 10,000 Korean won)

_______________________________ MW [Range: 1~9997] →Go to Bb214

Don’t know →Go to Bb209~Bb213 unfolding

Refuse to answer →Go to Bb209~Bb213 unfolding

**< Bb209~Bb213. Unfolding bracket questions >**

[IWER: Go back to Bb208 and record if R answers the amount during the interview.]

Bb209. Did it amount to less than, about equal to or more than 10 MW (10,000 Korean won) in the last calendar year (2017)?

1. Less than 10 MW
2. About 10 MW

⑤ More than 10 MW

Bb210. Did it amount to less than, about equal to or more than 30 MW (10,000 Korean won) in the last calendar year (2017)?

1. Less than 30 MW
2. About 30 MW

⑤ More than 30 MW

Bb211. Did it amount to less than, about equal to or more than 50 MW (10,000 Korean won) in the last calendar year (2017)?

1. Less than 50 MW
2. About 50 MW

⑤ More than 50 MW

Bb212. Did it amount to less than, about equal to or more than 100 MW (10,000 Korean won) in the last calendar year (2017)?

1. Less than 100 MW
2. About 100 MW

⑤ More than 100 MW

Bb213. Did it amount to less than, about equal to or more than 200 MW (10,000 Korean won) in the last calendar year (2017)?

1. Less than 200 MW
2. About 200 MW

⑤ More than 200 MW

Bb214. [Not live together_ Offer of regular financial support] How many month(s) did you give regular financial support to [receiver’s name chosen] in the last calendar year (2017)?

[IWER: If you alternated between co-residing and not co-residing in 2017, please consider only the financial support you gave while you did not live together]

_______________________________ months [Range:1~12]

**Logic**

- **If Bb207=②, ②③, ①②, ①②③, then ask Bb215.**

Bb215. [Not live together_ Offer of occasional financial support] What was the total amount of occasional financial support you gave to [receiver’s name chosen] in the last calendar year (2017)?

[IWER: Please enter ‘100,000,000 Korean won (one hundred million won)’ first then, enter ’10,000 Korean won (MW)’.]

______one hundred million won [Range: 1~9] _________MW [Range: 0 ~ 9997]

Don’t know →Go to Bb216~Bb220 unfolding

Refuse to answer →Go to Bb216~Bb220 unfolding

**< Bb216~Bb220. Unfolding bracket questions >**

[IWER: Go back to Bb215 and record if R answers the amount during the interview.]

Bb216. Did it amount to less than, about equal to or more than 100 MW (10,000 Korean won) in the last calendar year (2017)?

1. Less than 100 MW
2. About 100 MW

⑤ More than 100 MW

Bb217. Did it amount to less than, about equal to or more than 300 MW (10,000 Korean won) in the last calendar year (2017)?

1. Less than 300 MW
2. About 300 MW

⑤ More than 300 MW

Bb218. Did it amount to less than, about equal to or more than 500 MW (10,000 Korean won) in the last calendar year (2017)?

1. Less than 500 MW
2. About 500 MW

⑤ More than 500 MW

Bb219. Did it amount to less than, about equal to or more than 1,000 MW (10,000 Korean won) in the last calendar year (2017)?

1. Less than 1,000 MW
2. About 1,000 MW

⑤ More than 1,000 MW

Bb220. Did it amount to less than, about equal to or more than 2,000 MW (10,000 Korean won) in the last calendar year (2017)?

1. Less than 2,000 MW
2. About 2,000 MW

⑤ More than 2,000 MW

**Logic**

- **If Bb207=③, ①③, ②③, ①②③, then ask Bb221.**

Bb221. [Not live together_ Offer of non-financial support] If you gave, what was the type of non- financial support you gave to [receiver’s name chosen] in the last calendar year (2017)? Choose all that apply. (Select multiple responses.)

1. Leisure (e.g. travel) →Go to Bb223
2. Health-related products (e.g. vitamins, equipment, etc) →Go to Bb223
3. Household items →Go to Bb223
4. Electronics →Go to Bb223
5. Dining out and food →Go to Bb223
6. Clothes and shoes →Go to Bb223
7. Others

Bb222. If others, please specify.

_______________________________

**<The provision of care for the ADL/IADL>**

Bb223. Are there any family members over the age of 10 (spouse, parents, siblings, children, and spouse’s parents and siblings) who have any difficulty doing each of the everyday activities? Everyday activities includes dressing, washing face and hair/brushing teeth, bathing or showering, eating, getting across a room from getting out of bed, using the toilet and controlling urination or defecation. Please identify all family members with those difficulties. Choose all that apply. (Select multiple responses.)

1. Spouse
2. Mother
3. Father
4. Mother-in-law
5. Father-in-law

[7~26] Children list

[27~46] Sibling list

[47] Brother-in-law/sister-in-law

[48] Son-in-law/daughter-in-law

[49] Grandchildren

[50] Other relatives

[51] No member with ADL difficulties →Go to Bb227

Bb224. Did you care for any of [names listed in Bb169] during the past one year (not calendar year)? If so, whom did you help? Choose all family members you cared for. (Select multiple responses.)

[IWER: Past one year means one year ago from now, not 2017.]

1. Spouse
2. Mother
3. Father
4. Mother-in-law
5. Father-in-law

[7~26] Children list

[27~46] Sibling list

[47] Brother-in-law/sister-in-law

[48] Son-in-law/daughter-in-law

[49] Grandchildren

[50] Other relatives

[53] No, didn’t help (others helped caring e.g. helpers) →Go to Bb227

**Logic**

- **Loop Bb225~Bb226 as many times as the number of people in Bb224.**

Bb225.Roughly how many hours per week did you care for [name chosen in Bb224] during the past one year? (unit: an average hour per week)

[IWER: Past one year means one year ago from now, not 2017.]

_______________________________ Average hours per week [Range: 1~168]

Bb226. How many weeks did you care for [name chosen in Bb170] during the past one year? (unit: a week)

[IWER: Calculate 1 month as 4 weeks, 6 months as 26 weeks, 1 year as 52 weeks. For example, 5 months is converted into 20 weeks, and enter 30 weeks for 7 months because it is equal to 6 months (26 weeks) plus 1 month (4 weeks). Past one year means one year ago from now, not 2017.]

_______________________________ weeks [Range: 1~52]

Bb227. Are there any family members over the age of 10 (spouse, parents, parents-in-law, siblings and/or children) who are not living with you with any difficulty doing a few other activities due to physical, mental, emotional problems? Other activities include personal grooming, cleaning and tidying up the house, making bed and washing dishes, preparing meals, doing laundry, going out short distance, using transportation, shopping, managing money, making phone calls, and taking medications. If yes, did you help anyone for these activities during the past one year? If you did, whom did you help? Choose all family members you helped. (Select multiple responses.)

[IWER: Past one year means one year ago from now, not 2017.]

1. Spouse
2. Mother
3. Father
4. Mother-in-law
5. Father-in-law

[7~26] Children list

[27~46] Sibling list

[47] Brother-in-law/sister-in-law

[48] Son-in-law/daughter-in-law

[49] Grandchildren

[50] Other relatives

[54] No member with such difficulties →Go to Bb230

**Logic**

- **Loop through Bb228~Bb229 as many times as the number of people in Bb227.**

Bb228. Roughly how many hours per week did you help out [name chosen in Bb227] during the past one year? (unit: an average hour per week)

[IWER: Past one year means one year ago from now, not 2017.]

_______________________________ Average hours per week [Range: 1~168]

Bb229. How many weeks did you care for [name chosen in Bb173] during the past one year?

[IWER: Calculate 1 month as 4 weeks, 6 months as 26 weeks, 1 year as 52 weeks. For example, 5 months is converted into 20 weeks, and enter 30 weeks for 7 months because it is equal to 6 months (26 weeks) plus 1 month (4 weeks). And one year means one year ago from now, not 2017.]

_______________________________weeks [Range: 1~52]

Bb230. [IWER: How often did R receive assistance in answering section Bb–FAMILY AND FAMILY TRANSFER (PARENTS & SIBLINGS)?]

1. Never → Go to section C
2. A few times → Go to section C
3. Most of the time → Go to section C
4. The section was done by a proxy respondent

Bb231. If done by a proxy respondent, what is the proxy’s relationship to R? Please answer in view of the proxy.

1. Spouse
2. Mother
3. Father
4. Mother-in-law
5. Father-in-law
6. Brother/sister
7. Brother-in-law/sister-in-law
8. Son/daughter
9. Son-in-law/Daughter-in-law
10. Grandchild
11. Other relatives
12. Helper or other non-relatives

→ Go to section C

(Bb_end) [IWER] Please enter code 1 for completion of family (parents and siblings) section.

1. Completion

# **C1. HEALTH STATUS**

(C_Intro) [IWER] Now, this is to start C. Health section.

1. Check

**Logic**

- **C001 and C159 are asked at the start and end of HEALTH section, but in a random order.**

C001. Next I have some questions about your health. How would you describe your current healthy status?

1. Very good
2. Good
3. Fair
4. Bad
5. Very bad

C159. Next I have some questions about your health. How would you describe your current healthy status?

1. Excellent
2. Very good
3. Good
4. Fair
5. Poor

C002. Compared with your health in the previous interview (___ month ___ day ___ year), would you say that your health is better now, about the same, or worse?

1. Much better
2. Somewhat better
3. Same
4. Somewhat worse
5. Much worse

**Logic**

- **If R said in the previous interview that he/she didn’t receive a disability diagnosis, ask C003. Otherwise, ask C005.**

C003. Have you ever received a disability diagnosis from a doctor since the previous interview?

1. Yes, received a disability diagnosis
2. No, haven't received a disability diagnosis → Go to C005

C004. What was your type of disability? Choose all that apply.

1. Crippled disorder
2. Brain lesions
3. Visual handicap
4. Hearing impairment
5. Speech disorder
6. Renal disorder
7. Heart disorder
8. Intellectual and developmental disability
9. Others

C004-1. If others, please specify.

_______________________________

C005. Does your health condition hamper you doing work?

1. Yes, very much so
2. Yes, to some degree
3. No, not much
4. No, not at all

**< Chronic Disease >**

**< High blood pressure/ hypertension >**

**Logic**

- **If R said that he/she didn’t receive a diagnosis of high blood pressure or 'Don’t know/ Refuse to answer' in the previous interview, then ask C006. If R received such a diagnosis, then ask C008.**

C006. Has a doctor ever told you that you have high blood pressure or hypertension since the previous interview(___ month ___ day ___ year)?

1. Yes ⑤ No →Go to C011

C007. When was your high blood pressure or hypertension diagnosed for the first time? (unit: year and month combined into 6 digits)

[IWER: Enter the year and month using 6 digits. For example, mark 201801 for January 2018. If the month is not clear, enter 201800.]

____________________________________________________

[Range: 200600~201811] →Go to C009

C008. How have your symptoms of high blood pressure or hypertension changed since the previous interview(___ month ___ day ___ year)?

1. Completely recovered
2. Improved
3. Same
4. Worsened
5. Greatly worsened

C009. Are you currently taking any medication or receiving treatment to lower your high blood pressure?

1. Yes ⑤ No

C010. Does your high blood pressure or hypertension limit your daily activities?

1. Yes ⑤ No

**< Diabetes >**

**Logic**

- **If R said that he/she didn’t receive a diabetes diagnosis or 'Don’t know/ Refuse to answer' in the previous interview, then ask C011. If R received such a diagnosis, then ask C013.**

C011. Has a doctor ever told you that you have diabetes or high blood sugar since the previous interview(___ month ___ day ___ year)?

1. Yes ⑤ No →Go to C016

C012. When was your diabetes or high blood sugar diagnosed for the first time? (unit: year and month combined into 6 digits)

[IWER: Enter the year and month using 6 digits. For example, mark 201801 for January 2018. If the month is not clear, enter 201800.]

____________________________________________________

[Range: 200600~201811] →Go to C014

C013. How have your symptoms of diabetes or high blood sugar changed since the previous interview(___ month ___ day ___ year)?

1. Completely recovered
2. Improved
3. Same
4. Worsened
5. Greatly worsened

C014. Are you currently taking any medication or receiving treatment to treat your diabetes or stabilize your blood sugar level?

1. Yes ⑤ No

C015. Does your diabetes limit your daily activities?

1. Yes ⑤ No

**< Cancer or malignant tumor >**

**Logic**

- **If R said that he/she didn’t receive a diagnosis of cancer or a malignant tumor or 'Don’t know/ Refuse to answer' in the previous interview, then ask C016. If R received such a diagnosis, then ask C020.**

C016. Has a doctor ever told you that you have cancer or malignant tumor (including minor skin cancer) since the previous interview(___ month ___ day ___ year)?

1. Yes ⑤ No →Go to C023

C017. When was your cancer diagnosed for the first time? (unit: year and month combined into 6 digits)

[IWER: Enter the year and month using 6 digits. For example, mark 201801 for January 2018. If the month is not clear, enter 201800.]

____________________________________________________

[Range: 200600~201811]

C018. In which organ or part of your body do you have cancer?

[IWER: If cancer has spread out, please tell in which organ or part your cancer started]

1. Liver →Go to C021
2. Stomach →Go to C021
3. Lung →Go to C021
4. Colon →Go to C021
5. Thyroid →Go to C021
6. Breast →Go to C021
7. Cervix →Go to C021
8. Ovary →Go to C021
9. Prostate →Go to C021
10. Others

C019. If others, please specify.

_______________________________→Go to C021

C020. How have your symptoms of cancer or malignant tumor (including minor skin cancer) changed since the previous interview(___ month ___ day ___ year)?

1. Completely recovered
2. Improved
3. Same
4. Worsened
5. Greatly worsened

C021. Are you currently taking any medication to alleviate your symptoms (pain, nausea, rash, etc.) or receiving cancer treatment such as chemotherapy?

1. Yes ⑤ No

C022. Does your cancer limit your daily activities?

1. Yes ⑤ No

**< Chronic lung disease >**

**Logic**

- **If R said that he/she didn’t receive a diagnosis of chronic lung disease, such as bronchitis or emphysema, or 'Don’t know/ Refuse to answer' in the previous interview, then ask C023. If R received such a diagnosis, then ask C025.**

C023. Has a doctor ever told you that you have chronic lung disease, such as bronchitis or emphysema since the previous interview(___ month ___ day ___ year)?

1. Yes ⑤ No →Go to C028

C024. When was your chronic lung disease, such as bronchitis or emphysema diagnosed for the first time? (unit: year and month combined into 6 digits)

[IWER: Enter the year and month using 6 digits. For example, mark 201801 for January 2018. If the month is not clear, enter 201800.]

____________________________________________________

[Range: 200600~201811] →Go to C026

C025. How have your symptoms of chronic lung disease, such as bronchitis or emphysema, changed since the previous interview(___ month ___ day ___ year)?

1. Completely recovered
2. Improved
3. Same
4. Worsened
5. Greatly worsened

C026. Are you currently taking any medication or receiving treatment in relation to your lung disease?

1. Yes ⑤ No

C027. Does your lung condition limit your daily activities?

1. Yes ⑤ No

**< Liver disease >**

**Logic**

- **If R said that he/she didn’t receive a diagnosis of liver disease (all types of liver disease except fatty liver)or 'Don’t know/ Refuse to answer' in the previous interview, then ask C028. If R received such a diagnosis, then ask C030.**

C028. Has a doctor ever told you that you have liver disease since the previous interview(___ month ___ day ___ year)? (All types of liver disease except fatty liver)

1. Yes ⑤ No →Go to C033

C029. When was your liver disease diagnosed for the first time? (unit: year and month combined into 6 digits)

[IWER: Enter the year and month using 6 digits. For example, mark 201801 for January 2018. If the month is not clear, enter 201800.]

___________________________________________

[Range: 200600~201811] →Go to C031

C030. How have your symptoms of liver disease (All types of liver disease except fatty liver) changed since the previous interview(___ month ___ day ___ year)?

1. Completely recovered
2. Improved
3. Same
4. Worsened
5. Greatly worsened

C031. Are you currently taking any medication or receiving treatment due to your liver disease?

1. Yes ⑤ No

C032. Does your liver disease limit your daily activities?

1. Yes ⑤ No

**< Heart disease >**

**Logic**

- **If R said that he/she didn’t receive a diagnosis of a heart attack, angina pectoris, myocardial infarction, congestive heart failure or any other heart disease, or 'Don’t know/ Refuse to answer' in the previous interview, then ask C033. If R received such a diagnosis, then ask C035.**

C033. Has a doctor ever told you that you have heart attack, coronary heart disease, angina, congestive heart failure, or other heart problem since the previous interview(___ month ___ day ___ year)?

1. Yes ⑤ No →Go to C038

C034. When was your heart related diseases diagnosed for the first time? (unit: year and month combined into 6 digits)

[IWER: Enter the year and month using 6 digits. For example, mark 201801 for January 2018. If the month is not clear, enter 201800.]

____________________________________________________

[Range: 200600~201811] →Go to C036

C035. How have your symptoms of heart disease changed since the previous interview(___ month ___ day ___ year)?

1. Completely recovered
2. Improved
3. Same
4. Worsened
5. Greatly worsened

C036. Are you currently taking any medication or receiving treatment due to your heart disease?

1. Yes ⑤ No

C037. Does your heart disease limit your daily activities?

1. Yes ⑤ No

**< Cerebrovascular disease >**

**Logic**

- **If R said that he/she didn’t receive a diagnosis of cerebrovascular disease or 'Don’t know/ Refuse to answer' in the previous interview, then ask C038. If R received such a diagnosis, then ask C040.**

C038. Has a doctor ever told you that you have cerebrovascular disease (cerebral apoplexy, cerebral hemorrhage, cerebral infarction, etc.) since the previous interview(___ month ___ day ___ year)?

1. Yes
2. Cerebral apoplexy suspect or transient ischemic attack
3. No →Go to C043

C039. When was your cerebrovascular disease (cerebral apoplexy, cerebral hemorrhage, cerebral infarction, etc.) diagnosed for the first time? (unit: year and month combined into 6 digits)

[IWER: Enter the year and month using 6 digits. For example, mark 201801 for January 2018. If the month is not clear, enter 201800.]

____________________________________________________

[Range: 200600~201811] →Go to C041

C040. How have your symptoms of cerebrovascular disease changed since the previous interview(___ month ___ day ___ year)?

1. Completely recovered
2. Improved
3. Same
4. Worsened
5. Greatly worsened

C041. Are you currently taking any medication or receiving treatment due to your cerebrovascular disease or its complications?

1. Yes ⑤ No

C042. Does your cerebrovascular disease limit your daily activities?

1. Yes ⑤ No

**< Emotional, Neurotic, Psychiatric problems>**

**Logic**

- **If R said that he/she didn’t receive a diagnosis of emotional, nervous, or psychiatric problems or 'Don’t know/ Refuse to answer' in the previous interview, then ask C043. If R received such a diagnosis, then ask C045.**

C043. Has a doctor ever told you that you have any emotional (depression, anxious disorder, etc.), nervous (insomnia, excess stress, etc.), or psychiatric problems (mental disorder, interpersonal problem, etc.) since the previous interview?

1. Yes ⑤ No →Go to C048

C044. When was your emotional, nervous or psychiatric problems diagnosed for the first time? (unit: year and month combined into 6 digits)

[IWER: Enter the year and month using 6 digits. For example, mark 201801 for January 2018. If the month is not clear, enter 201800.]

____________________________________________________

[Range: 200600~201811] →Go to C046

C045. How have your symptoms of emotional, nervous, or psychiatric problems changed since the previous interview(___ month ___ day ___ year)?

1. Completely recovered
2. Improved
3. Same
4. Worsened
5. Greatly worsened

C046. Are you currently taking any medication such as tranquilizers, antidepressants, sedatives or sleeping pills or receiving psychiatric or psychological treatment?

1. Yes ⑤ No

C047. Do your emotional, nervous or psychiatric problems limit your daily activities?

1. Yes ⑤ No

**< Arthritis or rheumatism >**

**Logic**

- **If R said that he/she didn’t receive a diagnosis of arthritis or rheumatism or 'Don’t know/ Refuse to answer' in the previous interview, then ask C048. If R received such a diagnosis, then ask C050.**

C048. Has a doctor ever told you that you have arthritis or rheumatism since the previous interview(___ month ___ day ___ year)?

1. Yes ⑤ No →Go to C053

C049. When was your arthritis or rheumatism diagnosed for the first time? (unit: year and month combined into 6 digits)

[IWER: Enter the year and month using 6 digits. For example, mark 201801 for January 2018. If the month is not clear, enter 201800.]

____________________________________________________

[Range: 200600~201811] →Go to C051

C050. How have your symptoms of arthritis or rheumatism changed since the previous interview (___ month ___ day ___ year)?

1. Completely recovered
2. Improved
3. Same
4. Worsened
5. Greatly worsened

C051. Are you currently taking any medication or receiving treatment for your arthritis or rheumatism?

1. Yes ⑤ No

C052. Does your arthritis or rheumatism limit your daily activities?

1. Yes ⑤ No

**< Dementia >**

Cadd_01. Has a doctor ever told you that you have dementia (such as senile dementia, Alzheimer's disease)?

1. Yes
2. Mild cognitive impairment
3. No →Go to C053

Cadd_02. When was your dementia dementia (such as senile dementia, Alzheimer's disease) diagnosed for the first time? (unit: year and month combined into 6 digits)

[IWER: 0000 and 00 months in a row of 6 digits. For example, enter 201801 for January 2018 and 201800 for the exact month.]

__________ [Range: 190000 ~ 201811]

Cadd_03. Are you currently taking any medication or receiving treatment for your dementia (such as senile dementia, Alzheimer's disease)?

1. Yes ⑤ No

Cadd_03_1. Does your dementia (such as senile dementia, Alzheimer's disease) limit your daily activities?

1. Yes ⑤ No

**< Traffic accident >**

C053. Have you ever been in a traffic accident and received medical treatment since the previous interview?

1. Yes ⑤ No →Go to C056

C054. When did the traffic accident happen? If you had more than two accidents, please answer about the most recent one. (unit: year and month combined into 6 digits)

[IWER: Enter the year and month using 6 digits. For example, mark 201801 for January 2018. If the month is not clear, enter 201800.]

____________________________________________________

[Range: 200600~201811]

C055. Does your injury caused by the traffic accident limit your daily activities?

1. Yes ⑤ No

**< Fall >**

C056. Have you ever got hurt from a fall since the previous interview?

1. Yes ⑤ No →Go to C061

C057. How many times have you had a fall accident since the previous interview? (unit: a time)

_______________________________ times [Range: 1~100]

C058. In your most recent fall, did you injure yourself seriously enough to need medical treatment?

1. Yes ⑤ No

C059. Have you ever fractured your hip in a fall accident?

1. Yes ⑤ No

C060. Does your injury or fracture in a fall accident limit your daily activities?

1. Yes ⑤ No

C061. In your daily life, how much do you worry about falling down?

① Not at all

③ A little bit

⑤ A lot

C062. Are there any activities that you refrain from doing due to the fear of falling down?

1. Yes ⑤ No

**< Prostatic disease (Male) >**

**Logic**

- **If R is male, then ask C063~C067 (Prostatic disease).**
- **If R said that he didn’t receive a diagnosis of prostatic disease or ‘Don’t know/ Refuse to answer’ in the previous interview, then ask C063. If R received such a diagnosis, then ask C065.**

C063. Has a doctor ever told you that you have prostatic disease since the previous interview?

1. Yes ⑤ No →Go to C074

C064. When was your prostatic disease diagnosed for the first time? (unit: year and month combined into 6 digits)

[IWER: Enter the year and month using 6 digits. For example, mark 201801 for January 2018. If the month is not clear, enter 201800.]

____________________________________________________

[Range: 200600~201811] →Go to C066

C065. How have your symptoms of prostatic disease changed since the previous interview(___ month ___ day ___ year)?

1. Completely recovered
2. Improved
3. Same
4. Worsened
5. Greatly worsened

C066. Are you currently taking any medication or receiving treatment for your prostate disease?

1. Yes ⑤ No

C067. Does your prostate disease limit your daily activities?

1. Yes →Go to C074 ⑤ No →Go to C074

**< Urinary incontinence disease (Female) >**

**Logic**

- **If R is female, then go to C068~C073 (Urinary incontinence disease).**
- **If R said that she didn’t receive a diagnosis of urinary incontinence or 'Don’t know/ Refuse to answer' in the previous interview, then ask C068. If R received such a diagnosis, then ask C069.**

C068. You may feel inconvenient with the following questions; however, it would be much appreciated if you would answer frank as much as possible. Have you lost any amount of urine beyond your control during the past one year?

1. Yes →Go to C070 ⑤ No →Go to C074

C069. How have your symptoms of leaking urine changed since the previous interview(___ month ___ day ___ year)?

1. Completely recovered
2. Improved
3. Same
4. Worsened
5. Greatly worsened

C070. On about how many days in the last month have you lost any urine? (unit: a day)

_______________________________ days [Range: 0~31]→Go to C073

Don’t know →Go to C071

Refuse to answer →Go to C071

C071. Was that more than 5 days?

1. Yes →Go to C072 ⑤ No →Go to C073

C072. Was that more than 15 days?

1. Yes ⑤ No

C073. Do you ever use any absorbent products such as pads, special garments, sanitary napkins, or toilet paper for your urine loss condition?

1. Yes ⑤ No

**< Digestive system disease >**

C074. Has a doctor ever told you that you have diseases of the digestive system such as

gastritis, gastric ulcer and duodenal ulcer?

1. Yes ⑤ No →Go to C078

C075. When was your digestive system disease diagnosed for the first time? (unit: year and month combined into 6 digits)

[IWER: Enter the year and month using 6 digits. For example, mark 201801 for January 2018. If the month is not clear, enter 201800.]

____________________________________________________

[Range: 200600~201811]

Cadd_04. How have your symptoms of digestive system disease changed since the previous interview (___ month ___ day ___ year)?

1. Completely recovered
2. Improved
3. Same
4. Worsened
5. Greatly worsened

C076. Are you currently taking any medication or receiving treatment for your digestive system disease?

1. Yes ⑤ No

C077. Does your digestive system disease limit your daily activities?

1. Yes ⑤ No

**< Disc Disease >**

C078. Has a doctor ever told you that you have any disc in neck or waist, except back pain or muscle pain?

1. Yes ⑤ No →Go to C082

C079. When was your disc diagnosed for the first time? (unit: year and month combined into 6 digits)

[IWER: Enter the year and month using 6 digits. For example, mark 201801 for January 2018. If the month is not clear, enter 201800.]

____________________________________________________

[Range: 200600~201811]

Cadd_05. How have your symptoms of disc disease changed since the previous interview (___ month ___ day ___ year)?

1. Completely recovered
2. Improved
3. Same
4. Worsened
5. Greatly worsened

C080. Are you currently taking any medication or receiving treatment for your disc?

1. Yes ⑤ No

C081. Does your disc limit your daily activities?

1. Yes ⑤ No

**< Eyesight >**

C082. Now I have some questions about your eyesight. Do you usually wear glasses or corrective lens?

① Yes

③ Visually disabled (blind)→Go to C90

⑤ No

C083. How good is your eyesight (using glasses or corrective lens as usual)?

1. Very good
2. Good
3. Fair
4. Bad
5. Very bad

C084. How good is your eyesight for seeing things at a distance, like recognizing a friend across a street?

1. Very good
2. Good
3. Fair
4. Bad
5. Very bad

C085. How good is your eyesight for seeing things up close, like reading a newspaper?

1. Very good
2. Good
3. Fair
4. Bad
5. Very bad

C086. Have you ever had cataract surgery since the previous interview?

1. Yes ⑤ No →Go to C088

C087. Have you had cataract surgery on both eyes or just one?

1. Only one eye ⑤ Both eyes

C088. Has a doctor ever treated you for glaucoma since the previous interview?

1. Yes ⑤ No

C089. Does your eyesight limit your daily activities?

1. Yes ⑤ No

**< Hearing >**

C090. Now I have some questions about your hearing. Do you ever wear a hearing aid?

1. Yes ⑤ No

C091. How good is your hearing? If you use a hearing aid, answer about your hearing when you wear it.

1. Very good
2. Good
3. Fair
4. Bad
5. Very bad

C092. Does your hearing limit your daily activities?

1. Yes ⑤ No

**< Dental health >**

C093. Now I have some questions about your dental health. Do you wear dentures?

1. Yes ⑤ No

**Logic**

- **If C093=①, then ask C094.**

C094. How easily can you chew solid foods, such as meat or apples, if you wear dentures?

1. Very well
2. Pretty well
3. Fair
4. Not well
5. Not at all

**Logic**

- **Answer C095 whether to wear dentures (C094)**

C095. How well can you chew solid foods, such as meat or apples, without dentures?

1. Very well
2. Pretty well
3. Fair
4. Not well
5. Not at all

Cadd_06. How many teeth do you have under the following conditions?

[IWER:

- The maximum number of natural teeth a person has is 32 including wisdom teeth.

- Enter '0' if not applicable

- For partial dentures, enter the correct number. Enter '100' for full dentures or '55' for full lower or upper dentures

- If the root of the teeth is alive, it is not a false tooth (procedures such as amalgam, resin, and gold teeth, treatment with the roots remaining is considered natural teeth).

- Teeth that are intentionally removed for therapeutic purposes, such as dentures and braces, are not included in the number of missing teeth

- Number of implant teeth __________
- Number of teeth in dentures _______________
- Number of teeth that need implant or denture treatment but are left missing ___________
- Number of wisdom teeth remaining now_________

**< Quality of life related to oral health >**

Cadd_07. How often did you limit the kinds or amounts of food you eat because of problems with your teeth or dentures?

1. Always
2. Very often
3. Often
4. Sometimes
5. Almost none
6. None

Cadd_08. How often did you have trouble biting or chewing different kinds of food, such as firm meat or apples?

1. Always
2. Very often
3. Often
4. Sometimes
5. Almost none
6. None

Cadd_09. How often were you able to swallow comfortably?

1. Always
2. Very often
3. Often
4. Sometimes
5. Almost none
6. None

Cadd_10. How often have your teeth or dentures prevented you from speaking the way you want?

1. Always
2. Very often
3. Often
4. Sometimes
5. Almost none
6. None

Cadd_11. How often were you able to eat anything without feeling discomfort?

1. Always
2. Very often
3. Often
4. Sometimes
5. Almost none
6. None

Cadd_12. How often did you limit contact with other people because of the condition of your teeth or dentures?

1. Always
2. Very often
3. Often
4. Sometimes
5. Almost none
6. None

Cadd_13. How often were you pleased or happy with the looks of your teeth and gums or dentures?

1. Always
2. Very often
3. Often
4. Sometimes
5. Almost none
6. None

Cadd_14. How often did you use medication to relieve pain or discomfort around your mouth?

1. Always
2. Very often
3. Often
4. Sometimes
5. Almost none
6. None

Cadd_15. How often were you worried or concerned about problems with your teeth, gums or dentures?

1. Always
2. Very often
3. Often
4. Sometimes
5. Almost none
6. None

Cadd_16. How often did you feel nervous or self- conscious because of problems with teeth, gums or dentures?

1. Always
2. Very often
3. Often
4. Sometimes
5. Almost none
6. None

Cadd_17. How often did you feel uncomfortable eating in front of other people because of problems with your teeth or dentures?

1. Always
2. Very often
3. Often
4. Sometimes
5. Almost none
6. None

Cadd_18. How often were your teeth or gums sensitive to hot, cold or sweet foods?

1. Always
2. Very often
3. Often
4. Sometimes
5. Almost none
6. None

**< Pain >**

C096. Now I have some questions about body pain. In what part of your body do you feel pain? Choose all the parts in which you are currently feeling pain. (Select multiple responses except 14)

1. Head (Headache) →Go to C097
2. Shoulder →Go to C098
3. Arm →Go to C099
4. Wrist →Go to C100
5. Fingers →Go to C101
6. Chest →Go to C102
7. Stomach (Stomachache)→Go to C103
8. Back →Go to C104
9. Buttocks →Go to C105
10. Leg →Go to C106
11. Knee →Go to C107
12. Ankle →Go to C108
13. Toes →Go to C109
14. No pain →Go to C111

C097. How bad is the headache?

① Mild

③ Moderate

⑤ Severe

C098. How bad is the shoulder pain?

① Mild

③ Moderate

⑤ Severe

C099. How bad is the arm pain?

① Mild

③ Moderate

⑤ Severe

C100. How bad is the wrist pain?

① Mild

③ Moderate

⑤ Severe

C101. How bad is the finger pain?

① Mild

③ Moderate

⑤ Severe

C102. How bad is the chest pain?

① Mild

③ Moderate

⑤ Severe

C103. How bad is the stomachache?

① Mild

③ Moderate

⑤ Severe

C104. How bad is the back pain?

① Mild

③ Moderate

⑤ Severe

C105. How bad is the buttocks pain?

① Mild

③ Moderate

⑤ Severe

C106. How bad is the leg pain?

① Mild

③ Moderate

⑤ Severe

C107. How bad is the knee pain?

① Mild

③ Moderate

⑤ Severe

C108. How bad is the ankle pain?

① Mild

③ Moderate

⑤ Severe

C109. How bad is the toe pain?

① Mild

③ Moderate

⑤ Severe

C110. Does the pain make it difficult for you to do daily activities?

1. Yes ⑤ No

C111. Do you have any other disease or health problem we have not talked about so far?

1. Yes ⑤ No →Go to C113

C112. What is that disease or health problem?

_______________________________

**< Others >**

C113. How much do you weigh? (unit: Kilogram)

_______________________________ kilograms [Range: 30~200]

C114. Did you gain or lose 5 or more kilograms during the past one year?

[IWER: Past one year means one year ago from now, not 2017.]

1. I gained weight
2. I lost weight
3. I gained weight and then lost it again
4. I lost weight and then gained it again
5. There was no change

C115. How tall are you? (unit: Centimeter)

_______________________________centimeters [Range: 70~210]

C116. The next few questions are about exercise. Do you work out more than once a week?

1. Yes →Go to C119 ⑤ No

C117. What is the main reason that you cannot exercise regularly?

1. Too busy to do exercise →Go to C122
2. No space or place to work out →Go to C122
3. Too lazy/ non-habituating →Go to C122
4. Do not like exercise →Go to C122
5. Never thought about exercise →Go to C122
6. Others

C118. If others, please specify.

_______________________________

C119. How often do you work out per week? (unit: a time/per week)

_______________________________times / per week [Range: 1~97]

C120. How long do you work out per session? (unit: a minutes)

[IWER: Calculate one hour as 60 minutes. For example, mark 150 minutes for 2 hours and 30 minutes.]

_______________________________minutes [Range: 1~1440]

C121. How long have you been working out regularly?

1. Less than 3 months
2. 4~6 months
3. 7 months~1 year
4. 1~2 years
5. 2~3 years
6. 3~4 years
7. 4~5 years
8. 5~6 years
9. 6~7 years
10. More than 7 years

C122. Now I am going to ask you about the meals you had for the last two days. Starting with yesterday, did you have three meals including breakfast, lunch and dinner? Choose all that apply. (Select multiple responses.)

[IWER: Caution! Choose only the meals R actually had.]

1. I had breakfast
2. I had lunch
3. I had dinner
4. I did not eat anything

C123. Thinking of the day before yesterday, did you have three meals including breakfast, lunch and dinner? Choose all that apply. (Select multiple responses.)

[IWER: Caution! Choose only the meals R actually had.]

1. I had breakfast
2. I had lunch
3. I had dinner
4. I did not eat anything

**< Smoking >**

**Logic**

- **If (R was identified as a smoker in the previous interview and C124=①) or (R was identified as a non-smoker in the previous interview and C124=①), then ask C125.**
- **If R was identified as a smoker in the previous interview and C124=⑤, then ask C129.**
- **If R was identified as a non-smoker in the previous interview and C124=⑤ and C125=⑤, then go to C130.**

C124. Have you smoked more than 5 packs of cigarettes (100 cigarettes) in total since the previous interview(___ month ___ day ___ year)?

1. Yes ⑤ No

C125. Do you smoke cigarettes now?

1. Yes ⑤ No →Go to C128

**Logic**

- **R was identified as a non-smoker in the previous interview and C125=①, then ask C126**

C126. When did you first start smoking? (unit: year and month combined into 6 digits)

[IWER: Calculate the year and month after hearing R’s response. For example, enter 201803 if R started smoking in March 2018.]

_______________________________ [Range: 200600~201811]

**Logic**

- **If C125=①, then ask C127.**

C127. How many cigarettes do you currently smoke a day? (unit: a cigarette)

[IWER: One pack is 20 cigarettes. For example, mark 30 for one and a half packs]

_______________________________ cigarette(s) per day [Range: 1~100]

**Logic**

- **If R was identified as a smoker in the previous interview and C125=⑤, then ask C128.**

C128. When you were smoking the most, how many cigarettes do you currently smoke a day? (unit: a cigarette)

[IWER: One pack is 20 cigarettes. For example, mark 30 for one and a half packs.]

_______________________________ cigarette(s) per day [Range: 1~200]

**Logic**

- **If R was identified as a smoker in the previous interview and C125=⑤, then ask C129**

C129. When did you stop smoking? (unit: year and month combined into 6 digits)

[IWER: Calculate the year and month after hearing R’s response. For example, enter 201803 if R started smoking in March 2018.]

_______________________________ [Range: 200600~201811] →Go to C130

**< Drinking >**

**Logic**

- **If R answered in the previous interview that he/she drank and C130=①, then ask C133.**
- **If R answered in the previous interview that he/she did not drink and C130=①, then ask C132.**
- **If R answered in the previous interview that he/she drank and C130=⑤, then ask C131.**
- **If R answered in the previous interview that he/she did not drink and C130=⑤, then ask Cadd_19.**

C130. Do you sometimes or often drink any alcoholic beverages, such as beer, wine, or Makgeolli (rice wine)?

1. Yes →Go to C132 (Non-drinker in 2016) or C133 (Drinker in 2016)
2. No →Go to C131 (Drinker in 2016) or Cadd_19 (Non-drinker in 2016)

**Logic**

- **If R answered in the previous interview that he/she drank and C130=⑤, then ask C131.**

C131. When did you quit drinking? (unit: year and month combined into 6 digits)

[IWER: Calculate the year and month after hearing R’s response. For example, enter 201803 if R started smoking in March 2018.]

_______________________________ [Range: 200600~201811] →Go to Cadd_19

**Logic**

- **If R answered in the previous interview that he/she did not drink and C130=①, then ask C132.**

C132. When did you start drinking? (unit: year and month combined into 6 digits)

[IWER: Calculate the year and month after hearing R’s response. For example, enter 201803 if R started smoking in March 2018.]

_______________________________ [Range: 200600~201811]

C133. Now, I am going to ask you how often and how much you drank during the past one year. By each type of alcoholic beverage, you can answer how often you drank per month on average, and how much you drank at a time on average. I will now tell you the type of alcoholic beverage. If you don’t drink that type, you can answer no.

1. Check

C134. How often did you drink Soju (Korean liquor) in a month?

1. None or less than once a month →Go to C136
2. Once a month
3. 2~3 times a month
4. Once a week
5. 2~3 times a week
6. 4~6 times a week
7. Everyday/ once a day
8. Everyday/ more than twice a day

C135. How many glasses of Soju did you drink at a time? (unit: a glass)

[1 bottle = 6.5 glasses, a glass of Soju =50cc]

_______________________________ Average glasses

C136. How often did you drink beer in a month?

1. None or less than once a month →Go to C138
2. Once a month
3. 2~3 times a month
4. Once a week
5. 2~3 times a week
6. 4~6 times a week
7. Everyday/ once a day
8. Everyday/ more than twice a day

C137. How many glasses of beer did you drink at a time? (unit: a glass)

[IWER: If R answered a bottle, ask size of a bottle (500cc or 1.5L). And then calculate the number of glasses by a glass of beer (220cc). For example, two bottles of beer are about four glasses]

[1bottle = 2.5 glasses of beer, a glass of beer = 220cc]

_______________________________ Average glasses

C138. How often did you drink Makgeolli (rice wine) in a month?

1. None or less than once a month →Go to C140
2. Once a month
3. 2~3 times a month
4. Once a week
5. 2~3 times a week
6. 4~6 times a week
7. Everyday/ once a day
8. Everyday/ more than twice a day

C139. How many glasses of Makgeolli did you drink at a time?

[IWER: 1 bottle = 7 glasses, a glass of Makgeoli= 240cc]

_______________________________ Average glasses

C140. How often did you drink whiskey and other liquors in a month?

1. None or less than once a month →Go to C142
2. Once a month
3. 2~3 times a month
4. Once a week
5. 2~3 times a week
6. 4~6 times a week
7. Everyday/ once a day
8. Everyday/ more than twice a day

C141. How many glasses of whiskey or liquor did you drink at a time?

[IWER: a glass of whiskey = 30cc, calculate the number of a glass by size of a bottle]

_______________________________ Average glasses

C142. How often did you drink wine in a month?

1. None or less than once a month →Go to C144
2. Once a month
3. 2~3 times a month
4. Once a week
5. 2~3 times a week
6. 4~6 times a week
7. Everyday/ once a day
8. Everyday/ more than twice a day

C143. How many glasses of wine did you drink at a time?

[IWER: 1 bottle = 8 glasses, a glass of wine = 90cc]

_______________________________ Average glasses

C144. Have you ever felt that you should quit drinking alcoholic beverages?

1. Yes ⑤ No

C145. Has anyone complained about your drinking habit?

1. Yes ⑤ No →Go to C147

C146. Have you ever been angry because other people complained about your drinking habit?

1. Yes ⑤ No

C147. Have you ever felt bad or guilty about drinking?

1. Yes ⑤ No

C148. Have you ever taken a drink as soon as you get up in the morning to calm your nerves or relieve a hangover?

1. Yes ⑤ No

**< Depression >**

Cadd_19. Have you ever had sad, blue or depression feelings which persist longer than 2 weeks during the past one year?

① Yes

③ Did not have such feelings because I was taking an anti-depressants

⑤ No

C149. Next I will ask you about your feelings and behavior during the last week. Please answer during last week, how often you felt or behaved in the following ways.

[IWER: from C149 to C158, please keep repeating “during last week how often did R feel”]

People were unfriendly.

1. Rarely or none of the time (less than one day)
2. Some or a little of time (1~2 days)
3. Occasionally or a moderate amount of time (3~4 days)
4. Most of all of the time (5~7 days)

C150. I felt sad.

1. Rarely or none of the time (less than one day)
2. Some or a little of time (1~2 days)
3. Occasionally or a moderate amount of time (3~4 days)
4. Most of all of the time (5~7 days)

C151. I felt depressed

1. Rarely or none of the time (less than one day)
2. Some or a little of time (1~2 days)
3. Occasionally or a moderate amount of time (3~4 days)
4. Most of all of the time (5~7 days)

C152. I felt that everything I did was an effort

1. Rarely or none of the time (less than one day)
2. Some or a little of time (1~2 days)
3. Occasionally or a moderate amount of time (3~4 days)
4. Most of all of the time (5~7 days)

C153. I was happy.

1. Rarely or none of the time (less than one day)
2. Some or a little of time (1~2 days)
3. Occasionally or a moderate amount of time (3~4 days)
4. Most of all of the time (5~7 days)

C154. I felt that people disliked me.

1. Rarely or none of the time (less than one day)
2. Some or a little of time (1~2 days)
3. Occasionally or a moderate amount of time (3~4 days)
4. Most of all of the time (5~7 days)

C155. My sleep was restless.

1. Rarely or none of the time (less than one day)
2. Some or a little of time (1~2 days)
3. Occasionally or a moderate amount of time (3~4 days)
4. Most of all of the time (5~7 days)

C156. I enjoyed life.

1. Rarely or none of the time (less than one day)
2. Some or a little of time (1~2 days)
3. Occasionally or a moderate amount of time (3~4 days)
4. Most of all of the time (5~7 days)

C157. I felt lonely.

1. Rarely or f of the time (less than one day)
2. Some or a little of time (1~2 days)
3. Occasionally or a moderate amount of time (3~4 days)
4. Most of all of the time (5~7 days)

C158. I could not get “going”

1. Rarely or none of the time (less than one day)
2. Some or a little of time (1~2 days)
3. Occasionally or a moderate amount of time (3~4 days)
4. Most of all of the time (5~7 days)

**Logic**

- **C001 and C159 are asked at the start and end of HEALTH section, but in a random order.**

C001. Next I have some questions about your health. How would you describe your current healthy status?

1. Very good
2. Good
3. Fair
4. Bad
5. Very bad

C159. Next I have some questions about your health. How would you describe your current healthy status?

1. Excellent
2. Very good
3. Good
4. Fair
5. Poor

C160. [IWER: How often did R receive assistance in answering section C1‐HEALTH STATUS?]

1. Never → Go to section C2.
2. A few times → Go to section C2.
3. Most of the time → Go to section C2.
4. The section was done by a proxy respondent

C161. If done by a proxy respondent, what is the proxy’s relationship to R? Please answer in view of the proxy

1. Spouse
2. Mother
3. Father
4. Mother-in-law
5. Father-in-law
6. Brother/sister
7. Brother-in-law/sister-in-law
8. Son/daughter
9. Son-in-law/Daughter-in-law
10. Grandchild
11. Other relatives
12. Helper or other non-relatives

→ Go to section C2.

# **C2. FUNCTIONAL LIMITATIONS AND HELPERS**

**<ADL (Ability of Daily Life) >**

C201. Now I would like to know how much you need someone to help you with very simple daily activities. Please answer based on your activities over the past one week. If you are now receiving help with doing such activities due to an illness or injury but expect the situation to last less than three months, then answer that you don’t need help. Let me start.

Can you do dressing including taking clothes out of a closet and buttoning or zipping up all by yourself? Or do you need someone to help you with that?

1. No, I don't need any help.

③ Yes, I need help to some extent.

⑤ Yes, I need help in every respect.

C202. Can you do washing your face and hair and brushing your teeth all by yourself? Or do you need someone to help you with that?

1. No, I don't need any help.

③ Yes, I need help to some extent.

⑤ Yes, I need help in every respect.

C203. Can you do bathing or showering (you may not be able to scrub your back) all by yourself? Or do you need someone to help you with that?

1. No, I don't need any help.

③ Yes, I need help to some extent.

⑤ Yes, I need help in every respect.

C204. Can you do eating (you may use forks or other tools) all by yourself? Or do you need someone to help you with that?

1. No, I don't need any help.

③ Yes, I need help to some extent.

⑤ Yes, I need help in every respect.

C205. Can you do getting in/ out of bed and walking across a room (you may use equipment or devices) all by yourself? Or do you need someone to help you with that?

1. No, I don't need any help.

③ Yes, I need help to some extent.

⑤ Yes, I need help in every respect.

C206. Can you do using the toilet (including taking off clothes and cleaning oneself after defecating or urinating. You may use a portable toilet.) all by yourself? Or do you need someone to help you with that?

1. No, I don't need any help.

③ Yes, I need help to some extent.

⑤ Yes, I need help in every respect.

C207. Can you use the toilet without spilling out urine (you may use a catheter (conduit) or a pouch by yourself) all by yourself? Or do you need someone to help you with that?

1. No, I don't need any help.

③ Yes, I need help to some extent.

⑤ Yes, I need help in every respect.

**<IADL (Instrumental Ability of Daily Life) >**

C208. Now I will ask questions about a bit more difficult activities. Please answer based on your activities over the past one week. If you are temporarily receiving help with doing such activities for a certain reason but expect the situation to last less than three months, then answer that you don’t need help.

Can you do personal grooming including brushing hair, clipping nails/toenails, putting on make-up (female) and shaving (male) all by yourself? Or do you need someone to help you with that?

1. No, I don't need any help.

③ Yes, I need help to some extent.

⑤ Yes, I need help in every respect.

C209. Do you need someone to help you with household chores, such as cleaning or tidying up the house, making bed and washing dishes?

[IWER: If R can broom floor but cannot mop or if R can fold light blankets but cannot handle heavy beddings, then mark ③]

1. No, I don't need any help.

③ Yes, I need help to some extent.

⑤ Yes, I need help in every respect.

C210. Do you need someone to help you with preparing meals? Preparing meals means preparing ingredients, cooking, and setting the table.

[IWER: If R cooks rice but another person prepares ingredients or side dishes, then mark ③.]

1. No, I don't need any help.
2. Yes, I need help to some extent.
3. Yes, I need help in every respect.

C211. Do you need someone to help you with laundry, such as using a washing machine or by hand and hanging out the wash to dry?

[IWER: If R can do small laundry such as socks but cannot do heavy laundry or if R can do laundry but is unable to hang out the wash, then mark ③]

1. No, I don't need any help.

③ Yes, I need help to some extent.

⑤ Yes, I need help in every respect.

C212. Do you need someone to help you with going out a short distance without transportation?

[IWER: Mark ① if R uses assistance devices, such as a cane. Enter ③ if R needs to ride a wheelchair.]

1. No, I don't need any help.

③ Yes, I need help to some extent.

⑤ Yes, I need help in every respect.

C213. Do you need someone to help you with going out using transportation, such as buses, subways, taxies, and cars?

1. No, I don't need any help.

③ Yes, I need help to some extent.

⑤ Yes, I need help in every respect.

C214. Do you need someone to help you with shopping, which refers to make a decision on what to buy and to pay money and get the change?

1. No, I don't need any help.

③ Yes, I need help to some extent.

⑤ Yes, I need help in every respect.

C215. Do you need someone to help you with managing money, such as managing small sums of pocket money, savings accounts and other assets?

[IWER: Mark ③ if large sums of money is managed by someone else.]

1. No, I don't need any help.

③ Yes, I need help to some extent.

⑤ Yes, I need help in every respect.

C216. Do you need someone to help you with making or taking phone calls?

[IWER: Mark ③ if R can only dial the numbers she/he knows.]

1. No, I don't need any help.

③ Yes, I need help to some extent.

⑤ Yes, I need help in every respect.

C217. Do you need someone to help you with taking medications, which refers to take right portion as well as right on time?

1. No, I don't need any help.

③ Yes, I need help to some extent.

⑤ Yes, I need help in every respect.

**<ADL/IADL HELPER>**

**Logic**

- **If R selected any answer other than ① in any of C201~C217, then ask C218.**
- **If R answered ① in all of C201~C217, then ask C224.**

**Logic**

- **Loop through C218~C222 up to five times. If C218=56, then ask C223. (However, if C218=56 in the first loop, then ask C224).**

C218. If you need help with activities of daily living, name the five people who help you the most in order. Who is the person that comes [1st/2nd/..] in helping you? Please state the person's name or relationship with you.

1. Spouse
2. Mother
3. Father
4. Mother-in-law
5. Father-in-law

[7~26] Children list

[27~46] Sibling list

[47] Brother-in-law/sister-in-law

[48] Son-in-law/daughter-in-law

[49] Grandchildren

[50] Other relatives

[52] Voluntary worker

[53] Publicly provided helper

[54] Personally hired helper

[55] Others

[56] No one helped →Go to C223 (In the first loop, C224)

C219. How many days did [name selected in C218] help you during the past one month? (unit: a day)

_______________________________ days [Range: 1~31]

C220. How many hours per day does [name selected in C218] usually help you? (unit: an hour)

[IWER: less than an hour=1]

_______________________________ hours [Range: 1~24]

C221. Is [name selected in C218] paid to help you?

1. Yes ⑤ No →Go to C224

C222. How much did you pay [name selected in C218] in the past one month? (unit: 10,000 Korean won)

_______________________________MW [Range: 1~997]

Don’t know →Go to C218

Refuse to answer →Go to C218

**Logic**

- **If there is a loop total of C222, then ask C223.**

C223. You have said that you paid [C222] MW (10,000 Korean won) to your helpers in the past one month. How much did you pay out-of-pocket? Also, how much did your relatives or private health insurances pay on behalf of you? Please include in the private health insurance category any payments that you made and were reimbursed by private health insurances. (unit: 10,000 Korean won) Categories are ① out-of-pocket payment (C223a), ② payment by relatives including children and parents (C223b), ③ payment reimbursed by private health insurances (C223c).

[IWER: The sum of all items must be equal to [C222] MW.]

| ITEMS | | AMOUNT |
| --- | --- | --- |
| C223a | Out-of-pocket payments |  |
| C223b | Payments by relatives including children and parents |  |
| C223c | Payments reimbursed by private health insurances |  |
| Total | | [C222] MW |

C224. Who is likely to help you if you need help with activities of daily living, such as eating, dressing, or using the bathroom, over a long period of time in the future? Choose all that apply.

1. Spouse
2. Parents
3. Son
4. Daughter
5. Son-in-law/daughter-in-law
6. Sibling
7. Grandchildren
8. Others
9. Voluntary worker
10. Publicly provided helper
11. Personally hired helper
12. No helper

C225. [IWER: How often did R receive assistance in answering section C2–FUNCTIONAL LIMITATIONS AND HELPERS?]

1. Never →Go to section C3.
2. A few times →Go to section C3.
3. Most or all of the time →Go to section C3.
4. The section was done by a proxy respondent.

C226. If done by a proxy respondent, what is the proxy’s relationship to R? Please answer in view of the proxy

1. Spouse
2. Mother
3. Father
4. Mother-in-law
5. Father-in-law
6. Brother/sister
7. Brother-in-law/sister-in-law
8. Son/daughter
9. Son-in-law/Daughter-in-law
10. Grandchild
11. Other relatives
12. Helper or other non-relatives

→ Go to section C3.

# **C3. HEALTH INSURANCE AND SERVICES**

**<Health insurance>**

C301. The next questions are about health insurance. In Korea, there are two types of insurances; National Health insurance and Medical Aid insurance. Under National Health Insurance, there are two types of employee insured and self-employed insured. Under Medical Aid Program, there are class 1 and class 2 defined by the awardees of the National Merit Award and other honorary people as well as people with low incomes.

Are you currently covered by the National Health Insurance (employee insured or self-employed insured) or the Medical Aid Program (class 1 and class 2)?

1. National Health Insurance (employee insured/ self-employed insured)
2. Medical Aid Program (class 1/ class 2) →Go to C309

Don’t know →Go to Cadd_24

Refuse to answer →Go to Cadd_24

C302. Then, as for National Health Insurance, which of two are you belonging to?

1. Employee insured of R’s or R’s family
2. Self-employed insured →Go to C304

C303. If employee insured health insurance, are you the insured person or a dependent family member?

1. Insured person →Go to C305

⑤ Dependent family member →Go to C305

C304. If self-employed insured, are you the household head?

1. Yes ⑤ No

C305. Who is currently paying your health insurance premiums?

1. You
2. Spouse
3. Son/daughter
4. Son-in-law/daughter‐in‐law
5. Other relatives

C306. What is the average monthly amount of insurance premiums paid into the National Health Insurance? (unit: 10,000 Korean Won)

[IWER: less than ten thousands won = “ten thousands won”]

_______________________________ MW [Range: 1~120]

C307. Are you or any household member paying the National Health Insurance premium for which you are the insured currently in arrears?

1. Yes ⑤ No →Go to Cadd_23

Don’t know →Go to Cadd_23

Refuse to answer →Go to Cadd_23

C308. How many months have you or any household member who pays the National Health Insurance premium for which you are the insured in arrears? (unit : month)

_______________________________ Months [Range: 1~203] → Go to Cadd_23

Don’t know →Go to Cadd_23

Refuse to answer →Go to Cadd_23

Cadd_23. In the past year, have you ever been suspended for insurance coverage due to non-payment of health insurance premiums for more than 3 months in a row?

1. Yes → Cadd_24 ⑤ No → Cadd_24

C309. Then, as for Medical Aid Program, which of two are you belonging to?

1. Class 1 ⑤ Class 2

Cadd_24. In the past year, have you or your family been unable to go to the hospital because of financial difficulties?

1. Yes ⑤ No

C310. Are you currently covered by any private health insurance to compensate for hospital expenses? Private health insurance means every health insurance product regardless of its type, sold by insurance company, National Agricultural Cooperative Federation or post office, etc. even including those whose term expired. Please exclude health-related special policy conditions included in pension insurance or whole life insurance.

1. Yes ⑤ No →Go to C313

C311. How many private health insurances do you have? Please tell me only those with you as a beneficiary.

_______________________________ [Range: 1~30]

C312. What is the total amount of insurance premiums you pay each month for these private health insurances? (unit: 10,000 Korean Won)

_______________________________ MW [Range: 0~997]

C313. In the past two years, have you ever received basic medical check-up service provided by the National Health Insurance or the Medical Aid Program for free?

1. Yes →Go to C316 ⑤ No

C314. If not, why did you not receive medical checkup?

1. Wanted to receive it, but unable because I can’t move freely →Go to C317
2. Did not expect any problems because I was fine in the previous checkup →Go to C317
3. Too busy, could not have time →Go to C317
4. I could not trust the results →Go to C317
5. I was afraid of the results →Go to C317
6. Even if an illness is detected, no adequate treatment is available →Go to C317
7. Did not see a need of checkup →Go to C317
8. I did not know it is free →Go to C317
9. Others

C315. If others, please specify.

_______________________________

C316. Have you ever received any further medical checkup due to problems found in basic medical checkup?

1. Yes ⑤ No

C317. Have you had any other medical checkup using your out‐of‐pocket money in the past two years?

1. Yes ⑤ No

**< Long-Term Care Insurance >**

C318. Are you aware of ‘Long-Term Care Insurance’?

[IWER: Long-Term Care Insurance is a social insurance system that provides long term care benefits for movement support or household activities support to the elderly who can not hold a regular living due to old age or geriatric disease, thus improving old age health and stable living, decreasing the burden of family and making quality of life higher.]

1. Yes ⑤ No →Go to C329

C319. Have you applied for ‘Long-Term Care Insurance’?

1. Yes ⑤ No →Go to C329

C320. Are you currently using ‘Long-Term Care Insurance’ service?

1. Yes, I am currently using it →Go to C323 ⑤ No, I am not using it

C321. Why aren’t you using the service?

1. Because I am not qualified →Go to C329
2. Because I am not satisfied with the conditions for service receipt or the service quality →Go to C329
3. Others

C322. If others, please specify.

_______________________________→Go to 329

**Logic**

- **If R is aged under 65 years old, mark ① automatically**

C323. Why are you currently using ‘Long-Term Care Insurance’ service?

1. Because of geriatric disease
2. Because of mobility impaired →Go to C325
3. Both geriatric disease and mobility difficulty

C324. You said that you are using ‘Long-Term Care Insurance’ service because of geriatric disease, could you please specify?

_______________________________

C325. What is your grade of ‘Long-Term Care Insurance’ assessed by the National Health Insurance Service?

1. Grade 1
2. Grade 2
3. Grade 3
4. Grade 4
5. Grade 5
6. Cognitive support grade

C326. Then, your monthly average benefit for ‘Long-Term Care Insurance’ will be _____________.

1. Check

C327. Which service of ‘Long-Term Care Insurance’ are you currently using?

[IWER:

1. Institutional care service: This refers to long-term benefit that supports physical activity and provides education or training to maintain or improve the mental & physical functioning of the elderly who are residing in aged care facilities or senior congregate housings (except senior specialized hospitals)
2. Home care service: This refers to home-visit care that a long-term care staff visit a beneficiary’s home and helps in the following: bathing, excretion, going to the bathroom, changing clothes, washing hair, cooking, buying daily necessities, cleaning and clearing up. This also includes weekly care service that provides physiotherapy, language therapy, mental & physical functioning improvement, food service, bathing service, education or consulting service for elderly family, etc.
3. Special cash benefit: This refers to cash benefits, not gifts or goods, such as family care cash benefits, exceptional cash benefits and nursing expense of long-term care hospitals. Currently only family care benefits are provided to family members of those who live in the area with limited aged care facilities (e.g. island area, mountain village) or those who cannot use aged care facility due to natural disaster.]
4. Institutional care service
5. Home care service
6. Special cash benefit

C328. During the last one year, how much is the monthly average you paid for exceeding the service benefit? Please only consider when you exceed the monthly service benefit.

_______________________________MW →Go to C336

**< Care Service for the Elderly >**

**Logic**

- **If C318=⑤, C319=⑤, C320=⑤, then ask C329.**

C329. Are you aware of ‘Care Service for the Elderly’?

[IWER: Care Service for the Elderly is a senior welfare system for 65 years old that provides customized welfare services such as safety check, daily life education, service connection, household activity support and weekly protection, etc. to elderly who can not hold a regular living by himself/ herself.]

1. Yes ⑤ No →Go to C336

C330. Have you applied for ‘Care Service for the Elderly’?

1. Yes ⑤ No →Go to C336

C331. Are you currently using ‘Care Service for the Elderly’?

1. Yes, I am currently using it →Go to C334 ⑤ No, I am not using it

C332. Why aren’t you using the service?

1. Because I am not qualified →Go to C336
2. Because I am not satisfied with the conditions for service receipt or the service quality →Go to C336
3. Others

C333. If others, please specify.

_______________________________→Go to 336

C334. Which service of ‘Care Service for the Elderly’ are you currently using?

[IWER:

1. Basic Service: This refers to a protection service for those aged over 65 years old or disabled by providing regular safety check, daily life education, service connection via home-visit (4 hours/ per month) or calling
2. Care Service for the Elderly (e-voucher): This refers to a service for those who still require long-term care, except elegies who are already qualified for ‘long-term insurance’ service. This service provides household activities support service or weekly protection service to elderly who can not hold a regular living thus improving old age health and stable living]
3. Basic Service
4. Care Service for the Elderly (e-voucher)

C335. Which service of Care Service for the Elderly (e-voucher) are you currently using?

[IWER:

1. Short-term housekeeping service: 24 hours/ per month service prioritized for over 65 years old seniors who live alone or for those who are suffering with fracture illness or surgery experience for serious illness among elderly over 75 years old (both husband and wife)
2. Housekeeping activity support service (In-home visit): 27 hours/ per month or 36 hours/ per month service that provides daily household work such as safety check, cooking, cleaning and laundry.
3. Day care service: 27 hours/ per month (9 days), 36 hours/ per month (12 days) service that provides physiotherapy, language therapy, mental & physical functioning improvement, food service, bathing service, education or consulting service for elderly family.]
4. Dementia family vacation support service (short-term cafe service) : Protection of the elderly with dementia at the service provider for a certain period of time (6 days a year)
5. Short-term housekeeping service
6. Housekeeping activity support service (In-home visit)
7. Day care service
8. Dementia family vacation support service (short-term care service)

**<Hospitalization>**

**Logic**

- **If C336=0, then ask C348.**

C336. The following questions are about the health care services you have received since the previous interview (___ month ___ day ___ year). Were you ever hospitalized at a hospital, nursing hospital, convalescence clinic, or any other long-term health care facilities since the previous interview? If so, how many times were you hospitalized? Please exclude staying for living purpose. (unit: a time)

[IWER: If R never be hospitalized, mark ‘0’]

_______________________________ time(s) [Range: 0~52]

**Logic**

- **Loop through the following questions as many times as the number entered in C336.**

C337**.** In which health care facility were you hospitalized during your [1st/2nd/..] hospitalization?

1. General hospital

③ Government-designated nursing hospital

⑤ Other nursing hospitals

C338. How many days did you stay at [C337]? (unit: a day)

[IWER: For example, enter 180 days for 6 months, and 450 days for 1 year and 3 months.]

_______________________________ days [Range: 1~2190]

C339.You have said that you were hospitalized for [C338] days. At that time, how much did you pay for your hospitalization (medical treatment) in total? (unit: 10,000 Korean won)

_______________________________MW [Range: 1~9997]

Don’t know →Go to C341

Refuse to answer →Go to C341

**Logic**

- **(Only ask for the last loop) Ask C340 for the total amount for hospitalization at C339.**

C340. You have said that you paid [C339] MW (10,000 Korean won) for your [C336] rounds of hospitalization (medical treatment). How much did you pay out-of-pocket? Also, how much did your relatives or private health insurances pay on behalf of you? Please include in the private health insurance category any payments that you made and were reimbursed by private health insurances. (unit: 10,000 Korean won) Categories are ① out-of-pocket payment (C340a), ② payment by relatives including children and parents (C340b), ③ payment reimbursed by private health insurances (C340c).

[IWER: The sum of all items must be equal to [C339] MW.]

| ITEMS | | AMOUNT |
| --- | --- | --- |
| C340a | Out-of-pocket payments |  |
| C340b | Payments by relatives including children and parents |  |
| C340c | Payments reimbursed by private health insurances |  |
| Total | | [C339] MW |

**Logic**

- **If C341=(57), then ask C337.**

C341. Did anybody take care of you during your [1st/2nd/..] stay at the health care facility? If so, among the following people who took care of you? Please do not include those who simply visited. (Select multiple responses.)

1. Spouse →Go to C343
2. Mother →Go to C343
3. Father →Go to C343
4. Mother-in-law →Go to C343
5. Father-in-law →Go to C343

[7~26] Children list →Go to C343

[27~46] Sibling list →Go to C343

[47] Brother-in-law/sister-in-law →Go to C343

[48] Son-in-law/daughter-in-law →Go to C343

[49] Grandchildren →Go to C343

[50] Other relatives →Go to C343

[52] Voluntary worker →Go to C343

[53] Publicly provided helper →Go to C343

[54] Personally hired helper →Go to C343

[55] Others

[57] No one helped →Go to C337

C342. If others, please specify.

_______________________________

**Logic**

- **Loop C343~C346 as many times as the number of people chosen in C341.**

C343. How many days did the caregiver chosen in [C341] take care of you?

[IWER: For example, enter 180 days for 6 months, and 450 days for 1 year and 3 months.]

_______________________________ days [Range: 1~2190]

C344. How many hours per day did the caregiver chosen in [C341] take care of you? (unit: a time)

_______________________________ hours [Range: 1~24]

C345. Did you pay [C341] for taking care of you?

1. Yes ⑤ No →Go to C343

C346. How much did you pay [C341] for taking care of you in total? (unit: 10,000 Korean won)

_______________________________ MW [Range: 1~997]

Don’t know →Go to C343

Refuse to answer →Go to C343

**Logic**

- **(Only ask for the last loop) Ask C347 for the total amount at C346.**

C347. You have said that you paid [C346] MW (10,000 Korean won) for the caregiver services. How much did you pay out-of-pocket? Also, how much did your relatives or private health insurances pay on behalf of you? Please include in the private health insurance category any payments that you made and were reimbursed by private health insurances. (unit: 10,000 Korean won) Categories are ① out-of-pocket payments (C347a), ② payments by relatives including children and parents (C347b), ③ payments reimbursed by private health insurances (C348c).

[IWER: The sum of all items must be equal to [C346] MW]

| ITEMS | | AMOUNT |
| --- | --- | --- |
| C347a | Out-of-pocket payments |  |
| C347b | Payments by relatives including children and parents |  |
| C347c | Payments reimbursed by private health insurances |  |
| Total | | [C346] MW |

**<Dental Treatment>**

**Logic**

- **If C348=0, then ask C351.**

C348. Have you ever received dental care (including denture care) since the previous interview (___ month ___ day ___ year)? If so, how many times did you go to a dental hospital/clinic? (unit: a time)

[IWER: Enter 0 if R has never made such a visit.]

_______________________________ time [Range: 0~2190]

C349. You have said that you received dental care [C330] times since the previous interview (___ month ___ day ___ year). If so, how much did you pay for your dental care in total? (unit: 10,000 Korean won)

_______________________________MW [Range: 1~9997]

Don’t know →Go to C351

Refuse to answer →Go to C351

C350. You have said that you paid [C349] MW (10,000 Korean won) for your dental care. How much did you pay out-of-pocket? Also, how much did your relatives or private health insurances pay on behalf of you? Please include in the private health insurance category any payments that you made and were reimbursed by private health insurances. (unit: 10,000 Korean won) Categories are ① out-of-pocket payments (C350a), ② payments by relatives including children and parents (C350b), ③ payments reimbursed by private health insurances (C350c).

[IWER: The sum of all items must be equal to [C349] MW.]

| ITEMS | | AMOUNT |
| --- | --- | --- |
| C350a | Out-of-pocket payments |  |
| C350b | Payments by relatives including children and parents |  |
| C350c | Payments reimbursed by private health insurances |  |
| Total | | [C349] MW |

**<Public Health Clinic>**

C351. Have you ever visited a public health clinic since the previous interview (___ month ___ day ___ year)? If so, how many times did you go to a public health clinic? (unit: a time)

[IWER: Enter 0 if R has never made such a visit.]

_______________________________ time [Range: 0~2190]

**<Oriental Medicine Clinic>**

**Logic**

- **If C352=0, then ask C355.**

C352. Have you ever visited an oriental medicine clinic since the previous interview? If so, how many times did you go to an oriental medicine clinic? (unit: a time)

[IWER: Enter 0 if R has never made such a visit.]

_______________________________ time [Range: 0~2190]

C353. You have said that you went to an oriental medicine clinic [C334] times since the previous interview. If so, how much did you pay for your medical care including the purchase of oriental medicine in total? (unit: 10,000 Korean won)

_______________________________ MW [Range: 1~9997]

Don’t know →Go to C355

Refuse to answer →Go to C355

C354. You have said that you paid [C353] MW (10,000 Korean won) for care at the oriental medicine clinic including the purchase of oriental medicine. How much did you pay out-of-pocket? Also, how much did your relatives or private health insurances pay on behalf of you? Please include in the private health insurance category any payments that you made and were reimbursed by private health insurances. (unit: 10,000 Korean won) Categories are ① out-of-pocket payments (C354a), ② payments by relatives including children and parents (C354b), ③ payments reimbursed by private health insurances (C354c).

[IWER: The sum of all items must be equal to [C353] MW.]

| ITEMS | | AMOUNT |
| --- | --- | --- |
| C354a | Out-of-pocket payments |  |
| C354b | Payments by relatives including children and parents |  |
| C354c | Payments reimbursed by private health insurances |  |
| Total | | [C353] MW |

**< Other Outpatient Medical Treatment >**

**Logic**

- **If C355=0, then ask C358.**

C355. Aside from any visits to oriental medicine clinic, public health clinics, dental clinics and hospital stays, how many times have you seen or talked to a medical doctor about your health, including internal, ENT (ear-nose-and throat), surgery or hospital as an outpatient or to an emergency room since the previous interview (___ month ___ day ___ year)? If so, how many times did you go there? (unit: a time)

[IWER: Enter 0, if R has never made such a visit.]

_______________________________ time [Range: 0~2190]

C356. You have said that you went to a local clinic or hospital [C337] times since the previous interview (___ month ___ day ___ year). If so, how much did you pay for your outpatient care in total? (unit: 10,000 Korean won)

_______________________________ MW [Range: 1~9997]

Don’t know →Go to C358

Refuse to answer →Go to C358

C357. You have said that you paid [C356] MW (10,000 Korean won) for your outpatient care. How much did you pay out-of-pocket? Also, how much did your relatives or private health insurances pay on behalf of you? Please include in the private health insurance category any payments that you made and were reimbursed by private health insurances. (unit: 10,000 Korean won) Categories are ① out-of-pocket payments (C357a), ② payments by relatives including children and parents (C357b), ③ payments reimbursed by private health insurances (C357c).

[IWER: The sum of all items must be equal to [C356] MW.]

| ITEMS | | AMOUNT |
| --- | --- | --- |
| C357a | Out-of-pocket payments |  |
| C357b | Payments by relatives including children and parents |  |
| C357c | Payments reimbursed by private health insurances |  |
| Total | | [C356] MW |

**< In-home health care >**

**Logic**

- **If C358=0, then ask C361.**

C358. Has any medically-trained person come to your home to help you, yourself since the previous interview (___ month ___ day ___ year)? If so, how many times did they visit you? (unit: a time)

[IWER: Medically-trained person refers to nurses, nurse assistants, physical therapists, chemical therapists and oxygen therapists, who professionally trained for medical treatment purpose. Caregivers who just take care of patients are excluded. If R never received, then enter 0.]

_______________________________ time [Range: 0~2190]

C359. You have said that you received [C358] in-home health care since the previous interview (___ month ___ day ___ year). How much did you pay for your in-home health care in total? (unit: 10,000 Korean won)

_______________________________ MW [Range: 1~9997]

Don’t know →Go to C361

Refuse to answer →Go to C361

C360. You have said that you paid [C359] MW (10,000 Korean won) for your in-home health care. How much did you pay out-of-pocket? Also, how much did your relatives or private health insurances pay on behalf of you? Please include in the private health insurance category any payments that you made and were reimbursed by private health insurances. (unit: 10,000 Korean won) Categories are ① out-of-pocket payments (C360a), ② payments by relatives including children and parents (C360b), ③ payments reimbursed by private health insurances (C360c).

[IWER: The sum of all items must be equal to [C359] MW.]

| ITEMS | | AMOUNT |
| --- | --- | --- |
| C360a | Out-of-pocket payments |  |
| C360b | Payments by relatives including children and parents |  |
| C360c | Payments reimbursed by private health insurances |  |
| Total | | [C359] MW |

**<Prescription Drug>**

C361. Have you ever taken regular prescription drugs since the previous interview (___ month ___ day ___ year)? It refers to the medications for long-terms prescription such as antidiabetic or hypertension medicine

1. Yes ⑤ No →Go to C364

C362. How much did you pay for your regular prescription drugs since the previous interview (___ month ___ day ___ year) in total? (unit: 10,000 Korean won)

_______________________________ MW [Range: 1~9997]

Don’t know →Go to C364

Refuse to answer →Go to C364

C363. You have said that you paid [C362] MW (10,000 Korean won) for your prescription drugs. How much did you pay out-of-pocket? Also, how much did your relatives or private health insurances pay on behalf of you? Please include in the private health insurance category any payments that you made and were reimbursed by private health insurances. (unit: 10,000 Korean won) Categories are ① out-of-pocket payments (C363a), ② payments by relatives including children and parents (C363b), ③ payments reimbursed by private health insurances (C363c).

[IWER: The sum of all items must be equal to [C362] MW]

| ITEMS | | AMOUNT |
| --- | --- | --- |
| C363a | Out-of-pocket payments |  |
| C363b | Payments by relatives including children and parents |  |
| C363c | Payments reimbursed by private health insurances |  |
| Total | | [C362] MW |

**<Other Medical Assistance Equipment>**

C364. Have you ever purchased (leased) any medical assistance equipment since the previous interview? For example, it refers to medical assistance equipment for treatment purposes, such as hearing aids or wheelchairs.

1. Yes ⑤ No →Go to C367

C365.How much did you pay for purchasing (leasing) the medical assistance equipment since the previous interview in total? (unit: 10,000 Korean won)

_______________________________ MW [Range: 1~9997]

Don’t know →Go to C367

Refuse to answer →Go to C367

C366. You have said that you paid [C365] MW (10,000 Korean won) for purchasing (leasing) the medical assistance equipment. How much did you pay out-of-pocket? Also, how much did your relatives or private health insurances pay on behalf of you? Please include in the private health insurance category any payments that you made and were reimbursed by private health insurances. (unit: 10,000 Korean won) Categories are ① out-of-pocket payments (C366a), ② payments by relatives including children and parents (C366b), ③ payments reimbursed by private health insurances (C366c).

[IWER: The sum of all items must be equal to [C365] MW]

| ITEMS | | AMOUNT |
| --- | --- | --- |
| C366a | Out-of-pocket payments |  |
| C366b | Payments by relatives including children and parents |  |
| C366c | Payments reimbursed by private health insurances |  |
| Total | | [C365] MW |

C367. [IWER: How often did R receive assistance in answering section C3–HEALTH INSURANCE AND SERVICES?]

1. Never →Go to section C4.
2. A few times →Go to section C4.
3. Most or all of the time →Go to section C4.
4. The section was done by a proxy respondent.

C368. If done by a proxy respondent, what is the proxy’s relationship to R? Please answer in view of the proxy

1. Spouse
2. Mother
3. Father
4. Mother-in-law
5. Father-in-law
6. Brother/sister
7. Brother-in-law/sister-in-law
8. Son/daughter
9. Son-in-law/Daughter-in-law
10. Grandchild
11. Other relatives
12. Helper or other non-relatives

→Go to section C4.

# **C4. COGNITION**

C400. Before we go on to the next section, I would like to check if you are a proxy respondent. Are you [R’s name]?

1. Yes ⑤ No → Go to C421

C401. Now I’m going to ask several simple questions. Some may be easy and others may be hard to answer. Even if it is too easy, please don’t neglect it. In addition, even if it is too difficult, please don’t be disappointed. You may just answer for the one you know. Are you ready?

Starting with the date, what date is it today? Please tell me date, month and year.

[IWER: R doesn't have to answer year, month and date in an order. If R is an elderly person and says the date according to lunar calendar and it matches with the solar calendar, that date is considered correct. Check the accuracy, using the converter.]

1. One of the month / day / year combination is correct
2. Two of the month / day / year combination are correct
3. All of the month / day / year combination are correct

⑤ All of the month / day / year combination are incorrect

C402. What day of the week is it today? Is it Monday, Tuesday, Wednesday, Thursday, Friday, Saturday, or Sunday?

1. Day - Correct ⑤ Day - Incorrect

C403. What is the current season (among Spring, Summer, Fall, or Winter)?

1. Season - Correct ⑤ Season - Incorrect

C404. Please tell me about the place where you and I are now. What is this place used for?

[IWER: If R gives a specific description of the place, such as my home, family house, general house, senior citizens’ center, hospital, a place to live, a place to treat sick people, oo apartment, oo village, his/her answer is considered correct.]

1. Place - Correct ⑤ Place - Incorrect

C405. What is your address? Please tell me the city, Gun, Dong, APT #/Street Number.

[IWER: It is possible to repeat the question if R has missed one of the four components. It is also fine to ask a question with the name of the present location (e.g. Is here Busan/Gyeongsang-do?).]

1. One of the city / gun / dong / street address combination is correct
2. Two of the city / gun / dong / street address combination are correct
3. Three of the city / gun / dong / street address combination are correct
4. All of the city / gun / dong / street address combination are correct
5. All of the city / gun / dong / street address combination are incorrect

**Logic**

- **Lists are shown randomly.**
- LIST 1: airplane, pencil, and pine tree
- LIST 2: snowman, chair, and peach
- LIST 3: teacher, factory, and washing machine
- LIST 4: chopsticks, apple, and hanger
- LIST 5: bat, shoes, and sea water

C406. Please listen carefully. Now, I will read out three words just only one time and ask you to recall as many as you can. I cannot repeat them. When I finish, I will ask you to recall aloud as many of the words as you can, in any order. Are you ready?

[IWER: ① Tidy up the surroundings and start. ② Assigned words are displayed only to the IWER.

③ Read the words aloud clearly in steady pace and pause one second before the next word ④ If R is unable to recall any words or can recall only a few words, repeat reading out up to 5 times including the first try until he/she can recall all of them. ⑤ If R can't recall the words even after 5 times, please stop the process. ⑥ Answers given by repetition should be treated as incorrect answers]

1. Only one of the three words is correct, in any order
2. Two of the three words are correct, in any order
3. All of the three words are correct, in any order
4. None of the three words is correct

C407. Please try to remember the words I just read out to you. I'll ask you to recall them later. [Read once more if R did not recall any of the words, up to 3 times and go on. If R does not recall, reassure him/her that it is OK to make him/her feel comfortable.]

Now let's try some subtraction of numbers. One hundred minus 7 equals what?

[IWER: If R is unable to solve complex calculation, mark ‘0(incorrect answer)’ for all of C407 ~ C411. Instead, ask simple questions such as “What is 100 minus 30?” or “What is 10 minus 3?” lasting for about 3 minutes. However, please do not take longer than 3 minutes]

_______________________________ (Mark the number what the R said)

C408. Then, if you subtract 7 from that, what is it?

_______________________________ (Mark the number what the R said)

C409. Then, if you subtract 7 from that, what is it?

_______________________________ (Mark the number what the R said)

C410. Then, if you subtract 7 from that, what is it?

_______________________________ (Mark the number what the R said)

C411. Lastly, if you subtract 7 from that again, what is it?

_______________________________ (Mark the number what the R said)

**Logic**

- **The words shown in C406 are displayed again only for the IWER. They must be the same words as before.**

C412. A little while ago, I read out the three words to you and you repeated the ones you could remember. Please tell me any of the words that you remember now.

1. Only one of the three words is correct, in any order
2. Two of the three words are correct, in any order
3. All of the three words are correct, in any order
4. None of the three words is correct

C413. [IWER: Pointing to item #1] Now I'm going to ask you for the names of things. What is this?

[IWER: An item can be anything near that anybody can distinguish such as such as a cell phone, gloves, a hat and a ring. The elderly tend to call all types of writing instruments 'pencil', so consider the answer correct if they say ‘pencil’ even for ‘pen’.]

1. Correct ⑤ Incorrect

C414. [IWER: Pointing to item #2] What is this?

[IWER: If R calls a watch “clock”, the answer is considered correct.]

1. Correct ⑤ Incorrect

**Logic**

- **Lists are shown randomly.**
- Sentence 1: To see is to believe
- Sentence 2: Check the dead fire again
- Sentence 3: The Great Mountain is high but under the sky
- Sentence 4: God watch over our land forever
- Sentence 5: Thousand miles of gorgeous river and mountain

C415. Now you have to exactly repeat what I read out. Are you ready? Please listen carefully.

[IWER: ① Tidy up the surroundings. ② Assigned words are displayed only to the IWER.

③ Read the sentences aloud clearly in steady pace ④ Mark 'Incorrect' if the pronunciation is unclear, except when R has no teeth or problems in oral structure. In principle, answers given by repetition should be treated as incorrect answers. However, if R is deemed to be distracted on the first time, IWER may give second trial after a shore refresh time. Then, if R gets the right answer, mark 'Correct']

1. Correct ⑤ Incorrect

C416. [IWER: ① Tidy up the surroundings. ② As holding one A4 paper, ask the question. Please do not show any motions of folding or turning over the paper] Now, listen carefully and follow my directions. Are you ready? When I give you a piece of paper, please turn it over, fold it in half, and give it back to me.

[IWER: ① Tidy up the surroundings (especially the front) so it doesn't interfere with R. ② Do not repeat the question in the middle of the process. ③ Do not give out the paper in advance. ④ Directions can be repeated if R seems unable to understand the directions or if R asks you to repeat the directions. In this case, the paper given out should be collected and the process should start over.]

1. One of the turning / folding / returning actions is completed successfully
2. Two of the turning / folding / returning actions are completed successfully
3. All of the turning / folding / returning actions are completed successfully
4. None of the turning / folding / returning actions is completed successfully

C417. [IWER: Make sure R doesn't see the test paper with “close your eyes” written on it before asking the question. Make sure that someone does not read the sentence out to R.] I will show you a sentence. Please read the sentence aloud first and act it out as written.

1. R completed only one task (either reading or action of closing eyes)
2. R did both tasks

⑤ R did not complete any task

C418. [IWER: Give R a pen and point to the blank part of the paper] Please write one sentence about how you’re feeling today or about today’s weather.

1. Wrote a sentence

⑤ Couldn’t write a sentence

C419. [IWER: Show the picture of two pentagons overlapped] Do you see this picture? Please draw that picture on this paper as shown.

1. Drew the picture

⑤ Failed to draw the picture

C420. [IWER: How often did R receive assistance in answering section C4–COGNITION?]

1. Never →Go to section C5.
2. A few times →Go to section C5.
3. Most or all of the time →Go to section C5.

C421. [IWER: Section C4–COGNITION cannot be done by a proxy respondent.]

1. CONTINUE →Go to section C5.

# **C5. GRIP STRENGTH**

C500. Before we go on to the next section, I would like to check if you are a proxy respondent. Are you [R’s name]?

1. Yes ⑤ No →Go to C514

C501. Now I would like to measure the strength of your hand in a gripping action. Which is your dominant hand?

1. Right-handed

③ Left-handed

⑤ Both hands equally dominant

C502. I will ask you to squeeze this handle as hard as you can, just for a couple of seconds and then let go. I will take alternately two measurements from your right and left hands. If you have any uncomfortable hand, you can measure only the other. Are you willing to do this test?

1. Yes

③ Willing but unable to do the test →Go to C505

⑤ No →Go to C505

C503. Before we begin, I’d like to make sure it is safe for you to do this test. Within the last six months, in any of your hands, have you had any surgery or experienced any swelling, inflammation, severe pain, or injury that might prevent you from gripping?

1. No apparent restriction →Go to C507
2. Yes, recent surgery
3. Yes, sever pain
4. Yes, swelling or inflammation
5. Other problems

C504. Which hand hurts?

1. R is unable to use both hands →Go to C505
2. R is unable to use right hand →Go to C509
3. R is unable to use left hand →Go to C507

C505. [IWER: Why didn’t R complete the grip strength test?] (Select all that apply.)

1. R felt it would not be safe →Go to C513
2. IWER felt it would not be safe →Go to C513
3. R refused →Go to C513
4. R tried but was unable to complete the test →Go to C513
5. R did not understand the instructions →Go to C513
6. R had surgery, injury or swelling on both hands in the past six months →Go to C513
7. Others

C506. If others, please specify.

_______________________________ →Go to C513

C507. Now, I’m going to measure your RIGHT HAND. Please put your elbow onto your side and make a 90-degree elbow. Make sure the scale of the dynamometer is set to zero. Ready? Squeeze it now as hard as you can. (unit: Kg)

[IWER: Please ask R to take off his/her ring or watch for safety.]

_______________________________ First Trial [Range: 0~50]

C508. Now, I’m going to measure your RIGHT HAND again. Please set the scale of the dynamometer to zero, Squeeze it now. (unit: Kg.)

_______________________________ Second Trial [Range: 0~50]

**Logic**

- **If C504=⑤, then ask C511.**

C509. Now, I’m going to measure your LEFT HAND, Please put your elbow onto your side and make a 90-degree elbow. Make sure the scale of the dynamometer is set to zero. Ready? Squeeze it now as hard as you can. (unit: Kg)

[IWER: Please ask R to take off his/her ring or watch for safety.]

_______________________________ First Trial [Range: 0~50]

C510. Now, I’m going to measure your LEFT HAND again. Please set the scale of the dynamometer to zero. Squeeze it now. (unit: Kg.)

_______________________________ Second Trial [Range: 0~50]

C510_Check. [IWER] Please confirm that the measurement was done in unit (kg) and started with scale zero (0).

1. Check

C511. [IWER: What was R’s position for this test?]

1. Standing
2. Sitting
3. Lying down

C512. [IWER: Did R receive a support while performing this test?]

1. Yes ⑤ No

C513. [IWER: How much effort did R give to this measurement?]

1. R gave full effort →Go to section D.
2. R was prevented from giving full effort by illness, pain, symptoms or discomfort →Go to section D.
3. R did not appear to give full effort without an obvious reason →Go to section D.
4. R did not measure for a personal reason →Go to section D.

C514. [IWER: Section C5–GRIP STRENGTH cannot be done by a proxy respondent.]

1. CONTINUE

(C_end) [IWER] Please enter code 1 for completion of C. HEALTH section.

1. Completion

# **D. EMPLOYMENT**

(D_Intro) [IWER] Now, this is to start D. EMPLOYMENT section.

1. Check

**<Current Employment Status>**

**Logic**

- **R cannot answer ‘Don’t know’ or’ Refuse to answer’ to D001.**

D001. Are you currently employed for income? Being employed refers to working for an employer, being self‐employed, or working for your family or relative’s business.

1. Yes ⑤ No →Go to D005

**Logic**

- **R cannot answer ‘Don’t know’ or’ Refuse to answer’ to D002.**

D002. I would like to ask about your current main job. Which of the following best describes your main job?

1. I am employed by another person or a company and receive wages. →Go to D007 or D008
2. I am self-employed. →Go to D007 or D008
3. I help my family / relative’s business without pay for 18 hours or more per week. →Go to D007 or D008
4. I help my family / relative’s business without pay for less than 18 hours a week.

D003. What is the reason you work less than 18 hours a week without pay?

1. Health problems →Go to D005
2. Childcare or housework →Go to D005
3. Learning or attending school →Go to D005
4. Want to take a rest →Go to D005
5. Not enough work to do →Go to D005
6. Others

D004. If others, please specify.

_______________________________

**Logic**

- **R cannot answer ‘Don’t know’ or’ Refuse to answer’ to D005.**

D005. Are you looking for a job?

1. Yes ⑤ No

**Logic**

- **R cannot answer ‘Don’t know’ or’ Refuse to answer’ to D006.**

D006. Which of the following best describes your current employment status with regard to working in the labor market?

[IWER: Retirement refers to having stopped income-earning activities and currently not working or engaging only in pastime work. Also, she/he doesn’t have intention to do anything more serious than pastime work as long as there is no special change in circumstances.]

1. I have never worked as being employed for income
2. I have done a little work this and that but I have never had a clear job
3. Currently retired (no intend to work in the future unless circumstances change)
4. Intend to work if wage and working conditions meet my expectations under adequate circumstances
5. Intend to work if wage and working conditions meet my expectations
6. Intend to work if a proper job is provided now even if wage and working conditions are slightly below my expectations

**< Job history >**

**Logic**

- **If R answered in the previous interview that he/she was working, then ask D007. If R answered in the previous interview that he/she was not working, then ask D008.**
- **If R answered in the previous interview that he/she was working, then ask D007.**

D007. Now I am going to ask you about your job change since the previous interview.

In the previous interview, you responded that you were working as a [job title: occupation] at [name of workplace: industry]. Is this correct?

[IWER: Moving to another workplace is considered a job change. However, it is excluded if the name of workplace has changed. For the other cases, regard as changes in position or job title at workplace.]

① Yes → (wage workers) Go to D023, (non-wage worker) Go to D033

⑤ No →Go to D009

**Logic**

- **If R answered in the previous interview that he/she was not working and R≠① at D006, then ask D008.**

D008. Now I am going to ask you about your job change since the previous interview. In the previous interview, you responded that you were not working. Is this correct?

1. Yes →Go to D043 ⑤ No

D009. Please state the work you were doing at the time of the previous interview. What kind of work did you do? (Employment status)

① Employed by another person or a company and received wages

③ Self-employed or unpaid family worker

⑤ Not working at the time of the previous interview →Go to D043

D010. What kind of business or industry do you work in--that is, what do they make

or do at the place where you work?

1. Agriculture/forestry →Go to D015
2. Fishing →Go to D015
3. Mining and quarrying →Go to D015
4. Manufacturing →Go to D011
5. Electricity, gas, and water supply →Go to D015
6. Construction →Go to D015
7. Wholesale and retail trade →Go to D012
8. Hotels and restaurants →Go to D013
9. Transport →Go to D015
10. Post and telecommunications →Go to D015
11. Financial and insurance activities →Go to D015
12. Real estate and renting and leasing →Go to D015
13. Other activities →Go to D014

D011. To be specific, which industrial classification best describes your workplace?

① Manufacture of food products and beverages

② Manufacture of textiles, wearing apparel, luggage, and footwear

③ Manufacture of metal and machinery materials and products

④ Manufacture of electronic and electric materials and products

⑤ Manufacture of motor vehicles and transport equipment products

⑥ Manufacture of other products

→Go to D015

D012. To be specific, which industrial classification best describes your workplace?

① Sale of motor vehicles

② Wholesale

③ Retail trade

→Go to D015

D013. To be specific, which industrial classification best describes your workplace?

① Hotels

② Restaurants

→Go to D015

D014. To be specific, which industrial classification best describes your workplace?

① Business services

② Public administration, defense, and compulsory social security

③ Education

④ Health and social work

⑤ Recreation, culture, and sports related services

⑥ Other community, repair, and personal services

⑦ Housekeeping services

⑧ International organizations

D015. What kind of work did you do in your workplace?

1. Managers →Go to D020
2. (Associate) Professionals →Go to D020
3. Clerical support workers →Go to C016
4. Service workers →Go to D017
5. Sales workers →Go to D020
6. Skilled agricultural, forestry, and fishery workers →Go to D018
7. Craft and related trades workers →Go to D020
8. Plant and machine operators, and assemblers →Go to D020
9. Elementary occupations →Go to D019

Armed forces occupation →Go to D020

D016. To be specific, which occupational classification best describes your work?

1. General office clerks
2. Customer services clerks

→Go to D020

D017. To be specific, which occupational classification best describes your work?

1. Personal services related workers
2. Cooking and food service workers
3. Travel and transport related workers
4. Protective service workers

→Go to D020

D018. To be specific, which occupational classification best describes your work?

1. Skilled agricultural and livestock workers
2. Skilled forestry workers
3. Skilled fishery workers

→Go to D020

D019. To be specific, which occupational classification best describes your work?

1. Housekeeping, cleaning, and laundry workers
2. Building management and security related workers
3. Delivery, moving, and meter reading workers
4. Sanitation and cleaning environment related workers
5. Other services related elementary workers
6. Agriculture, forestry, and fishery related elementary workers
7. Manufacturing related elementary workers
8. Mining and construction related elementary workers
9. Transport related elementary workers

**Logic**

- **If D009=①, then ask D020~D022.**

D020. What was your official position or job title? [IWER: Mark 0 if no official position or title]

ⓞ No position or job title

① Staff member

② Assistant foreman

③ Foreman

④ Assistant manager

⑤ Manager

⑥ Deputy general manager

⑦ General manager

⑧ Board member

D021. What was your current employment status?

| **[IWER]**   - **Regular worker**: Workers with an employment contract of more than one year or workers who may continue work as long as they want without a predefined contract period - **Temporary worker**: Workers with an employment contract of more than one month and less than one year or workers who expect the work to be terminated within one year despite of no predefined contract period (Even if he/ she has been working for the company for a long time and/ or expects to continue working in the future, as long as the contract period is less than 1 year, he/ she has to be considered as a temporary worker) - **Daily worker**: Workers with an employment contract of less than one month, or workers who are hired and paid on a daily basis, or workers who work in various places without any designated place |
| --- |

① Regular worker

③ Temporary worker

⑤ Daily worker

D022. Did you work on a part-time basis or a full-time basis?

| **[IWER]**   - **Part-time**: Working on a part-time basis or as a side job, workers who work less hours than others in the same job, or get paid on hourly basis - **Full-time**: Working on a full-time basis, workers who work full-working days and full-hours, or not get paid on hourly basis |
| --- |

1. Part-time basis ⑤ Full-time basis

**Logic**

- **If R was identified as a wage worker in the previous interview or D009=①, then go to D023~D032, D701~D704, D711~D717.**
- **If R was identified as a self-employed/unpaid family worker in the previous interview or D009=③, then go to D033~D035, D711~D717.**

**Logic**

- **If R was identified as a wage worker in the previous interview or D009=①, then ask D023.**
- **If D024=⑤, then ask D027.**

**Logic**

- **(As loop=1) If D007=① or D009=① , then ask D023.**

D023. (As loop=1) You responded in the previous interview that your position or job title was ______. Choose a position/job title that best describes ______ from the following. (Workplace in the previous interview: [name of workplace, job title])

ⓞ No position or job title

① Staff member

② Assistant foreman

③ Foreman

④ Assistant manager

⑤ Manager

⑥ Deputy general manager

⑦ General manager

⑧ Board member

D024. (As loop=1) Did your position or job title change within the workplace since the previous interview? (Workplace in the previous interview: [name of workplace, job title])

(As loop>=2) Did your position or job title change again after that? (Workplace in the previous interview(2006/2018): [name of workplace, job title])

1. Yes ⑤ No →Go to D027

D025. What was your new position or job title? (Workplace in the previous interview: [name of workplace, job title])

ⓞ No position or job title

① Staff member

② Assistant foreman

③ Foreman

④ Assistant manager

⑤ Manager

⑥ Deputy general manager

⑦ General manager

⑧ Board member

D026. When were you appointed to the new position or title? (Unit: year and month combined into 6 digits, workplace in the previous interview: [name of workplace, job title])

[IWER: Enter the year and month using 6 digits. For example, mark 201701 for January 2017. If the month is not clear, enter 201700.]

_______________________________

**Logic**

- **If D027=⑤, then ask D030.**

D027. (As loop=1) You responded in the previous interview that your employment status was ______. Did your employment status change within the workplace since then? (Workplace in the previous interview: [name of workplace, job title])

(As loop>=2) Did your employment status change again after that? (Workplace in the previous interview: [name of workplace, job title])

1. Yes ⑤ No →Go to D030

D028. What was your new employment status? (Workplace in the previous interview: [name of workplace, job title])

| **[IWER]**   - **Regular worker**: Workers with an employment contract of more than one year or workers who may continue work as long as they want without a predefined contract period - **Temporary worker**: Workers with an employment contract of more than one month and less than one year or workers who expect the work to be terminated within one year despite of no predefined contract period (Even if he/ she has been working for the company for a long time and/ or expects to continue working in the future, as long as the contract period is less than 1 year, he/ she has to be considered as a temporary worker) - **Daily worker**: Workers with an employment contract of less than one month, or workers who are hired and paid on a daily basis, or workers who work in various places without any designated place |
| --- |

① Regular worker

③ Temporary worker

⑤ Daily worker

D029. When did your employment status change? (unit: year and month combined into 6 digits, workplace in the previous interview: [name of workplace, job title])

[IWER: Enter the year and month using 6 digits. For example, mark 201701 for January 2017. If the month is not clear, enter 201700.]

_______________________________

**Logic**

- **If D030=⑤, then ask D701.**

D030. (As loop=1) You responded in the previous interview that your working hours was _______. Did your working hours change within the workplace since then? (Workplace in the previous interview: [name of workplace, job title])

(As loop>=2) Did your working hours change again after that? (Workplace in the previous interview: [name of workplace, job title])

1. Yes ⑤ No →Go to D701

D031. Did you work on a part-time basis or a full-time basis? (Workplace in the previous interview: [name of workplace, job title])

| **[IWER]**   - **Part-time**: Working on a part-time basis or as a side job, workers who work less hours than others in the same job, or get paid on hourly basis - **Full-time**: Working on a full-time basis, workers who work full-working days and full-hours, or not get paid on hourly basis |
| --- |

1. Part-time basis ⑤ Full-time basis

D032. When did your working hours change? (unit: year and month combined into 6 digits, workplace in the previous interview: [name of workplace, job title])

[IWER: Enter the year and month using 6 digits. For example, mark 201701 for January 2017. If the month is not clear, enter 201700.]

_______________________________

**Logic**

- **If D701=⑤, then ask D711.**

D701. (As loop=1) Did you transfer to another team or to another location within the same workplace since the previous interview? (Workplace in the previous interview): [name of workplace, job title])

(As loop>=2) Did you have another transfer after that? (Workplace in the previous interview: [name of workplace, job title])

1. Yes ⑤ No →Go to D711

D702. When did you transfer to another team or to another location? (unit: year and month combined into 6 digits, workplace in the previous interview: [name of workplace, job title])

[IWER: Enter the year and month using 6 digits. For example, mark 201701 for January 2017. If the month is not clear, enter 201700.]

_______________________________

D703. How many employees do you have in your new location including yourself? (unit: a person)

_______________________________ People [Range: 1~9997]

Don’t know/ Refuse to answer →Go to D704

D704. Then what is the estimated number of employees that worked in the new location?

1. 1 to 4 people
2. 5 to 9 people
3. 10 to 29 people
4. 30 to 49 people
5. 50 to 99 people
6. 100 to 299 people
7. 300 to 499 people
8. 500 to 999 people
9. More than 1,000 people

**Logic**

- **If R was identified as a self-employed/unpaid family worker in the previous interview or D009=⑤, then ask D033.**
- **If D033=⑤, then ask D711.**

D033. (As loop=1) You responded in the previous interview that your employment status was ______. Did your employment status change within the workplace since then? (Workplace in the previous interview): [name of workplace, job title])

(As loop>=2) Did your employment status change again after that? (Workplace in the previous interview): [name of workplace, job title])

[IWER: Change in employment status means a change from “self-employed to unpaid family worker” or a change from “unpaid family worker to self-employed (employer)”.]

1. Yes ⑤ No →Go to D711

D034. What was your new employment status? (Workplace in the previous interview: [name of workplace, job title])

① Self-employed (employer) to unpaid family worker

⑤ Unpaid family worker to self-employed (employer)

D035. When did your employment status change? (unit: year and month combined into 6 digits, workplace in the previous interview: [name of workplace, job title])

[IWER: Enter the year and month using 6 digits. For example, mark 201701 for January 2017. If the month is not clear, enter 201700.]

_______________________________

**Logic**

- **If D711=⑤, then ask D036.**

D711. (As loop=1) You responded in the previous interview that the kind of work you did in the workplace was ___. Did your work change within the workplace since then? (Workplace in the previous interview): [name of workplace, job title])

(As loop>=2) Did your work change again after that? (Workplace in the previous interview): [name of workplace, job title])

[IWER: Only change in major group of occupational classification is considered work change.]

1. Yes ⑤ No →Go to D036

D712. What was your new work? (Workplace: [name of workplace, job title])

1. Managers →Go to D717
2. (Associate) Professionals →Go to D717
3. Clerical support workers →Go to D713
4. Service workers →Go to D714
5. Sales workers →Go to D717
6. Skilled agricultural, forestry, and fishery workers →Go to D715
7. Craft and related trades workers →Go to D717
8. Plant and machine operators, and assemblers →Go to D717
9. Elementary occupations →Go to D716
10. Armed forces occupation →Go to D717

D713. To be specific, which occupational classification best describes your work?

1. General office clerks
2. Customer services clerks

D714. To be specific, which occupational classification best describes your work?

1. Personal services related workers
2. Cooking and food service workers
3. Travel and transport related workers
4. Protective service workers

D715. To be specific, which occupational classification best describes your work?

1. Skilled agricultural and livestock workers
2. Skilled forestry workers
3. Skilled fishery workers

D716. To be specific, which occupational classification best describes your work?

1. Housekeeping, cleaning, and laundry workers
2. Building management and security related workers
3. Delivery, moving, and meter reading workers
4. Sanitation and cleaning environment related workers
5. Other services related elementary workers
6. Agriculture, forestry, and fishery related elementary workers
7. Manufacturing related elementary workers
8. Mining and construction related elementary workers
9. Transport related elementary workers

D717. When did your work change? (unit: year and month combined into 6 digits, workplace in the previous interview): [name of workplace, job title])

[IWER: Enter the year and month using 6 digits. For example, mark 201701 for January 2017. If the month is not clear, enter 201700.]

_______________________________

**Logic**

- **R cannot answer ‘Don’t know’ or’ Refuse to answer’ to D036.**

D036. Are you currently working in the workplace you responded you were working in the previous interview? (workplace in the previous interview): [name of workplace, job title])

[IWER: If R moved to another workplace, he/she is considered having a different job.]

1. Yes →Go to D085 ⑤ No, I quit working

D037. When did you quit this job? (unit: year and month combined into 6 digits)

[IWER: Enter the year and month using 6 digits. For example, mark 201701 for January 2017. If the month is not clear, enter 201700.]

_______________________________ [Range: 200600 ~ 201811]

D038. Why did you quit? [IWER: Choices are different by wage workers and non-wage workers]

(In the case of wage workers)

1. Bankruptcy, closure, temporary shutdown, etc. of business
2. Layoff, recommended resignation, voluntary early retirement, etc.
3. Contract expired
4. Retirement (at the mandatory retirement age)
5. Too low wage
6. Temporary job or no vision of work
7. Aptitude, knowledge, and skills did not match well
8. Long working hours/unfavorable working conditions
9. Marriage, childbirth, childcare, etc.
10. Further education or military service
11. Found a better job with better wage/ working condition
12. To start up a new business or to help family business
13. Poor health
14. Wanted to take a break
15. Others

(In the case of non-wage worker)

1. Business was falling or slow
2. No vision of work
3. Aptitude, knowledge, and skills did not match well
4. Long working hours/unfavorable working conditions
5. Marriage, childbirth, childcare, etc.
6. Moved house
7. Care for sick family member
8. Found a paid job
9. To start up a new business
10. For unpaid assistance in family business
11. Poor health
12. Wanted to take a break
13. Others

**Logic**

- **If R was identified as a wage worker in the previous interview or D009=①, then ask D039~D042.**

D039. Did you receive or expect to receive legal retirement benefits (or benefits based on collective bargaining agreement, employment rules, etc.) when you quit this job?

1. Received or expected to receive
2. Not applicable →Go to D041

D040.How much did you receive?

[IWER: Please enter ‘100,000,000 Korean won (one hundred million won)’ first then, enter ’10,000 Korean won (MW)’.]

______one hundred million won [Range: 1~99] ________ MW [Range: 0 ~ 9997]

D041. Did you receive extra retirement benefits (for example, honorary retirement allowance or retirement bonus) ?

1. Yes ⑤ No →Go to D043

D042. How much did you receive?

[IWER: Please enter ‘100,000,000 Korean won (one hundred million won)’ first then, enter ’10,000 Korean won (MW)’.]

______one hundred million won [Range: 1~99] ________ MW [Range: 0 ~ 9997]

**Logic**

- **R cannot answer ‘Don’t know’ or’ Refuse to answer’ to D043.**
- **If D008=① or D036=⑤, then ask D043.**

D043. Did you find a new job since the previous interview?

1. Yes ⑤ No →Go to D085

**Logic**

- **Until D081=① or D084=⑤, loop D044~D084.**

D044. As for your new job, were you employed by another person with wage? Or did you set-up your own business or help your family business? (Employment status)

[IWER: Please list all the jobs in order R had since the previous interview. For example, if R was a wage worker between March and September 2017, and then became an unpaid family worker from October 2017, the order is to be from ‘wage worker’ to ‘unpaid family worker’.]

1. Employed by another person or a company and received wage

⑤ Self-employed or unpaid family worker

D045. When did you start this job? (unit: year and month combined into 6 digits)

[IWER: Enter the year and month using 6 digits. For example, mark 201801 for January 2018. If the month is not clear, enter 201800.]

_______________________________ [Range: 200600 ~ 201811]

D046. What does your workplace do?

1. Agriculture/forestry →Go to D051
2. Fishing →Go to D051
3. Mining and quarrying →Go to D051
4. Manufacturing
5. Electricity, gas, and water supply →Go to D051
6. Construction →Go to D051
7. Wholesale and retail trade →Go to D048
8. Hotels and restaurants →Go to D049
9. Transport →Go to D051
10. Post and telecommunications →Go to D051
11. Financial and insurance activities →Go to D051
12. Real estate and renting and leasing →Go to D051
13. Other activities →Go to D050

D047. To be specific, which industrial classification best describes your workplace?

① Manufacture of food products and beverages

② Manufacture of textiles, wearing apparel, luggage, and footwear

③ Manufacture of metal and machinery materials and products

④ Manufacture of electronic and electric materials and products

⑤ Manufacture of motor vehicles and transport equipment products

⑥ Manufacture of other products

→Go to D051

D048. To be specific, which industrial classification best describes your workplace?

① Sale of motor vehicles

② Wholesale

③ Retail trade

→Go to D051

D049. To be specific, which industrial classification best describes your workplace?

① Hotels

② Restaurants

→Go to D051

D050. To be specific, which industrial classification best describes your workplace?

① Business services

② Public administration, defense, and compulsory social security

③ Education

④ Health and social work

⑤ Recreation, culture, and sports related services

⑥ Other community, repair, and personal services

⑦ Housekeeping services

⑧ International organizations

→Go to D051

D051. What kind of work did you do in the workplace?

1. Managers →(wage workers) Go to D056, (non-wage worker) Go to D071
2. (Associate) Professionals→(wage workers) Go to D056, (non-wage worker) Go to D071
3. Clerical support workers
4. Service workers →Go to D053
5. Sales workers →(wage workers) Go to D056, (non-wage worker) Go to D071
6. Skilled agricultural, forestry, and fishery workers →Go to D054
7. Craft and related trades workers →(wage workers) Go to D056, (non-wage worker) Go to D071
8. Plant and machine operators, and assemblers →(wage workers) Go to D056, (non-wage worker) Go to D071
9. Elementary occupations →Go to D055
10. Armed forces occupation→(wage workers) Go to D056, (non-wage worker) Go to D071

D052. To be specific, which occupational classification best describes your work?

1. General office clerks
2. Customer services clerks

→(wage workers) Go to D056, (non-wage worker) Go to D071

D053. To be specific, which occupational classification best describes your work?

1. Personal services related workers
2. Cooking and food service workers
3. Travel and transport related workers
4. Protective service workers

→(wage workers) Go to D056, (non-wage worker) Go to D071

D054. To be specific, which occupational classification best describes your work?

1. Skilled agricultural and livestock workers
2. Skilled forestry workers
3. Skilled fishery workers

→(wage workers) Go to D056, (non-wage worker) Go to D071

D055. To be specific, which occupational classification best describes your work?

1. Housekeeping, cleaning, and laundry workers
2. Building management and security related workers
3. Delivery, moving, and meter reading workers
4. Sanitation and cleaning environment related workers
5. Other services related elementary workers
6. Agriculture, forestry, and fishery related elementary workers
7. Manufacturing related elementary workers
8. Mining and construction related elementary workers
9. Transport related elementary workers

→(wage workers) Go to D056, (non-wage worker) Go to D071

**Logic**

- **If D044=① (wage workers), then ask D056~D070, D731~D734, D741~D747.**

D056. About how many employees work for your company or organization at all the locations such as a head office, branch offices, plants and all the other fields? (unit: a person, workplace since [D045]: [Industry D046] [Occupation D051])

_______________________________ people [Range: 1~9997] →Go to D721

Don't know/Refuse to answer →Go to D057

D057. Then what is the estimated number of employees that worked in your company in total including all the locations such as a head office, branch offices, plants and all the other fields? (unit: number of people, workplace since [D045]: [Industry D046] [Occupation D051])

1. 1 to 4 people
2. 5 to 9 people
3. 10 to 29 people
4. 30 to 49 people
5. 50 to 99 people
6. 100 to 299 people
7. 300 to 499 people
8. 500 to 999 people
9. More than 1,000 people

D721. About how many employees work for this company or organization at the location

where you work including yourself?

_______________________________people [Range: 1~9997] →Go to D058

Don't know/Refuse to answer →Go to D722

D722. Then what is the estimated number of employees that worked in the location where you worked?

1. 1 to 4 people
2. 5 to 9 people
3. 10 to 29 people
4. 30 to 49 people
5. 50 to 99 people
6. 100 to 299 people
7. 300 to 499 people
8. 500 to 999 people
9. More than 1,000 people

D058. What was your position or job title at the workplace? (Workplace since [D045]: [Industry D046] [Occupation D051])

ⓞ No position or job title

① Staff member

② Assistant foreman

③ Foreman

④ Assistant manager

⑤ Manager

⑥ Deputy general manager

⑦ General manager

⑧ Board member

D059. What was your current employment status? (Workplace since [D045]: [Industry D046] [Occupation D051])

| **[IWER]**   - **Regular worker**: Workers with an employment contract of more than one year or workers who may continue work as long as they want without a predefined contract period - **Temporary worker**: Workers with an employment contract of more than one month and less than one year or workers who expect the work to be terminated within one year despite of no predefined contract period (Even if he/ she has been working for the company for a long time and/ or expects to continue working in the future, as long as the contract period is less than 1 year, he/ she has to be considered as a temporary worker) - **Daily worker**: Workers with an employment contract of less than one month, or workers who are hired and paid on a daily basis, or workers who work in various places without any designated place |
| --- |

① Regular worker

③ Temporary worker

⑤ Daily worker

D060. Did you work on a part-time basis or a full-time basis? (workplace since [D045]: [Industry D046] [Occupation D051])

| **[IWER]**   - **Part-time**: Working on a part-time basis or as a side job, workers who work less hours than others in the same job, or get paid on hourly basis - **Full-time**: Working on a full-time basis, workers who work full-working days and full-hours, or not get paid on hourly basis |
| --- |

1. Part-time basis ⑤ Full-time basis

D061. Did your position / job title, employment status, working hours, work, or location change at this workplace? (Workplace since [D045]: [Industry D046] [Occupation D051])

[IWER: Enter ‘Yes’ if one of the following changed: job position/title, employment status, working hours, work or location.]

1. Yes ⑤ No → Go to D081

**Logic**

- **If D062=⑤, then ask D065.**

D062. (As loop=1) You have responded that your position or job title was [D058]. Did your position or job title change since then? (workplace since [D045]: [Industry D046] [Occupation D051])

(As loop>=2) Did your position or job title change again after that? (workplace since [D045]: [Industry D046] [Occupation D051])

1. Yes ⑤ No →Go to D065

D063. What was your new position or job title? (workplace since [D045]: [Industry D046] [Occupation D051])

ⓞ No position or job title

① Staff member

② Assistant foreman

③ Foreman

④ Assistant manager

⑤ Manager

⑥ Deputy general manager

⑦ General manager

⑧ Board member

D064. When were you appointed to the new position or title? (unit: year and month combined into 6 digits, workplace since [D045]: [Industry D046] [Occupation D051])

[IWER: Enter the year and month using 6 digits. For example, mark 201801 for January 2018. If the month is not clear, enter 201800.]

_______________________________

**Logic**

- **If D065=⑤, then ask D068.**

D065. (As loop=1) You have responded that your employment status was [D059]. Did your employment status change within the workplace since then? (workplace since [D045]: [Industry D046] [Occupation D051])

(As loop>=2) Did your employment status change again after that? (workplace since [D045]: [Industry D046] [Occupation D051])

1. Yes ⑤ No →Go to D068

D066. What was your new employment status? (Workplace since [D045]: [Industry D046] [Occupation D051])

| **[IWER]**   - **Regular worker**: Workers with an employment contract of more than one year or workers who may continue work as long as they want without a predefined contract period - **Temporary worker**: Workers with an employment contract of more than one month and less than one year or workers who expect the work to be terminated within one year despite of no predefined contract period (Even if he/ she has been working for the company for a long time and/ or expects to continue working in the future, as long as the contract period is less than 1 year, he/ she has to be considered as a temporary worker) - **Daily worker**: Workers with an employment contract of less than one month, or workers who are hired and paid on a daily basis, or workers who work in various places without any designated place |
| --- |

① Regular worker

③ Temporary worker

⑤ Daily worker

D067. When did your employment status change? (unit: year and month combined into 6 digits, workplace since [D045]: [Industry D046] [Occupation D051])

[IWER: Enter the year and month using 6 digits. For example, mark 201701 for January 2017. If the month is not clear, enter 201700.]

_______________________________

**Logic**

- **If D068=⑤, then ask D731.**

D068. (As loop=1) You have responded that your working hours was [D060]. Did your working hours change since then? (workplace since [D045]: [Industry D046] [Occupation D051])

(As loop>=2) Did your working hours change again after that? (workplace since [D045]: [Industry D046] [Occupation D051])

1. Yes ⑤ No →Go to D731

D069. Did you work on a part-time basis or a full-time basis? (workplace since [D045]: [Industry D046] [Occupation D051])

| **[IWER]**   - **Part-time**: Working on a part-time basis or as a side job, workers who work less hours than others in the same job, or get paid on hourly basis - **Full-time**: Working on a full-time basis, workers who work full-working days and full-hours, or not get paid on hourly basis |
| --- |

1. Part-time basis ⑤ Full-time basis

D070. When did your working hours change? (unit: year and month combined into 6 digits, workplace since [D045]: [Industry D046] [Occupation D051])

[IWER: Enter the year and month using 6 digits. For example, mark 201701 for January 2017. If the month is not clear, enter 201700.]

_______________________________

**Logic**

- **If D731=⑤, then ask D741.**

D731. (As loop=1) Did you transfer to another team or to another location within the same workplace since the previous interview? (Workplace since [D045]: [Industry D046] [Occupation D051])

(As loop>=2) Did you move again to a new location after that? (Workplace since [D045]: [Industry D046] [Occupation D051])

1. Yes ⑤ No →Go to D741

D732. When did you transfer to a new location? (unit: year and month combined into 6 digits, workplace since [D045]: [Industry D046] [Occupation D051])

[IWER: Enter the year and month using 6 digits. For example, mark 201701 for January 2017. If the month is not clear, enter 201700.

_______________________________

D733. How many employees worked in the new location? (unit: a person)

_______________________________ people [Range: 1~9997]

Don't know/Refuse to answer →Go to D734

D734. Then what is the closest approximation to the actual number of employees that worked in the new location?

1. 1 to 4 people
2. 5 to 9 people
3. 10 to 29 people
4. 30 to 49 people
5. 50 to 99 people
6. 100 to 299 people
7. 300 to 499 people
8. 500 to 999 people
9. More than 1,000 people

**Logic**

- **If D044=⑤(non-wage worker), then ask D071~D080, D741~D747.**

D071. What was your current employment status? (workplace since [D045]: [Industry D046] [Occupation D051])

① Self-employed (employer)

⑤ Unpaid family worker

D072. How many employees who are officially hired and paid do you have in your business? Please exclude temporary workers such as those employed in farming seasons or busy times (unit: a person, workplace since [D045]: [Industry D046] [Occupation D051])

[IWER: Mark 0 if there was no employee.]

_______________________________People [Range: 0~9997] →Go to D074

Don't know/Refuse to answer →Go to D073

D073. Then what is the estimated number of employees that worked in your business?

(Workplace since [D045]: [Industry D046] [Occupation D051])

1. 1 to 4 people
2. 5 to 9 people
3. 10 to 29 people
4. 30 to 49 people
5. 50 to 99 people
6. 100 to 299 people
7. 300 to 499 people
8. 500 to 999 people
9. More than 1,000 people

D074. Except those employees who are hired and paid officially, do you also have any unpaid family workers? If so, please state the number including yourself. (unit: a person, workplace since [D045]: [Industry D046] [Occupation D051])

[IWER: Mark 0 if there was no employee.]

_______________________________ People [Range: 0~20]

**Logic**

- **If D071=①(self-employed workers), then ask D075~D077.**

D075. Did you have any business partner in your business? (Workplace since [D045]: [Industry D046] [Occupation D051])

1. Yes ⑤ No →Go to D078

D076. How many partners did you have? Please state the number including you. (unit: a person, workplace since [D045]: [Industry D046] [Occupation D051])

_______________________________ people [Range: 1~20]

D077. What was your share of ownership in this business? (unit: %, workplace since [D045]: [Industry D046] [Occupation D051])

_______________________________ % [Range: 1~99]

**Logic**

- **If D078=⑤, then ask D741.**

D078. (As loop=1) Did your employment status change within the workplace since then? (Workplace since [D045]: [Industry D046] [Occupation D051])/ (As loop>=2) Did your employment status change again after that? (Workplace since [D045]: [Industry D046] [Occupation D051])

[IWER: Change in employment status means a change from “self-employed to unpaid family worker” or a change from “unpaid family worker to self-employed (employer)”.]

1. Yes ⑤ No →Go to D741

D079. What was your new employment status?(Workplace since [D045]: [Industry D046] [Occupation D051])

① Self-employed (employer) to unpaid family worker

⑤ Unpaid family worker to self-employed (employer)

D080. When did your employment status change? (unit: year and month combined into 6 digits, workplace since [D045]: [Industry D046] [Occupation D051])

[IWER: Enter the year and month using 6 digits. For example, mark 201701 for January 2017. If the month is not clear, enter 201700.]

_______________________________

**Logic**

- **If D741=⑤, then ask D081.**

D741. (As loop=1) You have responded that your job was [D051]. Did your work change within the workplace since then? (Workplace since [D045]: [Industry D046] [Occupation D051])/ (As loop>=2) Did your work change within the workplace again after that?

[IWER: Only change in major group of occupational classification is considered work change.]

1. Yes ⑤ No →Go to D081

D742. What was your new work? (Workplace since [D045]: [Industry D046] [Occupation D051])

1. Managers →Go to D747
2. (Associate) Professionals →Go to D747
3. Clerical support workers →Go to D743
4. Service workers →Go to D744
5. Sales workers →Go to D747
6. Skilled agricultural, forestry, and fishery workers →Go to D745
7. Craft and related trades workers →Go to D747
8. Plant and machine operators, and assemblers →Go to D747
9. Elementary occupations →Go to D746
10. Armed forces occupation →Go to D747

D743. To be specific, which occupational classification best describes your work?

1. General office clerks
2. Customer services clerks

→Go to D747

D744. To be specific, which occupational classification best describes your work?

1. Personal services related workers
2. Cooking and food service workers
3. Travel and transport related workers
4. Protective service workers

→Go to D747

D745. To be specific, which occupational classification best describes your work?

1. Skilled agricultural and livestock workers
2. Skilled forestry workers
3. Skilled fishery workers

→Go to D747

D746. To be specific, which occupational classification best describes your work?

1. Housekeeping, cleaning, and laundry workers
2. Building management and security related workers
3. Delivery, moving, and meter reading workers
4. Sanitation and cleaning environment related workers
5. Other services related elementary workers
6. Agriculture, forestry, and fishery related elementary workers
7. Manufacturing related elementary workers
8. Mining and construction related elementary r workers
9. Transport related elementary workers

→Go to D747

D747. When did your work change? (unit: year and month combined into 6 digits, workplace since [D045]: [Industry D046] [Occupation D051])

[IWER: Enter the year and month using 6 digits. For example, mark 201701 for January 2017. If the month is not clear, enter 201700.]

_______________________________

**Logic**

- **R cannot answer ‘Don’t know’ or’ Refuse to answer’ to D081.**

D081. Are you currently working in this workplace? (Workplace since [D045]: [Industry D046] [Occupation D051])

[IWER:

- In case of wage workers, moving to another workplace is regarded as having a different job.

- In case of non-wage worker, changing business content is regarded as having a different job.

- Changing employment status from wage worker to non-wage worker or vice versa is regarded as having a different job.]

1. Yes →Go to D085 ⑤ No

D751. How many days per week did you work on average?

_______________________________ days [Range: 1~7]

D752. How many hours per week did you work on average including lunch time, break, etc.?

_______________________________ hours [Range: 1~160]

**Logic**

- **If D044=① or D071=①, then ask D753. If D071=⑤ (unpaid family workers), then ask D082.**

D753. How much did you receive from monthly average net earnings or profits? As for a self-employer, please tell the net income including all the expenses for your business.

[IWER:

- “Please state profits including legally required deductions, such as taxes, national pension contributions, health insurance premiums and employment insurance premiums.”

- Mark 0 if R was self-employed and made no net income, and enter 999997 if R’s business is deficit.

- Please enter ‘100,000,000 Korean won (one hundred million won)’ first then, enter ’10,000 Korean won (MW)’.]

______one hundred million won [Range: 1~99] _________ MW [Range: 0 ~ 9997]

D082. When did you quit this job? (unit: year and month combined into 6 digits, workplace since [D045]: [Industry D046] [Occupation D051])

[IWER: Enter the year and month using 6 digits. For example, mark 201701 for January 2017. If the month is not clear, enter 201700.]

_______________________________ [Range: 200600 ~ 201811]

D083. Why did you quit? (workplace since [D045]: [Industry D046] [Occupation D051])

[IWER: Choices are different by wage workers and non-wage workers]

(In the case of wage workers)

1. Bankruptcy, closure, temporary shutdown, etc. of business
2. Layoff, recommended resignation, voluntary early retirement, etc.
3. Contract expired
4. Retirement (at the mandatory retirement age)
5. Too low wage
6. Temporary job or no vision of work
7. Aptitude, knowledge, and skills did not match well
8. Long working hours/unfavorable working conditions
9. Marriage, childbirth, childcare, etc.
10. Further education or military service
11. Found a better job with better wage/ working condition
12. To start up a new business or to help family business
13. Poor health
14. Wanted to take a break
15. Others

(In the case of non-wage worker)

1. Business was falling or slow
2. No vision of work
3. Aptitude, knowledge, and skills did not match well
4. Long working hours/unfavorable working conditions
5. Marriage, childbirth, childcare, etc.
6. Moved house
7. Care for sick family member
8. Found a paid job
9. To start up a new business
10. For unpaid assistance in family business
11. Poor health
12. Wanted to take a break
13. Others

**Logic**

- **R cannot answer ‘Don’t know’ or’ Refuse to answer’ to D084.**

D084. Did you get a different job after you quit? (workplace since [D045]: [Industry D046] [Occupation D051])

1. Yes →Go to D044 ⑤ No

D085. Your current employment status is (answer from the related question). Now, I will ask questions according to your current employment status (wage worker, self-employed worker, unpaid family worker, job seeker/ non-worker, etc.).

1. Check

**<Current job: Wage worker>**

**Logic**

- **If D002=①, then go to a section of wage worker.**

**<Current job: A new wage worker>**

**Logic**

- **If D043=① (a new wage worker), then ask D101~D114.**

D101. Is the duration of the employment contract fixed or non-fixed at your current job?

1. Fixed ⑤ Not fixed →Go to D104

D102. How long is the fixed duration of your employment contract? (unit: a month)

[IWER: If R answered a year, calculate to months]

_______________________________ months [Range: 1~60]

D103. Has the current employment contract ever been renewed?

1. Yes →Go to D105 ⑤ No (In case of first contract)→Go to D105

D104. If the employment duration is not fixed, do you work for a few days or a few weeks when there is work available?

[IWER: Mark 'No' if R’s work lasts more than one month.]

1. Yes ⑤ No

D105. Did you receive a written employment contract from your current employer?

1. Yes ⑤ No

D106. Do you think you could continue your current job unless the company closes down due to business difficulties or you are laid off?

1. Yes →Go to D110 ⑤ No

D107. How long do you expect to work at your current workplace?

1. Less than one year
2. One to two years
3. Two to three years →Go to D110
4. More than three years →Go to D110

D108. Why do you expect so?

1. Because the fixed contract period will be expired →Go to D110
2. Because the contract will be expired conventionally even if it’s not written →Go to D110
3. Because my condition includes that I will resign upon employer’s request →Go to D110
4. Because the current job / project will be completed →Go to D110
5. Because the person I am replacing will return to work →Go to D110
6. Because I can only work during certain seasons →Go to D110
7. Because I plan to find another job that better suits my job aptitude, ability, and preference →Go to D110
8. Because I will reach at retirement age set by regulations / practice →Go to D110
9. Because of family care responsibility, poor health, etc. →Go to D110
10. Others

D109. If others, please specify.

_______________________________

D110. Do you receive your wages from your current employer directly or from a dispatch company or a service contract agency?

| **[IWER]**   - **Dispatch company worker**: Workers who are employed by a dispatch company but work in the workplace of a person using their services, subject to the Act on the Protection of Dispatched Workers. Their wages and employment status are controlled by the dispatch company; however with regard to work-related instructions or work functions, they are under the direction and supervision of the company who uses the dispatch company.   - **Service contract agency worker**: Workers who are employed by a service agency but work in the workplace who uses this service agency. They are not subject to the Act on the Protection of Dispatched Workers. With regard to wages, employment status, work-related instructions, and so on, they are under the direction and supervision of the service agency. Examples include contract cleaning workers, contract security guards, etc. |
| --- |

1. Employer
2. Dispatch Company
3. Service Contract Agency

D111. Is there a mandatory retirement age at your current workplace?

1. Yes ⑤ No →Go to D113

D112. What is the mandatory retirement age? (unit: an age)

[IWER: Count a person’s age in full, not Korean age (same as American age).]

_______________________________ years old [Range: 45~80]

D113. Are there any cases where employees retire conventionally upon reaching at a certain age regardless of regulations?

1. Yes ⑤ No →Go to D115

D114. What is the age that employees retire conventionally? (unit: an age)

[IWER: Count a person’s age in full, not Korean age (same as American age).]

_______________________________ years old [Range: 45~97]

**< Information on job: All wage worker >**

D115. Are you in a position to supervise others?

1. Yes ⑤ No →Go to D117

D116. How many people are there under your supervision?

1. 1~5 people
2. 6~10 people
3. 11~15 people
4. 16~30 people
5. 31~99 people
6. More than 100 people

D117. Do you work based on a fixed-working hours at your current workplace?

1. Yes ⑤ No

D118. How many days per week did you work on average? (unit: a day)

_______________________________ days [Range: 1~7]

D119. How many hours per week did you work on average including lunch time, break, etc.?

(unit: an hour/per week)

_______________________________ hours [Range: 1~160]

D120. What do you think of your current working hours including regular and overtime hours?

1. Too long
2. Appropriate →Go to D127
3. Too short →Go to D124

D121. If you consider your working hours too long, would you like to reduce them even if your earnings are reduced?

1. Yes ⑤ No →Go to D127

D122. What % of your total working hours would you like to reduce? (unit: %)

_______________________________% [Range: 1~99]

D123. What % cut from your total earnings are you willing to accept? (unit: %)

_______________________________% [Range: 1~99] →Go to D127

D124. If you consider your working hours too short, are you willing to increase them if your earnings increase as a result?

1. Yes ⑤ No →Go to D127

D125. What % of your total working hours would you like to increase?

[IWER: Maximum is 100. if R answered up to 100%, just mark 100]

_______________________________% [Range: 1~100]

D126. What % of your total earnings has to be increased?

_______________________________% [Range: 1~99]

D127. Do you have regularly scheduled days off? If you do, how many days off do you have per month?

1. None
2. One day per month
3. 2~3 days per month
4. 4~5 days per month
5. 6~7 days per month
6. More than 8 days per month

D128. Except for national public holidays, how many days of paid leave do you have this year at your current workplace? (unit: a day)

[IWER: Mark 0 if there is no paid leave.]

_______________________________ days [Range: 0~365]

D129. How many days of paid sick leave do you have this year at your current workplace?

_______________________________days [Range: 0~365]

**Logic**

- - **If R started working at his/her current workplace before JAN 2017, go to D130.**

**If not, ask D131.**

D130. In the last one year, how many days did you miss from your work because of your health?

[IWER: Mark 0 if R didn’t miss any work day.]

_______________________________ days [Range: 0~365]

D131. How are you salaried on this job, paid by the hour, or what? Choose the most important one.

1. Yearly basis →Go to D133
2. Monthly basis →Go to D133
3. Weekly basis →Go to D133
4. Daily basis →Go to D133
5. Hourly basis →Go to D133
6. Contract-based →Go to D133
7. Performance-based (no basic salary) →Go to D133
8. Others

D132. If others, please specify.

_______________________________

D133. Is your wage automatically raised according to your career years?

1. Yes ⑤ No

D134. [Post-tax income] How much are you paid per month? (unit: 10,000 Korean won)

[IWER: “Please state the total amount **including** legally required deductions, such as taxes, national pension contributions, health insurance premiums and employment insurance premiums.”]

_______________________________ MW [Range: 1~9997]

D134-1. [Pre-tax income] How much are you paid per month? (unit: 10,000 Korean won)

[IWER: Please state the total amount **including** legally required deductions, such as taxes, national pension contributions, health insurance premiums and employment insurance premiums.”]

_______________________________ MW [Range: 1~9997]

D134-2. Therefore, your tax rate on earned income is to be ____ (%).

1. Check

D135. Now, I would like to ask you about your current state of insurance. First, are you currently enrolled in the Public Pension System?

1. Yes, enrolled in the National Pension by employee insured
2. Yes, enrolled in the Specific Corporate Pension (for private school teachers, government employees, military personnel)
3. Yes, enrolled in the National Pension by self-employed insured
4. No, not enrolled

D136. Are you currently enrolled in the National Health Insurance?

1. Yes, enrolled in the National Health Insurance as employee insured
2. Yes, enrolled in the National Health Insurance as self-employed insured
3. No, not enrolled

D137. Are you currently enrolled in the Unemployment Compensation Insurance?

1. Yes ⑤ No

D138. Does your employer get the Industrial Accident Compensation Insurance? The Industrial Accident Compensation Insurance provides protection against job-related accidents and injuries.

1. Yes ⑤ No

D139. What is the current pension or retirement plan in your company?

| **[IWER]**   - - **Retirement Insurance System**: An employer deposit retirement payment in an outside insurance company. When an employee retires, this insurance company will provide in annuity or lump-sum settlement.   - **Retirement Pension System**: As converting lump-sum payment into annuity, an employee will receive annuity payments after the retirement age (e.g. 55 y.o.) |
| --- |

1. Yes, Retirement Allowance System
2. Yes, Retirement Insurance System
3. Yes, Retirement Pension System →Go to D142
4. No, no retirement plan →Go to D143

D140. Did you withdraw retirement payment in the middle in your company?

1. Yes ⑤ No →Go to D143

D141. When was it? [IWER: Enter the year and month using 6 digits. For example, mark 201801 for January 2018. If the month is not clear, enter 201800.]

_______________________________ [Range: 190000~201811] →Go to D143

D142. What is the type of your retirement pension?

| **[IWER]**   - - **Defined Benefit**: Retirement payment is predetermined and employer’s contribution fluctuate on the basis of investment earnings of reserve.   - **Defined Contribution**: Employer’s contributions are pre set-up while employee’s future benefits fluctuate on the basis of investment earnings of reserve. |
| --- |

1. Defined Benefits (DB)
2. Defined Contributions (DC)

D143. Now I would like to ask you about incentive or fringe benefits at your company. Does your company provide meal allowances?

1. Yes ⑤ No

D144. Does your company support children’s school expenses?

1. Yes ⑤ No

D145. Does your company provide a loan for housing purchase?

1. Yes ⑤ No

D146. Does your employer compensate for lost wages in the case of temporary shutdown?

1. Yes ⑤ No

D147. Does your employer pay insurance premiums for your personal pension plan?

[IWER: Personal pension refers to privately owned retirement savings, not including the National Pension.]

1. Yes ⑤ No

D148. Is there a labor union at your current workplace?

1. Yes ⑤ No →Go to D154

D149. Are you eligible to join a labor union?

1. Yes ⑤ No →Go to D154

D150. Are you a member of a labor union?

1. Yes ⑤ No →Go to D152

D151. If you are a member, was membership mandatory?

1. Yes →Go to D155 ⑤ No →Go to D155

D152. If you are not a member, why did not you join?

1. Not satisfied with union activities →Go to D155
2. Dissuaded by my family, friends, colleagues, etc. →Go to D155
3. Dissuaded by my employer →Go to D155
4. Did not feel the need →Go to D155
5. Others

D153. If others, please specify.

_______________________________

D154. If the company had a labor union or if you were eligible to join a labor union, would you join it?

1. Yes ⑤ No

D155. How much do you agree with the following descriptions for your current job?

My job requires lots of physical effort.

1. Strongly agree
2. Agree
3. Disagree
4. Strongly disagree

D156. My job requires lifting heavy loads.

1. Strongly agree
2. Agree
3. Disagree
4. Strongly disagree

D157. My job requires stooping, kneeling, or crouching.

1. Strongly agree
2. Agree
3. Disagree
4. Strongly disagree

D158. My job requires good eyesight.

1. Strongly agree
2. Agree
3. Disagree
4. Strongly disagree

D159. My job requires intense concentration and attention to details.

1. Strongly agree
2. Agree
3. Disagree
4. Strongly disagree

D160. My job requires skills in dealing with people.

1. Strongly agree
2. Agree
3. Disagree
4. Strongly disagree

D161. My job requires me to work with computers.

1. Strongly agree
2. Agree
3. Disagree
4. Strongly disagree

D162. My job requires me to do more difficult things that it used to.

1. Strongly agree
2. Agree
3. Disagree
4. Strongly disagree

D163. Promotion is done on the basis of seniority in my company.

1. Strongly agree
2. Agree
3. Disagree
4. Strongly disagree

D164. My co-workers make older workers feel that they ought to retire before reaching at the retirement age.

1. Strongly agree
2. Agree
3. Disagree
4. Strongly disagree

D165. My employer would let older workers move to a less demanding job with less pay if they wanted to.

1. Strongly agree
2. Agree
3. Disagree
4. Strongly disagree

D166. I am satisfied with current wage.

1. Strongly agree
2. Agree
3. Disagree
4. Strongly disagree

D167. I feel my job is secure.

1. Strongly agree
2. Agree
3. Disagree
4. Strongly disagree

D168. I am satisfied with the work environment of my job.

1. Strongly agree
2. Agree
3. Disagree
4. Strongly disagree

D169. I am satisfied with my work at my current job.

1. Strongly agree
2. Agree
3. Disagree
4. Strongly disagree

D170. My job involves a lot of stress.

1. Strongly agree
2. Agree
3. Disagree
4. Strongly disagree

D171. In general, I am satisfied with my current job.

1. Strongly agree
2. Agree
3. Disagree
4. Strongly disagree

D172. Considering my educational background, the level of my current job is….

1. Very low
2. Low
3. Appropriate
4. High
5. Very high

D173. Considering my skills and expertise, the level of my current job is…

1. Very low
2. Low
3. Appropriate
4. High
5. Very high

D174. Would you like to continue working at your current job?

1. Yes, I would like to continue my current job, as it is →D178
2. Yes, I would like to increase work load at my current job →D178
3. Yes, I would like to transfer to add new work at my current job
4. Yes, I would like to decrease work load at my current job →D178
5. No, I would like to take a different job
6. No, I would like to quit working completely →D178

D175. What would be the ideal employment status for you?

1. Full-time wage worker
2. Part-time wage worker
3. Self-employed

D176. What do you want to do for your new job? Choose from below.

1. Managers
2. (Associate) Professionals
3. Clerical support workers
4. Service workers
5. Sales workers
6. Skilled agricultural, forestry, and fishery workers
7. Craft and related trades workers
8. Plant and machine operators, and assemblers
9. Elementary occupations
10. Armed forces occupation

D177. What would be the minimum wages or salaries per month? (Unit: 10,000 Korean won)

[IWER: Mark 0 if R does not matter.]

_______________________________ MW [Range: 0~99997]

D178. Now I want to ask about your retirement plan. At what age do you plan to retire?

[IWER:

- Count a person’s age in full, not Korean age (same as American age).

- Mark 0 if R plans to keep working as long as he/she is physically capable.

- Retirement refers to having stopped income-earning activities and currently not working or engaging only in pastime work. Also, she/he doesn’t have intention to do anything more serious than pastime work as long as there is no special change in circumstances.]

_______________________________ years old [Range: 0~97]

D179. Other than the main job you have been telling us, are you doing any other work for pay now such as a second job?

[IWER: Please include only paid-work as including non-paid activities such as community services]

1. Yes ⑤ No →Go to D184_3

D180. How many jobs do you currently have including the main job? (unit: number)

_______________________________ [Range: 2~7]

D181. What are the reasons for doing other work than your main job?

[IWER: Please state the most important reason only.]

1. To earn more money →Go to D183
2. For my health →Go to D183
3. Because it is the work I wanted to do →Go to D183
4. For self-development →Go to D183
5. To utilize spare time →Go to D183
6. To prepare myself when I quit my main job →Go to D183
7. To help society or other people →Go to D183
8. Others

D182. If others, please specify.

_______________________________

D183. Except your main job, how many hours do you usually work on these second jobs per week? (unit: an average hour per week)

_______________________________ hours [Range: 1~97]

D184. [Post-tax income] On average how much do you earn from these second jobs per month? (Unit: 10,000 Korean won)

[IWER: “Please state the total amount **including** legally required deductions, such as taxes, national pension contributions, health insurance premiums and employment insurance premiums.”]

_______________________________ MW [Range: 1~9997]

D184_1. [Pre-tax income] How much do you earn from these second jobs per month? (unit: 10,000 Korean won)

[IWER: Please state the total amount **including** legally required deductions, such as taxes, national pension contributions, health insurance premiums and employment insurance premiums.”]

_______________________________ MW [Range: 1~9997]

D184_2. Therefore, your tax rate on earned income is to be ____ (%).

1. Check

D184_3. What are the reasons for you to keep working without retiring? Please choose two main reasons. First : _________

1. (Finance) Working would help resolving economic problems after retirement
2. (Emotion) I am worried that I could overcome my sense of loss after retirement (Interaction) Working would help my relationships with family, friends, neighbors, and the community
3. (Social) Because I think I can continue to contribute to society by working.
4. (Health) Working would help me maintain my health
5. Others

→ From code 1~5, go to D184_3_1

D184_4. If others, please specify.

_______________________________

D184_3_1. Why do you keep working without retiring? Please choose two main reasons.

Second : _________

1. (Finance) Working would help resolving economic problems after retirement
2. (Emotion) I am worried that I could overcome my sense of loss after retirement
3. (Interaction) Working would help my relationships with family, friends, neighbors, and the community
4. (Social) Because I think I can continue to contribute to society by working.
5. (Health) Working would help me maintain my health
6. Others

→ From code 1~5, go to D184_5

D184_4_1. If others, please specify.

_______________________________

D184_5. Have you ever attended any employment programs sponsored by the government or community last year (2017)?

1. Yes ⑤ No →Go to D185

D184_6. Among the employment programs you have attended last year, what is the most recent one?

1. Elderly employment program →Go to D185.
2. Public work employment program →Go to D185
3. Community employment program →Go to D185
4. Others

D184_7. If others, please specify.

_______________________________

D185. [IWER: How often did R receive assistance in answering section D-EMPLOYMENT?]

1. Never →Go to DC515.
2. A few times →Go to DC515.
3. Most or all of the time →Go to DC515.
4. The section was done by a proxy respondent.

D186. If done by a proxy respondent, what is the proxy’s relationship to R? Please answer in view of the proxy.

1. Spouse
2. Mother
3. Father
4. Mother-in-law
5. Father-in-law
6. Brother/sister
7. Brother-in-law/sister-in-law
8. Son/daughter
9. Son-in-law/Daughter-in-law
10. Grandchild
11. Other relatives
12. Helper or other non-relatives

→Go to section DC515.

**Logic**

- **Ask below only if D001=①**

DC515. If the score ‘10’ is for the best state to work, how is the current state of your ability to work? Please indicate with the scale card where ‘0’ means ‘absolutely cannot work’ and ‘10’ means ‘the best state to work’.

|  |  |  |  |  |  |  |  |  |  |  |  |  |  |  |  |  |  |  |  |  |  | |
| --- | --- | --- | --- | --- | --- | --- | --- | --- | --- | --- | --- | --- | --- | --- | --- | --- | --- | --- | --- | --- | --- | --- |
|  |  |  |  |  |  |  |  |  |  |  |  |  |  |  |  |  |  |  |  |  |  | |
| 0 | | 1 | | 2 | | 3 | | 4 | | 5 | | 6 | | 7 | | 8 | | 9 | | 10 | |

DC516. As for your job tasks, how is your ability to work on physical tasks?

1. Very bad
2. Bad
3. Fair
4. Good
5. Very good

DC517. As for your job tasks, how is your ability to work on mental tasks?

1. Very bad
2. Bad
3. Fair
4. Good
5. Very good

→Go to section E (E. INCOME AND CONSUMPTION)

**<Current job: Self-employed worker>**

**Logic**

- **If D002=②, then go to a section of self-employed worker.**
- **Only a new self-employed worker can answer this section.**

**Logic**

- **If D043=①(a new self-employed worker), then ask D201~D211.**

D201. What is the most important reason for you to choose self-employed instead of another paid job? Please choose the most important reason.

1. Because I can earn more money →Go to D203
2. Because it is the work I wanted to do →Go to D203
3. Because it gives me more flexibility →Go to D203
4. Because of tax benefits →Go to D203
5. Because I couldn’t get the job I wanted in terms of inadequate salary, work environment, etc. →Go to D203
6. Because it was difficult to get any paid job →Go to D203
7. Others

D202. If others, please specify.

_______________________________

**Logic**

- **If D203=0 (Startup expenses=zero), then ask D204, If D203>0, then ask D205.**

D203. How much did you spend or invest to startup this business?

[IWER: Please enter ‘100,000,000 Korean won (one hundred million won)’ first then, enter ’10,000 Korean won (MW)’.]

______one hundred million won [Range: 1~99] _________ MW [Range: 0 ~ 9997]

D204. What is the reason that no startup expense is required?

1. Because it is retail business without a store →Go to D206
2. Because it was taken over without any cost →Go to D206
3. Because it is R’s own land or building →Go to D206
4. Others →Go to D206

D204-1. If others, please specify.

_______________________________

D205. How did you finance your business expenses? Please tell us how each of sources accounts for to finance. Please make sure the sum of all items should be same with D203. (unit: 10,000 Korean won)

[IWER:① R’s and his/her spouse's savings, ②Help or borrowings from R’s and his/her spouse’s parents, ③ Help or borrowings from R’s children/grandchildren, ④ Borrowings from R’s and his/her relatives, ⑤ Borrowings from other individuals, ⑥ Partner, ⑦ Loans from banks and other financial institutions, ⑧ Government subsidy, ⑨ Others]

| NO | ITEMS | MW |
| --- | --- | --- |
| D205a | Own/spouse's savings |  |
| D205b | Help or borrowings from parents/spouse’s parents |  |
| D205c | Help or borrowings from children/grandchildren |  |
| D205d | Borrowings from relatives/spouse ‘relatives |  |
| D205e | Borrowings from other individuals |  |
| D250f | Partner |  |
| D205g | Loans from banks and other financial institutions |  |
| D205h | Government subsidy |  |
| D205i | Others |  |
|  | Total | [D203] |

D206. Next I will ask you about the general difficulties people may face in starting a new business. Please answer which one you belonged to.

How difficult was it to secure sufficient financing?

1. Very difficult
2. Difficult
3. Fair
4. Easy
5. Very easy

D207. How difficult was it to secure technology?

1. Very difficult
2. Difficult
3. Fair
4. Easy
5. Very easy

D208. How difficult was it to secure appropriate human resources?

1. Very difficult
2. Difficult
3. Fair
4. Easy
5. Very easy

D209. How difficult was it to deal with administrative procedures (licenses, start-up support, etc.)?

1. Very difficult
2. Difficult
3. Fair
4. Easy
5. Very easy

D210. How difficult was it to choose the industry?

1. Very difficult
2. Difficult
3. Fair
4. Easy
5. Very easy

D211. How difficult was it to choose the business location?

1. Very difficult
2. Difficult
3. Fair
4. Easy
5. Very easy

**<Ask to all self-employed workers>**

D212. How much is the current monthly sales of your business? As for agricultural and fisheries industries, please tell based on the revenue from selling agricultural and fisheries’ products including expenses.

[IWER:- Enter 0 if there is currently no sales.

- Please enter ‘100,000,000 Korean won (one hundred million won)’ first then, enter ’10,000 Korean won (MW)’.]

______one hundred million won [Range: 1~99] ________ MW [Range: 0 ~ 9997] → D214

Don’t know →Go to D213

Refuse to answer →Go to D213

D213. What is the estimated number of your current monthly sales figure? (Unit: MW=10,000 Korean won)

1. Less than 100 MW
2. 100~300 MW
3. 300~500 MW
4. 500~800 MW
5. 800~1,000 MW
6. 1,000~3,000 MW
7. 3,000~5,000 MW
8. 5,000~10,000 MW
9. 10,000 MW or more

D214. How much is your monthly income of your current business? Please state a net income including all the expenses.

[IWER - Mark 0 if there is no net income, and mark 999997 if R is running a deficit

- Please enter ‘100,000,000 Korean won (one hundred million won)’ first then, enter ’10,000 Korean won (MW)’.]

______one hundred million won [Range: 1~99] ________ MW [Range: 0 ~ 9997]

D215. How many days per week did you work on average? (unit: a day)

_______________________________ days [Range: 1~7]

D216. How many hours per week did you work on average including lunch time, break, etc.? (unit: an hour per week)

_______________________________ hours [Range: 1~160]

D217. Do you work based on a fixed-working hours at your current workplace?

1. Yes →Go to D220 ⑤ No

D218. How did you determine the working hours?

1. Working hours were determined given the nature of the business →Go to D220
2. Working hours were determined depending on workloads →Go to D220
3. Working hours were determined depending on sales figures →Go to D220
4. Others

D219. To be specific, what criteria did you use to determine the working hours?

_______________________________

D220. What do you think of your current working hours? Is it too long, appropriate, or too short?

1. Too long
2. Appropriate →Go to D227
3. Too short →Go to D224

D221. If you consider your working hours too long, would you like to reduce them even if your earnings are reduced?

1. Yes ⑤ No →Go to D227

D222. What % of your total working hours would you like to reduce? (unit: %)

_______________________________ % [Range: 1~99]

D223. What % cut from your total earnings are you willing to accept? (unit: %)

_______________________________ % [Range: 1~99] →Go to D227

D224. If you consider your working hours too short, are you willing to increase them if your earnings increase as a result?

1. Yes ⑤ No →Go to D227

D225. What % of your total working hours would you like to increase? (unit: %)

_______________________________ % [Range: 1~99]

D226. What % of your total earnings has to be increased? (unit: %)

_______________________________ % [Range: 1~99]

D227. Do you have regularly scheduled days off? If you do, how many days off do you have per month?

1. None
2. One day per month
3. 2~3 days per month
4. 4~5 days per month
5. 6~7 days per month
6. More than 8 days per month

**Logic**

- **If R started his/her current business before JAN 2017, ask D228.**
- **If not, go to D229.**

D228. How many days off did you have for health problems during last one year?

[IWER: Mark 0 if R didn’t miss any work day.]

_______________________________ days [Range: 0~365]

D229. How much do you agree with the following descriptions for your current job?

My job requires lots of physical effort.

1. Strongly agree
2. Agree
3. Disagree
4. Strongly disagree

D230. My job requires lifting heavy loads.

1. Strongly agree
2. Agree
3. Disagree
4. Strongly disagree

D231. My job requires stooping, kneeling, or crouching.

1. Strongly agree
2. Agree
3. Disagree
4. Strongly disagree

D232. My job requires good eyesight.

1. Strongly agree
2. Agree
3. Disagree
4. Strongly disagree

D233. My job requires intense concentration and attention to details.

1. Strongly agree
2. Agree
3. Disagree
4. Strongly disagree

D234. My job requires skills in dealing with people.

1. Strongly agree
2. Agree
3. Disagree
4. Strongly disagree

D235. My job requires me to work with computers.

1. Strongly agree
2. Agree
3. Disagree
4. Strongly disagree

D236. My job requires me to do more difficult things that it used to.

1. Strongly agree
2. Agree
3. Disagree
4. Strongly disagree

D237. I am satisfied with current wage.

1. Strongly agree
2. Agree
3. Disagree
4. Strongly disagree

D238. I feel my job is secure.

1. Strongly agree
2. Agree
3. Disagree
4. Strongly disagree

D239. I am satisfied with the work environment of my job.

1. Strongly agree
2. Agree
3. Disagree
4. Strongly disagree

D240. I am satisfied with my work at my current job.

1. Strongly agree
2. Agree
3. Disagree
4. Strongly disagree

D241. My job involves a lot of stress.

1. Strongly agree
2. Agree
3. Disagree
4. Strongly disagree

D242. In general, I am satisfied with my current job.

1. Strongly agree
2. Agree
3. Disagree
4. Strongly disagree

D243. Considering my educational background, the level of my current job is….

1. Very low
2. Low
3. Appropriate
4. High
5. Very high

D244. Considering my skills and expertise, the level of my current job is…

1. Very low
2. Low
3. Appropriate
4. High
5. Very high

D245. Would you like to continue working at your current job?

1. Yes, I would like to continue my current job, as it is →D249
2. Yes, I would like to increase work load at my current job →D249
3. Yes, I would like to transfer to add new work at my current job
4. Yes, I would like to decrease work load at my current job →D249
5. No, I would like to take a different job
6. No, I would like to quit working completely →D249

D246. What would be the ideal employment status for you?

1. Full-time wage worker
2. Part-time wage worker
3. Self-employed

D247. What do you want to do for your new job? Choose from below.

1. Managers
2. (Associate) Professionals
3. Clerical support workers
4. Service workers
5. Sales workers
6. Skilled agricultural, forestry, and fishery workers
7. Craft and related trades workers
8. Plant and machine operators, and assemblers
9. Elementary occupations

D248. What would be the minimum wage or income per month? (Unit:10,000 Korean won/average per month)

[IWER - Mark 0 if R says salary doesn’t matter.

- Please enter ‘100,000,000 Korean won (one hundred million won)’ first then, enter ’10,000 Korean won (MW)’.]

______one hundred million won [Range: 1~9] ________ MW [Range: 0 ~ 9997]

D249. Now I want to ask about your retirement plan. At what age do you plan to retire?

[IWER:

- Count a person’s age in full, not Korean age (same as American age).

- Mark 0 if R plans to keep working as long as he/she is physically capable.

- Retirement refers to having stopped income-earning activities and currently not working or engaging only in pastime work. Also, she/he doesn’t have intention to do anything more serious than pastime work as long as there is no special change in circumstances.]

_______________________________ years old [Range: 0~97]

D250. Other than the main job you have been telling us, do you currently do any other work on the side?

[IWER: Please include only paid-work as including non-paid activities such as community services]

1. Yes ⑤ No →Go to D256

D251. How many jobs do you have including your main job? (unit: number)

_______________________________ [Range: 2~7]

D252. What are the reasons for doing other works on the side than your main job? Please state the main reason only.

1. To earn more money →Go to D254
2. For my health →Go to D254
3. Because it is the work I wanted to do →Go to D254
4. For self-development →Go to D254
5. To utilize spare time →Go to D254
6. To prepare myself when I quit my main job →Go to D254
7. To help society or other people →Go to D254
8. Others

D253. If others, please specify.

_______________________________

D254. How many hours per week do you usually work on this other job/these other jobs? (unit: an hour)

_______________________________ hours [Range: 1~97]

D255. On average how much per month do you earn from this other job/these other jobs? (Unit:10,000 Korean won)

_______________________________ MW [Range: 1~9997]

D255_1. What are the reasons for you to keep working without retiring? Please choose two main reasons. First : ______

1. (Finance) Working would help resolving economic problems after retirement
2. (Emotion) I am worried that I could overcome my sense of loss after retirement
3. (Interaction) Working would help my relationships with family, friends, neighbors, and the community
4. (Social) Because I think I can continue to contribute to society by working.
5. (Health) Working would help me maintain my health
6. Others

→ From code 1~5, go to D255_1_1

D255_2. If others, please specify.

_______________________________

D255_1_1. What are the reasons for you to keep working without retiring? Please choose two main reasons. Second : ______

1. (Finance) Working would help resolving economic problems after retirement
2. (Emotion) I am worried that I could overcome my sense of loss after retirement
3. (Interaction) Working would help my relationships with family, friends, neighbors, and the community
4. (Social) Because I think I can continue to contribute to society by working.
5. (Health) Working would help me maintain my health
6. Others

→ From code 1~5, go to D255_3

D255_2_1. If others, please specify.

_______________________________

D255_3. Have you ever attended any employment programs sponsored by the government or community last year (2017)?

1. Yes ⑤ No →Go to D256

D255_4. Among the employment programs you have attended last year, what is the most recent one?

1. Elderly employment program →Go to D256
2. Public work employment program →Go to D256
3. Community employment program →Go to D256
4. Others

D255_5. If others, please specify.

_______________________________

D256. [IWER: How often did R receive assistance in answering section D-EMPLOYMENT?]

1. Never →Go to DC515.
2. A few times →Go to DC515.
3. Most or all of the time →Go to DC515.
4. The section was done by a proxy respondent.

D257. If done by a proxy respondent, what is the proxy’s relationship to R? Please answer in view of the proxy.

1. Spouse
2. Mother
3. Father
4. Mother-in-law
5. Father-in-law
6. Brother/sister
7. Brother-in-law/sister-in-law
8. Son/daughter
9. Son-in-law/Daughter-in-law
10. Grandchild
11. Other relatives
12. Helper or other non-relatives

→Go to DC515.

**Logic**

- **Ask below only if D001=①**

DC515. If the score ‘10’ is for the best state to work, how is the current state of your ability to work? Please indicate with the scale card where ‘0’ means ‘absolutely cannot work’ and ‘10’ means ‘the best state to work’.

|  |  |  |  |  |  |  |  |  |  |  |  |  |  |  |  |  |  |  |  |  |  | |
| --- | --- | --- | --- | --- | --- | --- | --- | --- | --- | --- | --- | --- | --- | --- | --- | --- | --- | --- | --- | --- | --- | --- |
|  |  |  |  |  |  |  |  |  |  |  |  |  |  |  |  |  |  |  |  |  |  | |
| 0 | | 1 | | 2 | | 3 | | 4 | | 5 | | 6 | | 7 | | 8 | | 9 | | 10 | |

DC516. As for your job tasks, how is your ability to work on physical tasks?

1. Very bad
2. Bad
3. Fair
4. Good
5. Very good

DC517. As for your job tasks, how is your ability to work on mental tasks?

1. Very bad
2. Bad
3. Fair
4. Good
5. Very good

→Go to section E (E. INCOME AND CONSUMPTION)

**<Current job : Unpaid family worker>**

**Logic**

- **If D002=③, then go to a section of unpaid family worker.**
- **Only a new unpaid family worker can answer this section.**

**Logic**

- **If D043=①(a new unpaid family worker), then go to D301~D302.**

D301. What is the most important reason for you to choose unpaid family worker instead of another paid job? Please choose the most important reason.

1. Because I can earn more money →Go to D303
2. Because it is the work I wanted to do →Go to D303
3. Because it gives me more flexibility →Go to D303
4. Because I couldn’t get the job I wanted (e.g. salary and work environment) →Go to D303
5. Because it was difficult to get a paid job →Go to D303
6. Others

D302. If others, please specify.

_______________________________

**<Ask to all unpaid family workers>**

D303. Who is the representative of your current workplace?

1. Spouse →Go to D305
2. Parents →Go to D305
3. Parents-in-law →Go to D305
4. Brother/sister →Go to D305
5. Brother-in-law/sister-in-law →Go to D305
6. Children and grandchildren →Go to D305
7. Other relatives →Go to D305
8. Others

D304. To be specific, who is the representative of your current workplace?

_______________________________

D305. How much is the current monthly sales of your business? As for agricultural and fisheries industries, please tell based on the revenue from selling agricultural and fisheries’ products including expenses. (Unit: 10,000 Korean won)

[IWER: Enter 0 if there is currently no sales]

_______________________________ MW [Range: 0~9999997] →Go to D307

Don’t know →Go to D306

Refuse to answer →Go to D306

D306. What is the estimated number of your current monthly sales figure? (Unit: MW=10,000 Korean won)

1. Less than 100 MW
2. 100~300 MW
3. 300~500 MW
4. 500~800 MW
5. 800~1,000 MW
6. 1,000~3,000 MW
7. 3,000~5,000 MW
8. 5,000~10,000 MW
9. 10,000 MW or more

D307. How many hours per week did you work on average including lunch time, break, etc.? (unit: an average hour per week)

[IWER: It is possible to enter 18 hours or more. If R says 17 hours or less, go back to D002 and mark ‘I help my family / relative’s business without pay for less than 18 hours a week’.]

_______________________________ hours / week [Range: 18~160]

D308. Do you work based on a fixed-working hours at your current workplace?

1. Yes →Go to D311 ⑤ No

D309. How did you determine the working hours?

1. Working hours were determined given the nature of the business →Go to D311
2. Working hours were determined depending on workloads →Go to D311
3. Working hours were determined depending on sales figures →Go to D311
4. Others

D310. To be specific, what criteria did you use to determine the working hours?

_______________________________

D311. How many days per week did you work on average? (unit: a day)

_______________________________ Average days / per week [Range: 1~7]

D312. What do you think of your current working hours? Is it too long, appropriate, or too short?

1. Too long
2. Appropriate →Go to D317
3. Too short →Go to D315

D313. If you consider your working hours too long, would you like to reduce them?

1. Yes ⑤ No →Go to D317

D314. What % of your total working hours would you like to reduce? (unit : %)

_______________________________% [Range: 1~99] →Go to D317

D315. If you consider your working hours too short, are you willing to increase them?

1. Yes ⑤ No →Go to D317

D316. What % of your total working hours would you like to increase? (unit : %)

_______________________________% [Range: 1~99]

D317. Do you have regularly scheduled days off? If you do, how many days off do you have per month?

1. None
2. One day per month
3. 2~3 days per month
4. 4~5 days per month
5. 6~7 days per month
6. More than 8 days per month

D318. How many days off did you have for health problems during last one year?

[IWER: Mark 0 if R didn’t miss any work day.]

_______________________________ days [Range: 0~365]

D319. How much do you agree with the following descriptions for your current job?

My job requires lots of physical effort.

1. Strongly agree
2. Agree
3. Disagree
4. Strongly disagree

D320. My job requires lifting heavy loads.

1. Strongly agree
2. Agree
3. Disagree
4. Strongly disagree

D321. My job requires stooping, kneeling, or crouching.

1. Strongly agree
2. Agree
3. Disagree
4. Strongly disagree

D322. My job requires good eyesight.

1. Strongly agree
2. Agree
3. Disagree
4. Strongly disagree

D323. My job requires intense concentration and attention to details.

1. Strongly agree
2. Agree
3. Disagree
4. Strongly disagree

D324. My job requires skills in dealing with people.

1. Strongly agree
2. Agree
3. Disagree
4. Strongly disagree

D325. My job requires me to work with computers.

1. Strongly agree
2. Agree
3. Disagree
4. Strongly disagree

D326. My job requires me to do more difficult things that it used to.

1. Strongly agree
2. Agree
3. Disagree
4. Strongly disagree

D327. I am satisfied with the work environment of my job.

1. Strongly agree
2. Agree
3. Disagree
4. Strongly disagree

D328. I am satisfied with my work at my current job.

1. Strongly agree
2. Agree
3. Disagree
4. Strongly disagree

D329. My job involves a lot of stress.

1. Strongly agree
2. Agree
3. Disagree
4. Strongly disagree

D330. In general, I am satisfied with my current job.

1. Strongly agree
2. Agree
3. Disagree
4. Strongly disagree

D331. How helpful do you think your working as an unpaid family worker is to your household income?

1. Very helpful
2. Somewhat helpful
3. Fair
4. Not helpful
5. Not at all helpful

D332. Considering my educational background, the level of my current job is….

1. Very low
2. Low
3. Appropriate
4. High
5. Very high

D333. Considering my skills and expertise, the level of my current job is…

1. Very low
2. Low
3. Appropriate
4. High
5. Very high

D334. Would you like to continue working at your current job?

1. Yes, I would like to continue my current job, as it is →D338
2. Yes, I would like to increase work load at my current job →D338
3. Yes, I would like to transfer to add new work at my current job
4. Yes, I would like to decrease work load at my current job →D338
5. No, I would like to take a different job
6. No, I would like to quit working completely →D338

Don’t know →Go to D338

Refuse to answer →Go to D338

D335. What would be the ideal employment status for your new job?

① Full time wage worker

③ Part time wage worker

⑤ Self-employed

D336. What do you want to do for your new job? Choose from below.

1. Managers
2. (Associate) Professionals
3. Clerical support workers
4. Service workers
5. Sales workers
6. Skilled agricultural, forestry, and fishery workers
7. Craft and related trades workers
8. Plant and machine operators, and assemblers
9. Elementary occupations

D337. What would be the minimum wage or income per month for this job?

[IWER - Mark 0 if R says salary doesn’t matter.

- Please enter ‘100,000,000 Korean won (one hundred million won)’ first then, enter ’10,000 Korean won (MW)’.]

______one hundred million won [Range: 1~9] ________ MW [Range: 0 ~ 9997]

D338. Now I want to ask about your retirement plan. At what age do you plan to retire?

[IWER:

- Count a person’s age in full, not Korean age (same as American age).

- Mark 0 if R plans to keep working as long as he/she is physically capable.

- Retirement refers to having stopped income-earning activities and currently not working or engaging only in pastime work. Also, she/he doesn’t have intention to do anything more serious than pastime work as long as there is no special change in circumstances.]

_______________________________ years old [Range: 0~97]

D339. Other than the main job you have been telling us, do you currently do any other work on the side?

[IWER: Please include only paid-work as including non-paid activities such as community services]

1. Yes ⑤ No →Go to D344_1

D340. How many jobs do you have including your main job? Please include paid jobs only. (unit: number)

_______________________________ [Range: 2~7]

D341. What are the reasons for doing other works on the side than your main job?.

1. To earn more money →Go to D343
2. For my health →Go to D343
3. Because it is the work I wanted to do →Go to D343
4. For self-development →Go to D343
5. To utilize spare time →Go to D343
6. To prepare myself when I quit my main job →Go to D343
7. To help society or other people →Go to D343
8. Others

D342. If others, please specify.

_______________________________

D343. How many hours a week do you usually work on this other job / these other jobs? (unit: an average hour per week)

_______________________________ hours / week [Range: 1~97]

D344. How much do you earn per month on average from this other job / these other jobs? (Unit: 10,000 Korean won)

_______________________________ MW [Range: 1~9997]

D344_1. What are the reasons for you to keep working without retiring? Please choose two main reasons. First : _________

1. (Finance) Working would help resolving economic problems after retirement
2. (Emotion) I am worried that I could overcome my sense of loss after retirement
3. (Interaction) Working would help my relationships with family, friends, neighbors, and the community
4. (Social) Because I think I can continue to contribute to society by working.
5. (Health) Working would help me maintain my health
6. Others

→ From code 1~5, go to D344_1_1

D344_2. If others, please specify.

_______________________________

D344_1_1. What are the reasons for you to keep working without retiring? Please choose two main reasons. Second : _________

1. (Finance) Working would help resolving economic problems after retirement
2. (Emotion) I am worried that I could overcome my sense of loss after retirement
3. (Interaction) Working would help my relationships with family, friends, neighbors, and the community
4. (Social) Because I think I can continue to contribute to society by working.
5. (Health) Working would help me maintain my health
6. Others

→ From code 1~5, go to D344_3

D344_2_1. If others, please specify.

_______________________________

D344_3. Have you ever attended any employment programs sponsored by the government or community last year (2017)?

1. Yes ⑤ No →Go to D345

D344_4. Among the employment programs you have attended last year, what is the most recent one?

1. Elderly employment program →Go to D345
2. Public work employment program →Go to D345
3. Community employment program →Go to D345
4. Others

D344_5. If others, please specify.

_______________________________

D345. [IWER: How often did R receive assistance in answering section D-EMPLOYMENT?]

1. Never →Go to DC515.
2. A few times →Go to DC515.
3. Most or all of the time →Go to DC515.
4. The section was done by a proxy respondent.

D346. If done by a proxy respondent, what is the proxy’s relationship to R? Please answer in view of the proxy.

1. Spouse
2. Mother
3. Father
4. Mother-in-law
5. Father-in-law
6. Brother/sister
7. Brother-in-law/sister-in-law
8. Son/daughter
9. Son-in-law/Daughter-in-law
10. Grandchild
11. Other relatives
12. Helper or other non-relatives

→Go to DC515.

**Logic**

- **Ask below only if D001=①**

DC515. If the score ‘10’ is for the best state to work, how is the current state of your ability to work? Please indicate with the scale card where ‘0’ means ‘absolutely cannot work’ and ‘10’ means ‘the best state to work’.

|  |  |  |  |  |  |  |  |  |  |  |  |  |  |  |  |  |  |  |  |  |  | |
| --- | --- | --- | --- | --- | --- | --- | --- | --- | --- | --- | --- | --- | --- | --- | --- | --- | --- | --- | --- | --- | --- | --- |
|  |  |  |  |  |  |  |  |  |  |  |  |  |  |  |  |  |  |  |  |  |  | |
| 0 | | 1 | | 2 | | 3 | | 4 | | 5 | | 6 | | 7 | | 8 | | 9 | | 10 | |

DC516. As for your job tasks, how is your ability to work on physical tasks?

1. Very bad
2. Bad
3. Fair
4. Good
5. Very good

DC517. As for your job tasks, how is your ability to work on mental tasks?

1. Very bad
2. Bad
3. Fair
4. Good
5. Very good

→Go to section E (E. INCOME AND CONSUMPTION)

**<Current job: a job seeker/an unemployed person>**

**Logic**

- **If R has answered D006, ask D401.**

D401. Did you look for a job or other work to earn money in the past week?

1. Yes →Go to D403 ⑤ No

D402. Did you look for a job or other work to earn money in the past four weeks?

1. Yes ⑤ No →Go to D404

D403. Would you have been able to work last week if there had been an appropriate job or work?

1. Yes →Go to D410 ⑤ No →Go to D405

D404. Are you willing to work if there is an appropriate job?

1. Yes →Go to D407 ⑤ No

**Logic: After R has answered D405, only except D405=⑧)**

- **If D006=①, ③, then go to D601~D613**
- **If D006=⑤, then go to D501~D510**
- **If D006=⑦, ⑨, ⑪, then go to D422**

D405. What is the main reason you were not willing to work even if there is a job? Choose the main reason only.

1. Family responsibility/ Child responsibility
2. Household chores
3. No financial need/ Affordable
4. Too old
5. Poor health/ Disability
6. Wish to take a break
7. (After retirement/quitting a job) Do not want to work
8. Others

D406. If others, please specify.

_______________________________

D407. What is the main reason you were not looking for a job although you want a job?

1. I believe there would be no job that matches my major or professional background →Go to D409
2. I believe there would be no job that meets my desired wage level or working conditions →Go to D409
3. I don’t believe I can find work →Go to D409
4. I don’t have needed skills, education or other capabilities →Go to D409
5. I need to care for a family member →Go to D409
6. Household chores →Go to D409
7. Poor health or disability →Go to D409
8. Others

D408. If others, please specify.

_______________________________

D409. Then, have you ever looked for a job to earn money prior to the past four weeks?

1. Yes →Go to D409-1 ⑤ No →Go to D422

D409-1. How many times did you look for a job after the previous interview?

_______________________________(times)

D410. What is the main reason you are looking for a job?

1. To earn a living expenses →Go to D412
2. Because my spouse/ family member lost job →Go to D412
3. To earn pocket money →Go to D412
4. For self‐development →Go to D412
5. To utilize spare time →Go to D412
6. Others

D411. If others, please specify.

_______________________________

**Logic**

- **If R answered ‘one time’ at D409-1, then ask only D412 (no need to ask D412-1)**
- **If R answered more than 2 times, then ask D412-1.**

D412. When did you start looking for a job after the previous interview? (unit: year and month combined into 6 digits)

[IWER: Enter the year and month using 6 digits. For example, mark 201701 for January 2017. If the month is not clear, enter 201700.]

_______________________________ [Range: 200600~201811]→Go to D413 or D414

D412-1. When did you start looking for a job most recently? (unit: year and month combined into 6 digits)

[IWER: Enter the year and month using 6 digits. For example, mark 201701 for January 2017. If the month is not clear, enter 201700.]

_______________________________ [Range: 200600~201811]

**Logic**

- **If D409=①, then ask D413.**
- **D413 is to check when R stopped looking for a job for D412-1.**

D413. When did you stop looking for a job? (unit: year and month combined into 6 digits)

[IWER: Enter the year and month using 6 digits. For example, mark 201701 for January 2017. If the month is not clear, enter 201700.]

_______________________________ [Range: 200600~201811]

D414. On average how many hours per week did you spend on looking for a job when you were engaged in job search activities? (unit: an average hour per week)

[IWER: Mark 1 for less than 1 hour.]

_______________________________hours per week [Range: 1~160]

D415. How did you look for a job when you were engaged in job search activities? Choose the two main activities that you have done mostly.

What is the activity you were most engaged in?

1. Search through a public employment agency (Job Center of Ministry of Employment and Labor, Human Resources Bank of the aged, Senior Biz Plaza, Function Senior Club etc.) →Go to D417
2. Search through a private employment agency →Go to D417
3. Search through advertisements in newspapers, TV and billboards →Go to D417
4. Search through the Internet →Go to D417
5. Search through friends, family members, or relatives →Go to D417
6. Others

D416. If others, please specify.

_______________________________

D417. Then, what is the second activity that you were engaged in?

1. Search through a public employment agency(Job Center of Ministry of Employment and Labor, Human Resources Bank of the aged, Senior Biz Plaza, Function Senior Club etc.) →Go to D419
2. Search through a private employment agency →Go to D419
3. Search through advertisements in newspapers, TV and billboards →Go to D419
4. Search through the Internet →Go to D419
5. Search through friends, family members, or relatives →Go to D419
6. Others

D418. If others, please specify.

_______________________________

**Logic**

- **If D419=0, then go to D422.**

D419. Did you find a job or were you offered a job (workplace, business, work, etc.) when you were engaged in job search activities? If so, how many times? (unit: a time)

[IWER: Mark 0 if there is none.]

_______________________________ [Range: 0~97]

D420. What is the reason that you did not take the job you found or were offered?

1. Was not hired
2. Wage and working conditions didn’t meet my expectation
3. Expertise, experience, skills, and aptitude did not match well
4. Seems physically demanding
5. Poor health/ disability
6. Household chores, childcare, etc.
7. Others

D421.If others, please specify.

_______________________________

D422. You described your work status as [D006] at the beginning of this section. If so, what would be the ideal employment status for you?

| **[IWER]**   - **Part-time**: Working on a part-time basis or as a side job, workers who work less hours than others in the same job, or get paid on hourly basis - **Full-time**: Working on a full-time basis, workers who work full-working days and full-hours, or not get paid on hourly basis |
| --- |

① Full time wage worker →Go to D425

③ Part time wage worker

⑤ Self-employed →Go to D425

D423. Why do you want a part time job? Please state the main reason only.

1. Because I would like to do household core, childcare as well
2. Because I don’t think I can find a full time job
3. Poor health/ disability
4. Because I just don’t want to work long hours (full time)
5. Others

D424. If others, please specify.

_______________________________

D425. Which workplace do you want to work in? Please state the industry that you would like to work in.

1. Agriculture/forestry
2. Fishing
3. Mining and quarrying
4. Manufacturing
5. Electricity, gas, and water supply
6. Construction
7. Wholesale and retail trade
8. Hotels and restaurants
9. Transport
10. Post and telecommunications
11. Financial and insurance activities
12. Real estate and renting and leasing
13. Other activities

D426. To be specific, what kind of work do you want to do in the workplace?

1. Managers
2. (Associate) Professionals
3. Clerical support workers
4. Service workers
5. Sales workers
6. Skilled agricultural, forestry, and fishery workers
7. Craft and related trades workers
8. Plant and machine operators, and assemblers
9. Elementary occupations

D427. If someone offered you a regular job now, how high would the wages or salaries have to be for you to take it? Please state the monthly income. (unit: 10,000 Korean Won / month)

[IWER - Mark 0 if R says salary doesn’t matter.

- Please enter ‘100,000,000 Korean won (one hundred million won)’ first then, enter ’10,000 Korean won (MW)’.]

______one hundred million won [Range: 1~9] ________ MW [Range: 0 ~ 9997]

D428. The following is common difficulties in finding jobs. How much do you agree with each of difficulties?

There is a shortage of jobs.

1. Strongly agree
2. Agree
3. Fair
4. Disagree
5. Strongly disagree

D429. There is not enough recruitment information around.

1. Strongly agree
2. Agree
3. Fair
4. Disagree
5. Strongly disagree

D430. I don’t have needed education, skills or expertise.

1. Strongly agree
2. Agree
3. Fair
4. Disagree
5. Strongly disagree

D431. I don’t have needed experience.

1. Strongly agree
2. Agree
3. Fair
4. Disagree
5. Strongly disagree

D432. The wage offered is too low.

1. Strongly agree
2. Agree
3. Fair
4. Disagree
5. Strongly disagree

D433. The working environment and hours are not suitable.

1. Strongly agree
2. Agree
3. Fair
4. Disagree
5. Strongly disagree

D434. Old age makes employment difficult.

1. Strongly agree
2. Agree
3. Fair
4. Disagree
5. Strongly disagree

**Logic**

- **If R is female, then ask D435.**
- **After D435, if D006=①, ③, then go to D601~D613.**
- **After D435, if D006=⑤, then go to D501~D510.**

D435. Being a woman makes employment difficult.

1. Strongly agree
2. Agree
3. Fair
4. Disagree
5. Strongly disagree

**Logic**

- **If D006=⑦, ⑨ or ⑪, then go to D436.**

D436. Now I want to ask about your retirement plan. At what age do you plan to retire?

[IWER:

- Count a person’s age in full, not Korean age (same as American age).

- Mark 0 if R plans to keep working as long as he/she is physically capable.

- Retirement refers to having stopped income-earning activities and currently not working or engaging only in pastime work. Also, she/he doesn’t have intention to do anything more serious than pastime work as long as there is no special change in circumstances.]

_______________________________ years old [Range: 0~97]

D436_1. Have you ever attended any employment programs sponsored by the government or community last year (2017)?

1. Yes ⑤ No →Go to D437

D436_2. Among the employment programs you have attended last year, what is the most recent one?

1. Elderly employment program →Go to D437
2. Public work employment program →Go to D437
3. Community employment program →Go to D437
4. Others

D436_3. If others, please specify.

_______________________________

D437. [IWER: How often did R receive assistance in answering section D-EMPLOYMENT?]

1. Never →Go to section Ea.
2. A few times →Go to section Ea.
3. Most or all of the time →Go to section Ea.
4. The section was done by a proxy respondent.

D438. If done by a proxy respondent, what is the proxy’s relationship to R? Please answer in view of the proxy.

1. Spouse
2. Mother
3. Father
4. Mother-in-law
5. Father-in-law
6. Brother/sister
7. Brother-in-law/sister-in-law
8. Son/daughter
9. Son-in-law/Daughter-in-law
10. Grandchild
11. Other relatives
12. Helper or other non-relatives

→Go to section E.

**< Retiree >**

**Logic**

- **If D006=⑤, then go to a section of retiree.**

**<Only for a new retiree>**

**Logic**

- **If D036=⑤ and D043=⑤(a new retiree) or D084=⑤, then go to D501~D508.**

D501. When did you retire after the previous interview? (unit: year and month combined into 6 digits)

[IWER: Enter the year and month using 6 digits. For example, mark 201701 for January 2017. If the month is not clear, enter 201700.]

_______________________________ [Range: 200600 ~ 201811]

D502. What were the most important reasons you retired? Choose the two main reasons.

What is the most important reason you retired?

1. Having enough income to get by →Go to D504
2. Having enough income from spouse →Go to D504
3. Didn’t like to continue to work →Go to D504
4. To spend more time on leisure →Go to D504
5. To do volunteer work or to pursue hobby →Go to D504
6. Poor health/ disability →Go to D504
7. Poor health of spouse →Go to D504
8. Poor health of other family members →Go to D504
9. Household chore or childcare →Go to D504
10. Could not find another job →Go to D504
11. Mandatory retirement →Go to D504
12. Others

D503. If others, please specify.

_______________________________

D504. What was the second most important reason you retired?

1. Having enough income to get by →Go to D506
2. Having enough income from spouse →Go to D506
3. Did not like to continue to work →Go to D506
4. To spend more time on leisure →Go to D506
5. To do volunteer work or to pursue hobby →Go to D506
6. Poor health/ disability →Go to D506
7. Poor health of spouse →Go to D506
8. Poor health of other family members →Go to D506
9. Household chore or childcare →Go to D506
10. Could not find another job →Go to D506
11. Others

D505. If others, please specify.

_______________________________

D506. Did you have a spouse when you retired?

1. Yes ⑤ No →Go to D509

D507. What was your spouse’s economic status at the time of your retirement?

1. Employed and paid salary or wage →Go to D509
2. Running his/her own business →Go to D509
3. Helping family or relative’s business without wage for more than 18 hours a week →Go to D509
4. Helping family or relative’s business without pay for less than 18 hours a week →Go to D509
5. Unemployed and currently looking for a job →Go to D509
6. Unemployed and not looking for a job →Go to D509
7. Retired →Go to D509
8. Others

D508. If others, please specify.

_______________________________

**< Ask to all retirees >**

D509. All in all, would you say that your retirement has turned out to be very satisfying, moderately satisfying, or not at all satisfying?

1. Very satisfying
2. Satisfying
3. Not at all satisfying

D510. Thinking about your retirement years compared to the years just before you retired, would you say the retirement years have been better, about the same, or not as good?

1. Better →Go to D601
2. About the same →Go to D601
3. Not as good →Go to D601

**<About second job >**

**Logic**

- **If D006=①, ③ or ⑤, then go to a section of** second job**.**

D601. Are you currently engaged in a paid second job?

1. Yes ⑤ No →Go to D607

D602. Aside from your main job, you have responded that you have another second job. What kind of work are you doing?

1. Agriculture, forestry, and fishery →Go to D603
2. Pick up recycled or scraped items to sell →Go to D603
3. Delivery service (silver delivery service) →Go to D603
4. Simple manufacturing activities →Go to D603
5. Others

D602-1. If others, please specify.

_______________________________

D603. When did you start having that work? (unit: year and month combined into 6 digits)

[IWER: Enter the year and month using 6 digits. For example, mark 201701 for January 2017. If the month is not clear, enter 201700.]

_______________________________ [Range: 190000~201811]

D604. How many days per week do you usually work on second job? (unit: a day)

_______________________________ days per week [Range: 1~7]

D605. How many hours per week do you usually work on second job? (unit: an hour)

_______________________________ hours per week [Range: 1~97]

D606. How much do you earn from your second job?

[IWER - Mark 0 if there is no net income, and mark 99997 if R is running a deficit.

- Please enter ‘100,000,000 Korean won (one hundred million won)’ first then, enter ’10,000 Korean won (MW)’.]

______one hundred million won [Range: 1~9] ________ MW [Range: 0 ~ 9997]

D607. Do you want to work for pay regardless of retirement status?

1. Yes ⑤ No →Go to D611_1

D608. What is the reason?

1. I just want to work as long as my health allows →Go to D610
2. I need/ want extra money for living expenses →Go to D610
3. Society still needs my knowledge/ expertise →Go to D610
4. To maintain healthy physical and mental condition →Go to D610
5. Bored at home →Go to D610
6. Others

D609. If others, please specify.

_______________________________

D610. If that is the case, what sort of work do you want to have?

1. Small job regardless of income →Go to D612
2. Part time work for some pocket money →Go to D612
3. Full time work for living expenses →Go to D612
4. Self‐employed (business start‐up) →Go to D612
5. Others

D611. If others, please specify.

_______________________________

D611_1. Have you ever attended any employment programs sponsored by the government or community last year (2017)?

1. Yes ⑤ No →Go to D612

D611_2. Among the employment programs you have attended last year, what is the most recent one?

1. Elderly employment program →Go to D612
2. Public work employment program →Go to D612
3. Community employment program →Go to D612
4. Others

D611_3. If others, please specify.

_______________________________

D612. [IWER: How often did R receive assistance in answering section D-EMPLOYMENT?]

1. Never →Go to section Ea.
2. A few times →Go to section Ea.
3. Most or all of the time →Go to section Ea.
4. The section was done by a proxy respondent.

D613. If done by a proxy respondent, what is the proxy’s relationship to R? Please answer in view of the proxy.

1. Spouse
2. Mother
3. Father
4. Mother-in-law
5. Father-in-law
6. Brother/sister
7. Brother-in-law/sister-in-law
8. Son/daughter
9. Son-in-law/Daughter-in-law
10. Grandchild
11. Other relatives
12. Helper or other non-relatives

→Go to section Ea.

(D_end) [IWER] Please enter code 1 for completion of D. EMPLOYMENT section.

1. Completion

# **E. INCOME AND CONSUMPTION**

(E_Intro) [IWER] Now, this is to start E. Income and Consumption section.

1. Check

**<Wages or Salaries income>**

E001. Did you earn any income by being employed by another person or in a workplace in the last calendar year (2017)?

1. Yes ⑤ No →Go to E010

E002. How many month(s) did you get paid in the last calendar year (2017)? (unit: month)

_______________________________ months [Range:0~12]

E003. What was your average monthly wages or salaries in the last calendar year (2017) including various costs or taxes? (unit: 10,000 Korean won)

[IWER:

- In previous interview, an average monthly wages or salaries income: About (PL) 10,000 Korean won

- Please enter ‘100,000,000 Korean won (one hundred million won)’ first then, enter ’10,000 Korean won (MW)’.]

______one hundred million won [Range: 1~9] ________ MW [Range: 0 ~ 9997]

Don’t know →Go to E005~E009 unfolding

Refuse to answer →Go to E005~E009 unfolding

E004. So you received a total of (amount calculated based on E002 and E003) in the last calendar year (2017). Is this correct?

[IWER: In previous interview, the total of wages or salaries income: About (PL) 10,000 Korean won]

1. Yes →Go to E010 ⑤ No →Go to E005~E009 unfolding

Don’t know →Go to E005~E009 unfolding

Refuse to answer →Go to E005~E009 unfolding

**< E005~E009. Unfolding bracket questions >**

[IWER: Go back to E003 and record if R answers the amount during the interview.]

E005. Did it amount to a total of less than, about equal to or more than 600 MW (10,000 Korean won) in the last calendar year (2017)?

① Less than 600 MW

③ About 600 MW

⑤ More than 600 MW

E006. Did it amount to a total of less than, about equal to or more than 1,200 MW (10,000 Korean won)? in the last calendar year (2017)

① Less than 1,200 MW

③ About 1,200 MW

⑤ More than 1,200 MW

E007. Did it amount to a total of less than, about equal to or more than 2,400 MW (10,000 Korean won) in the last calendar year (2017)?

① Less than 2,400 MW

③ About 2,400 MW

⑤ More than 2,400 MW

E008. Did it amount to a total of less than, about equal to or more than 6,000 MW (10,000 Korean won) in the last calendar year (2017)?

① Less than 6,000 MW

③ About 6,000 MW

⑤ More than 6,000 MW

E009. Did it amount to a total of less than, about equal to, or more than 12,000 MW (10,000 Korean won) in the last calendar year (2017)?

① Less than 12,000 MW

③ About 12,000 MW

⑤ More than 12,000 MW

**< Business owner/ Self-employment Income >**

E010. Did you earn any income from self-employment or running your own business in the last calendar year (2017)? Income from agricultural and fisheries business is excluded.

1. Yes ⑤ No →Go to E019

E011. How many month(s) did you earn income from self-employment or running your own business in the last calendar year (2017)? (unit: month)

_______________________________ months [Range:1~12]

E012. What was your average monthly net income in the last calendar year (2017) after taxes and other deductions? (unit: 10,000 Korean won)

[IWER:

- In previous interview, an average monthly net income: About (PL) 10,000 Korean won

- Please enter ‘100,000,000 Korean won (one hundred million won)’ first then, enter ’10,000 Korean won (MW)’.]

______one hundred million won [Range: 1~9] ________ MW [Range: 0 ~ 9997]

Don’t know →Go to E014~E018 unfolding

Refuse to answer →Go to E014 ~ E018 unfolding

E013. So you earned a total of (amount calculated based on E011 and E012) in the last calendar year (2017). Is this correct?

[IWER: In previous interview, the total of income: About (PL) 10,000 Korean won]

1. Yes →Go to E019 ⑤ No →Go to E014~E018 unfolding

Don’t know →Go to E014~E018 unfolding

Refuse to answer →Go to E014~E018 unfolding

**< E014~E018. Unfolding bracket questions >**

[IWER: Go back to E012 and record if R answers the amount during the interview.]

E014. Did it amount to a total of less than, about equal to or more than 600 MW (10,000 Korean won) in the last calendar year (2017)?

① Less than 600 MW

③ About 600 MW

⑤ More than 600 MW

E015. Did it amount to a total of less than, about equal to or more than 1,200 MW (10,000 Korean won) in the last calendar year (2017)?

① Less than 1,200 MW

③ About 1,200 MW

⑤ More than 1,200 MW

E016. Did it amount to a total of less than, about equal to or more than 2,400 MW (10,000 Korean won) in the last calendar year (2017)?

① Less than 2,400 MW

③ About 2,400 MW

⑤ More than 2,400 MW

E017. Did it amount to a total of less than, about equal to or more than 6,000 MW (10,000 Korean won) in the last calendar year (2017)?

① Less than 6,000 MW

③ About 6,000 MW

⑤ More than 6,000 MW

E018. Did it amount to a total of less than, about equal to, or more than 12,000 MW (10,000 Korean won) in the last calendar year (2017)?

1. Less than 12,000 MW
2. About 12,000 MW
3. More than 12,000 MW

**< Agriculture and fishery income >**

E019. Did you earn any income from agricultural and fisheries business in the last calendar year (2017)?

1. Yes ⑤ No →Go to E026

E020. How much did you earn from agricultural and fisheries business in the last calendar year (2017)? (unit: 10,000 Korean won)

[IWER: “Please state net income by subtracting all the expenses and taxes from total revenue.” (Wage worker in the agricultural and fisheries industries are excluded.) In previous interview, agriculture and fishery income: About (PL) 10,000 Korean won

- Please enter ‘100,000,000 Korean won (one hundred million won)’ first then, enter ’10,000 Korean won (MW)’.]

______one hundred million won [Range: 1~9] ________ MW [Range: 0 ~ 9997] → E026

Don’t know →Go to E021~E025 unfolding

Refuse to answer →Go to E021~E025 unfolding

**<E021~E025. Unfolding bracket questions >**

[IWER: Go back to E020 and record if R answers the amount during the interview.]

E021. Did it amount to a total of less than, about equal to or more than 600 MW (10,000 Korean won) in the last calendar year (2017)?

① Less than 600 MW

③ About 600 MW

⑤ More than 600 MW

E022. Did it amount to a total of less than, about equal to, or more than 1,200 MW (10,000 Korean won) in the last calendar year (2017)?

① Less than 1,200 MW

③ About 1,200 MW

⑤ More than 1,200 MW

E023. Did it amount to a total of less than, about equal to or more than 2,400 MW (10,000 Korean won) in the last calendar year (2017)?

① Less than 2,400 MW

③ About 2,400 MW

⑤ More than 2,400 MW

E024. Did it amount to a total of less than, about equal to or more than 6,000 MW (10,000 Korean won) in the last calendar year (2017)?

① Less than 6,000 MW

③ About 6,000 MW

⑤ More than 6,000 MW

E025. Did it amount to a total of less than, about equal to or more than 12,000 MW (10,000 Korean won) in the last calendar year (2017)?

① Less than 12,000 MW

③ About 12,000 MW

⑤ More than 12,000 MW

**< Second job >**

E026. Did you earn any income from any second job in the last calendar year (2017)?

1. Yes ⑤ No →Go to E033

Don’t know →Go to E028~E032 unfolding

Refuse to answer →Go to E028~E032 unfolding

E027. How much did you earn from your second job in the last calendar year (2017)? Please state the amount of income after taxes and other deductions. (unit: 10,000 Korean Won)

[IWER:

- In previous interview, second job income: About (PL) 10,000 Korean won]

- Please enter ‘100,000,000 Korean won (one hundred million won)’ first then, enter ’10,000 Korean won (MW)’.

______one hundred million won [Range: 1~9] ________ MW [Range: 0 ~ 9997] → E033

Don’t know →Go to E028~E032 unfolding

Refuse to answer →Go to E028~E032 unfolding

**< E028~E032. Unfolding bracket questions >**

[IWER: Go back to E027 and record if R answers the amount during the interview.]

E028. Did it amount to a total of less than, about equal to or more than 300 MW (10,000 Korean won) in the last calendar year (2017)?

① Less than 300 MW

③ About 300 MW

⑤ More than 300 MW

E029. Did it amount to a total of less than, about equal to or more than 600 MW (10,000 Korean won) in the last calendar year (2017)?

① Less than 600 MW

③ About 600 MW

⑤ More than 600 MW

E030. Did it amount to a total of less than, about equal to or more than 1,200 MW (10,000 Korean won) in the last calendar year (2017)?

① Less than 1,200 MW

③ About 1,200 MW

⑤ More than 1,200 MW

E031. Did it amount to a total of less than, about equal to or more than 2,400 MW (10,000 Korean won) in the last calendar year (2017)?

① Less than 2,400 MW

③ About 2,400 MW

⑤ More than 2,400 MW

E032. Did it amount to a total of less than, about equal to or more than 6,000 MW (10,000 Korean won) in the last calendar year (2017)?

① Less than 6,000 MW

③ About 6,000 MW

⑤ More than 6,000 MW

**<Income from the National Pension Benefit>**

E033. Did you receive any benefits from the National Pension in the last calendar year (2017)? If yes, did you receive your benefits monthly or in a lump-sum? National pensions include old-age pension, disability pension, survivor pension, and lump-sum refund/ death payments.

1. Only received monthly benefits
2. Only received lump-sum benefits →Go to E036
3. Received both
4. No →Go to E044

E034. How many month(s) did you receive your monthly national pension benefits in the last calendar year (2017)? (unit: month)

_______________________________ months [Range:0~12]

E035. What was the average monthly amount of national pension benefits in the last calendar year (2017)? (unit: 10,000 Korean won)

[IWER: In previous interview, an average monthly national pension benefit: About (PL) 10,000 Korean won]

_______________________________ MW [Range: 0 ~ 9997]

Don’t know →Go to E038~E042 unfolding

Refuse to answer →Go to E038~E042 unfolding

**Logic**

- **If E033=①, then ask E037.**
- **If E033=② or ③, then ask E036.**

E036. What was the total amount of national pension benefits you received for a lump sum in the last calendar year (2017)? (unit:10,000 Korean won)

[IWER:

- In previous interview, the total of your lump sum benefit from the national pension: About (PL) 10,000 Korean won

- Please enter ‘100,000,000 Korean won (one hundred million won)’ first then, enter ’10,000 Korean won (MW)’.]

______one hundred million won [Range: 1~999] ________MW [Range: 0 ~ 9997]

Don’t know→Go to E038~E042 unfolding

Refuse to answer →Go to E038~E042 unfolding

E037. So you received a total of MW (calculated based onE034, E035, and E036) from the National Pension System in the last calendar year (2017). Is this correct?

[IWER: In previous interview, the total of national pension benefit: About (PL) 10,000 Korean won]

1. Yes →Go to E043 ⑤ No →Go to E038~E042 unfolding

Don’t know →Go to E038~E042 unfolding

Refuse to answer →Go to E038~E042 unfolding

**< E038~E042. Unfolding bracket questions >**

[IWER: Go back to E036 and record if R answers the amount during the interview.]

E038. Did it amount to a total of less than, about equal to or more than 120 MW (10,000Korean won) in the last calendar year (2017)?

① Less than 120 MW

③ About 120 MW

⑤ More than 120 MW

E039. Did it amount to a total of less than, about equal to or more than 240 MW (10,000Korean won) in the last calendar year (2017)?

① Less than 240 MW

③ About 240 MW

⑤ More than 240 MW

E040. Did it amount to a total of less than, about equal to or more than 480 MW (10,000Korean won) in the last calendar year (2017)?

① Less than 480 MW

③ About 480 MW

⑤ More than 480 MW

E041. Did it amount to a total of less than, about equal to or more than 600 MW (10,000Korean won) in the last calendar year (2017)?

① Less than 600 MW

③ About 600 MW

⑤ More than 600 MW

E042. Did it amount to a total of less than, about equal to, or more than 1,200 MW (10,000Korean won) in the last calendar year (2017)?

① Less than 1,200 MW

③ About 1,200 MW

⑤ More than 1,200 MW

E043. When did you start receiving the national pension benefits? (unit: year and month combined into 6 digits)

[IWER: Enter the year and month using 6 digits. For example, mark 201701 for January 2017. If the month is not clear, enter 201700.. In previous interview, the time of receiving national pension benefit: (PL) year (PL) month]

_______________________________ [Range: 198800~201712]

**< Income from the Specific Corporate Pension Benefit>**

E044. Did you receive any benefits from the specific corporate pension in the last calendar year (2017)? If yes, did you receive your benefits monthly or in a lump-sum? Specific corporate pensions includes private school teachers pension, government employees pension, military personnel pension and specific post office pension. If yes, did you receive your benefits monthly or in a lump sum?

1. Only received monthly benefits
2. Only received lump-sum benefits →Go to E047
3. Received both
4. No →Go to E055

E045. How many month(s) did you receive your specific corporate pension benefits in the last calendar year (2017)? (unit: month)

_______________________________ months [Range:0~12]

E046. What was the average monthly amount of specific corporate pension benefits in the last calendar year (2017)? (unit: 10,000 Korean won)

[IWER: In previous interview, an average monthly specific corporate pension benefit: About (PL) 10,000 Korean won]

_______________________________ MW [Range: 1 ~ 9997] →Go to E047

Don’t know→Go to E049∼E053 unfolding

Refuse to answer →Go to E049∼E053 unfolding

**Logic**

- **If E044=①, then ask E048.**
- **If E044=② or ③, then ask E047.**

E047. What was the total amount of specific corporate pension benefits you received for a lump sum in the last calendar year (2017)? (unit: 10,000 Korean won)

[IWER:

In previous interview, the total of specific corporate pension benefit (a lump sum): About (PL) 10,000 Korean won.

- Please enter ‘100,000,000 Korean won (one hundred million won)’ first then, enter ’10,000 Korean won (MW)’.]

______one hundred million won [Range: 1~9] _________ MW [Range: 0 ~ 9997]

Don’t know→Go to E049∼E053 unfolding

Refuse to answer →Go to E049∼E053 unfolding

E048. So you received a total of (amount calculated based on E045, E046, and E047) from the specific corporate pension in the last calendar year (2017). Is this correct?

[IWER: In previous interview, the total of specific corporate pension benefit: About (PL) 10,000 Korean won]

1. Yes →Go to E054 ⑤ No →Go to E049∼E053 unfolding

Don’t know→Go to E049~E053 unfolding

Refuse to answer →Go to E049~E053 unfolding

**< E049~E053. Unfolding bracket questions >**

[IWER: Go back to E047 and record if R answers the amount during the interview.]

E049. Did it amount to a total of less than, about equal to or more than 300 MW (10,000 Korean won)in the last calendar year (2017)?

① Less than 300 MW

③ About 300 MW

⑤ More than 300 MW

E050. Did it amount to a total of less than, about equal to, or more than 600 MW (10,000 Korean won) in the last calendar year (2017)?

① Less than 600 MW

③ About 600 MW

⑤ More than 600 MW

E051. Did it amount to a total of less than, about equal to or more than 1,200 MW (10,000 Korean won) in the last calendar year (2017)?

① Less than 1,200 MW

③ About 1,200 MW

⑤ More than 1,200 MW

E052. Did it amount to a total of less than, about equal to or more than 2,400 MW (10,000 Korean won) in the last calendar year (2017)?

① Less than 2,400 MW

③ About 2,400 MW

⑤ More than 2,400 MW

E053. Did it amount to a total of less than, about equal to or more than 6,000 MW (10,000 Korean won) in the last calendar year (2017)?

① Less than 6,000 MW

③ About 6,000 MW

⑤ More than 6,000 MW

E054. When did you start receiving specific corporate pension benefits? (unit: year and month combined into 6 digits)

[IWER: Enter the year and month using 6 digits. For example, mark 201701 for January 2017. If the month is not clear, enter 201700. In previous interview, the time of receiving specific corporate pension benefit: (PL) year (PL) month]

_______________________________ [Range: 190000~201712]

**< Income from the Private Pension Benefit>**

E055. Aside from public pensions, such as national pension and specific corporate pension, did you also receive any benefits from a private pension plan in the last calendar year (2017)? If yes, did you receive your benefits monthly or in a lump-sum?

1. Only received monthly benefits
2. Only received lump-sum benefits →Go to E058
3. Received both
4. No →Go to E070

E056. How many month(s) did you receive your private pension benefits in the last calendar year (2017)? (unit: month)

_______________________________ months [Range:1~12]

E057. What was the average monthly amount of private pension benefits in the last calendar year (2017)? (unit: 10,000 Korean won)

[IWER: In previous interview, an average monthly private pension benefit: About (PL) 10,000 Korean won]

_______________________________ MW [Range: 1 ~ 9997] →Go to E058

Don’t know →Go to E060~E064 unfolding

Refuse to answer →Go to E060~E064 unfolding

**Logic**

- **If E055=①, then ask E059.**
- **If E055=② or ③, then ask E058.**

E058. What was the total amount of private pension benefits you received for a lump sum in the last calendar year (2017)? (unit: 10,000 Korean won)

[IWER:

- In previous interview, the total of private pension benefit (a lump sum): About (PL) 10,000 Korean won

- Please enter ‘100,000,000 Korean won (one hundred million won)’ first then, enter ’10,000 Korean won (MW)’.]

______one hundred million won [Range: 1~9] ________ MW [Range: 0 ~ 9997]

Don’t know →Go to E060~E064 unfolding

Refuse to answer →Go to E060~E064 unfolding

E059. So you received a total of (amount calculated based on E056, E057 and E058) in private pension benefits in the last calendar year (2017). Is this correct?

[IWER: In previous interview, the total of private pension benefit: About (PL) 10,000 Korean won]

1. Yes →Go to E065 ⑤ No →Go to E060~E064 unfolding

Don’t know →Go to E060~E064 unfolding

Refuse to answer →Go to E060~E064 unfolding

**< E060~E064. Unfolding bracket questions >**

[IWER: Go back to E058 and record if R answers the amount during the interview.]

E060. Did it amount to a total of less than, about equal to or more than 120 MW (10,000 Korean won) in the last calendar year (2017)?

① Less than 120 MW

③ About 120 MW

⑤ More than 120 MW

E061. Did it amount to a total of less than, about equal to or more than 240 MW (10,000Korean won) in the last calendar year (2017)?

① Less than 240 MW

③ About 240 MW

⑤ More than 240 MW

E062. Did it amount to a total of less than, about equal to or more than 360 MW (10,000Korean won) in the last calendar year (2017)?

① Less than 360 MW

③ About 360 MW

⑤ More than 360 MW

E063. Did it amount to a total of less than, about equal to or more than 600 MW (10,000Korean won) in the last calendar year (2017)?

① Less than 600 MW

③ About 600 MW

⑤ More than 600 MW

E064. Did it amount to a total of less than, about equal to or more than 1,200 MW (10,000Korean won) in the last calendar year (2017)?

① Less than 1,200 MW

③ About 1,200 MW

⑤ More than 1,200 MW

E065. When did you start receiving private pension benefits? (unit: year and month combined into 6 digits)

[IWER:

- Enter the year and month using 6 digits. For example, mark 201701 for January 2017. If the month is not clear, enter 201700.

- In previous interview, the time of receiving national pension benefit: (PL) year (PL) month]

_______________________________ [Range: 190000~201712]

E066. Would you receive this private pension benefit continuously while you are alive?

1. Yes →Go to E068 ⑤ No

E067. How many more years would you receive this private pension benefit? (unit: a year)

_______________________________ years [Range: 0~997]

E068. If you were to die (before then), would the pension benefit stop, continue unchanged, or continue at a reduced level?

1. Will stop →Go to E070
2. Will continue unchanged →Go to E070
3. Will continue at a reduced level →Go to E070
4. Others

E069. If others, please specify.

_______________________________

**< Income from the Social Security Benefit >**

E070. Did you receive any of the following social security benefit in the last calendar year (2017)? Choose all that apply. (Select multiple responses, but with 6.)

1. Unemployment Compensation Benefit
2. Industrial Accident Compensation Insurance Benefit →Go to E081
3. National Basic Livelihood Security Benefit (customized benefit) →Go to E091
4. Veterans Benefit →Go to E100
5. Basic Pension (Ex Basic Old-Age Pension) →Go to E111
6. Disabled Benefit →Go to E121
7. Other Welfare Benefit →Go to E131
8. Earned Income Tax Credit →Go to E140-0
9. No →Go to E140

**< Unemployment Compensation Income >**

**Logic**

- **If E070=①, ask E071.**

E071. If you received unemployment compensation benefit, did you receive your benefit monthly or in a lump sum in the last calendar year (2017)? Unemployment compensation benefit includes job‐seeking benefit, extended-salary benefit, and early reemployment benefit.

1. Only received monthly benefit
2. Only received lump-sum benefit →Go to E074
3. Received both

**Logic**

- **If E071=① or ③, then ask E072**
- **If E071=②, then ask E074**

E072. How many month(s) did you receive unemployment compensation benefit in the last calendar year (2017)? (unit: month)

_______________________________ months [Range:0~12]

E073. What was the average monthly amount of unemployment compensation benefit in the last calendar year (2017)? (unit:10,000 Korean won)

[IWER: In previous interview, an average monthly unemployment compensation benefit: About (PL) 10,000 Korean won]

_______________________________ MW [Range: 0 ~ 150]

Don’t know →Go to E076~E080 unfolding

Refuse to answer →Go to E076~E080 unfolding

**Logic**

- **If E071=①, then ask E075.**
- **If E071=② or ③, then ask E074.**

E074. What was the total amount of unemployment compensation benefit you received for a lump sum in the last calendar year (2017)? (unit:10,000 Korean won)

[IWER:

- In previous interview, the total of unemployment compensation benefit (a lump sum): About (PL) 10,000 Korean won

- Please enter ‘100,000,000 Korean won (one hundred million won)’ first then, enter ’10,000 Korean won (MW)’.]

______one hundred million won [Range: 1~9] _________ MW [Range: 0 ~ 9997]

Don’t know →Go to E076~E080 unfolding

Refuse to answer →Go to E076~E080 unfolding

E075. So you received a total of (amount calculated based on E072, E073 and E074) in the last calendar year (2017). Is this correct?

[IWER: In previous interview, the total of unemployment compensation benefit: About (PL) 10,000 Korean won]

1. Yes →Go to E081 ⑤ No →Go to E076~E080 unfolding

Don’t know →Go to E076~E080 unfolding

Refuse to answer →Go to E076~E080 unfolding

**< E076~E080. Unfolding bracket questions >**

[IWER: Go back to E074 and record if R answers the amount during the interview.]

E076. Did it amount to a total of less than, about equal to or more than 100 MW (10,000 Korean won) in the last calendar year (2017)?

① Less than 100 MW

③ About 100 MW

⑤ More than 100 MW

E077. Did it amount to a total of less than, about equal to or more than 200 MW (10,000 Korean won) in the last calendar year (2017)?

① Less than 200 MW

③ About 200 MW

⑤ More than 200 MW

E078. Did it amount to a total of less than, about equal to or more than 400 MW (10,000 Korean won) in the last calendar year (2017)?

① Less than 400 MW

③ About 400 MW

⑤ More than 400 MW

E079. Did it amount to a total of less than, about equal to or more than 600 MW (10,000 Korean won) in the last calendar year (2017)?

① Less than 600 MW

③ About 600 MW

⑤ More than 600 MW

E080. Did it amount to a total of less than, about equal to or more than 800 MW (10,000 Korean won) in the last calendar year (2017)?

① Less than 800 MW

③ About 800 MW

⑤ More than 800 MW

**< Industrial Accident Compensation Insurance Benefit >**

**Logic**

- **If E070=②, then ask E081.**

E081. If you received industrial accident compensation insurance benefit, did you receive it monthly or in a lump sum in the last calendar year (2017)? Industrial accident compensation insurance benefit includes wage-replacement benefit, disability benefit, and survivors’ benefit.

1. Only received monthly benefit
2. Only received lump-sum benefit →Go to E084
3. Received both

**Logic**

- **If E081=① or ③, then ask E082.**
- **If E081=②, then ask E084.**

E082. How many month(s) did you receive industrial accident compensation insurance benefit in the last calendar year (2017)? (unit: month)

_______________________________ months [Range:0~12]

E083. What was the average monthly amount of industrial accident compensation insurance benefit in the last calendar year (2017)? (unit: 10,000 Korean won)

[IWER: In previous interview, an average monthly industrial accident compensation insurance benefit : About (PL) 10,000 Korean won.]

_______________________________ MW [Range: 1 ~ 9997]

Don’t know →Go to E086~E090 unfolding

Refuse to answer →Go to E086~E090 unfolding

**Logic**

- **If E081=①, then ask E085.**
- **If E081=② or ③, then ask E084.**

E084. What was the total amount of industrial accident compensation insurance benefit you received for a lump sum in the last calendar year (2017)? (unit: 10,000 Korean won)

[IWER:

- In previous interview, the total of industrial accident compensation insurance benefit (a lump sum): About (PL) 10,000 Korean won.

- Please enter ‘100,000,000 Korean won (one hundred million won)’ first then, enter ’10,000 Korean won (MW)’.]

______one hundred million won [Range: 1~9] ________ MW [Range: 0 ~ 9997]

Don’t know →Go to E086~E090 unfolding

Refuse to answer →Go to E086~E090 unfolding

E085. So you received a total of (amount calculated based on E082, E083, and E084) industrial accident compensation insurance benefit in the last calendar year (2017). Is this correct?

[IWER: In previous interview, the total of industrial accident compensation insurance benefit : About (PL) 10,000 Korean won]

1. Yes →Go to E091 ⑤ No →Go to E086~E090 unfolding

Don’t know →Go to E086~E090 unfolding

Refuse to answer →Go to E086~E090 unfolding

**< E086~E090. Unfolding bracket questions >**

[IWER: Go back to E084 and record if R answers the amount during the interview.]

E086. Did it amount to a total of less than, about equal to or more than 300 MW (10,000 Korean won) in the last calendar year (2017)?

① Less than 300 MW

③ About 300 MW

⑤ More than 300 MW

E087. Did it amount to a total of less than, about equal to or more than 600 MW (10,000 Korean won) in the last calendar year (2017)?

① Less than 600 MW

③ About 600 MW

⑤ More than 600 MW

E088. Did it amount to a total of less than, about equal to or more than 1,200 MW (10,000 Korean won) in the last calendar year (2017)?

① Less than 1,200 MW

③ About 1,200 MW

⑤ More than 1,200 MW

E089. Did it amount to a total of less than, about equal to or more than 2,400 MW (10,000 Korean won) in the last calendar year (2017)?

① Less than 2,400 MW

③ About 2,400 MW

⑤ More than 2,400 MW

E090. Did it amount to a total of less than, about equal to or more than 6,000 MW (10,000 Korean won) in the last calendar year (2017)?

① Less than 6,000 MW

③ About 6,000 MW

⑤ More than 6,000 MW

**< National Basic Livelihood Security Benefit (customized benefit) >**

**Logic**

- **If E070=③, then ask E091.**

E091-0. You mentioned that you have received national basic livelihood security benefit (customized benefit) in the last calendar year (2017). Which of the followings apply to you?

① I applied for the first half of 2017 and received the benefit →Go to E091a

③ I applied for the second half of 2017 and received the benefit →Go to E091b

⑤ I applied for both the first half and the second half of 2017 and received the benefit → Ask both E091a and E091b

**Logic**

- **If E091-0=①, ⑤, then ask E091a~E093a.**

E091a. How many month(s) did you receive national basic livelihood security benefit in the first half of last calendar year (January~June in 2017)? (unit: month)

_______________________________ months [Range: 1~6]

Don’t know →Go to E094~E098 unfolding

Refuse to answer →Go to E094~E098 unfolding

E092a. What was the average monthly amount of national basic livelihood security benefit in the first half of last calendar year (January~June in 2017)? (unit: 10,000 Korean won)

[IWER: In previous interview, an average monthly national basic livelihood security benefit: About (PL) 10,000 Korean won.]

_______________________________ MW [Range: 1 ~ 150]

Don’t know →Go to E094~E098 unfolding

Refuse to answer →Go to E094~E098 unfolding

E093a. So you received a total of (amount calculated based on E091a and E092a) in national basic livelihood security benefit in the first half of last calendar year (January~June in 2017). Is this correct?

[IWER: In previous interview, the total of national basic livelihood security benefit: About (PL) 10,000 Korean won]

1. Yes ⑤ No →Go to E094~E098 unfolding

Don’t know →Go to E094~E098 unfolding

Refuse to answer →Go to E094~E098 unfolding

**Logic**

- **If E091-0=③, ⑤, then ask E091b~E093b.**
- **If R answer ‘don’t know’ or ‘refuse to answer’ in any of E091b_1~E092b_3, go to unfolding question**

E091b. How many month(s) did you receive the National basic livelihood security benefit (customized benefit) in the second half of last calendar year (July~December in 2017)? (unit: month)

E091b_1. Living benefit: _______________________________ months [Range: 1~6]

E091b_2. Housing benefit: _____________________________ months [Range: 1~6]

E091b_3. Education benefit: ____________________________ months [Range: 1~6]

E092b. What was the average monthly amount of national basic livelihood security benefit (customized benefit) in the second half of last calendar year (July~December in 2017)? (unit: 10,000 Korean won)

E092b_1. Living benefit: _______________________________ MW [Range: 1 ~ 150]

E092b_2. Housing benefit: _____________________________ MW [Range: 1 ~ 150]

E092b_3. Education benefit: ____________________________ MW [Range: 1 ~ 150]

Don’t know →Go to E094~E098 unfolding

Refuse to answer →Go to E094~E098 unfolding

E093. So you received a total of (amount calculated based on E091b and E092b) in national basic livelihood security benefit in the second half of last calendar year (July~December in 2017). Is this correct?

1. Yes →Go to E099 ⑤ No →Go to E094~E098 unfolding

Don’t know →Go to E094~E098 unfolding

Refuse to answer →Go to E094~E098 unfolding

**< E094~E098. Unfolding bracket questions >**

E094. Did it amount to a total of less than, about equal to or more than 60 MW (10,000 Korean won) in the last calendar year (2017)?

① Less than 60 MW

③ About 60 MW

⑤ More than 60 MW

E095. Did it amount to a total of less than, about equal to or more than 120 MW (10,000 Korean won) in the last calendar year (2017)?

① Less than 120 MW

③ About 120 MW

⑤ More than 120 MW

E096. Did it amount to a total of less than, about equal to or more than 240 MW (10,000 Korean won) in the last calendar year (2017)?

① Less than 240 MW

③ About 240 MW

⑤ More than 240 MW

E097. Did it amount to a total of less than, about equal to or more than 300 MW (10,000 Korean won) in the last calendar year (2017)?

① Less than 300 MW

③ About 300 MW

⑤ More than 300 MW

E098. Did it amount to a total of less than, about equal to or more than 600 MW (10,000 Korean won) in the last calendar year (2017)?

① Less than 600 MW

③ About 600 MW

⑤ More than 600 MW

E099. When did you start receiving national basic livelihood security benefit? (unit: year and month combined into 6 digits)

[IWER: Enter the year and month using 6 digits. For example, mark 201701 for January 2017. If the month is not clear, enter 201700. In previous interview, the time of receiving national basic livelihood security benefit: (PL) year (PL) month]

_______________________________ [Range: 190000~201712]

**< Veterans benefit >**

**Logic**

- **If E070=④, then ask E100.**

E100. If you received veterans benefit, did you receive it monthly or in a lump sum in the last calendar year (2017)? Veterans benefit includes the benefit paid patriots & veterans affairs such as veteran’s salary for patriots or their surviving families, nursing allowances, and pension for wounded policemen and soldiers.

① Only received monthly benefit

② Only received lump-sum benefit →Go to E103

③ Received both

E101. How many month(s) did you receive veterans benefit in the last calendar year (2017)? (unit: month)

_______________________________ months [Range: 1~12]

E102. What was the average monthly amount of veterans benefit in the last calendar year (2017)? (unit: 10,000 Korean won)

[IWER: In previous interview, an average monthly veterans benefit: About (PL) 10,000 Korean won.]

_______________________________ MW [Range: 0 ~ 9997]

Don’t know →Go to E105~E109 unfolding

Refuse to answer →Go to E105~E109 unfolding

**Logic**

- **If E100=①, then ask E104.**
- **If E100=② or ③, then ask E103.**

E103. What was the total amount of veterans benefit you received for a lump sum in the last calendar year (2017)? (unit: 10,000 Korean won)

[IWER:

- In previous interview, the total of veterans benefit (a lump sum): About (PL) 10,000 Korean won.

- Please enter ‘100,000,000 Korean won (one hundred million won)’ first then, enter ’10,000 Korean won (MW)’.]

______one hundred million won [Range: 1~9] _________ MW [Range: 0 ~ 9997]

Don’t know →Go to E105~E109 unfolding

Refuse to answer →Go to E105~E109 unfolding

E104. So you received a total of (amount calculated based on E101, E102, and E103) in veterans benefit in the last calendar year (2017).Is this correct?

[IWER: In previous interview, the total of veterans benefit: About (PL) 10,000 Korean won]

1. Yes →Go to E110 ⑤ No →Go to E105~E109 unfolding

Don’t know →Go to E105~E109 unfolding

Refuse to answer →Go to E105~E109 unfolding

**< E105~E109. Unfolding bracket questions >**

[IWER: Go back to E103 and record if R answers the amount during the interview.]

E105. Did it amount to a total of less than, about equal to or more than 120 MW (10,000 Korean won) in the last calendar year (2017)?

① Less than 120 MW

③ About 120 MW

⑤ More than 120 MW

E106. Did it amount to a total of less than, about equal to or more than 240 MW (10,000 Korean won) in the last calendar year (2017)?

① Less than 240 MW

③ About 240 MW

⑤ More than 240 MW

E107. Did it amount to a total of less than, about equal to or more than 480 MW (10,000 Korean won) in the last calendar year (2017)?

① Less than 480 MW

③ About 480 MW

⑤ More than 480 MW

E108. Did it amount to a total of less than, about equal to or more than 600 MW (10,000 Korean won) in the last calendar year (2017)?

① Less than 600 MW

③ About 600 MW

⑤ More than 600 MW

E109. Did it amount to a total of less than, about equal to or more than 1,200 MW (10,000 Korean won) in the last calendar year (2017)?

① Less than 1,200 MW

③ About 1,200 MW

⑤ More than 1,200 MW

E110. When did you start receiving veterans benefit? (unit: year and month combined into 6 digits)

[IWER: Enter the year and month using 6 digits. For example, mark 201701 for January 2017. If the month is not clear, enter 201700.]

_______________________________ [Range: 190000~201712]

**< Basic Pension (Ex Basic Old-Age Pension) >**

**Logic**

- **If E070=⑤, then ask E111.**

**[IWER: Only consider if R has received for himself/ herself. If R has received benefit for couple, only regard 50% of total benefit.]**

E111. You have received the basic pension last year (2017). The basic old-age pension that has been implemented since 2008 is abolished and now new basic pension is implemented from 2014. This Basic Pension is currently supporting approximately 70% of the population over the age of 65 by paying them up to 25 MW per person or 40 MW per couple to cover their basic living expenses

① Check

E112. How many month(s) did you receive basic old-age pension benefit in the last calendar year (2017)? (unit: month)

_______________________________ months [Range: 1~12]

**Logic**

- **If the answer is from E115~E119, divide the answer by the number of months given at E112 and mark it at E113.**

E113. What was the average monthly amount of basic old-age pension benefit in the last calendar year (2017)? (unit: 10,000 Korean won)

[IWER:

- Round up for 1000 KRW

- Consider for one person. If R has received benefit for couple, only regard 50% of total benefit. (unit: MW)]

_______________________________ MW [Range:2~21]

Don’t know →Go to E115~E119 unfolding

Refuse to answer →Go to E115~E119 unfolding

E114. So you received a total of (amount calculated based on E112 and E113) in basic old-age pension benefit in the last calendar year (2017).Is this correct?

1. Yes →Go to E120 ⑤ No →Go to E115~E119 unfolding

Don’t know →Go to E115~E119 unfolding

Refuse to answer →Go to E115~E119 unfolding

**< E115~E119. Unfolding bracket questions >**

[IWER: Go back to E113 and record if R answers the amount during the interview.]

E115. Did it amount to a total of less than, about equal to or more than 20 MW (10,000 Korean won) in the last calendar year (2017)?

① Less than 20 MW

③ About 20 MW

⑤ More than 20 MW

E116. Did it amount to a total of less than, about equal to or more than 40 MW (10,000 Korean won) in the last calendar year (2017)?

① Less than 40 MW

③ About 40 MW

⑤ More than 40 MW

E117. Did it amount to a total of less than, about equal to or more than 60 MW (10,000 Korean won) in the last calendar year (2017)?

① Less than 60 MW

③ About 60 MW

⑤ More than 60 MW

E118. Did it amount to a total of less than, about equal to or more than 120 MW (10,000 Korean won) in the last calendar year (2017)?

① Less than 120 MW

③ About 120 MW

⑤ More than 120 MW

E119. Did it amount to a total of less than, about equal to or more than 240 MW (10,000 Korean won) in the last calendar year (2017)?

① Less than 240 MW

③ About 240 MW

⑤ More than 240 MW

E120. When did you start receiving basic old-age pension benefit? (unit: year and month combined into 6 digits)

[IWER: Enter the year and month using 6 digits. For example, mark 201701 for January 2017. If the month is not clear, enter 201700.]

_______________________________ [Range: 200800~201712]

**< Disabled Benefit >**

**Logic**

- **If E070=⑥, then ask E121.**

E121. You have received disabled benefit in 2017.

① Check

E122. How many month(s) did you receive disabled benefit in the last calendar year (2017)? (unit: month)

_______________________________ months [Range: 1~12]

**Logic**

- **If the answer is from E125~E129, divide the answer by the number of months given at E122 and mark it at E123.**

E123. What was the average monthly amount of disabled benefit in the last calendar year (2017)? (unit: 10,000 Korean won)

_______________________________ MW [Range 0~9997]

Don’t know →Go to E125~E129 unfolding

Refuse to answer →Go to E125~E129 unfolding

E124. So you received a total of (amount calculated based on E112 and E113) in disabled benefit in the last calendar year (2017).Is this correct?

1. Yes →Go to E130 ⑤ No →Go to E125~E129 unfolding

Don’t know →Go to E125~E129 unfolding

Refuse to answer →Go to E125~E129 unfolding

**< E125~E129. Unfolding bracket questions >**

[IWER: Go back to E123 and record if R answers the amount during the interview.]

E125. Did it amount to a total of less than, about equal to or more than 50 MW (10,000 Korean won) in the last calendar year (2017)?

① Less than 50 MW

③ About 50 MW

⑤ More than 50 MW

E126. Did it amount to a total of less than, about equal to or more than 100 MW (10,000 Korean won) in the last calendar year (2017)?

① Less than 100 MW

③ About 100 MW

⑤ More than 100 MW

E127. Did it amount to a total of less than, about equal to or more than 200 MW (10,000 Korean won) in the last calendar year (2017)?

① Less than 200 MW

③ About 200 MW

⑤ More than 200 MW

E128. Did it amount to a total of less than, about equal to or more than 400 MW (10,000 Korean won) in the last calendar year (2017)?

① Less than 400 MW

③ About 400 MW

⑤ More than 400 MW

E129. Did it amount to a total of less than, about equal to or more than 800 MW (10,000 Korean won) in the last calendar year (2017)?

① Less than 800 MW

③ About 800 MW

⑤ More than 800 MW

E130. When did you start receiving disabled benefit? (unit: year and month combined into 6 digits)

[IWER: Enter the year and month using 6 digits. For example, mark 201701 for January 2017. If the month is not clear, enter 201700.]

_______________________________ [Range: 190000 ~ 201712]

**< Other Welfare Benefit >**

**Logic**

- **If E070=⑦, then ask E131.**

E131. You have received other welfare benefit in 2017.

① Check

E132. How many months did you receive other welfare benefit in the last calendar year (2017)? (unit: month)

_______________________________ months [Range: 1~12]

E133. What was the average monthly amount of other welfare benefit received per month in the last calendar year (2017)? (unit: 10,000 Korean won)

[IWER: In previous interview, an average monthly other welfare benefit: About (PL) 10,000 Korean won.]

_______________________________ MW [Range: 1 ~ 9997]

Don’t know →Go to E135~E139 unfolding

Refuse to answer →Go to E135~E139 unfolding

E134. So you received a total of (amount calculated based on E132 and E133) in other welfare benefit in the last calendar year (2017). Is this correct?

[IWER: In previous interview, the total of other welfare benefit: About (PL) 10,000 Korean won]

1. Yes → Go to E140 ⑤ No →Go to E135~E139 unfolding

Don’t know →Go to E135~E139 unfolding

Refuse to answer →Go to E135~E139 unfolding

**< E135~E139. Unfolding bracket questions >**

[IWER: Go back to E133 and record if R answers the amount during the interview.]

E135. Did it amount to a total of less than, about equal to or more than 50 MW (10,000 Korean won) in the last calendar year (2017)?

① Less than 50 MW

③ About 50 MW

⑤ More than 50 MW

E136. Did it amount to a total of less than, about equal to or more than 100 MW (10,000 Korean won) in the last calendar year (2017)?

① Less than 100 MW

③ About 100 MW

⑤ More than 100 MW

E137. Did it amount to a total of less than, about equal to or more than 200 MW (10,000 Korean won) in the last calendar year (2017)?

① Less than 200 MW

③ About 200 MW

⑤ More than 200 MW

E138. Did it amount to a total of less than, about equal to or more than 300 MW (10,000 Korean won) in the last calendar year (2017)?

① Less than 300 MW

③ About 300 MW

⑤ More than 300 MW

E139. Did it amount to a total of less than, about equal to or more than 600 MW (10,000 Korean won) in the last calendar year (2017)?

① Less than 600 MW

③ About 600 MW

⑤ More than 600 MW

**<Earned Income Tax Credit>**

**Logic**

- **If E070=⑧, then ask E140-0.**

E140-0. What was the total amount of earned income tax credit received in the last calendar year (2017)? (unit: 10,000 Korean won)

_______________________________ MW [Range: 1 ~ 230]

**<Other Income>**

E140. Aside from the income you have talked about so far, did you have any other income in the last calendar year (2017)? Please exclude income from property and financial assets.

[IWER: Other income includes income from alimony, copyright and patent fee. It does not include income from property, house rent, etc., and financial income from bank accounts, securities, bonds, etc. These will be covered in the ASSETS section.]

1. Yes ⑤ No →Go to E147

E141. What was the total amount of other income earned in the last calendar year (2017), after taxes and other deductions? (unit: 10,000 Korean won)

[IWER:

- In previous interview, other income: About (PL) 10,000 Korean won.

- Please enter ‘100,000,000 Korean won (one hundred million won)’ first then, enter ’10,000 Korean won (MW)’.]

______one hundred million won [Range: 1~9] _________ MW [Range: 0 ~ 9997]

Don’t know →Go to E142~E146 unfolding

Refuse to answer →Go to E142~E146 unfolding

**< E142~E146. Unfolding bracket questions >**

[IWER: Go back to E141 and record if R answers the amount during the interview.]

E142. Did it amount to a total of less than, about equal to or more than 120 MW (10,000 Korean won) in the last calendar year (2017)?

① Less than 120 MW

③ About 120 MW

⑤ More than 120 MW

E143. Did it amount to a total of less than, about equal to or more than 240 MW (10,000 Korean won)in the last calendar year (2017)?

① Less than 240 MW

③ About 240 MW

⑤ More than 240 MW

E144. Did it amount to a total of less than, about equal to or more than 480 MW (10,000 Korean won) in the last calendar year (2017)?

① Less than 480 MW

③ About 480 MW

⑤ More than 480 MW

E145. Did it amount to a total of less than, about equal to or more than 600 MW (10,000 Korean won) in the last calendar year (2017)?

① Less than 600 MW

③ About 600 MW

⑤ More than 600 MW

E146. Did it amount to a total of less than, about equal to or more than 1,200 MW (10,000 Korean won) in the last calendar year (2017)?

① Less than 1,200 MW

③ About 1,200 MW

⑤ More than 1,200 MW

**< Household Income >**

E147. What was the total household income of your household members, including you, earned in the last calendar year (2017)? (unit: 10,000Korean won)

[IWER:

- In previous interview, the total of household income: About (PL) 10,000 Korean won.

- Please enter ‘100,000,000 Korean won (one hundred million won)’ first then, enter ’10,000 Korean won (MW)’.]

______one hundred million won [Range: 1~99] ________ MW [Range: 0 ~ 9997] → E153

Don’t know →Go to E148~E152 unfolding

Refuse to answer →Go to E148~E152 unfolding

**<E148~E152. Unfolding bracket questions >**

[IWER: Go back to E147 and record if R answers the amount during the interview.]

E148. Did it amount to a total of less than, about equal to or more than 1,000 MW (10,000 Korean won) in the last calendar year (2017)?

① Less than 1,000 MW

③ About 1,000 MW

⑤ More than 1,000 MW

E149. Did it amount to a total of less than, about equal to or more than 2,000 MW (10,000 Korean won) in the last calendar year (2017)?

① Less than 2,000 MW

③ About 2,000 MW

⑤ More than 2,000 MW

E150. Did it amount to a total of less than, about equal to or more than 3,000 MW (10,000 Korean won) in the last calendar year (2017)?

① Less than 3,000 MW

③ About 3,000 MW

⑤ More than 3,000 MW

E151. Did it amount to a total of less than, about equal to or more than 5,000 MW (10,000 Korean won) in the last calendar year (2017)?

① Less than 5,000 MW

③ About 5,000 MW

⑤ More than 5,000 MW

E152. Did it amount to a total of less than, about equal to or more than 10,000 MW (10,000 Korean won) in the last calendar year (2017)?

① Less than 10,000 MW

③ About 10,000 MW

1. More than 10,000 MW

Eadd_01. Did any household members (including respondent) living with you last year have bad credit?

1. Yes ⑤ No

E153. [IWER: How often did R receive assistance in answering section E-INCOME?]

1. Never →Go to section E2 or F.
2. A few times →Go to section E2 or F.
3. Most or all of the times →Go to section E2 or F.
4. The section was done by a proxy respondent.

E154. If done by a proxy respondent, what is the proxy’s relationship to R? Please answer in view of the proxy.

1. Spouse
2. Mother
3. Father
4. Mother-in-law
5. Father-in-law
6. Brother/sister
7. Brother-in-law/sister-in-law
8. Son/daughter
9. Son-in-law/Daughter-in-law
10. Grandchild
11. Other relatives
12. Helper or other non-relatives

→Go to sectionE2 or F.

(E_end) [IWER] Please enter code 1 for completion of E. Income section.

1. Completion

**< Consumption >**

(Eb_Intro) [IWER] Now, this is to start Eb. CONSUMPTION section.

1. Check

E201.Now I am going to ask you about your living expenses and other items in the last calendar year (2017). Other items include food and eating out, public and private education, housing, medical, clothing, insurance premium, vehicle maintenance, telecommunication, leisure, family events/ tribute fee, etc. First, how much did your household spend living expenses in an average month in the last calendar year (2017)? (unit: 10,000 Korean won)

_______________________________ MW [Range: 0~9997] →Go to E207

Don't know →Go to E202~E206 unfolding

Refuse to answer →Go to E202~E206 unfolding

**< E202~E206. Unfolding bracket questions >**

[IWER: Go back to E201 and record if R answers the amount during the interview.]

E202. Did it amount to a total of less than, about equal to or more than 50 MW (10,000 Korean won) in the last calendar year (2017)?

① Less than 50 MW

③ About 50 MW

⑤ More than 50 MW

E203. Did it amount to a total of less than, about equal to or more than 100 MW (10,000 Korean won) in the last calendar year (2017)?

① Less than 100 MW

③ About 100 MW

⑤ More than 100 MW

E204. Did it amount to a total of less than, about equal to or more than 200 MW (10,000 Korean won) in the last calendar year (2017)?

① Less than 200 MW

③ About 200 MW

⑤ More than 200 MW

E205. Did it amount to a total of less than, about equal to or more than 300 MW (10,000 Korean won) in the last calendar year (2017)?

① Less than 300 MW

③ About 300 MW

⑤ More than 300 MW

E206. Did it amount to a total of less than, about equal to or more than 500 MW (10,000 Korean won) in the last calendar year (2017)?

① Less than 500 MW

③ About 500 MW

⑤ More than 500 MW

**< Food expenses >**

E207. How much did your household spend on food (including main meals and snack) in an average month in the last calendar year (2017)? (unit: 10,000 Korean won)

_______________________________ MW [Range: 0~9997] →Go to E213

Don't know →Go to E208~E212 unfolding

Refuse to answer →Go to E208~E212 unfolding

**< E208~E212. Unfolding bracket questions >**

[IWER: Go back to E207 and record if R answers the amount during the interview.]

E208. Did it amount to a total of less than, about equal to or more than 10 MW (10,000 Korean won) in the last calendar year (2017)?

① Less than 10 MW

③ About 10 MW

⑤ More than 10 MW

E209. Did it amount to a total of less than, about equal to or more than 30 MW (10,000 Korean won) in the last calendar year (2017)?

① Less than 30 MW

③ About 30 MW

⑤ More than 30 MW

E210. Did it amount to a total of less than, about equal to or more than 40 MW (10,000 Korean won) in the last calendar year (2017)?

① Less than 40 MW

③ About 40 MW

⑤ More than 40 MW

E211. Did it amount to a total of less than, about equal to or more than 60 MW (10,000 Korean won) in the last calendar year (2017)?

① Less than 60 MW

③ About 60 MW

⑤ More than 60 MW

E212. Did it amount to a total of less than, about equal to or more than 100 MW (10,000 Korean won) in the last calendar year (2017)?

① Less than 100 MW

③ About 100 MW

⑤ More than 100 MW

**< Eating out expenses >**

E213. How much did your household spend eating out in an average month in the last calendar year (2017)? (unit: 10,000 Korean won)

[IWER: Please include all the costs for eating outside of the home]

_______________________________ MW [Range: 0~9997] →Go to E219

Don't know →Go to E214~E218 unfolding

Refuse to answer →Go to E214~E218 unfolding

**< E214~E218. Unfolding bracket questions >**

[IWER: Go back to E213 and record if R answers the amount during the interview.]

E214. Did it amount to a total of less than, about equal to or more than 5 MW (10,000 Korean won) in the last calendar year (2017)?

① Less than 5 MW

③ About 5 MW

⑤ More than 5 MW

E215. Did it amount to a total of less than, about equal to or more than 10 MW (10,000 Korean won) in the last calendar year (2017)?

① Less than 10 MW

③ About 10 MW

⑤ More than 10 MW

E216. Did it amount to a total of less than, about equal to or more than 20 MW (10,000 Korean won) in the last calendar year (2017)?

① Less than 20 MW

③ About 20 MW

⑤ More than 20 MW

E217. Did it amount to a total of less than, about equal to or more than 30 MW (10,000 Korean won) in the last calendar year (2017)?

① Less than 30 MW

③ About 30 MW

⑤ More than 30 MW

E218. Did it amount to a total of less than, about equal to or more than 50 MW (10,000 Korean won) in the last calendar year (2017)?

① Less than 50 MW

③ About 50 MW

1. More than 50 MW

**< Public education expenses >**

E219. How much did your household spend on public education (tuition, textbooks, materials, etc.) in an average month in the last calendar year (2017)? (unit: 10,000 Korean won)

[IWER: Boarding expenses during the course of education are included in housing costs, so record them in E231.]

_______________________________ MW [Range: 0~9997] →Go to Eadd_02

Don't know →Go to E220~E224 unfolding

Refuse to answer →Go to E220~E224 unfolding

**< E220~E224. Unfolding bracket questions >**

[IWER: Go back to E219 and record if R answers the amount during the interview.]

E220. Did it amount to a total of less than, about equal to or more than 5 MW (10,000 Korean won) in the last calendar year (2017)?

① Less than 5 MW

③ About 5 MW

⑤ More than 5 MW

E221. Did it amount to a total of less than, about equal to or more than 10 MW (10,000 Korean won) in the last calendar year (2017)?

① Less than 10 MW

③ About 10 MW

⑤ More than 10 MW

E222. Did it amount to a total of less than, about equal to or more than 30 MW (10,000 Korean won) in the last calendar year (2017)?

① Less than 30 MW

③ About 30 MW

⑤ More than 30 MW

E223. Did it amount to a total of less than, about equal to or more than 50 MW (10,000 Korean won) in the last calendar year (2017)?

① Less than 50 MW

③ About 50 MW

⑤ More than 50 MW

E224. Did it amount to a total of less than, about equal to or more than 100 MW (10,000 Korean won) in the last calendar year (2017)?

① Less than 100 MW

③ About 100 MW

⑤ More than 100 MW

Eadd_02. Have you been unable to pay for your child's (including college students) public education for more than a month last year because of financial difficulties?

[Iwer:

- Persons eligible for subsidies for public education expenses are limited to children (excluding grandchildren, siblings, etc.)

- Public education only covers elementary, middle, high school, and university education, except graduate school or higher.

1. Yes

⑤ No

⑥ Not applicable (no children, graduate student)

**< Private educational expenses >**

E225. How much did you spend on private education (private educational institutions, tutoring, textbooks, materials, etc.) in an average month in the last calendar year (2017)? (unit: 10,000 Korean won)

_______________________________ MW [Range: 0~9997] →Go to E231

Don't know →Go to E226~E230 unfolding

Refuse to answer →Go to E226~E230 unfolding

**< E226~E230. Unfolding bracket questions >**

[IWER: Go back to E225 and record if R answers the amount during the interview.]

E226. Did it amount to a total of less than, about equal to or more than 10 MW (10,000 Korean won) in the last calendar year (2017)?

① Less than 10 MW

③ About 10 MW

⑤ More than 10 MW

E227. Did it amount to a total of less than, about equal to or more than 20 MW (10,000 Korean won) in the last calendar year (2017)?

① Less than 20 MW

③ About 20 MW

⑤ More than 20 MW

E228. Did it amount to a total of less than, about equal to or more than 50 MW (10,000 Korean won) in the last calendar year (2017)?

① Less than 50 MW

③ About 50 MW

⑤ More than 50 MW

E229. Did it amount to a total of less than, about equal to or more than 100 MW (10,000 Korean won) in the last calendar year (2017)?

① Less than 100 MW

③ About 100 MW

⑤ More than 100 MW

E230. Did it amount to a total of less than, about equal to or more than 200 MW (10,000 Korean won) in the last calendar year (2017)?

① Less than 200 MW

③ About 200 MW

⑤ More than 200 MW

**< Housing expenses >**

E231. How much did your household spend on housing (monthly rent, utilities including electricity, water and gas, etc.) in an average month in the last calendar year (2017)? (unit: 10,000 Korean won)

[IWER: Boarding expenses are included in housing costs.]

_______________________________ MW [Range: 0~9997] →Go to Eadd_03

Don't know →Go to E232~E236 unfolding

Refuse to answer →Go to E232~E236 unfolding

**< E232~E236. Unfolding bracket questions >**

[IWER: Go back to E231 and record if R answers the amount during the interview.]

E232. Did it amount to a total of less than, about equal to or more than 5 MW (10,000 Korean won) in the last calendar year (2017)?

① Less than 5 MW

③ About 5 MW

⑤ More than 5 MW

E233. Did it amount to a total of less than, about equal to or more than 10 MW (10,000 Korean won) in the last calendar year (2017)?

① Less than 10 MW

③ About 10 MW

⑤ More than 10 MW

E234. Did it amount to a total of less than, about equal to or more than 20 MW (10,000 Korean won) in the last calendar year (2017)?

① Less than 20 MW

③ About 20 MW

⑤ More than 20 MW

E235. Did it amount to a total of less than, about equal to or more than 50 MW (10,000 Korean won) in the last calendar year (2017)?

① Less than 50 MW

③ About 50 MW

⑤ More than 50 MW

E236. Did it amount to a total of less than, about equal to or more than 100 MW (10,000 Korean won) in the last calendar year (2017)?

① Less than 100 MW

③ About 100 MW

⑤ More than 100 MW

**< Medical expenses >**

Eadd_03. Have you ever moved house last year because your monthly rent (half-chonsei, monthly rent) was overdue for more than two months or you could not pay due to financial difficulties?

① Yes

⑤ No

⑥ Not applicable (own home)

Eadd_04. Have you ever been unable to pay utility bills (e.g. electricity, telephone, water or gas charges) due to financial difficulties last year?

1. Yes ⑤ No

Eadd_05. Have you ever been cut off from electricity, telephone, water, or gas last year because you couldn't pay more than one of the charges for electricity, telephone, water, or gas?

1. Yes ⑤ No

Eadd_06. Have you ever been unable to heat in the cold winter because of financial difficulties last year?

[Iwer: Meaning minimum basic heating, except for the use of additional heaters (hot water heat mats, air heaters, electric blankets, etc.)]

1. Yes ⑤ No

E237. How much did your household spend on medical expenditure (including health insurance premiums) in an average month in the last calendar year (2017)? (unit: 10,000 Korean won)

_______________________________ MW [Range: 0~9997] →Go to E243

Don't know →Go to E238~E242 unfolding

Refuse to answer →Go to E238~E242 unfolding

**< E238~E242. Unfolding bracket questions >**

[IWER: Go back to E237 and record if R answers the amount during the interview.]

E238. Did it amount to a total of less than, about equal to or more than 5 MW (10,000 Korean won) in the last calendar year (2017)?

① Less than 5 MW

③ About 5 MW

⑤ More than 5 MW

E239. Did it amount to a total of less than, about equal to or more than 10 MW (10,000 Korean won) in the last calendar year (2017)?

① Less than 10 MW

③ About 10 MW

⑤ More than 10 MW

E240. Did it amount to a total of less than, about equal to or more than 20 MW (10,000 Korean won) in the last calendar year (2017)?

① Less than 20 MW

③ About 20 MW

⑤ More than 20 MW

E241. Did it amount to a total of less than, about equal to or more than 50 MW (10,000 Korean won) in the last calendar year (2017)?

① Less than 50 MW

③ About 50 MW

⑤ More than 50 MW

E242. Did it amount to a total of less than, about equal to or more than 100 MW (10,000 Korean won) in the last calendar year (2017)?

① Less than 100 MW

③ About 100 MW

⑤ More than 100 MW

**< Clothing expenses >**

E243. How much did your household spend on clothing (clothes, shoes, etc.) in an average month in the last calendar year (2017)? (unit: 10,000 Korean won)

_______________________________ MW [Range: 0~9997] →Go to E249

Don't know →Go to E244~E248 unfolding

Refuse to answer →Go to E244~E248 unfolding

**< E244~E248. Unfolding bracket questions >**

[IWER: Go back to E243 and record if R answers the amount during the interview.]

E244. Did it amount to a total of less than, about equal to or more than 5 MW (10,000 Korean won) in the last calendar year (2017)?

① Less than 5 MW

③ About 5 MW

⑤ More than 5 MW

E245. Did it amount to a total of less than, about equal to or more than 10 MW (10,000 Korean won) in the last calendar year (2017)?

① Less than 10 MW

③ About 10 MW

⑤ More than 10 MW

E246. Did it amount to a total of less than, about equal to or more than 20 MW (10,000 Korean won) in the last calendar year (2017)?

① Less than 20 MW

③ About 20 MW

⑤ More than 20 MW

E247. Did it amount to a total of less than, about equal to or more than 30 MW (10,000 Korean won) in the last calendar year (2017)?

① Less than 30 MW

③ About 30 MW

⑤ More than 30 MW

E248. Did it amount to a total of less than, about equal to or more than 50 MW (10,000 Korean won) in the last calendar year (2017)?

① Less than 50 MW

③ About 50 MW

⑤ More than 50 MW

**< Insurance premium expense >**

E249. How much did your household spend on insurance premium in an average month in the last calendar year (2017)? (unit: 10,000 Korean won)

_______________________________ MW [Range: 0~9997] →Go to E255

Don't know →Go to E250~E254 unfolding

Refuse to answer →Go to E250~E254 unfolding

**< E250~E254. Unfolding bracket questions >**

[IWER: Go back to E249 and record if R answers the amount during the interview.]

E250. Did it amount to a total of less than, about equal to or more than 5 MW (10,000 Korean won) in the last calendar year (2017)?

① Less than 5 MW

③ About 5 MW

⑤ More than 5 MW

E251. Did it amount to a total of less than, about equal to or more than 10 MW (10,000 Korean won) in the last calendar year (2017)?

① Less than 10 MW

③ About 10 MW

⑤ More than 10 MW

E252. Did it amount to a total of less than, about equal to or more than 20 MW (10,000 Korean won) in the last calendar year (2017)?

① Less than 20 MW

③ About 20 MW

⑤ More than 20 MW

E253. Did it amount to a total of less than, about equal to or more than 50 MW (10,000 Korean won) in the last calendar year (2017)?

① Less than 50 MW

③ About 50 MW

⑤ More than 50 MW

E254. Did it amount to a total of less than, about equal to or more than 100 MW (10,000 Korean won) in the last calendar year (2017)?

① Less than 100 MW

③ About 100 MW

⑤ More than 100 MW

**<Vehicle maintenance expenses>**

E255. How much did your household spend on vehicle maintenance in an average month in the last calendar year (2017)? (unit: 10,000 Korean won)

_______________________________ MW [Range: 0~9997] →Go to E261

Don't know →Go to E256~E260 unfolding

Refuse to answer →Go to E256~E260 unfolding

**< E256~E260. Unfolding bracket questions >**

[IWER: Go back to E255 and record if R answers the amount during the interview.]

E256. Did it amount to a total of less than, about equal to or more than 5 MW (10,000 Korean won) in the last calendar year (2017)?

① Less than 5 MW

③ About 5 MW

⑤ More than 5 MW

E257. Did it amount to a total of less than, about equal to or more than 10 MW (10,000 Korean won) in the last calendar year (2017)?

① Less than 10 MW

③ About 10 MW

⑤ More than 10 MW

E258. Did it amount to a total of less than, about equal to or more than 20 MW (10,000 Korean won) in the last calendar year (2017)?

① Less than 20 MW

③ About 20 MW

⑤ More than 20 MW

E259. Did it amount to a total of less than, about equal to or more than 50 MW (10,000 Korean won) in the last calendar year (2017)?

① Less than 50 MW

③ About 50 MW

⑤ More than 50 MW

E260. Did it amount to a total of less than, about equal to or more than 100 MW (10,000 Korean won) in the last calendar year (2017)?

① Less than 100 MW

③ About 100 MW

⑤ More than 100 MW

**<Telecommunication expenses >**

E261. How much did your household spend on telecommunication service in an average month in the last calendar year (2017)? (unit: 10,000 Korean won)

_______________________________ MW [Range: 0~9997] →Go to E267

Don't know →Go to E262~E266 unfolding

Refuse to answer →Go to E262~E266 unfolding

**< E262~E266. Unfolding bracket questions >**

[IWER: Go back to E261 and record if R answers the amount during the interview.]

E262. Did it amount to a total of less than, about equal to or more than 5 MW (10,000 Korean won) in the last calendar year (2017)?

① Less than 5 MW

③ About 5 MW

⑤ More than 5 MW

E263. Did it amount to a total of less than, about equal to or more than 10 MW (10,000 Korean won) in the last calendar year (2017)?

① Less than 10 MW

③ About 10 MW

⑤ More than 10 MW

E264. Did it amount to a total of less than, about equal to or more than 20 MW (10,000 Korean won) in the last calendar year (2017)?

① Less than 20 MW

③ About 20 MW

⑤ More than 20 MW

E265. Did it amount to a total of less than, about equal to or more than 50 MW (10,000 Korean won) in the last calendar year (2017)?

① Less than 50 MW

③ About 50 MW

⑤ More than 50 MW

E266. Did it amount to a total of less than, about equal to or more than 100 MW (10,000 Korean won) in the last calendar year (2017)?

① Less than 100 MW

③ About 100 MW

⑤ More than 100 MW

**< Leisure expenses >**

E267. How much did your household spend on leisure activities (travel, picnic, movie, performance, exhibition, sport game, hobby or any community activities) in an average month in the last calendar year (2017)? (unit: 10,000 Korean won)

_______________________________ MW [Range: 0~9997] →Go to E273

Don't know →Go to E268~E272 unfolding

Refuse to answer →Go to E268~E272 unfolding

**< E268~E272. Unfolding bracket questions >**

[IWER: Go back to E267 and record if R answers the amount during the interview.]

E268. Did it amount to a total of less than, about equal to or more than 5 MW (10,000 Korean won) in the last calendar year (2017)?

① Less than 5 MW

③ About 5 MW

⑤ More than 5 MW

E269. Did it amount to a total of less than, about equal to or more than 10 MW (10,000 Korean won) in the last calendar year (2017)?

① Less than 10 MW

③ About 10 MW

⑤ More than 10 MW

E270. Did it amount to a total of less than, about equal to or more than 20 MW (10,000 Korean won) in the last calendar year (2017)?

① Less than 20 MW

③ About 20 MW

⑤ More than 20 MW

E271. Did it amount to a total of less than, about equal to or more than 50 MW (10,000 Korean won) in the last calendar year (2017)?

① Less than 50 MW

③ About 50 MW

⑤ More than 50 MW

E272. Did it amount to a total of less than, about equal to or more than 100 MW (10,000 Korean won) in the last calendar year (2017)?

① Less than 100 MW

③ About 100 MW

⑤ More than 100 MW

**<Family events/ tribute fee >**

E273. How much did your household spend on family events/ tributes in an average month in the last calendar year (2017)? (unit: 10,000 Korean won)

_______________________________ MW [Range: 0~9997] →Go to E279

Don't know →Go to E274~E278 unfolding

Refuse to answer →Go to E274~E278 unfolding

**< E274~E278. Unfolding bracket questions >**

[IWER: Go back to E273 and record if R answers the amount during the interview.]

E274. Did it amount to a total of less than, about equal to or more than 5 MW (10,000 Korean won) in the last calendar year (2017)?

① Less than 5 MW

③ About 5 MW

⑤ More than 5 MW

E275. Did it amount to a total of less than, about equal to or more than 10 MW (10,000 Korean won) in the last calendar year (2017)?

① Less than 10 MW

③ About 10 MW

⑤ More than 10 MW

E276. Did it amount to a total of less than, about equal to or more than 20 MW (10,000 Korean won) in the last calendar year (2017)?

① Less than 20 MW

③ About 20 MW

⑤ More than 20 MW

E277. Did it amount to a total of less than, about equal to or more than 50 MW (10,000 Korean won) in the last calendar year (2017)?

① Less than 50 MW

③ About 50 MW

⑤ More than 50 MW

E278. Did it amount to a total of less than, about equal to or more than 100 MW (10,000 Korean won) in the last calendar year (2017)?

① Less than 100 MW

③ About 100 MW

⑤ More than 100 MW

**Logic**

- **If E147<(E201ⅹ12), then ask E279.**

E279. If your income is not sufficient for living expenses, how did you cover your living expenses? Choose the three main activities that you have done mostly.

[IWER: Cancellation of an insurance contract or lodge money from private savings club in advance falls into ‘⑧ Cancelled savings, deposits or installment savings’. Raising rent falls into ‘⑥Sold property or raised house rent’.]

First: ___________________

Second: ________________

Third: __________________

1. Used bank loans or overdrafts
2. Relied on cash advances
3. Borrowed money from relatives
4. Borrowed money from friends and neighbors
5. Used private loans
6. Sold property or raised house rent of property
7. Reduced the price of lease (e.g. monthly rent feel)
8. Cancelled savings, deposits or installment savings
9. Sold financial assets including stocks and bonds
10. Sold car, durables or valuables such as gold and silver
11. Others

E280. If others, please specify.

_______________________________

**< Savings >**

E281. I am going to ask you about savings. How much was your household’s savings in an average month in the last calendar year (2017)? Savings include general saving, installment savings, insurance, personal pension and private savings club (Gye). (unit: 10,000 Korean won)

_______________________________MW [Range: 0~9997] →Go to E287

Don't know →Go to E282~E286 unfolding

Refuse to answer →Go to E282~E286 unfolding

**< E282~E286. Unfolding bracket questions >**

[IWER: Go back to E281 and record if R answers the amount during the interview.]

E282. Did it amount to a total of less than, about equal to or more than 20 MW (10,000 Korean won) in the last calendar year (2017)?

① Less than 20 MW

③ About 20 MW

⑤ More than 20 MW

E283. Did it amount to a total of less than, about equal to or more than 50 MW (10,000 Korean won) in the last calendar year (2017)?

① Less than 50 MW

③ About 50 MW

⑤ More than 50 MW

E284. Did it amount to a total of less than, about equal to or more than 100 MW (10,000 Korean won) in the last calendar year (2017)?

① Less than 100 MW

③ About 100 MW

⑤ More than 100 MW

E285. Did it amount to a total of less than, about equal to or more than 200 MW (10,000 Korean won) in the last calendar year (2017)?

① Less than 200 MW

③ About 200 MW

⑤ More than 200 MW

E286. Did it amount to a total of less than, about equal to or more than 500 MW (10,000 Korean won) in the last calendar year (2017)?

① Less than 500 MW

③ About 500 MW

⑤ More than 500 MW

E287. [IWER: How often did R receive assistance in answering section E-INCOME AND CONSUMPTION?]

1. Never →Go to section F.
2. A few times →Go to section F.
3. Most or all of the time →Go to section F.
4. The section was done by a proxy respondent.

E288. If done by a proxy respondent, what is the proxy’s relationship to R? Please answer in view of the proxy.

1. Spouse
2. Mother
3. Father
4. Mother-in-law
5. Father-in-law
6. Brother/sister
7. Brother-in-law/sister-in-law
8. Son/daughter
9. Son-in-law/Daughter-in-law
10. Grandchild
11. Other relatives
12. Helper or other non-relatives

→Go to section F.

(Eb_end) [IWER] Please enter code 1 for completion of CONSUMPTION section.

1. Completion

# **F. ASSETS AND DEBTS**

(F_Intro) [IWER] Now, this is to start F. ASSET section.

1. Check

F001. Are you living in your own home or a rented home?

[IWER: “Please tell me the tenure type (own home, deposit-based rental home without monthly rent, monthly rental home with or without a deposit) of the home you are currently residing in. This is not about whose name of home holder.” For example, if R is living in his/her child’s home (home under the name of his/her child), the tenure type is R’s own home.]

1. Own home (home owned by R or R’s relative) →Go to F004
2. Deposit-based rental home without monthly rent (i.e., JEON-SE) →Go to F033
3. Monthly rental home with a deposit (i.e., JEON‐WOL‐SE) →Go to F041
4. Monthly rental home without a deposit (i.e., WOL‐SE) →Go to F049
5. Others →Go to F002

F002. If others, please specify.

_______________________________ →Go to F057

**Logic**

- **If CV013=⑤ and (response to F001 in the previous interview≠response to F001 in this interview), then go to F003.**
- **After asking F003, (if F001=①), then go to F004.**
- **After asking F003, (if F001=②), then go to F033.**
- **After asking F003, (if F001=③), then go to F041.**
- **After asking F003, (if F001=④), then go to F049**
- **After asking F003, (if F001=⑤), then go to F057.**

F003. The tenure type (own home, deposit-based rental home without monthly rent, monthly rental home with or without a deposit) of the home you are currently residing in is different from your response in the previous interview. Has the tenure type of your home been changed since the previous interview?

1. Yes ⑤ No

**< Housing (own home) >**

**Logic**

- **If F001=①, then ask F004.**

F004. Is the home you are currently residing in under your name?

1. Yes, R's name (including joint ownership)
2. No, other's name – spouse →Go to F057
3. No, other's name - (grand)children →Go to F057
4. No, other's name - other family members →Go to F057
5. No, other's name - other persons →Go to F057

F005. Is the home you are currently residing in under multiple persons’ names? If so, how many names are listed on the home deed? (unit: a person)

[IWER: If R’s name is listed, he/she should be counted in. For example, R and his/her spouse are both listed on the home deed, enter 2. If the home is not under multiple persons’ names, enter 0.]

_______________________________ people [Range: 0~20]

**Logic**

- **If F005>=2, then ask F006. If not, then go to F007.**

F006. Then, how much is your share (of the home you are currently residing in)? (unit: %)

_______________________________ % [Range: 1~99]

F007. How much is your home price now? If you sold it today, about how much would you get? (unit:10,000 Korean won)

[IWER: Please enter ‘100,000,000 Korean won (one hundred million won)’ first then, enter ’10,000 Korean won (MW)’.]

______one hundred million won [Range: 1~999] _______ MW [Range: 0 ~ 9997] → F013

Don’t know →Go to F008~F012 unfolding

Refuse to answer →Go to F008~F012 unfolding

**< F008~F012. Unfolding bracket questions >**

[IWER: Go back to F007 and record if R answers the amount during the interview.]

F008. Would it amount to less than, about equal to or more than 1,000 MW (10,000 Korean won)?

① Less than 1,000 MW

③ About 1,000 MW

⑤ More than 1,000 MW

F009. Would it amount to less than, about equal to, or more than 5,000 MW (10,000 Korean won)?

① Less than 5,000 MW

③ About 5,000 MW

⑤ More than 5,000 MW

F010. Would it amount to less than, about equal to or more than 10,000 MW (10,000 Korean won)?

① Less than 10,000 MW

③ About 10,000 MW

⑤ More than 10,000 MW

F011. Would it amount to less than, about equal to or more than 50,000 MW (10,000 Korean won)?

① Less than 50,000 MW

③ About 50,000 MW

⑤ More than 50,000 MW

F012. Would it amount to less than, about equal to or more than 100,000 MW(10,000 Korean won)?

① Less than 100,000 MW

③ About 100,000 MW

⑤ More than 100,000 MW

F013. Did you get a loan or incur a debt in order to purchase the home you are currently residing in? How much is the remaining loan or debt? (unit: 10,000 Korean won)

[IWER: “Please tell me only the amount of loan or debt **you** have.”

- If R has no loan or debt, enter 0.

- Please enter ‘100,000,000 Korean won (one hundred million won)’ first then, enter ’10,000 Korean won (MW)’.

______one hundred million won [Range: 0~999] _________ MW [Range: 0 ~ 9997]

F014. Are you (and your spouse) currently leasing parts of the home you are currently residing in to other people? Choose all that apply.

1. Leasing for a deposit basis without monthly rent (i.e. JEON‐SE)
2. Leasing for a secured monthly rent basis (i.e. JEON‐WOL‐SE)
3. Leasing for a monthly rent basis without deposit (i.e. WOL‐SE)
4. Not leasing →Go to F057

**Logic**

- **If F014=①, ①② ,①③, ①②③, then ask F015.**

F015. When you leased it, how much security deposit did you receive for the deposit-based rental home without monthly rent (i.e. JEON‐SE)? Please only include the amount of security deposit of the home you are currently residing in. (unit: 10,000 Korean won)

[IWER: Please enter ‘100,000,000 Korean won (one hundred million won)’ first then, enter ’10,000 Korean won (MW)’.]

______one hundred million won [Range: 1~99] ________ MW [Range: 0 ~ 9997] → F021

Don’t know →Go to F016~F020 unfolding

Refuse to answer →Go to F016~F020 unfolding

**< F016~F020 Unfolding bracket questions >**

[IWER: Go back to F015 and record if R answers the amount during the interview.]

F016. Did it amount to less than, about equal to or more than 1,000 MW (10,000 Korean won)?

① Less than 1,000 MW

③ About 1,000 MW

⑤ More than 1,000 MW

F017. Did it amount to less than, about equal to or more than 3,000 MW (10,000 Korean won)?

① Less than 3,000 MW

③ About 3,000 MW

⑤ More than 3,000 MW

F018. Did it amount to less than, about equal to or more than 5,000 MW (10,000 Korean won)?

① Less than 5,000 MW

③ About 5,000 MW

⑤ More than 5,000 MW

F019. Did it amount to less than, about equal to or more than 10,000 MW (10,000 Korean won)?

① Less than 10,000 MW

③ About 10,000 MW

⑤ More than 10,000 MW

F020. Did it amount to less than, about equal to or more than 30,000 MW (10,000 Korean won)?

① Less than 30,000 MW

③ About 30,000 MW

⑤ More than 30,000 MW

**Logic**

- **If F014=②, ①②, ②③, ①②③, then go to F021. If not, then go to F027.**

F021. When you leased it, how much security deposit did you receive for the monthly rental home with a deposit (i.e. JEON‐WOL‐SE)? Please only include the amount of security deposit of the home you are currently residing in. (unit: 10,000 Korean won).

[IWER: Please enter ‘100,000,000 Korean won (one hundred million won)’ first then, enter ’10,000 Korean won (MW)’.]

______one hundred million won [Range: 1~99] ________ MW [Range: 0 ~ 9997] → F027

Don’t know →Go to F022~F026 unfolding

Refuse to answer →Go to F022~F026 unfolding

**< F022~F026. Unfolding bracket questions >**

[IWER: Go back to F021 and record if R answers the amount during the interview.]

F022. Did it amount to less than, about equal to or more than 300 MW (10,000 Korean won)?

① Less than 300 MW

③ About 300 MW

⑤ More than 300 MW

F023. Did it amount to less than, about equal to or more than 500 MW (10,000 Korean won)?

① Less than 500 MW

③ About 500 MW

⑤ More than 500 MW

F024. Did it amount to less than, about equal to or more than 1,000 MW (10,000 Korean won)?

① Less than 1,000 MW

③ About 1,000 MW

⑤ More than 1,000 MW

F025. Did it amount to less than, about equal to or more than 5,000 MW (10,000 Korean won)?

① Less than 5,000 MW

③ About 5,000 MW

⑤ More than 5,000 MW

F026. Did it amount to less than, about equal to or more than 10,000 MW (10,000Korean won)?

① Less than 10,000 MW

③ About 10,000 MW

⑤ More than 10,000 MW

**Logic**

- **If F014=②, ①②, then ask F027.**
- **If F014=③, ①③, ②③, ①②③, then ask F027. If not, then ask F055.**

F027. When you leased it, how much rent did you receive per month on average? Please only include the lease of the home you are currently residing in. (unit: 10,000 Korean won)

[IWER: Please enter ‘100,000,000 Korean won (one hundred million won)’ first then, enter ’10,000 Korean won (MW)’.]

______one hundred million won [Range: 1~99] ________ MW [Range: 0 ~ 9997] → F055

Don’t know →Go to F028~F032 unfolding

Refuse to answer →Go to F028~F032 unfolding

**< F028~F032. Unfolding bracket questions >**

[IWER: Go back to F027 and record if R answers the amount during the interview.]

F028. Does it amount to less than, about equal to or more than 10 MW (10,000 Korean won)?

① Less than 10 MW

③ About 10 MW

⑤ More than 10 MW

F029. Does it amount to less than, about equal to or more than 30 MW (10,000 Korean won)?

① Less than 30 MW

③ About 30 MW

⑤ More than 30 MW

F030. Does it amount to less than, about equal to or more than 50 MW (10,000 Korean won)?

① Less than 50 MW

③ About 50 MW

⑤ More than 50 MW

F031. Does it amount to less than, about equal to or more than 100 MW (10,000 Korean won)?

① Less than 100 MW

③ About 100 MW

⑤ More than 100 MW

F032. Does it amount to less than, about equal to or more than 300 MW (10,000 Korean won)?

① Less than 300 MW

③ About 300 MW

⑤ More than 300 MW

**< Housing (JEON-SE: Deposit-based rental home without monthly rent)>**

**Logic**

- **If F001=②, then ask F033.**

F033. Is the JEON-SE contract (of the home you are currently residing in) under your name?

1. Yes, R's name (including joint ownership)
2. No, other's name - spouse →Go to F057
3. No, other's name - (grand)children →Go to F057
4. No, other's name - other family members →Go to F057
5. No, other's name - other persons →Go to F057

F034. Is the JEON-SE contract under multiple persons’ names? If so, how many names are listed on the contract? (unit: a person)

[IWER: If R’s name is listed, he/she should be counted in. For example, R and his/her spouse are both listed on the home deed, enter 2. If the home is not under multiple persons’ names, enter 0.]

_______________________________ people [Range: 0~20]

F035. How much JEON‐SE security deposit did you pay on your current home? (unit:10,000 Korean won)

[IWER: Please enter ‘100,000,000 Korean won (one hundred million won)’ first then, enter ’10,000 Korean won (MW)’.]

______one hundred million won [Range: 1~99] ________ MW [Range: 0 ~ 9997] → F055

Don’t know →Go to F036~F040 unfolding

Refuse to answer →Go to F036~F040 unfolding

**< F036~F040. Unfolding bracket questions >**

[IWER: Go back to F035 and record if R answers the amount during the interview.]

F036. Did it amount to less than, about equal to or more than 1,000 MW (10,000 Korean won)?

① Less than 1,000 MW

③ About 1,000 MW

⑤ More than 1,000 MW

F037. Did it amount to less than, about equal to or more than 3,000 MW (10,000 Korean won)?

① Less than 3,000 MW

③ About 3,000 MW

⑤ More than 3,000 MW

F038. Did it amount to less than, about equal to or more than 5,000 MW (10,000 Korean won)?

① Less than 5,000 MW

③ About 5,000 MW

⑤ More than 5,000 MW

F039. Did it amount to less than, about equal to or more than 10,000 MW (10,000 Korean won)?

① Less than 10,000 MW

③ About 10,000 MW

⑤ More than 10,000 MW

F040. Did it amount to less than, about equal to or more than 30,000 MW (10,000Korean won)?

① Less than 30,000 MW

③ About 30,000 MW

⑤ More than 30,000 MW

**<Housing (WOL‐SE: Monthly rental home with or without a deposit)>**

**Logic**

- **If F001=③, then ask F041.**

F041. Is the WOL-SE contract (of the home you are currently residing in) under your name?

1. Yes, R's name (including joint ownership)
2. No, other's name – spouse →Go to F057
3. No, other's name - (grand)children →Go to F057
4. No, other's name - other family members →Go to F057
5. No, other's name - other persons →Go to F057

F042. Is the WOL-SE contract under multiple persons’ names? If so, how many names are listed on the contract? (unit: a person)

[IWER: If R’s name is listed, he/she should be counted in. For example, R and his/her spouse are both listed on the home deed, enter 2. If the home is not under multiple persons’ names, enter 0.]

_______________________________ people [Range: 0~20]

F043. How much WOL_SE security deposit did you pay on your current home? (unit: 10,000 Korean won)

[IWER: Please enter ‘100,000,000 Korean won (one hundred million won)’ first then, enter ’10,000 Korean won (MW)’.]

______one hundred million won [Range: 1~99] ________ MW [Range: 0 ~ 9997] → F049

Don’t know →Go to F044~F048 unfolding

Refuse to answer →Go to F044~F048 unfolding

**< F044 ~ F048. Unfolding bracket questions >**

[IWER: Go back to F043 and record if R answers the amount during the interview.]

F044. Did it amount to less than, about equal to or more than 300 MW (10,000 Korean won)?

① Less than 300 MW

③ About 300 MW

⑤ More than 300 MW

F045. Did it amount to less than, about equal to or more than 500 MW (10,000 Korean won)?

① Less than 500 MW

③ About 500 MW

⑤ More than 500 MW

F046. Did it amount to less than, about equal to or more than 1,000 MW (10,000 Korean won)?

① Less than 1,000 MW

③ About 1,000 MW

⑤ More than 1,000 MW

F047. Did it amount to less than, about equal to or more than 3,000 MW (10,000 Korean won)?

① Less than 3,000 MW

③ About 3,000 MW

⑤ More than 3,000 MW

F048. Did it amount to less than, about equal to or more than 6,000 MW (10,000 Korean won)?

① Less than 6,000 MW

③ About 6,000 MW

⑤ More than 6,000 MW

**Logic**

- **If F001=③ or ④, then ask F049.**

F049. How much rent do you pay per month? (unit: 10,000 Korean won)

_______________________________ MW [Range: 1~999999] →Go to F055

Don’t know →Go to F050~F054 unfolding

Refuse to answer →Go to F050~F054 unfolding

**< F050 ~ F054. Unfolding bracket questions >**

[IWER: Go back to F049 and record if R answers the amount during the interview.]

F050. Does it amount to less than, about equal to or more than 10 MW (10,000 Korean won)?

① Less than 10 MW

③ About 10 MW

⑤ More than 10 MW

F051. Does it amount to less than, about equal to or more than 30 MW (10,000 Korean won)?

① Less than 30 MW

③ About 30 MW

⑤ More than 30 MW

F052. Does it amount to less than, about equal to or more than 50 MW (10,000 Korean won)?

① Less than 50 MW

③ About 50 MW

⑤ More than 50 MW

F053. Does it amount to less than, about equal to or more than 100 MW (10,000 Korean won)?

① Less than 100 MW

③ About 100 MW

⑤ More than 100 MW

F054. Does it amount to less than, about equal to or more than 300 MW (10,000Korean won)?

① Less than 300 MW

③ About 300 MW

⑤ More than 300 MW

**Logic**

- **If R has moved to another home or the tenure type has changed and the price in the previous interview > the price in this interview (i.e. the price of home in this interview is more than 20% below that in the previous interview.), ask F055.**
- **If not, then ask F057.**

F055. The price of the home you are currently residing in has changed since the previous interview. What did you do with the difference? Choose the one for which you used the difference the most.

[IWER: If the incorrect answer in the previous interview caused the difference, enter ‘⑤ Others’ and then go to F056 and write that the previous interview is incorrect.]

| ITEMS | Previous interview | This interview (2018) |
| --- | --- | --- |
| Type of housing tenure | [Own/Lease on a deposit basis without monthly rent(i.e. JEON-SE)/  Monthly rent with a deposit(i.e. JEON-WOL-SE)/Monthly rent without a deposit(i.e. WOL-SE)/Others] | [Own/Lease on a deposit basis without monthly rent(i.e. JEON-SE)/Monthly rent with a deposit(i.e. JEON-WOL-SE)/Monthly rent without a deposit(i.e. WOL-SE)/Other] |
| Value | [PL1] MW | [PL2] MW |
| Difference | [PL1-PL2] MW | |

1. Invested in financial assets or savings accounts →Go to F057
2. Invested in other business →Go to F057
3. Used for living expenses →Go to F057
4. Inheritance/ Give →Go to F057
5. Others

F056. If others, please specify.

_______________________________

**< Real Estate Property>**

F057. Aside from the current home you are residing in, do you have any other real estate properties, such as other home/ second home, buildings, commercial property, studio apartments, land, pre-sale rights for apartments or studio apartments, or condominium membership? Please exclude the home you are currently living in.

1. Yes ⑤ No →Go to F078

F058. If you sold those real estate properties except the home you are currently residing in, how much would they cost? (unit: 10,000 Korean won)

[IWER: Please enter ‘100,000,000 Korean won (one hundred million won)’ first then, enter ’10,000 Korean won (MW)’.]

______one hundred million won [Range: 1~999] _______ MW [Range: 0 ~ 9997] → F064

Don’t know →Go to F059~F063 unfolding

Refuse to answer →Go to F059~F063 unfolding

**< F059~F063. Unfolding bracket questions >**

[IWER: Go back to F058 and record if R answers the amount during the interview.]

F059. Would it amount to less than, about equal to or more than 1,000 MW (10,000 Korean won)?

① Less than 1,000 MW

③ About 1,000 MW

⑤ More than 1,000 MW
[truncated: 135,282 more chars]
