# Supplementary material for: Explainable artificial intelligence on life satisfaction, diabetes mellitus and its comorbid condition
Source: Sci Rep. 2023 Jul 19;13:11651. doi: 10.1038/s41598-023-36285-z (PMC10356918; doi:10.1038/s41598-023-36285-z)
Supplement: Supplementary file 2 — Supplementary Tables. [file 41598_2023_36285_MOESM2_ESM.doc]

Table S1. Descriptive Statistics for Participants’ Categorical Variables for Year 2018/2016

| **Variable** | *Count* | *Percentage (%)* |
| --- | --- | --- |
|  |  |  |
| Comorbidity of Diabetes (in 2018) |  |  |
| Cancer |  |  |
| NN^a^ | 3995 | 72.3 |
| YN^b^ | 1133 | 20.5 |
| NY | 299 | 5.4 |
| YY | 100 | 1.8 |
| Heart Disease |  |  |
| NN^c^ | 3906 | 70.7 |
| YN^d^ | 1016 | 18.4 |
| NY | 388 | 7.0 |
| YY | 217 | 3.9 |
| Mental Disease |  |  |
| NN^e^ | 4066 | 73.6 |
| YN^f^ | 1158 | 21.0 |
| NY | 228 | 4.1 |
| YY | 75 | 1.4 |
|  |  |  |
| Education (in 2016 Hereafter) |  |  |
| Elementary or Below | 2482 | 44.9 |
| Junior High | 1604 | 29.0 |
| Senior High | 977 | 17.7 |
| College or Above | 464 | 8.4 |
|  |  |  |
| Gender |  |  |
| Male | 3170 | 57.4 |
| Female | 2357 | 42.6 |
|  |  |  |
| Marriage |  |  |
| Married | 4133 | 74.8 |
| Separated | 1212 | 21.9 |
| Divorced | 114 | 2.1 |
| Widowed | 36 | 0.7 |
| Unmarried | 32 | 0.6 |
|  |  |  |
| Religion |  |  |
| Non | 3161 | 57.2 |
| Protestant | 992 | 17.9 |
| Catholic | 945 | 17.1 |
| Buddhist | 391 | 7.1 |
| Won-Buddhist | 25 | 0.5 |
| Other | 13 | 0.2 |
|  |  |  |
| Residential Type |  |  |
| Apartment | 3303 | 59.8 |
| Other | 2224 | 40.2 |
|  |  |  |
| Region |  |  |
| Urban, Big | 2271 | 41.1 |
| Urban, Small | 1771 | 32.0 |
| Rural | 1485 | 26.9 |
|  |  |  |
| Parents Alive |  |  |
| Father & Mother | 190 | 3.4 |
| Father | 82 | 1.5 |
| Mother | 753 | 13.6 |
| None | 4502 | 81.5 |
|  |  |  |
| Health Insurance |  |  |
| Medicare | 5245 | 94.9 |
| Medicaid | 282 | 5.1 |
|  |  |  |
| Economic Activity |  |  |
| Employed | 2026 | 36.7 |
| Unemployed | 3501 | 63.3 |
|  |  |  |
| Subjective Health |  |  |
| Very Good | 42 | 0.8 |
| Good | 1424 | 25.8 |
| Middle (Neither Good nor Poor) | 2509 | 45.4 |
| Poor | 1264 | 22.9 |
| Very Poor | 288 | 5.2 |
|  |  |  |
| Smoker |  |  |
| Non | 3808 | 68.9 |
| Former | 1145 | 20.7 |
| Current | 574 | 10.4 |
|  |  |  |
| Drinker |  |  |
| Non | 1772 | 32.1 |
| Former | 936 | 16.9 |
| Current | 2819 | 51.0 |
|  |  |  |
| Subjective Class |  |  |
| High-A | 38 | 0.7 |
| High-B | 123 | 2.2 |
| Middle-A | 944 | 17.1 |
| Middle-B | 1913 | 34.6 |
| Low-A | 1526 | 27.6 |
| Low-B | 983 | 17.8 |
|  |  |  |
| Diabetes Miletus |  |  |
| Yes | 1138 | 20.6 |
| No | 4389 | 79.4 |
|  |  |  |
| Cancer |  |  |
| Yes | 344 | 6.2 |
| No | 5183 | 93.8 |
|  |  |  |
| Heart Disease |  |  |
| Yes | 553 | 10.0 |
| No | 4974 | 90.0 |
|  |  |  |
| Mental Disease |  |  |
| Yes | 273 | 4.9 |
| No | 5254 | 95.1 |
|  |  |  |

| a | NN for | Diabetes Milieus No | Cancer No |  |
| --- | --- | --- | --- | --- |
| b | YN for | Diabetes Milieus Yes | Cancer No |  |
| c | NN for | Diabetes Milieus No | Heart Disease No |  |
| d | YN for | Diabetes Milieus Yes | Heart Disease No |  |
| e | NN for | Diabetes Milieus No | Mental Disease No |  |
| f | YN for | Diabetes Milieus Yes | Mental Disease No |  |

Table S2. Descriptive Statistics for Participants’ Continuous Variables for Year 2016

| **Variable** | *Mean* | *SD* | *Min* | *25%* | *50%* | *75%* | *Max* |
| --- | --- | --- | --- | --- | --- | --- | --- |
|  |  |  |  |  |  |  |  |
| Age | 70.87 | 9.15 | 56 | 63 | 70 | 78 | 101 |
| Activity - Religious | 0.46 | 1.19 | 0 | 0 | 0 | 0 | 10 |
| Activity - Friendship | 2.40 | 2.28 | 0 | 0 | 2 | 4 | 10 |
| Activity - Leisure | 0.20 | 0.91 | 0 | 0 | 0 | 0 | 10 |
| Activity - Family | 0.79 | 1.95 | 0 | 0 | 0 | 0 | 9 |
| Activity - Voluntary | 0.03 | 0.34 | 0 | 0 | 0 | 0 | 8 |
| Activity - Political | 0.01 | 0.20 | 0 | 0 | 0 | 0 | 7 |
| # Children Alive | 2.93 | 1.40 | 0 | 2 | 3 | 4 | 9 |
| # Brothers/Sisters Cohabiting | 0.02 | 0.16 | 0 | 0 | 0 | 0 | 5 |
| Income (Monthly, $) | 1205.39 | 1844.58 | 5 | 250 | 630 | 1571 | 60000 |
| Body Mass Index | 23.92 | 6.15 | 2 | 22 | 23 | 25 | 114 |
| Life Satisfaction - Health | 58.05 | 19.46 | 0 | 50 | 60 | 70 | 100 |
| Life Satisfaction - Economic | 55.63 | 19.28 | 0 | 40 | 60 | 70 | 100 |
| Life Satisfaction - Overall | 62.36 | 15.91 | 0 | 50 | 60 | 70 | 100 |
|  |  |  |  |  |  |  |  |

Table S3. Logistic Regression Results: Odds Ratios

| **Independent Variable** | *Diabetes-Cancer* | |  | *Diabetes-Heart Disease* | | | *Diabetes-Mental Disease* | | |
| --- | --- | --- | --- | --- | --- | --- | --- | --- | --- |
|  | *YN^a^* | *NY* | *YY* | *YN* | *NY* | *YY* | *YN* | *NY* | *YY* |
| Education (in 2016 Hereafter) |  |  |  |  |  |  |  |  |  |
| Elementary, Below |  |  |  |  |  |  |  |  |  |
| Junior High | 1.3154 | 1.0538 | 1.2960 | 1.3287 | 0.8638 | 3.0322 | 1.2042 | 0.0000 | 0.0008 |
| Senior High | 0.7348 | 0.5729 | 1.7471 | 0.8200 | 1.1616 | 1.3609 | 0.5754 | 4.2063 | 13.0357 |
| College, Above | 1.5799 | 0.3363 | 2.4780 | 1.5773 | 3.0839 | 4.2465 | 1.2335 | 6.8687 | 71.0737 |
| Gender |  |  |  |  |  |  |  |  |  |
| Male |  |  |  |  |  |  |  |  |  |
| Female | 0.7377 | 1.2201 | 1.2071 | 1.3419 | 1.1417 | 18.5406 | 1.0619 | 16.1370 | 0.1048 |
| Age | 0.9971 | 1.0416 | 1.0197 | 1.0216 | 1.0554 | 1.0755 | 1.0295 | 1.1159 | 0.8664 |
| Marriage |  |  |  |  |  |  |  |  |  |
| Married |  |  |  |  |  |  |  |  |  |
| Separated | 0.0042 | 0.0089 | 0.0000 | 0.0415 | 0.0341 | 0.0043 | 0.0000 | 0.0000 | 0.0000 |
| Divorced | 0.4838 | 6.7493 | 0.0002 | 1.5266 | 0.0132 | 0.0000 | 2.2618 | 0.0000 | 0.0000 |
| Widowed | 1.3123 | 0.4863 | 0.4227 | 0.8919 | 1.1668 | 0.3714 | 0.6294 | 0.1237 | 0.5900 |
| Unmarried | 0.0071 | 0.0076 | 0.0003 | 0.0265 | 0.0261 | 0.0000 | 0.0002 | 2.4193 | 0.0000 |
| Religion |  |  |  |  |  |  |  |  |  |
| Non |  |  |  |  |  |  |  |  |  |
| Protestant | 2.4125 | 1.7811 | 9.1664 | 1.7494 | 1.3186 | 3.5011 | 2.1422 | 2.1164 | 63.4109 |
| Catholic | 1.5020 | 0.1162 | 2.8946 | 1.8743 | 1.0390 | 1.8828 | 1.9576 | 0.2386 | 139.8174 |
| Buddhist | 1.9991 | 0.5309 | 2.2384 | 1.5570 | 0.7174 | 1.3756 | 1.6742 | 1.7094 | 46.2139 |
| Won-Buddhist | 0.0512 | 0.0159 | 0.1098 | 0.1023 | 0.0013 | 0.0278 | 0.0000 | 0.0000 | 0.3917 |
| Other | 0.0000 | 9.1988 | 1.9921 | 0.0005 | 0.0004 | 0.6308 | 0.0000 | 31.8008 | 27.4666 |
| Activity - Religious | 0.8958 | 1.2111 | 0.7855 | 0.8641 | 1.0636 | 1.3418 | 0.9305 | 1.1821 | 0.4898 |
| Activity - Friendship | 0.9050 | 1.1058 | 1.1084 | 0.9494 | 1.1372 | 1.1796 | 0.9715 | 1.0505 | 0.9598 |
| Activity - Leisure | 0.8509 | 1.2891 | 0.1423 | 0.7644 | 0.4572 | 0.1227 | 0.9263 | 1.0495 | 0.2234 |
| Activity - Family | 1.0984 | 1.2179 | 1.0650 | 1.0715 | 0.8054 | 0.7478 | 1.0716 | 1.0245 | 0.3532 |
| Activity - Voluntary | 0.4017 | 1.3827 | 0.1338 | 0.3860 | 0.2475 | 0.0847 | 0.0423 | 0.2026 | 0.0552 |
| Activity - Political | 0.5267 | 0.0444 | 0.0006 | 0.8649 | 0.5005 | 0.4274 | 0.6086 | 0.0236 | 2.7997 |
| Residential Type |  |  |  |  |  |  |  |  |  |
| Apartment |  |  |  |  |  |  |  |  |  |
| Other | 0.7662 | 0.8366 | 0.3120 | 0.6699 | 1.2718 | 0.1616 | 0.5490 | 0.5252 | 0.5751 |
| Region |  |  |  |  |  |  |  |  |  |
| Urban, Big |  |  |  |  |  |  |  |  |  |
| Urban, Small | 0.9518 | 1.1694 | 1.6525 | 0.7659 | 2.3276 | 0.7256 | 0.7417 | 1.7610 | 17.3638 |
| Rural | 0.4404 | 0.4679 | 0.1808 | 0.5421 | 1.0505 | 0.4931 | 0.3974 | 1.0788 | 1.9123 |
| # Children Alive | 1.0407 | 1.0863 | 1.0722 | 0.9591 | 1.0598 | 0.6539 | 0.9530 | 0.9890 | 1.9073 |
| # Brothers/Sisters Cohabiting | 0.9330 | 0.0156 | 0.0020 | 1.3617 | 0.0513 | 0.0023 | 1.3661 | 5.7614 | 0.0000 |
| Parents Alive |  |  |  |  |  |  |  |  |  |
| Father & Mother |  |  |  |  |  |  |  |  |  |
| Father | 5.6370 | 3.4976 | 0.4835 | 40.4984 | 435.5579 | 24535.1447 | 3.5442 | 433.2413 | 151.2626 |
| Mother | 2.8237 | 292.1582 | 43.6685 | 17.9150 | 185.7501 | 814.2460 | 2.5541 | 129144.5000 | 915.0425 |
| None | 1.8316 | 275.4366 | 1461.9817 | 13.6599 | 155.1851 | 553.0411 | 1.9231 | 217763.2000 | 15.5844 |
| Health Insurance |  |  |  |  |  |  |  |  |  |
| Medicare |  |  |  |  |  |  |  |  |  |
| Medicaid | 1.0780 | 0.3563 | 0.2158 | 1.3833 | 2.5978 | 4.1701 | 1.2090 | 4.5209 | 595.6843 |
| Economic Activity |  |  |  |  |  |  |  |  |  |
| Employed |  |  |  |  |  |  |  |  |  |
| Unemployed | 1.5780 | 0.6740 | 12.3149 | 0.7921 | 0.3939 | 1.7601 | 0.8920 | 0.7592 | 83995.3600 |
| Income (Monthly, $) | 0.1335 | 0.0000 | 17.3510 | 28.3352 | 0.0000 | 1172974.0000 | 47.7114 | 22673.1100 | 0.0000 |
| Subjective Health |  |  |  |  |  |  |  |  |  |
| Very Good |  |  |  |  |  |  |  |  |  |
| Good | 0.4081 | 4.1501 | 0.1573 | 86.8522 | 27.9087 | 1.6055 | 0.5655 | 1422776 | 20.2165 |
| Middle | 0.5523 | 10.6614 | 186.8836 | 130.2165 | 37.2964 | 5.6172 | 0.4971 | 2690232 | 511.8356 |
| Poor | 0.4510 | 6.5308 | 213.2079 | 132.4108 | 30.7614 | 13.2739 | 0.4197 | 7357549 | 10303.0100 |
| Very Poor | 0.3831 | 3.4300 | 142.8822 | 197.9649 | 157.5514 | 78.3270 | 0.6141 | 54340640 | 84160.0000 |
| Body Mass Index | 1.0325 | 0.9164 | 0.9785 | 1.0302 | 0.9929 | 1.0223 | 1.0391 | 0.9479 | 0.8196 |
| Smoker |  |  |  |  |  |  |  |  |  |
| Non |  |  |  |  |  |  |  |  |  |
| Former | 1.4547 | 1.7942 | 0.7698 | 1.5904 | 1.0127 | 9.4104 | 1.6929 | 5.2608 | 20.0850 |
| Current | 1.6536 | 2.0388 | 1.1990 | 1.6196 | 0.8048 | 0.5622 | 1.3926 | 5.5238 | 2.0677 |
| Drinker |  |  |  |  |  |  |  |  |  |
| Non |  |  |  |  |  |  |  |  |  |
| Former | 1.0186 | 1.5153 | 0.4978 | 1.0060 | 1.0675 | 0.3447 | 0.9384 | 0.7903 | 0.9291 |
| Current | 0.9124 | 0.6163 | 0.4873 | 0.6700 | 0.8116 | 0.0958 | 0.9202 | 0.4177 | 2.2941 |
| Life Satisfaction - Health | 0.9962 | 0.9701 | 1.0289 | 1.0048 | 1.0104 | 0.9864 | 0.9951 | 1.0246 | 1.0742 |
| Life Satisfaction - Economic | 1.0049 | 1.0129 | 0.9911 | 1.0024 | 0.9986 | 1.0248 | 0.9974 | 0.9975 | 1.0633 |
| Life Satisfaction - Overall | 0.9887 | 1.0222 | 0.9811 | 0.9925 | 1.0208 | 1.0103 | 1.0028 | 0.9977 | 0.8718 |
| Subjective Class |  |  |  |  |  |  |  |  |  |
| High-A |  |  |  |  |  |  |  |  |  |
| High-B | 25.7401 | 0.1382 | 0.0024 | 33.4950 | 4.1180 | 186.8142 | 709543500 | 772 | 0 |
| Middle-A | 42.9354 | 41.1473 | 96.6587 | 22.6863 | 5.8164 | 34.1556 | 429246600 | 79822490 | 1534 |
| Middle-B | 50.7448 | 80.5796 | 303.8731 | 25.4059 | 7.9603 | 66.5861 | 586639766 | 466687987 | 384222 |
| Low-A | 42.8769 | 47.6650 | 155.9326 | 30.5756 | 12.3102 | 190.2298 | 475729584 | 414289394 | 816168 |
| Low-B | 44.8024 | 31.6025 | 448.7620 | 43.0324 | 14.4727 | 89.3898 | 613485255 | 709991326 | 20619 |
| Diabetes Miletus |  |  |  |  |  |  |  |  |  |
| Yes |  |  |  |  |  |  |  |  |  |
| No | 0.0000 | 11.7713 | 0.0000 | 0.0000 | 866.7322 | 0.0000 | 0.0000 | 4.7753 | 0.0000 |
| Cancer |  |  |  |  |  |  |  |  |  |
| Yes |  |  |  |  |  |  |  |  |  |
| No | 330.1985 | 0.0000 | 0.0000 |  |  |  |  |  |  |
| Heart Disease |  |  |  |  |  |  |  |  |  |
| Yes |  |  |  |  |  |  |  |  |  |
| No |  |  |  | 1355.6580 | 0.0000 | 0.0000 |  |  |  |
| Mental Disease |  |  |  |  |  |  |  |  |  |
| Yes |  |  |  |  |  |  |  |  |  |
| No |  |  |  |  |  |  | 11355520000 | 0 | 0 |
|  |  |  |  |  |  |  |  |  |  |

Note: ^a^ YN Diabetes Yes Cancer No, NY Diabetes No Cancer Yes, YY Diabetes Yes Cancer Yes (Reference: Diabetes No Cancer No)
